# Atomically precise copper clusters with dual sites for highly chemoselective and efficient hydroboration

**DOI:** 10.1038/s41467-024-53950-7

**Published:** 2024-11-05

**Authors:** Teng Jia, Jie Ai, Xiaoguang Li, Miao-Miao Zhang, Yue Hua, Yi-Xin Li, Cai-Fang Sun, Feng Liu, Ren-Wu Huang, Zheng Wang, Shuang-Quan Zang

**Affiliations:** 1 Henan Key Laboratory of Crystalline Molecular Functional Materials and College of Chemistry, Zhengzhou University, Zhengzhou, 450001 China; 2 Institute for Advanced Study, Shenzhen University, Shenzhen, 518060 China; 3 School of Chemistry and Chemical Engineering, Henan University of Technology, Zhengzhou, 450001 China; 4 Yunnan Precious Metals Lab Co., LTD, Kunming, 650106 China

**Keywords:** Organometallic chemistry, Crystal engineering

## Abstract

The hydroboration of alkynes into vinylboronate esters is a vital transformation, but achieving high chemoselectivity of targeted functional groups and an appreciable turnover number is a considerable challenge. Herein, we develop two dynamically regulating dual-catalytic-site copper clusters (Cu_4_NC and Cu_8_NC) bearing N-heterocyclic thione ligands that endow Cu_4_NC and Cu_8_NC catalysts with performance. In particular, the performance of microcrystalline Cu_4_NC in hydroboration is characterized by a high turnover number (77786), a high chemoselectivity, high recovery and reusability under mild conditions. Mechanistic studies and density functional theory calculations reveal that, compared with the Cu_8_NC catalyst, the Cu_4_NC catalyst has a lower activation energy for hydroboration, accounting for its high catalytic activity. This work reveals that precisely constructed cluster catalysts with dual catalytic sites may provide a way to substantially improve catalytic properties by fully leveraging synergistic interactions and dynamic ligand effects, thus promoting the development of cluster catalysts.

## Abstract (web summary)

Alkyne hydroboration into vinylboronate esters is an important reaction, yet reaching high selectivity of targeted functional groups is challenging. Here, the authors develop dual-site copper clusters to achieve highly selective hydroboration.”

## Introduction

**Fig. 1:**
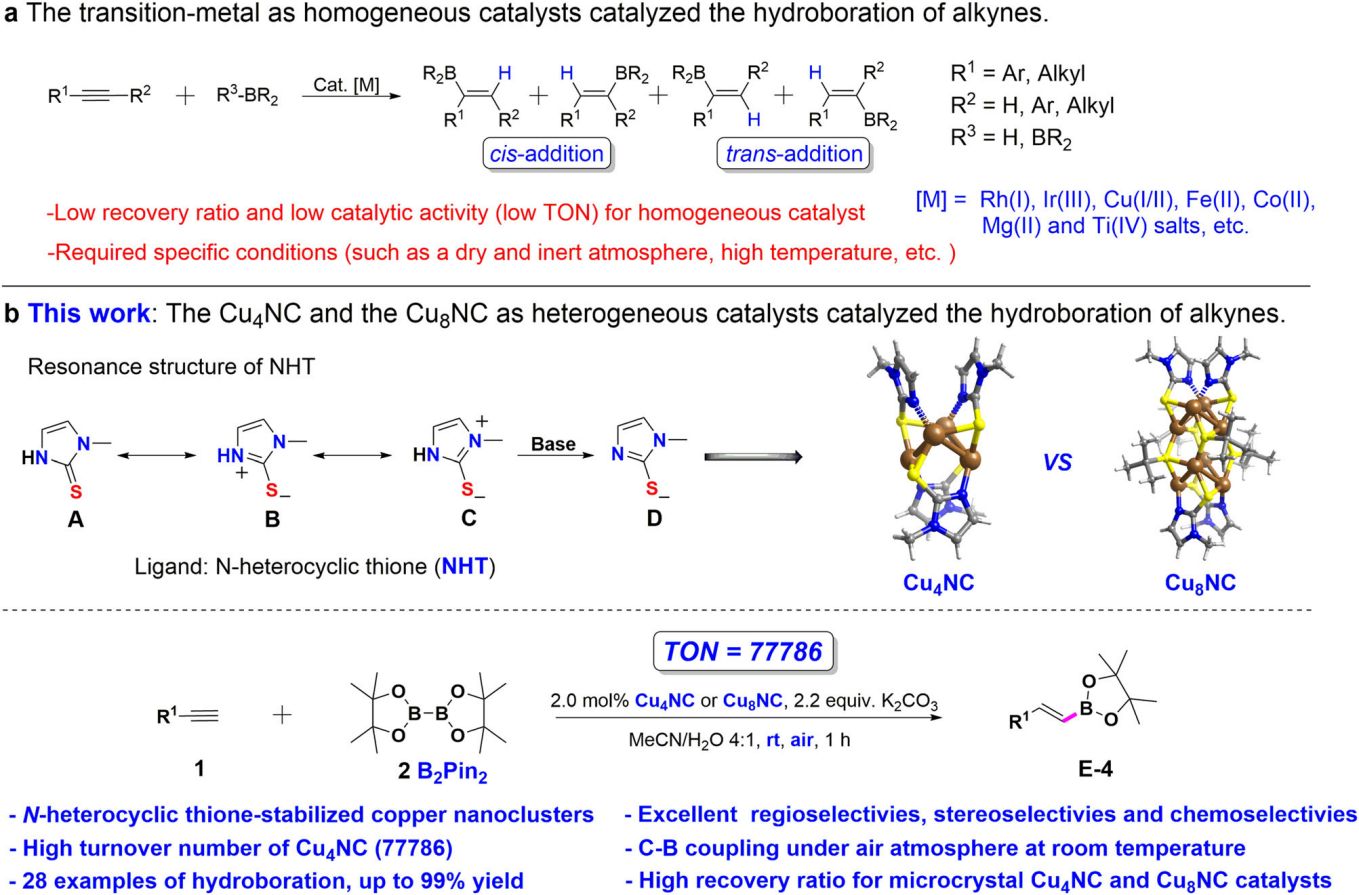
Representative synthesis strategies for vinylboronate esters. **a** Transition metal-catalyzed hydroboration of alkynes. **b** This work: microcrystalline Cu_4_NC and Cu_8_NC, as heterogeneous catalysts, catalyzed the hydroboration of alkynes. R_1_ represents the type of functional group.

Vinylboron compounds are potential multifunctional chemical targets that can be used to directly construct various complex organic molecules in a streamlined and efficient manner and are thus highly important compounds in various areas of chemistry^1–4^, particularly in materials science, pharmaceuticals and organic synthesis, where these compounds have been used in the Suzuki−Miyaura cross−coupling^5^, boronic acid Mannich (BAM)^6^, Chan−Lam coupling^7^ and Hayashi−Miyaura^8^ reactions. Various approaches for synthesizing vinylboron compounds via the hydroboration of alkynes have been investigated. To achieve regioselective and stereoselective synthesis of vinylboronate esters, diverse transition metal catalysts, particularly copper complexes, have been utilized to produce organic boron building blocks, but the turnover number (TON) for hydroboration is usually <5000 under specific conditions, such as dry and inert atmospheres (Fig. 1a)^9–13^. In addition, the lack of specificity in terms of the functional group reactivity during hydroboration is also a challenge. These challenges occur because the existing transition metal complexes contain a single catalytic center that simultaneously activates alkyne substrates and boron reagents to afford boron compounds, thus greatly limiting the catalytic activity of the transition metal complexes. Dual-catalytic-site catalysts, which can exhibit enhanced catalytic activity through exposed atomic-scale interfaces and the synergistic effect between two contiguous catalytic sites, have been widely applied to various types of catalysis^14–18^. The use of dual-catalytic-site catalysts is a good strategy for overcoming the challenges associated with hydroboration. For example, Li^19^ reported that metal−organic framework nanosheets, including dual-catalytic-site complexes containing Cu and N, efficiently promoted hydroboration with a high TON, further illustrating that synergistic interactions can tune the catalytic properties. However, the construction of precisely regulated dual-catalytic-site catalysts that fully leverage these synergistic effects to achieve improved catalytic properties remains challenging.

Metal nanoclusters (NCs) containing multiple metal atoms, which have been rapidly developed in the field of chemical catalysis^20–23^, are important materials for designing and developing efficient dual-catalytic-site catalysts. Owing to the good chemical purity, well-defined crystal structures, high surface-to-volume ratios, and homogeneous distribution of catalytic sites of such NCs, their targeted modification is convenient and is beneficial for establishing a solid correlation between their structure and performance^24–28^. Compared with traditional dual-catalytic-site catalysts, NCs with dual catalytic sites have the following advantages: (I) Cluster kernels are constructed through metal-metal bonds between different metal atoms, which is beneficial for regulating the catalytic activity^21,24,28^. (II) Dual-catalytic-site NCs protected by dynamic ligands can prevent catalyst passivation and deactivation to maintain their catalytic activity, thus helping to increase the substrate tolerance. (III) Dissociated dynamic ligands are convenient for regulating the chemical environment around dual catalytic sites, laying a solid foundation for improving the selectivity of catalysts. Nevertheless, in previous catalytic studies, the ligands of NCs were usually removed to expose more catalytic sites, leading to changes in the cluster structures and aggregation of clusters, which decreased the structural uniformity. If the dynamic coordination properties of the cluster shell ligands can be effectively utilized to achieve precise dual-catalytic-site control while maintaining the integral cluster structure, then ligand-protected NCs should be the most ideal catalysts. The stability of copper NCs is worse than that of gold NCs and silver NCs, which has caused their development to be slower than that of other materials. Improving the stability of copper NCs remains challenging. The versatile ligands of N-heterocyclic thiones (NHTs) with zwitterionic resonance structures (Fig. 1b) have been widely applied to various transition metal nanoparticles and homogeneous catalysts to catalyze various kinds of chemical reactions^29–33^. Owing to the ease with which the steric and electronic effects of NHTs can be tuned, the good stability of NHTs and the formation of stable Cu-S bonds between NHT ligands and copper atoms, NHT ligands are ligand stabilizers for copper NC catalysts that can improve the catalytic activity via ligand engineering strategies^32–35^. To date, copper NCs with dual catalytic sites as heterogeneous catalysts have not been reported to catalyze the highly efficient and highly chemoselective hydroboration of alkynes under mild conditions.

Therefore, we designed the bidentate NHT ligand methimazole as a shell ligand for copper NCs. Dynamically regulating dual-catalytic-site (DRDS) copper clusters [Cu_4_(NHT)_4_] (Cu_4_NC) and [Cu_8_(NHT)_4_(^*t*^BuS)_4_] (Cu_8_NC) were efficiently synthesized in the gram scale. The bidentate NHT ligand participates in two different types of bonding modes in the copper clusters, i.e., a reversible Cu-N bond and a stable Cu-S bond between the shell ligands and copper cluster cores, which are beneficial for improving the stability and maintaining the catalytic activity of the clusters. As expected, the microcrystalline Cu_4_NC catalyst provided a solid foundation for achieving high efficiency (turnover number (TON) = 77786) (Supplementary Table 1), and regio-, stereo- and chemoselectivity in hydroboration at room temperature in an air atmosphere (Fig. 1b). Microcrystalline Cu_4_NC and Cu_8_NC catalysts were also recovered and reused. Through single-crystal structure analysis, control experiments, in situ characterization and density functional theory (DFT) calculations, the dual catalytic sites of Cu_4_NC and Cu_8_NC were further characterized. The hydroboration mechanisms were well elucidated, effectively presenting the relationships between the cluster structure and performance.

## Results

### Catalyst development and characterization

**Fig. 2:**
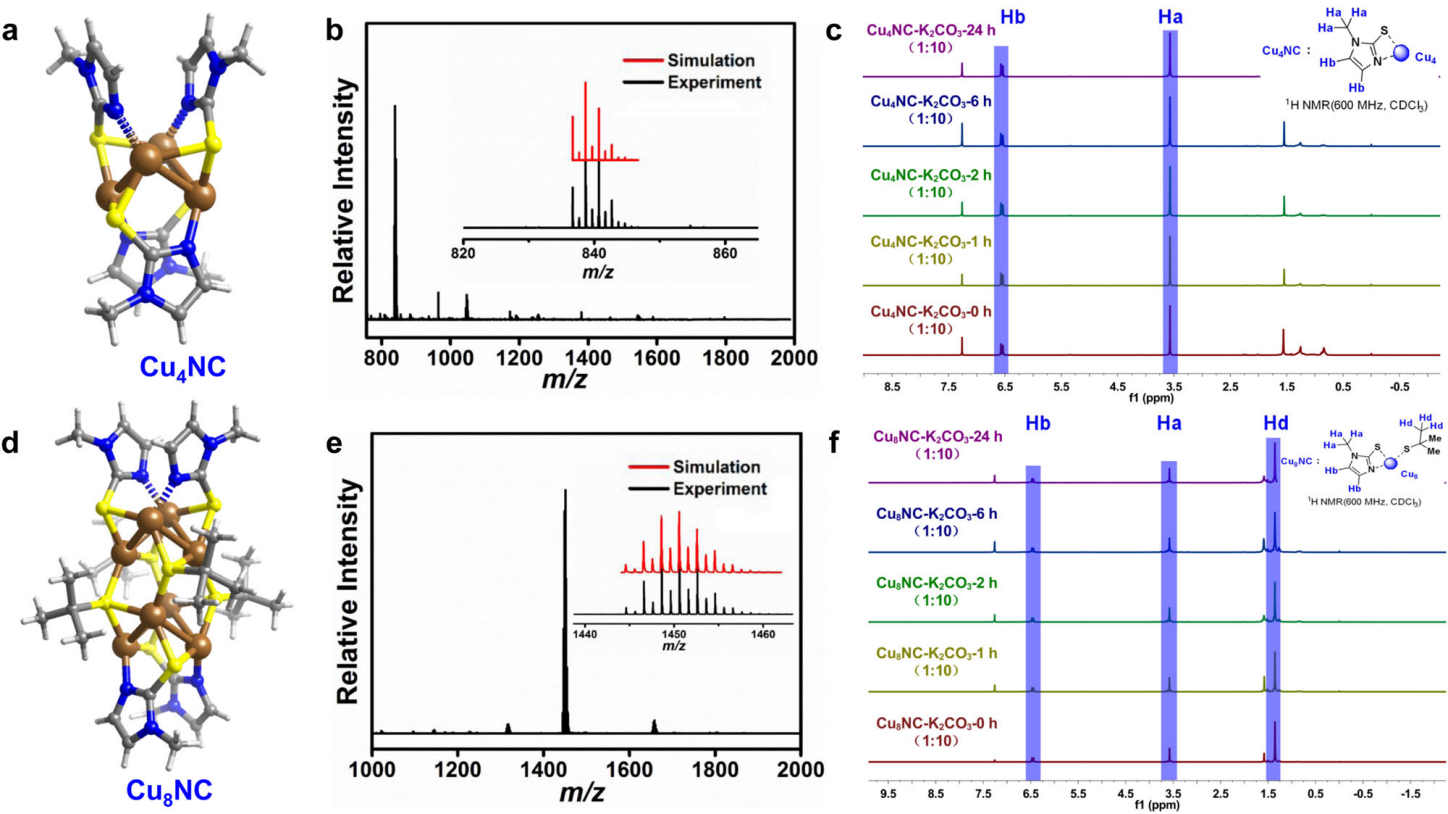
Schematic illustration and characterization of Cu_4_NC and Cu_8_NC. **a** Structure of Cu_4_NC. Color legend: brown, Cu; blue, N; yellow, S; gray, C; and white, H. **b** ESI‒MS spectra of Cu_4_NC dissolved in dimethyl sulfoxide (DMSO) solution and measured in positive ion mode. Inset: experimental (black) and simulated (red) isotopic distributions of Cu_4_NC. **c** ^1^H NMR spectra of Cu_4_NC-K_2_CO_3_ (1:10) in CDCl_3_ (0–24 h). Ha and Hb represent the characteristic H atoms of Cu_4_NC. **d** Structure of Cu_8_NC. Color legend: brown, Cu; blue, N; yellow, S; gray, C; and white, H. **e** ESI‒MS spectra of Cu_8_NC dissolved in DMSO solution and measured in positive ion mode. Inset: experimental (black) and simulated (red) isotopic distributions of Cu_8_NC. **f** ^1^H NMR spectra of Cu_8_NC-K_2_CO_3_ (1:10) in CDCl_3_ (0–24 h). Ha, Hb and Hd represent the characteristic H atoms of Cu_8_NC.

NHT ligands are classic ligand stabilizers and are air- and moisture-stable molecules. The resonance structures of NHT ligands can be drawn as zwitterions, with a negative charge located on the sulfur atom, which indicates that the sulfur anion has a higher associated electron density than a standard thiocarbonyl. The constructed conditions for Cu_4_NC and Cu_8_NC are under basic conditions. When NHT ligands are under basic conditions, they can undergo tautomerization, in which the thione form structure changes to an enol-like form structure (Fig. 1b, compound D), which implies that the electron density of the sulfur anion is further increased, endowing it with strong interactions with copper. The donation of a pair of electrons by the S anion, as an electron donor, to electron acceptors of copper cluster cores is beneficial for the construction of stable Cu-S bonds. The methimazole molecule, a classic NHT ligand, was designed as a model bidentate ligand with two different coordination modes: flexible and reversible Cu-N bonds and stable Cu-S bonds. The Cu_4_NC catalyst was synthesized via the reaction of bidentate NHT ligands and the copper salt [Cu(MeCN)_4_]PF_6_ under basic conditions through a one-pot method. Similarly, Cu_8_NC could be obtained by using ^*t*^BuSCu as the copper source instead of [Cu(MeCN)_4_]PF_6_ under basic conditions. Details of the synthesis are presented in the Supplementary Information. Compared with the Cu-N bonds, the Cu-S bonds were more stable. According to DFT calculations of the Cu-S and Cu-N bonds in Cu_4_NC and Cu_8_NC, the binding energies of the Cu-S bonds (−20.7 kcal/mol and −24.2 kcal/mol, respectively). were lower than the binding energies of Cu-N bonds (−15.0 kcal/mol and −17.6 kcal/mol, respectively) (Supplementary Fig. 71). This result indicates that the Cu-S bond is stronger than the Cu-N bond. Our findings align with previously reported results in the literature^25^, which also demonstrated that the Cu-N bond is weaker than the Cu-S bond. Single-crystal X-ray diffraction (SCXRD) results demonstrated that Cu_4_NC was composed of a Cu_4_ metal core bridged by four NHT ligands. The NHT ligands contained S and N donors, which could be firmly anchored on the surface of the Cu_4_NC cluster via Cu-S bonds and Cu-N bonds, further improving the rigidity and stability of Cu_4_NC (Fig. 2a, Supplementary Fig. 5). In the Cu_4_ unit, an average Cu-Cu distance of 2.662 Å was observed, which was less than twice the van der Waals radius (2 × 1.40 Å), indicating the occurrence of cuprophilic interactions^36,37^. Structurally, Cu_8_NC was found to contain a Cu_8_ metal core, four NHT ligands and four ^*t*^BuS- ligands (Fig. 2d, Supplementary Fig. 6). The skeleton of Cu_8_NC consisted of two identical Cu_4_ units, which were fused through four thiolate ligands, similar to a cage configuration. The Cu-Cu bond lengths ranged from 2.663 to 2.710 Å, suggesting the occurrence of metal-metal interactions. Furthermore, compared with the overall metal skeleton of Cu_4_NC, the thiolate ligands changed the inner structure from a Cu_4_ unit to two identical Cu_4_ units linked together by Cu-S interactions. SCXRD analysis revealed that the Cu-Cu distances were 2.662 Å and 2.663 Å in the Cu_4_NC unit and Cu_8_NC unit, respectively, which were slightly shorter than that of the tert-butoxide dicopper species^15^. This shorter distance is beneficial for the formation of key intermediates A and B to achieve synergistic effects on dual catalytic sites.

The research revealed that microcrystalline Cu_4_NC and Cu_8_NC have moderate solubilities in DCM, CHCl_3_ and DMSO solutions, respectively. To confirm the precise molecular masses and formulas of the copper NCs, the clusters were further characterized by electrospray ionization mass spectrometry (ESI‒MS) in positive ion mode in DMSO solution (Fig. 2b and e, Supplementary Figs. 9 and 10). The high-abundance peak at m/z 838.7003, can be assigned to [Cu_4_NC + Cs]^+^ (calcd m/z 838.6913) (Fig. 2b, Supplementary Fig. 9). The mass spectrum of the Cu_8_NC showed an intense peak at 1450.5869, corresponding to the molecular ion [Cu_8_NC + Cs]^+^ (calcd m/z 1450.5766) (Fig. 2e, Supplementary Fig. 10). The experimental isotopic distribution patterns of these copper NCs were in good agreement with the simulated results. The phase purities of Cu_4_NC and Cu_8_NC were demonstrated by elemental analysis, powder X-ray diffraction (PXRD) and ^1^H nuclear magnetic resonance (NMR) spectroscopy (Supplementary Figs. 7, 8, 13−15, 18−20). Energy-dispersive spectrometry (EDS) revealed the presence of the expected elements in microcrystalline Cu_4_NC and Cu_8_NC (Supplementary Figs. 11 and 12). The time-dependent UV−vis absorption spectra of these clusters remained unchanged in dichloromethane (DCM) for 72 h of treatment, which confirmed the good stability of the clusters in organic solution (Supplementary Figs. 25 and 26). Furthermore, microcrystalline Cu_4_NC and Cu_8_NC were also characterized by time-dependent NMR measurements (Fig. 2c and f, Supplementary Figs. 27−30). The ^1^H NMR spectra of Cu_4_NC and Cu_8_NC did not shift for 48 h or 24 h according to the in situ ^1^H NMR measurements of the copper NCs in CDCl_3_ or alkaline CDCl_3_, illustrating that these copper NCs were stable in neutral and alkaline organic systems (Fig. 2c and f, Supplementary Figs. 27−30). A series of characterizations of Cu_4_NC and Cu_8_NC fully illustrated that these copper NCs could be efficiently constructed and display good stability, validating the feasibility of using NHT as a bidentate protective agent to stabilize copper NCs.

### Development of the hydroboration of alkynes

To further explore the relationships between the copper NC structures and properties, microcrystalline Cu_4_NC and Cu_8_NC were used as catalysts to catalyze the hydroboration of alkynes. Initially, under an air atmosphere, the model substrate phenylacetylene (**1a**) (0.2 mmol, 1.0 eq), the microcrystalline Cu_4_NC catalyst (2.0 mol%), K_2_CO_3_ (0.44 mmol, 2.2 equiv.) and B_2_Pin_2_ (0.44 mmol, 2.2 eq) were added to a vial to react in a mixture solvent (MeCN/H_2_O) at room temperature for 30 min, which provided single vinylboron products with a good yield of 87% (Table 1, entry 2). It showed that microcrystalline Cu_4_NC can achieve high regio- and stereoselectivity in catalyzing hydroboration. A control experiment was carried out under a N_2_ atmosphere by substituting a Schlenk tube for the vial to maintain the airtightness of the catalytic system (Table 1, entry 3). The yield of and selectivity for vinylboron products were not obviously different between the control and experimental groups, illustrating that the microcrystalline Cu_4_NC catalytic system had the characteristics of high efficiency, high selectivity and strong tolerance to air and protic solvents during hydroboration. In addition, owing to the complete insolubility of microcrystalline Cu_4_NC in MeCN and H_2_O solutions, microcrystalline Cu_4_NC was considered to be a heterogeneous catalyst for hydroboration in the mixture solvent (MeCN/H_2_O) (Supplementary Figs. 16 and 17). Previously, most metal catalysts used to catalyze the hydroboration of alkynes with B_2_Pin_2_ were applied under an inert atmosphere^12,19,38–50^. Copper NCs used as heterogeneous catalysts, for highly efficient, highly regio- and stereoselective hydroboration of alkynes under mild conditions, have rarely been reported. To overcome this limitation, more investigations of using copper NCs as catalysts for catalyzing the hydroboration of alkynes were performed to construct a platform that can illustrate the precise relationships between copper NC catalysts and their catalytic properties.

**Table 1:** Optimization of hydroboration conditions^a,b^


Entry	Variations	Yield of E-4a
1	None	98%
2	30 min.	87%
3	N_2_, 30 min.	86%
4	N_2_	97%
5	Dark	95%
6	Cu_8_NC	64%
7	Cu(MeCN)_4_PF_6_	43%
8	4.0 mol% Cu powder	27%
9	HBpin instead of B_2_Pin_2_	n.r
10	Cs_2_CO_3_ instead of K_2_CO_3_	77%
11	Na_2_CO_3_ instead of K_2_CO_3_	70%
12	THF as solvent	trace
13	Dioxane as solvent	trace
14	EtOH as solvent	47%
15	DMF as solvent	trace
16	MeCN as solvent	trace
17	DCM/H_2_O as solvent	n.r
18	CHCl_3_/H_2_O as solvent	n.r
19	5.0 mol% NHT ligand	n.r
20	No K_2_CO_3_	n.r
21	No copper	n.r

^a^General reaction conditions: Phenylacetylene **1** (0.2 mmol, 1.0 equiv.), B_2_Pin_2_ **2** (0.44 mmol, 2.2 equiv.), microcrystalline Cu_4_NC catal. (0.004 mmol, 2.0 mol%) and K_2_CO_3_ (0.44 mmol, 2.2 equiv.) were added to the mixture solvent (2.0 mL, MeCN/H_2_O 4:1) under air atmosphere at room temperature and allowed to react for 1 h. ^b^The reported yield was determined by ^1^H NMR using 1,3,5-trimethoxybenzene as an internal standard.

To determine the optimal conditions, focused screening was performed, the results of which are summarized in Table 1. When the reaction time was prolonged to 1 h, vinylboronate ester **E-4a** was obtained under air or N_2_ atmosphere in a yield of 98% or 97%, respectively (Table 1, entries 1 and 4). When the hydroboration catalyzed by microcrystalline Cu_4_NC was carried out in the dark, little difference in the yield and selectivity was observed compared with the results obtained under natural light conditions (Table 1, entries 1 and 5). Since the intervention of light had no impact on the catalytic activity, the results showed that light irradiation was not a necessary condition for this organic transformation. The boron radical species might not be involved during the process supported by this reaction^43,51^. Similar to microcrystalline Cu_4_NC, Cu_8_NC was also completely insoluble under the same reaction conditions (Supplementary Figs. 21 and 22). Microcrystalline Cu_8_NC catalyzed the hydroboration of alkyne under the reaction condition described in entry 1, offering a vinylboronate ester product in moderate yield (64%) (Table 1, entry 6). These results further support that microcrystalline Cu_4_NC has a higher catalytic activity in hydroboration, which may originate from the lower activation energy for hydroboration of microcrystalline Cu_4_NC, compared to that of microcrystalline Cu_8_NC. Notably, when the catalyst loading of microcrystalline Cu_4_NC was reduced to 7.0 × 10^−4 ^ mmol, the customized cluster achieved a TON of up to 77786 (Supplementary Table 1), which was higher than those of catalysts reported for the hydroboration of alkynes (Supplementary Table 5)^38–51^.

As observed in the control experiments involving other homogeneous and heterogeneous copper salts, the desired products were obtained in poor yields (43% and 27%) under an air atmosphere at room temperature (Table 1, entries 7 and 8). When HBPin replaced B_2_Pin_2_ as the boron source for hydroboration, no reaction was observed under the optimal conditions (Table 1, entry 9). Different types of bases, such as Cs_2_CO_3_ and Na_2_CO_3_, were applied to this reaction, resulting in decreased yields (Table 1, entries 10 and 11). The influence of the solvents on the catalytic activity of microcrystalline Cu_4_NC was also investigated. Some solvents, including single strongly polar solvents, single weakly polar solvents and the mixture solvents, hindered the reaction process, resulting in a serious reduction in the yield or no reaction (Table 1, entries 12–18). Control experiments revealed that hydroboration could not be achieved when the NHT ligand was used as the catalyst, under neutral conditions or in the absence of copper catalysts (Table 1, entries 19–21). These results illustrated that the base is crucial for efficiently catalyzing this reaction under mild conditions and that the hydroboration of alkynes is not a spontaneous reaction under an air atmosphere at room temperature.

After the experiments, the microcrystalline Cu_4_NC or Cu_8_NC catalysts was filtered, dried and collected. The recycled microcrystalline copper NCs were further characterized via PXRD, UV−vis spectroscopy, ^1^H NMR spectroscopy and ESI‒MS (Supplementary Figs. 31–38), and the results revealed that the characteristic peaks of the copper NCs after catalysis well matched the characteristic peaks of the fresh copper NCs, confirming that the microcrystalline Cu_4_NC and Cu_8_NC remained intact under air, basic and organic solvents during hydroboration. The filtered supernatant was subsequently characterized via inductively coupled plasma mass spectrometry (ICP-MS), which revealed that no Cu^+^ ions were found in the reaction solution, suggesting that the Cu^+^ ions did not leach from the metal cluster catalysts into the solution over the course of the reaction. In addition, three parallel experiments involving hydroboration catalyzed by microcrystalline Cu_4_NC were also carried out. The parallel experiments were stopped at the 10th minute, 30th minute and 60th minute. The microcrystalline Cu_4_NC catalyst was filtered to recycle it. The recycled microcrystalline Cu_4_NC catalyst was further characterized via PXRD (Supplementary Fig. 96). The characteristic peaks of the microcrystalline Cu_4_NC after catalysis at different times well matched those of the microcrystalline Cu_4_NC before catalysis, confirming that structure of the recycled catalyst was maintained. These results further confirmed that microcrystalline Cu_4_NC and Cu_8_NC were stable catalysts.

**Fig. 3:**
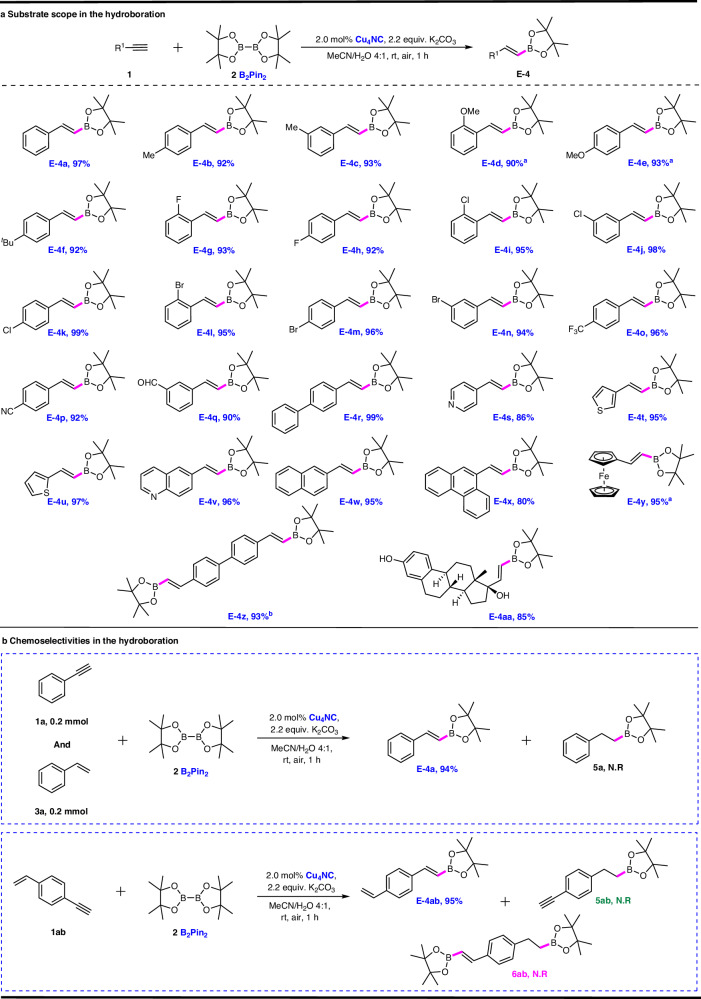
Performance of the Cu_4_NC-catalyzed hydroboration of alkynes. **a** Scope of alkynes for the Hydroboration. General reaction conditions: alkyne **1** (0.2 mmol, 1.0 equiv.), B_2_Pin_2_ **2** (0.44 mmol, 2.2 equiv.), the microcrystalline Cu_4_NC catal. (0.004 mmol, 2.0 mol%) and K_2_CO_3_ (0.44 mmol, 2.2 equiv.) were added to the mixture solvent (2.0 mL, MeCN/H_2_O 4:1) under air atmosphere at room temperature to react for 1 h. Isolated yields are given. ^a^Reaction time: 2 h. ^b^4,4’-Diethynyl-1,1’-biphenyl 1z (0.1 mmol) was used. **b** Investigation on chemoselectivity in the Cu_4_NC-catalyzed intermolecular and intramolecular hydroboration of alkynes and alkenes. Intermolecular hydroboration reaction conditions: phenylacetylene **1a** (0.2 mmol, 1.0 equiv.), styrene **3a** (0.2 mmol, 1.0 equiv.), B_2_Pin_2_ **2** (0.44 mmol, 2.2 equiv.), the microcrystalline Cu_4_NC catal. (0.004 mmol, 2.0 mol%) and K_2_CO_3_ (0.44 mmol, 2.2 equiv.) were added to the mixture solvent (2.0 mL, MeCN/H_2_O 4:1) under air atmosphere at room temperature to react for 1 h. Isolated yields are given. Intramolecular hydroboration reaction conditions: 1-ethynyl-4-vinylbenzene **1ab** (0.2 mmol, 1.0 equiv.), B_2_Pin_2_ **2** (0.44 mmol, 2.2 equiv.), the microcrystalline Cu_4_NC catal. (0.004 mmol, 2.0 mol%) and K_2_CO_3_ (0.44 mmol, 2.2 equiv.) were added to the mixture solvent (2.0 mL, MeCN/H_2_O 4:1) under air atmosphere at room temperature to react for 1 h. Isolated yields are given.

Under the optimal reaction conditions, the scope of alkyne substrates suitable for hydroboration transformations was investigated, and these results are summarized in Fig. 3. Various alkynes, including aryl and aliphatic alkynes, were considered as coupling partners with B_2_Pin_2_. For aryl alkynes (**1a–1r**), the catalytic system was quite well-tolerated, and performed well for electron-rich and electron-deficient aromatic substrates, resulting in yields (up to 99%). When 4-cyanophenylacetylene (**1p**) and 3-ethynylbenzaldehyde (**1q**) were used as alkyne substrates, vinylboronate esters **E-4p** and **E-4q**, respectively, were obtained as single products, which illustrated that microcrystalline Cu_4_NC was a highly regio-, stereo- and chemoselective heterogeneous catalyst capable of achieving highly chemoselective hydroboration between C ≡ C bonds and C ≡ N bonds (or C = O bonds). N-heterocyclic and S-heterocyclic aromatic alkynes also readily afforded single vinylboronate esters (**E-4s–E-4v**) in yields (86%-97%). Furthermore, aromatic alkynes possessing functional groups with high steric hindrance, ferrocene functional groups and diethynyl functional groups were investigated. The results showed that these substrates worked well, offering single organoboronate products (**E-4v–E-4z**) in yields (80%-96%). Ethynyl oestradiol, which is an aliphatic alkyne with high steric hindrance, also provided the desired single organoboronate compound (**E-4aa**) in yield of 85%. These results further support that reversible Cu-N bonds can dissociate from the metal core of Cu_4_NC to reduce the steric effect of the catalytic sites, thus improving the catalytic activity and enhancing the substrate adaptability. No reactions were observed with the internal alkynes bis(4-tolyl) acetylene, diphenylacetylene and 1-phenylpropyne, indicating a high activation barrier for internal alkynes.

The chemoselectivity between C ≡ C bonds and C = C bonds was further explored via control experiments catalyzed by microcrystalline Cu_4_NC. Equal amounts of phenylacetylene **1a** and styrene **3a** as substrates were added to the reaction system, and the reaction was catalyzed by the microcrystalline Cu_4_NC catalyst. Single vinylboronate ester **E-4a** was acquired in 94% yield, whereas alkylboronate ester **5a** was not observed in the hydroboration. In the intramolecular control experiment, 1-ethynyl-4-vinylbenzene as a substrate afforded only a single vinylboronate ester **E-4ab** in yield of 95%. These results showed that the microcrystalline Cu_4_NC catalyst possessed high chemoselectivity between alkynes and alkenes for substrates containing C ≡ C bonds under mild conditions; thus, alkynes were the active substrates, and alkenes were inert substrates in the hydroboration catalyzed by microcrystalline Cu_4_NC. When the reaction substrates contained both C ≡ C bonds and C = C bonds, the microcrystalline Cu_4_NC catalyst had high value in the special hydroboration of C ≡ C bond functional groups.

### Mechanistic studies and Density Functional Theory (DFT) calculations

**Fig. 4:**
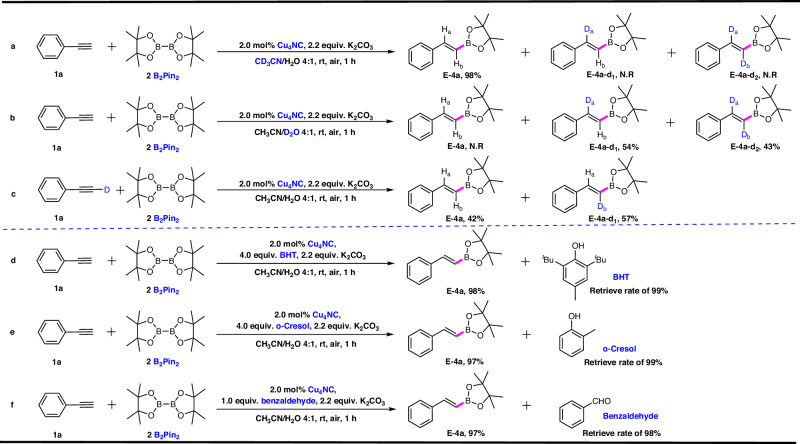
Investigation on the mechanism of the Cu_4_NC-catalyzed hydroboration of alkynes. **a** Deuterium experiments of the Cu_4_NC-catalyzed hydroboration reaction in the mixture solvent (CD_3_CN/H_2_O). **b** Deuterium experiments of the Cu_4_NC-catalyzed hydroboration reaction in the mixture solvent (CH_3_CN/D_2_O). **c** Deuterium experiments of the Cu_4_NC-catalyzed hydroboration of phenylacetylene-d_1_. **d**, **e** Radical scavenging experiments of Cu_4_NC-catalyzed hydroboration. **f** Investigation on the chemoselectivity in Cu_4_NC-catalyzed hydroboration.

To further elucidate the mechanism of hydroboration catalyzed by microcrystalline Cu_4_NC and Cu_8_NC and determine why the microcrystalline Cu_4_NC catalyst has higher catalytic activity than microcrystalline Cu_8_NC, a series of control experiments and density functional theory (DFT) calculations were conducted. First, deuterium experiments of hydroboration catalyzed by microcrystalline Cu_4_NC were explored under the optimal conditions with either CD_3_CN/H_2_O or CH_3_CN/D_2_O as the mixture solvent (Figs. 4a, b, Supplementary Figs. 42–46). When CD_3_CN/H_2_O was used as the catalytic system solvent, the normal vinylboronate ester product **E-4a** was afforded in 98% yield and the deuterated vinylboronate ester was not observed, indicating that the H_a_ atom did not come from CD_3_CN (Fig. 4a, Supplementary Fig. 42). When H_2_O was replaced with D_2_O, two kinds of deuterated products were observed (Fig. 4b, Supplementary Figs. 42–46). The D_a_ and D_b_ atoms clearly originated from D_2_O. The D_a_ atom was introduced into the deuterated products during the elimination reaction in hydroboration. Notably, the manner in which the D_b_ atom was introduced into the deuterated products remains unknown. Two hypotheses are presented here. The first hypothesis concerning the source of the β-site D_b_, is that copper catalysts easily activated the C ≡ C bond of alkynes to form an intermediate π-metal–alkyne complex, which enhanced the acidity of the alkyne proton, facilitating the formation of copper acetylides under basic conditions. In this case, copper acetylides, as active species, participated in hydroboration. During the elimination reaction, deuterated products containing D_b_ atoms were easily obtained. The second hypothesis concerning the source of the β-site D_b_, is that partial phenylacetylene was directly deuterated to phenylacetylene-d_1_ under the action of the strong base K_2_CO_3_ and protic solvent D_2_O.

To confirm which of the above hypotheses regarding the source of the β-site D_b_ is correct, a series of control experiments were carried out. When phenylacetylene-d_1_ was used instead of normal phenylacetylene (Fig. 4c, Supplementary Figs. 42, 47–50), the normal product and deuterated product were obtained, which further proved that the D_b_ atom of the β-site was easily replaced by the H atoms of H_2_O. When CH_3_CN/D_2_O was used as the mixture solvent in the absence of B_2_Pin_2_, and phenylacetylene (**1a**) and K_2_CO_3_ were added to the reaction system catalyzed by microcrystalline Cu_4_NC, providing phenylacetylene-d_1_ in 96% yield (Supplementary Figs. 60–63). Another control experiment was performed: phenylacetylene (**1a**) and K_2_CO_3_ were directly added to the CH_3_CN/D_2_O mixture solvent under the reaction conditions without the addition of the microcrystalline Cu_4_NC catalyst, affording phenylacetylene-d_1_ in 94% yield (Supplementary Figs. 60 and 67–69). These results indicated that the deuteration of phenylacetylene was independent of microcrystalline Cu_4_NC, and the second hypothesis is thus more reasonable, in which the partial phenylacetylene was directly deuterated to phenylacetylene-d_1_ under the strong base K_2_CO_3_. When phenylacetylene and phenylacetylene-d_1_ were applied to the hydroboration reaction catalyzed by microcrystalline Cu_4_NC in the CH_3_CN/D_2_O mixture solvent, two deuterated products were obtained. Based on these results, relevant control experiments were also conducted with Cu_8_NC to gain more insight into the hydroboration of these copper clusters (Supplementary Figs. 51–60 and 64–66). As expected, the process and principle of deuterium experiments with the microcrystalline Cu_8_NC catalyst were consistent with those of microcrystalline Cu_4_NC.

To further explore the possibility of the presence of boron radical species, BHT (4.0 equiv.) or o-cresol (4.0 equiv.) was added to the hydroboration reaction systems catalyzed by microcrystalline Cu_4_NC under standard conditions, and the single vinylboronate ester **E-4a** was still obtained in yield of 98% or 97%, which further verified that the radical species may not participate in the process^43,51^ (Fig. 4d, e, Supplementary Fig. 39). When both the C ≡ C bonds of alkynes and the C = O bonds of aldehydes were simultaneously present within the microcrystalline Cu_4_NC-catalyzed reaction system, the single vinylboronate ester product **E-4a** was still generated in yield of 97%, and benzaldehyde was almost completely recovered, proving that microcrystalline Cu_4_NC is a specific catalyst for the hydroboration of alkynes (Fig. 4f, Supplementary Figs. 40 and 41). To verify the high regio-, stereo- and chemoselectivity in hydroboration catalyzed by microcrystalline Cu_4_NC, we monitored the hydroboration process via in situ ^19^F NMR spectroscopy and found that the characteristic peak of 2-F-PA at -110.10 ppm gradually disappeared and that only the characteristic peak of vinylboronate ester **E-4g** at -117.65 ppm gradually appeared (Supplementary Fig. 70), which demonstrated that microcrystalline Cu_4_NC is an efficient and highly selective catalyst for hydroboration.

In-depth understanding of the mechanism of hydroboration catalyzed by microcrystalline Cu_4_NC or Cu_8_NC was obtained via in situ Fourier transform infrared (FT-IR) spectroscopy and in situ Raman spectroscopy analysis (Supplementary Figs. 83–89). As shown in Supplementary Figs. 87 and 89, the top view of the three-dimensional surface of the in situ FT-IR spectra provided the selected individual spectra of key signals for the hydroboration reaction catalyzed by microcrystalline Cu_4_NC or Cu_8_NC. The IR peak intensities of B-B bonds (1124 cm^-1^ for stretching vibrations) and B-O bonds (1284 cm^-1^ for stretching vibrations) in B_2_Pin_2_ gradually decreased as the reaction proceeded, illustrating the progressive consumption of the B_2_Pin_2_ substrate^52–54^. Moreover, the IR peaks appeared at 1148 cm^-1^, 1352 cm^-1^ and 1624 cm^-1^, which could be assigned to the B-C bond, B-O bond and C = C bond stretching vibrations of the vinylboronate ester target product, respectively^52–54^. The IR peak intensities of the B-C bonds, B-O bonds and C = C bonds in the target product gradually increased with increasing reaction time. A comparison of the in situ FT-IR spectra of the microcrystalline Cu_4_NC and microcrystalline Cu_8_NC catalytic systems further revealed that the catalytic activity of the microcrystalline Cu_4_NC was the stronger than that of microcrystalline Cu_8_NC. As shown in Supplementary Figs. 83 and 84, the characteristic Raman peak at 690 cm^-1^ was attributed to the stretching vibration peak of the Cu-N bond of microcrystalline Cu_4_NC^55,56^. The Raman peak intensities of the Cu-N bonds gradually decreased as the reaction proceeded, indicating that the Cu-N bonds may have dissociated during the hydroboration reaction. These changes in the Raman spectra were attributed to the interaction between the substrates and the microcrystalline Cu_4_NC catalyst, and further supported that the flexible Cu-N bonds of the copper NCs could dynamically dissociate from the copper cluster core to generate key intermediates.

**Fig. 5:**
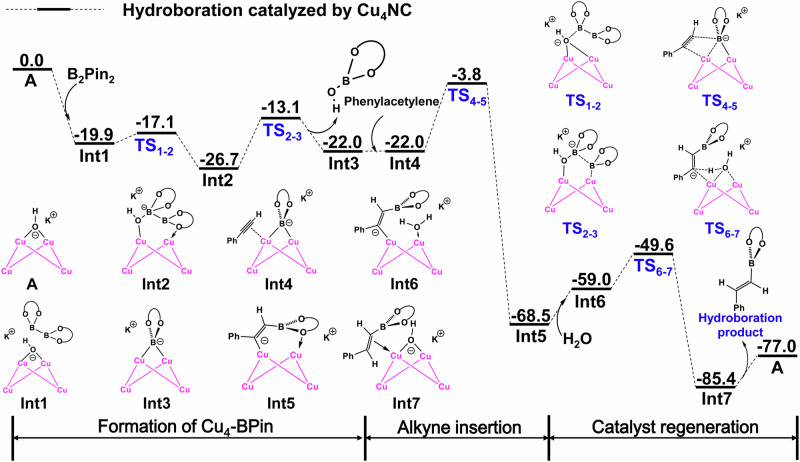
DFT-calculated Gibbs free energy profile. DFT-calculated Gibbs free energy profile (ΔG in kcal/mol) for the hydroboration catalyzed by microcrystalline Cu_4_NC.

**Fig. 6:**
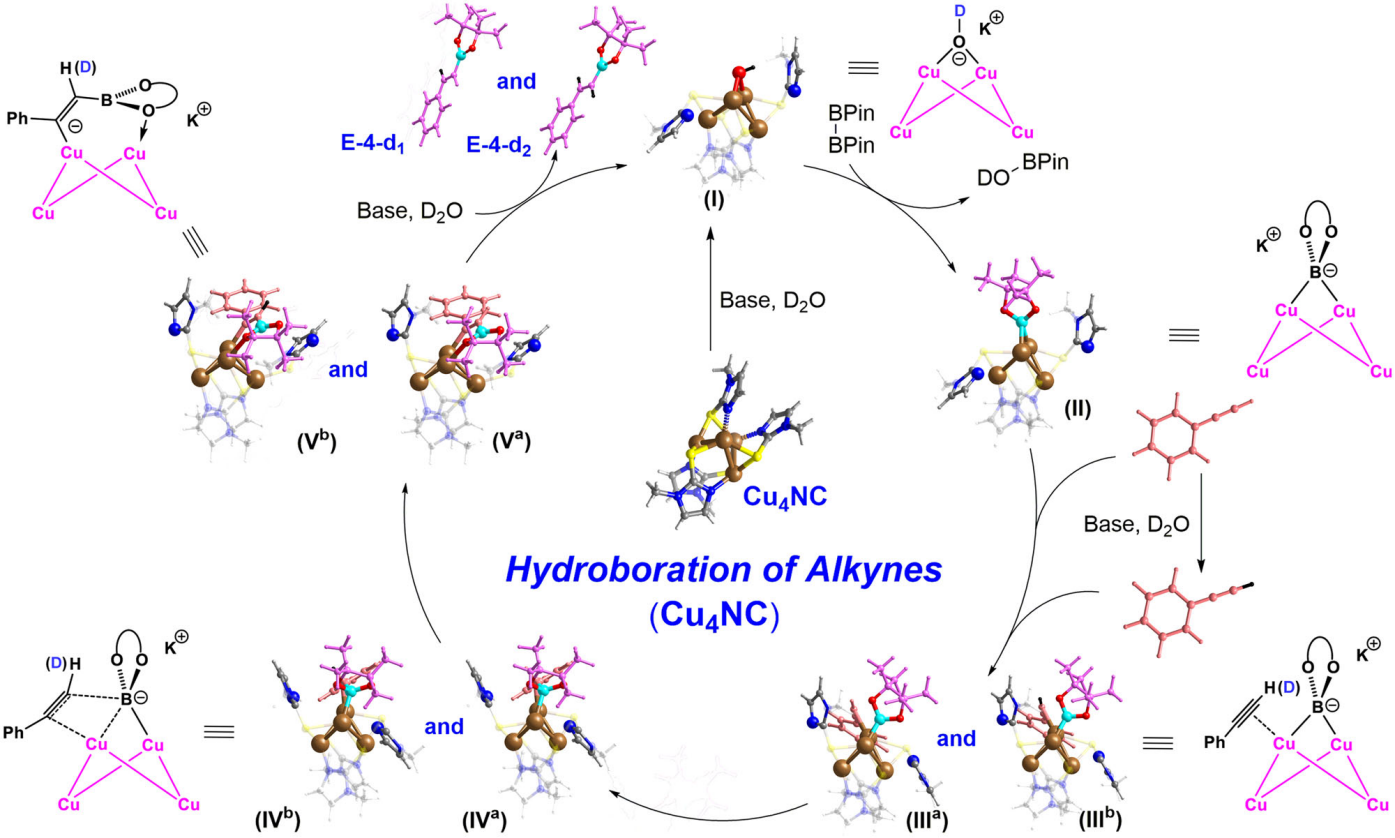
A Proposed reaction mechanism. A Proposed reaction mechanism for the formation of vinylboronate esters catalyzed by microcrystalline Cu_4_NC.

To obtain a deeper understanding of the mechanism of copper NC-catalyzed hydroboration, DFT calculations were performed to explain why the catalytic activity of microcrystalline Cu_4_NC is stronger than that of microcrystalline Cu_8_NC (Figs. 5, 6, Supplementary Figs. 72–79, Supplementary Tables 2 and 3). As shown in Fig. 5 and Supplementary Fig. 78, the catalytic process of the hydroboration reaction catalyzed by copper NCs can be divided into three major steps: borylation to form Cu NCs-BPin, alkyne insertion and catalyst regeneration. In a basic proton environment, the two flexible Cu-N bonds of the copper NCs could dynamically dissociate from the copper cluster core to generate key intermediates A and B (Supplementary Figs. 72 and 73). As shown in Supplementary Fig. 72, the energy barrier leading to intermediate A was only 2.9 kcal/mol and the energy of intermediate A was -19.3 kcal/mol, indicating that this process was both kinetically and thermodynamically favorable. The theoretical calculation results were consistent with the experimental results, which further illustrated that the Cu-N bonds were the flexible in this system. Compared with the Cu-N bonds, the Cu-S bonds were stable during this process, thus maintaining the catalyst integrity and efficient catalytic activity. The *in situ-*generated intermediate Cu NCs-OH and electrophile B_2_Pin_2_ afforded Int3 Cu_4_NC-BPin and Int10 Cu_8_NC-BPin through addition reactions, releasing energies of 22.0 kcal/mol and 19.6 kcal/mol, respectively. The intermediate Cu NCs-BPin with exposed catalytic sites adsorbed alkynes, and then, the insertion of alkynes occurred via transition states TS_4-5_ and TS_11-12_, which had energy barriers of 18.2 kcal/mol and 24.7 kcal/mol, respectively. Int2, Int10, TS_4-5_ and TS_11-12_ represent the intermediates and transition states of the rate-determining step in copper NC-catalyzed hydroboration. The activation energy of Cu_4_NC-catalyzed hydroboration was 22.9 kcal/mol, corresponding to the free energy difference between Int_2_ and TS_4-5_ (ΔG_1_ + ΔG_2_), which was lower than the activation energy of Cu_8_NC-catalyzed hydroboration (ΔG_3_ = 24.7 kcal/mol) (Figs. 5, 6 and Supplementary Figs. 72–79). In addition, to better explain the differences in the catalytic performance of Cu_4_NC and Cu_8_NC in the hydroboration reaction from a theoretical calculation perspective, several popular functionals were tested. In particular, the energy difference between the profiles for Cu_4_NC and Cu_8_NC calculated via the gold standard M06 functional was 12.8 kcal/mol (Supplementary Table 2). These results clearly illustrate that the catalytic activity of Cu_4_NC in the hydroboration reaction is better than that of Cu_8_NC. In an alkaline proton solution, Int5 and Int12 could produce vinylboronate esters and key intermediates A and B via protonation, proving that the copper NCs were regenerated and could enter the next cycle. These theoretical calculation results further support and explain why the microcrystalline Cu_4_NC catalyst displayed good hydroboration performance.

As shown in Fig. 6, plausible mechanisms of the hydroboration reactions catalyzed by copper NCs were proposed based on the above-described in situ characterization, control experiments and theoretical calculations. The organic transformation commences with dynamic dissociation of two flexible Cu-N bonds between the Cu_4_NC metal core and the NHT ligands, releasing the catalytic center to enhance the catalytic activity and promote the generation of the key intermediate Cu_4_NC-OD (I) in a deuterated alkaline protic solution. The intermolecular nucleophilic attack between the electrophile B_2_Pin_2_ and the nucleophile intermediate complex (I) generates the intermediates Cu_4_NC-BPin (II) and DO-BPin. Owing to the presence of a deuterated alkaline protic solvent, some alkynes are deuterated by D_2_O, providing alkyne mixtures of alkyne-d_1_ compounds and normal alkynes during the hydroboration reaction. The exposed catalytic sites of the intermediate Cu_4_NC-BPin (II), which originate from the dynamic dissociation of the Cu-N bonds, have sufficient space to adsorb the alkyne mixtures, resulting in the intermediates (III^a^) and (III^b^). The in situ-generated intermediate (III) can generate intermediates (IV^a^) and (IV^b^) by activating the alkyne mixtures via the exposed copper catalytic sites of Cu_4_NC. Upon dissociation of the Cu-B bond and coordination of the flexible Cu-O bond between the Cu_4_NC metal core and the BPin of intermediate (IV), the in situ-generated intermediate (IV) affords the intermediates (V^a^) and (V^b^) via addition. The key intermediate Cu_4_NC-OD (I) is regenerated by protonation of the intermediate (V) in the deuterated alkaline protic solvent to produce the vinylboronate ester products **E-4d**_**1**_ and **E-4d**_**2**_. As shown in Supplementary Fig. 76, the mechanism of the hydroboration process catalyzed by Cu_8_NC is almost identical to that of the reaction catalyzed by Cu_4_NC, which needs to proceed from the key intermediate Cu_8_NC-OD (VI) to the intermediate (X), resulting in the desired vinylboronate ester products. When the hydroboration process is complete, the copper NC catalysts are recoordinated via the dissociated flexible and reversible Cu-N bonds between the metal core and the NHT ligands of the copper NCs, as revealed by the PXRD patterns, UV−vis spectra, ^1^H NMR spectra, ESI‒MS spectra and Raman spectra obtained to demonstrate the ultrahigh stability of the copper NCs and the rationality of the underlying mechanisms (Supplementary Figs. 31–38 and 85).

**Fig. 7:**
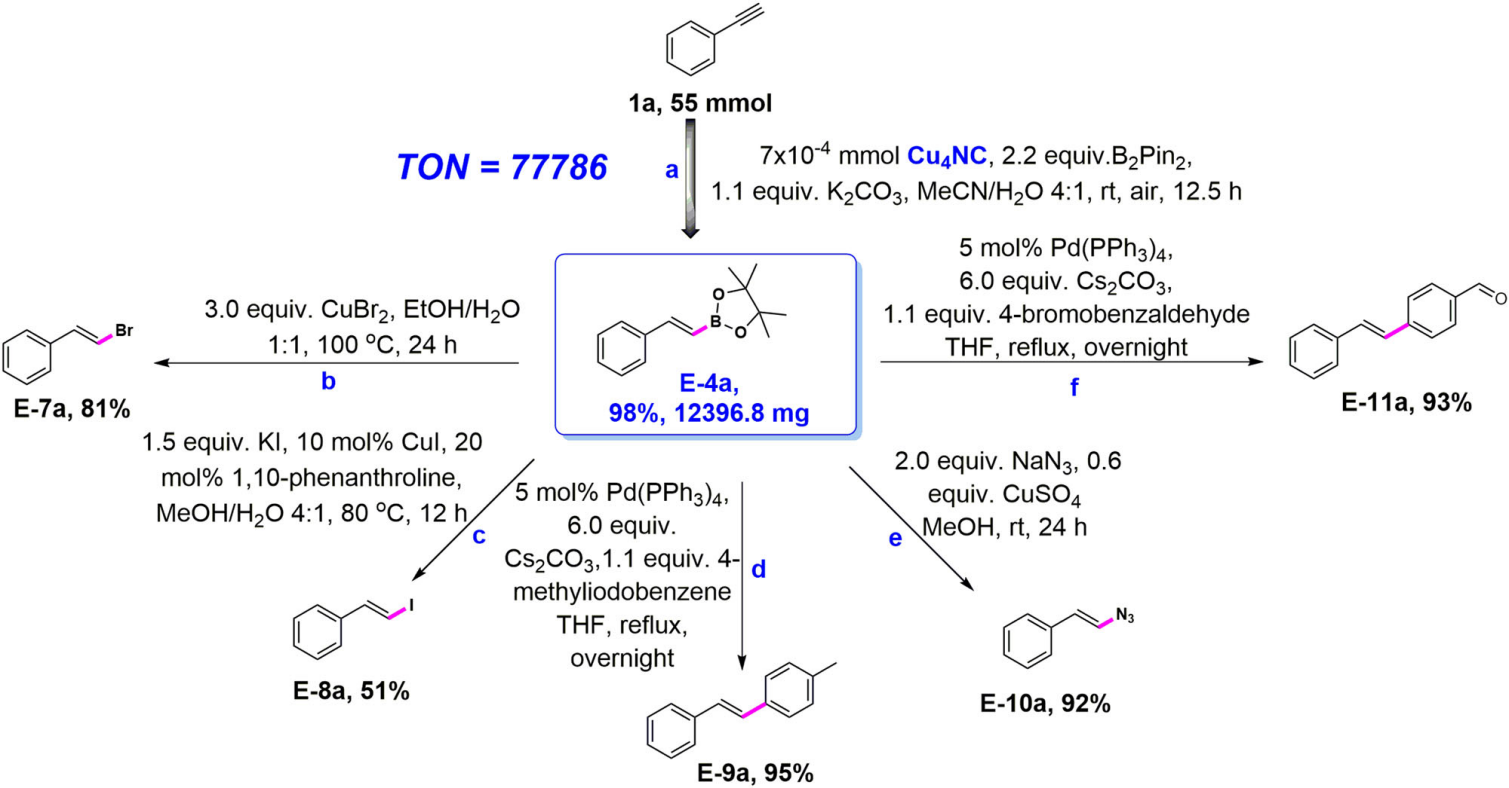
Catalytic performance of Cu_4_NC and derivatization of the B–C bond of vinylboronate ester E-4a. **a** Large-scale synthesis of vinylboronate ester **E-4a**. **b**, **c** Synthesis of disubstituted E-alkenyl halides via halogenation reactions. **d** Construction of C − C bonds through Suzuki cross-coupling of vinylboronate ester and Ar-I. **e** Synthesis of (E)-(2-azidovinyl) benzene from a vinylboronate ester as substrate. **f** Exploration of the functional group derivatizations of trans-stilbenes via a method.

Microcrystalline Cu_4_NC, as a catalyst, exhibits superb stability, good catalytic activity and regio-, stereo- and chemoselectivity, and is thus expected to become a practical catalyst for hydroboration reactions. To further investigate the physical and chemical properties of the microcrystalline Cu_4_NC catalyst, preliminary kinetic studies of hydroboration were performed. An analysis of the linear fit of the ln(C_0_/C) vs. reaction time (t) curve, revealed that the hydroboration transformations had a pseudo-first-order rate dependence on the concentration of phenylacetylenes (Supplementary Figs. 80 and 81). Using a 7 × 10^-4 ^mmol microcrystalline Cu_4_NC catalyst, large-scale vinylboronate ester **E-4a** (12396.8 mg) was synthesized in 98% isolated yield under mild conditions for 12.5 h (Fig. 7a, Supplementary Table 1), which further confirmed the catalytic performance of microcrystalline Cu_4_NC. Significantly, the boryl groups of vinylboronate esters are important building blocks of organic intermediates, which are beneficial for the conversion of organic functional groups through halogenation, arylation, etc. Disubstituted E-alkenyl halides (**E-7a** and **E-8a**) could be efficiently synthesized through halogenation reactions of vinylboronate ester **E-4a** (Fig. 7b, c)^57^. The (E)-(2-azidovinyl) benzene **E-10a** with a defined configuration was also generated in 92% yield via CuSO_4_-mediated transformation of vinylboronate ester **E-4a** (Fig. 7e)^4,41^. Through the Suzuki cross-coupling reaction of vinylboronate ester **E-4a** and Ar-X (X: Br or I) compounds, functional group derivatizations of vinylboronate ester **E-4a** was achieved to provide the trans-stilbenes **E-9a** and **E-11a** (Fig. 7d and f)^4,41^. The flexibility of the boryl groups was fully utilized to promote the development of vinylboronate ester derivatizations. Through an in-depth study of the microcrystalline Cu_4_NC catalyst, its good stability and reusability were recognized. The hydroboration reaction of alkynes was conducted for 5 cycles with the microcrystalline Cu_4_NC catalyst. Nearly 100% yields were achieved for vinylboronate ester in several cyclic experiments (Supplementary Fig. 94).

## Discussion

In conclusion, DRDS copper NCs bearing NHT ligands with dynamic ligand effects were successfully developed in the gram scale and were further investigated as heterogeneous catalysts with high stability, atomically precise structures and dynamic dual catalytic sites. In particular, the designed microcrystalline Cu_4_NC with a well-defined structure displayed high catalytic performance for the hydroboration of alkynes with regio-, stereo- and chemoselectivity (up to 100%), achieving yields (up to 99%) under mild conditions (air atmosphere, protic solvent and room temperature). Importantly, microcrystalline Cu_4_NC achieved a TON of up to 77786, which was higher than those reported for other catalysts. A combination of SCXRD analysis, control experiments, in situ characterization and theoretical calculations consistently revealed that the performance of Cu_4_NC originated from the stable Cu-S bond and the reversible Cu-N bond, which promoted the formation of dynamic dual catalytic centres. This work emphasizes the importance of precisely constructing cluster catalysts with dual catalytic sites to achieve improved catalytic properties and elaborates the structure-activity relationship between the structure of cluster catalysts and their catalytic properties, thus offering a model for designing and constructing heterogeneous cluster catalysts for catalytic research, which can be expected to break ground in organic catalysis of heterogeneous copper clusters and promote the rapid development of cluster chemistry.

## Methods

All the reactions were carried out under ambient atmosphere (air) conditions unless otherwise noted. All commercial reagents and solvents were obtained from the commercial provider and used without further purification. ^1^H NMR, ^13^C**{**^**1**^**H}** NMR, ^19^F NMR and ^11^B{^1^H} NMR spectra were recorded on Bruker 600 MHz spectrometers. Chemical shifts were reported relative to internal tetramethylsilane (δ 0.00 ppm), CDCl_3_ (δ 7.26 ppm) for ^1^H NMR and CDCl_3_ (δ 77.0 ppm) for ^13^C NMR. The nuclear magnetic spectra were analyzed using MestReNova software. Flash column chromatography was performed on 300–400 mesh silica gel.

### Instrumentation

Electrospray ionization mass spectrometry (ESI–MS) of the clusters was recorded using an AB Sciex X500R Q-TOF spectrometer. Powder X-ray diffraction (PXRD) patterns of Cu_4_NC and Cu_8_NC were obtained using a Rigaku B/Max-RB X-ray diffractometer with Cu-K*α* radiation (*λ* = 1.5418 Å) in air at room temperature. UV‒vis absorption spectra were obtained by means of a Hitachi UH4150 UV‒visible spectrophotometer. Energy dispersive spectroscopy (EDS) and elemental mapping measurements were carried out via Zeiss Sigma 500. The in situ IR spectra of all reactions were recorded on ReactIR 701 C230516193 spectrometer. The in situ Roman spectra of hydroboration catalyzed by microcrystalline Cu_4_NC were recorded on a labRAM HR Evolution-HORIBA Raman system equipped with a CCD detector using a 532 nm He-Cd laser as the excitation source. Inductively coupled plasma mass spectrometer (ICP-MS) was performed on IRIS Advantage (Thermo). Elemental analyses (EA) were carried out with a Perkin-Elmer 240 elemental analyser. The X-ray diffraction datum of Cu_4_NC and Cu_8_NC were measured on a Rigaku XtaLAB Pro diffractometer (Supplementary Table 4).

### Materials

Hydroboration of alkynes was carried out under ambient atmosphere unless otherwise noted. Cu_4_NC and Cu_8_NC were synthesized under air atmosphere. The synthesis of deuterated phenylacetylene and 1-ethynyl-4-vinylbenzene substrates were carried out under a dry and oxygen-free N_2_ atmosphere using standard Schlenk techniques. THF (HPLC grade) was dried over sodium/benzophenone and distilled under nitrogen prior to use. Methimazole (99%), B_2_Pin_2_ (99%), (Ph_3_P)_2_PdCl_2_ (98%), 4-bromostyrene (98%), 2-methyl-2-propanethiol (99%), alkynes (97%-98%), ^*n*^BuLi (1.6 mol/l in hexane), methanol-d_4_ (99.8%), various solvents (HPLC grade) and various copper salts (97%-98%) were directly purchased from companies of Energy Chemical, Bidepharm and Heowns without further purification.

### Synthesis of Cu_4_NC

Under air atmosphere, the copper salt [Cu(MeCN)_4_]PF_6_ (1490.9 mg, 4.0 mmol) was dissolved in 100 mL of MeCN, to which 100 mL of methanol solution containing methimazole (456.7 mg, 4.0 mmol) was added under vigorous stirring at room temperature. After the solution turned blue, the reaction was stirred for another 10 min. Then excess triethylamine (2.0 ml) was added to the stirring reaction, resulting in immediate formation of microcrystalline. The suspension was centrifuged, and the microcrystalline was collected. The microcrystalline was dissolved in DCM, and the resulting solution was diffused with MeCN to obtain colorless crystals of **Cu**_**4**_**NC** after 2 days at room temperature (Yield: 75 %, calculated based on methimazole ligand). ^**1**^**H NMR** (600 MHz, CDCl_3_) δ 6.55 (d, *J* = 20.6 Hz, 8H), 3.57 (s, 12H). ^**13**^**C{**^**1**^**H} NMR** (151 MHz, CDCl_3_) δ 151.1, 125.7, 119.9, 34.2. **HRMS(ESI)** m/z: [M+Cs]^+^ calcd for C_16_H_20_CsCu_4_N_8_S_4_^+^ 838.6913. found: 838.7003. Anal. Calc. for Cu_4_NC (C_16_H_20_Cu_4_N_8_S_4_): C, 27.19; H, 2.85; N, 15.85; S, 18.14. Found: C, 27.23; H, 2.76; N, 15.75; S, 18.21.

### Synthesis of Cu_8_NC

Under air atmosphere, ^*t*^BuSCu (305.5 mg, 2.0 mmol) was dissolved in 100 mL of DCM. After sonication, the DCM solution of ^*t*^BuSCu became clear, to which 100 mL of methanol solution containing methimazole (114.2 mg, 1.0 mmol) was added under stirring at room temperature. Then excess triethylamine (500.0 *μ*L) was added to the stirring reaction, and the reaction stirred for another 10 min. The resulting pale-yellow solution was allowed to evaporate slowly at room temperature. After 3 days, yellow block crystals of **Cu**_**8**_**NC** were obtained in a yield of 91.0% (calculated based on ^*t*^BuSCu). ^**1**^**H NMR** (600 MHz, CDCl_3_) δ 6.46 (dd, *J* = 18.9, 1.4 Hz, 8H), 3.58 (s, 12H), 1.36 (s, 36H). ^**13**^**C{**^**1**^**H} NMR** (151 MHz, CDCl_3_) δ 150.6, 124.8, 119.5, 100.0, 48.0, 35.5, 34.0. **HRMS(ESI)** m/z: [M+Cs]^+^ calcd for C_32_H_56_CsCu_8_N_8_S_8_^+^ 1450.5766. found: 1450.5869. Anal. Calc. for Cu_8_NC (C_32_H_56_Cu_8_N_8_S_8_): C, 29.17; H, 4.28; N, 8.50; S, 19.46. Found: C, 29.22; H, 4.24; N, 8.42; S, 19.52.

### General procedure for Cu_4_NC-catalyzed the hydroboration reaction

Under air atmosphere, alkynes **1** (0.2 mmol, 1.0 eq), B_2_Pin_2_ **2** (0.44 mmol, 2.2 equiv.), microcrystalline Cu_4_NC catalysts (2.0 mol%), K_2_CO_3_ (0.44 mmol, 2.2 equiv.) and mixture solvent (2.0 mL, MeCN-H_2_O, v/v 4/1) were added into the tube. The reaction mixture was stirred at room temperature for 1 h or 2 h. The reactions were monitored by TLC. When alkynes were consumed, the reactions were quenched and concentrated. The crude products were then purified by column chromatography to give the target products.

### General procedure for Cu_4_NC-catalyzed the hydroboration reaction in 55 mmol scale

Under air atmosphere, phenylacetylene **1** (5615.5 mg, 55.0 mmol, 1.0 eq), B_2_Pin_2_ **2** (30721.9 mg, 121.0 mmol, 2.2 equiv.), microcrystalline Cu_4_NC catalysts (0.5 mg, 7 × 10^-4 ^ mmol, 1.3 × 10^-3 ^ mol%), K_2_CO_3_ (8361.7 mg, 60.5 mmol, 1.1 equiv.) and the mixture solvent (55.0 mL, MeCN-H_2_O, v/v 4/1) were added into a 100 mL flask. The reaction mixture was stirred at room temperature for 12.5 h. The reactions were monitored by TLC. When alkynes were consumed, the reactions were quenched and concentrated. The crude products were then purified by column chromatography to give the (E)-4,4,5,5-tetramethyl-2-styryl-1,3,2-dioxaborolane **(E-4a)** products as colorless oil with an overall isolated yield: 98% (12396.8 mg).

### Recycling procedure for Cu_4_NC-catalyzed the hydroboration reaction

Under air atmosphere, phenylacetylene **1** (20.4 mg, 0.2 mmol, 1.0 eq), B_2_Pin_2_ **2** (111.8 mg, 0.44 mmol, 2.2 equiv.), microcrystalline Cu_4_NC catalysts (2.8 mg, 2.0 mol%), K_2_CO_3_ (60.7 mg, 0.44 mmol, 2.2 equiv.) and the mixture solvent (2.0 mL, MeCN-H_2_O, v/v 4/1) were added into a 10 mL flask. The reaction mixture was stirred at room temperature for 1 h. Conversions and yields were determined by ^1^H NMR using 1,3,5-trimethoxybenzene as an internal standard. After the reaction is completed, the reaction mixture was centrifuged at 8000 rpm for 5 min. The microcrystalline Cu_4_NC catalyst was filtered, washed with hexane and then dried in vacuum to recycle the microcrystalline Cu_4_NC. The recycled microcrystalline Cu_4_NC catalyst was used again in the next recycle. In this way, the hydroboration reaction of alkynes was conducted for 5 cycles with the microcrystalline Cu_4_NC catalyst (Supplementary Fig. 94).

## Supplementary information

Supplementary Information

Peer Review File

## Source data

Source Data

## Data Availability

All other data are available from the corresponding author upon request. All data needed to evaluate the conclusions in the paper are present in the paper and/or the Supplementary Materials (including Supplementary Figs. 1–176, details of the chemicals, instrumentation, synthesis, characterization, DFT and X-ray crystal details for Cu_4_NC (CIF) and Cu_8_NC (CIF)). The source data for Figs. 5 and 6 and Supplementary Figs. 74–79 are provided with this paper. The X-ray crystallographic coordinates for the structures reported in this article have been deposited at the Cambridge Crystallographic Data Centre (CCDC), under deposition number CCDC 2330281 (Cu_4_NC) 2330282 (Cu_8_NC). These data can be obtained free of charge via www.ccdc.cam.ac.uk/data_request/cif. Source data are provided with this paper.
