# Supplementary material for: Atomically precise copper clusters with dual sites for highly chemoselective and efficient hydroboration
Source: Nat Commun. 2024 Nov 5;15:9551. doi: 10.1038/s41467-024-53950-7 (PMC11538399; doi:10.1038/s41467-024-53950-7)
Supplement: Supplementary file 1 — Supplementary Information [file 41467_2024_53950_MOESM1_ESM.pdf]

Supplementary information

Atomically Precise Copper Clusters with Dual Sites for Highly Chemoselective and Efficient Hydroboration

---

In the format provided by the authors and unedited

Supplementary Information (SI) for

Atomically Precise Copper Clusters with Dual Sites for Highly Chemoselective and Efficient Hydroboration

Teng Jia[1], Jie Ai[1], Xiaoguang Li[2], Miao-Miao Zhang[1,3], Yue Hua[1], Yi-Xin Li[1], Cai-Fang Sun[1], Feng Liu[4], Ren-Wu Huang[1]*, Zheng Wang[2]* & Shuang-Quan Zang[1]*

[1]Henan Key Laboratory of Crystalline Molecular Functional Materials and College of Chemistry, Zhengzhou University, Zhengzhou, 450001, China.
[2]Institute for Advanced Study, Shenzhen University, Shenzhen, 518060, China.
[3]School of Chemistry and Chemical Engineering, Henan University of Technology, Zhengzhou, 450001, China.
[4]Yunnan Precious Metals Lab Co., LTD, Kunming, 650106, China.

Correspondence to: zangsqzg@zzu.edu.cn, renwuhuang@zzu.edu.cn, chzwang@szu.edu.cn

Contents

General Methods and Materials 3
Synthetic Procedures 4
Characterizations of Cu_4NC and Cu_8NC 7
General Procedure for Cu_4NC-Catalyzed the Hydroboration Reaction 24
General Procedure for Cu_4NC-Catalyzed Hydroboration Reaction in 55 mmol Scale. 25
Investigation on the Lowest Load of Cu_4NC Catalyst for the Hydroboration Transformation 26
Catalytic Mechanism Studies by a Series of Control Experiments 27
Catalytic Mechanism Studies by Density Functional Theory (DFT) Calculations 47
The Preliminary Kinetic Studies of the Hydroboration Reaction 54
The *in situ* Raman Experiment 55
The *in situ* Fourier Transform Infrared (FT-IR) Experiment 57
Deprotection of 1,3,5-Trimethoxybenzene Catalyzed by Microcrystalline Cu Clusters 59
Recyclability of the Cu_4NC Catalyzed Hydroboration Reaction 61
Monitor the State of the Microcrystalline Cu_4NC during the Catalytic Process Using PXRD 63
Monitor the Catalytic Ability of the Supernatant Using *in situ* ^{1}H NMR 65
Derivatization of the Products from Hydroboration of Alkynes 66
Tables of Crystal Data and Structure Refinements 74
Compounds Characterization 75
Comparison of TON and TOF 89
^{1}H, ^{13}C and ^{19}F NMR Spectra of Compounds 90
Coordinates of the Structures 123
Supplementary References 188

General Methods and Materials

All the reactions were carried out under ambient atmosphere (air) conditions unless otherwise noted. All commercial reagents and solvents were obtained from the commercial provider and used without further purification. ^{1}H NMR, ^{13}C**{^{1}H}** NMR, ^{19}F NMR and ^{11}B{^{1}H} NMR spectra were recorded on Bruker 600 MHz spectrometers. Chemical shifts were reported relative to internal tetramethylsilane (δ 0.00 ppm), $CDCl_3$ (δ 7.26 ppm) for ^{1}H NMR and $CDCl_3$ (δ 77.0 ppm) for ^{13}C NMR. The nuclear magnetic spectra were analyzed using MestReNova software. Flash column chromatography was performed on 300–400 mesh silica gel. Electrospray ionization mass spectrometry (ESI–MS) of the clusters was recorded using an AB Sciex X500R Q-TOF spectrometer. Powder X-ray diffraction (PXRD) patterns of Cu_4NC and Cu_8NC were obtained using a Rigaku B/Max-RB X-ray diffractometer with Cu-K*α* radiation (*λ* = 1.5418 Å) in air at room temperature. UV–vis absorption spectra were obtained by means of a Hitachi UH4150 UV–visible spectrophotometer. Energy dispersive spectroscopy (EDS) and elemental mapping measurements were carried out via Zeiss Sigma 500. The *in situ* IR spectra of all reactions were recorded on ReactIR 701 C230516193 spectrometer. Inductively coupled plasma mass spectrometer (ICP-MS) was performed on IRIS Advantage (Thermo). Elemental analyses (EA) were carried out with a Perkin-Elmer 240 elemental analyser.

The *in situ* Raman spectra of hydroboration catalyzed by microcrystalline Cu_4NC were recorded on a labRAM HR Evolution-HORIBA Raman system equipped with a CCD detector using a 532 nm He-Cd laser as the excitation source. The microscope attachment of the spectrometer is a × 50_VIS_LWD objective. The laser spot on the sample is approximately 500 nm in diameter, and the spectral resolution is approximately ≤ 0.35 cm^{-1}. Low laser power (3.2%) and exposure time (1 s) were used during the experiment to minimize the impact of laser irradiation during spectral recording. The temperature was held constant at 25 °C. The spectra of the microcrystalline **Cu_4NC** were collected at different time (Supplementary Figure 82).

Single crystal analysis: Single-crystal X-ray diffraction measurements of Cu_4NC and Cu_8NC were performed on a Rigaku XtaLAB Pro diffractometer with Cu-Kα radiation (*λ* = 1.5418 Å) at 200 K. Data collection and reduction were performed by the program CrysAlisPro[(1)]. The crystal structures were solved with direct methods (*SHELXS*)[(2)] and refined by full-matrix least squares on F^2 using *OLEX2*[(3)], which utilizes the *SHELXL*-2015 module[(4)]. All non-hydrogen atoms were refined anisotropically. Hydrogen atoms were placed in calculated positions refined using idealized geometries and assigned fixed isotropic displacement parameters. Structure refinement was handled with different strategies according to the electron density distribution. The imposed restraints in least-squares refinement of each structure were noted in the corresponding CIF files. Detailed information about the X-ray crystal data, intensity collection procedure and refinement results for all cluster compounds is summarized in Supplementary Table 4.

Materials

Hydroboration of alkynes was carried out under ambient atmosphere unless otherwise noted. Cu_4NC and Cu_8NC were synthesized under air atmosphere. The synthesis of deuterated phenylacetylene and 1-ethynyl-4-vinylbenzene substrates were carried out under a dry and oxygen-free N_2 atmosphere using standard Schlenk techniques. THF (HPLC grade) was dried over sodium/benzophenone and distilled under nitrogen prior to use. Methimazole (99%), B_2Pin_2 (99%), $(Ph_3P)_2PdCl_2$ (98%), 4-bromostyrene (98%), 2-methyl-2-propanethiol (99%), alkynes (97%-98%), ^{n}BuLi (1.6mol/l in hexane), methanol-d_4 (99.8%), various solvents (HPLC grade) and various copper salts (97%-98%) were directly purchased from companies of Energy Chemical, Bidepharm and Heowns without further purification.

The ^{t}BuSCu was prepared according to the reported literature(5).

Synthetic Procedures

General Procedure for the Synthesis of Cu_4NC and Cu_8NC

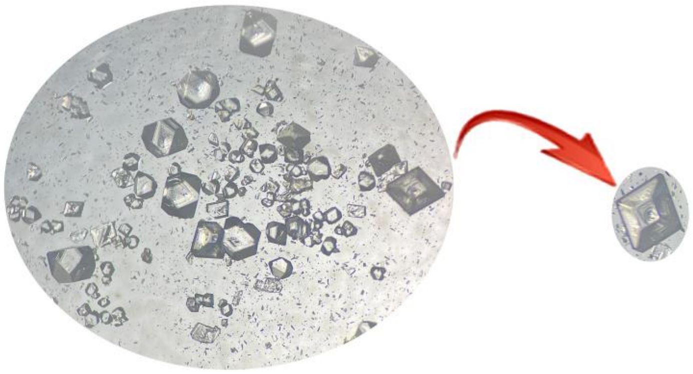

Supplementary Figure 1. Photograph of the Cu_4NC single crystals. The Cu_4NC single crystals were prepared in DCM /MeCN solution.

Synthesis of Cu_4NC: Under air atmosphere, the copper salt $[Cu(MeCN)_4]PF_6$ (1490.9 mg, 4.0 mmol) was dissolved in 100 mL of MeCN, to which 100 mL of methanol solution containing methimazole (456.7 mg, 4.0 mmol) was added under vigorous stirring at room temperature. After the solution turned blue, the reaction was stirred for another 10 minutes. Then excess triethylamine (2.0 ml) was added to the stirring reaction, resulting in immediate formation of microcrystalline. The suspension was centrifuged, and the microcrystalline was collected. The microcrystalline was dissolved in DCM, and the resulting solution was diffused with MeCN to obtain colorless crystals of **Cu_4NC** after 2 days at room temperature (Yield: 75 %, calculated based on methimazole ligand). **^{1}H NMR** (600 MHz, $CDCl_3$) δ 6.55 (d, J = 20.6 Hz, 8H), 3.57 (s, 12H). **^{13}C{^{1}H} NMR** (151 MHz, $CDCl_3$) δ 151.1, 125.7, 119.9, 34.2. **HRMS(ESI)** m/z: $[M+Cs]^+$ calcd for $C_{16}H_{20}CsCu_4N_8S_4^+$ 838.6913. found: 838.7003. Anal. Calc. for Cu_4NC ($C_{16}H_{20}Cu_4N_8S_4$): C, 27.19; H, 2.85; N, 15.85; S, 18.14. Found: C, 27.23; H, 2.76; N, 15.75; S, 18.21.

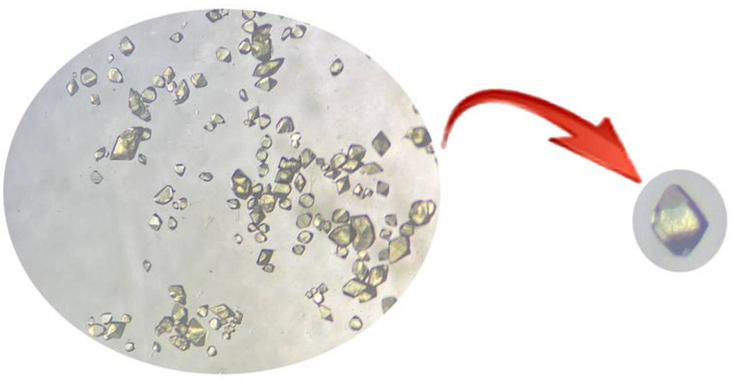

Supplementary Figure 2. Photograph of the Cu_8NC single crystals. The Cu_8NC single crystals were prepared in DCM /MeOH solution.

Synthesis of Cu_8NC: Under air atmosphere, ^{t}BuSCu (305.5 mg, 2.0 mmol) was dissolved in 100 mL of DCM. After sonication, the DCM solution of ^{t}BuSCu became clear, to which 100 mL of methanol solution containing methimazole (114.2 mg, 1.0 mmol) was added under stirring at room temperature. Then excess triethylamine (500.0 μL) was added to the stirring reaction, and the reaction stirred for another 10 minutes. The resulting pale-yellow solution was allowed to evaporate slowly at room temperature. After 3 days, yellow block crystals of **Cu_8NC** were obtained in a yield of 91.0% (calculated based on ^{t}BuSCu). **^{1}H NMR** (600 MHz, $CDCl_3$) δ 6.46 (dd, J = 18.9, 1.4 Hz, 8H), 3.58 (s, 12H), 1.36 (s, 36H). **^{13}C{^{1}H} NMR** (151 MHz, $CDCl_3$) δ 150.6, 124.8, 119.5, 100.0, 48.0, 35.5, 34.0. **HRMS(ESI)** m/z: $[M+Cs]^+$ calcd for $C_{32}H_{56}CsCu_8N_8S_8^+$ 1450.5766. found: 1450.5869. Anal. Calc. for Cu_8NC ($C_{32}H_{56}Cu_8N_8S_8$): C, 29.17; H, 4.28; N, 8.50; S, 19.46. Found: C, 29.22; H, 4.24; N, 8.42; S, 19.52.

General Procedure for the Synthesis of Deuterated Phenylacetylene

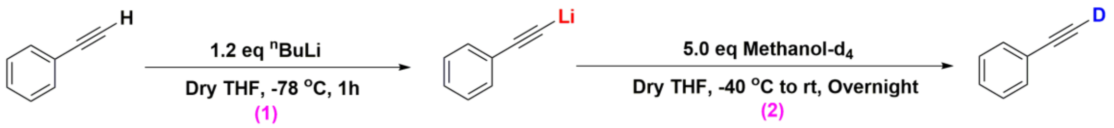

Supplementary Figure 3. The Synthetic route for deuterated substrate. The synthetic route for deuterated phenylacetylene.

Under nitrogen atmosphere, ^{n}BuLi (1.60 M in hexane, 3.75 mL, 6.0 mmol) was added dropwise to a solution of phenylacetylene (510.5 mg, 5.00 mmol) in 60 mL dry THF at –78 ºC. The mixture was stirred for 1 hour to obtain lithium acetylide solution then allowed to warm to –40 ºC and stirred for another 3 h before the addition of methanol-d_4 (900.0 mg, 25.0 mmol) in one portion. The resulting mixture was allowed to warm to room temperature gradually and stirred overnight. Removal of the volatiles under reduced pressure gave a pale-yellow residue that was then purified by column chromatography to give a pale-yellow oil with an overall isolated yield: 92%.

General Procedure for the Synthesis of 1-Ethynyl-4-vinylbenzene[(6)]

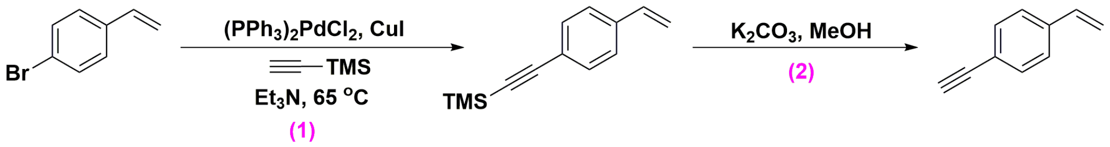

Supplementary Figure 4. The Synthetic route for substrate. The synthetic route for 1-ethynyl-4-vinylbenzene.

Under N_2 atmosphere, a mixture of 4-bromostyrene (1830.5 mg, 10.0 mmol), $(Ph_3P)_2PdCl_2$ (140.4 mg, 0.2mmol) and CuI (19.1 mg, 0.1mmol) in a flask was degassed, then Et_3N (0.2 M for substrate, 50ml) was added. To this solution ethynyl trimethylsilane (4911.0 mg, 50mmol) was added by syringe. The mixture was heated to 65 ºC for 12 h, and TLC showed the starting 4-bromostyrene was completely consumed. After cooling to room temperature, the reaction mixture was filtrated through celite. The filtrate was concentrated and the crude product was purified by chromatography (PE-EA, v/v 1/0) to give a yellow oil with an overall isolated yield: 80%. **^{1}H NMR** (600 MHz, $CDCl_3$) δ 7.43 (d, *J* = 8.3 Hz, 2H), 7.34 (d, *J* = 8.2 Hz, 2H), 6.69 (dd, *J* = 17.6, 10.9 Hz, 1H), 5.76 (d, *J* = 17.6 Hz, 1H), 5.29 (d, *J* = 10.9 Hz, 1H), 0.26 (s, 9H). **^{13}C{^{1}H} NMR** (151 MHz, $CDCl_3$) δ 137.6, 136.2, 132.1, 126.0, 122.4, 114.8, 105.1, 94.8, 0.0.

Under air atmosphere, the product was dissolved in MeOH (0.2 M for substrate), and then K_2CO_3 (138.2 mg, 1.0 mmol) was added. Desilylation was conducted at room temperature for 2 hours and concentrated. The product was purified by chromatography to give a yellow solid with an overall isolated yield: 95%. **^{1}H NMR** (600 MHz, $CDCl_3$) δ 7.45 (d, *J* = 8.1 Hz, 2H), 7.36 (d, *J* = 8.1 Hz, 2H), 6.70 (dd, *J* = 17.6, 10.9 Hz, 1H), 5.77 (d, *J* = 17.6 Hz, 1H), 5.30 (d, *J* = 10.9 Hz, 1H), 3.11 (s, 1H). **^{13}C{^{1}H} NMR** (151 MHz, $CDCl_3$) δ 138.0, 136.1, 132.3, 126.1, 121.3, 115.1, 83.6, 77.7.

Characterizations of Cu_4NC and Cu_8NC

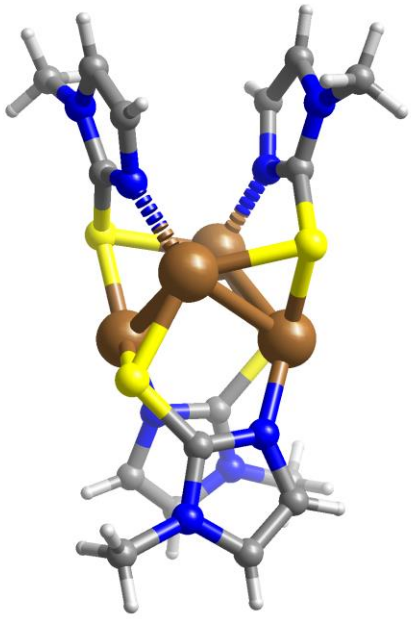

Supplementary Figure 5. Characterization of Cu_4NC. Crystal structure of Cu_4NC (CCDC: 2330281).

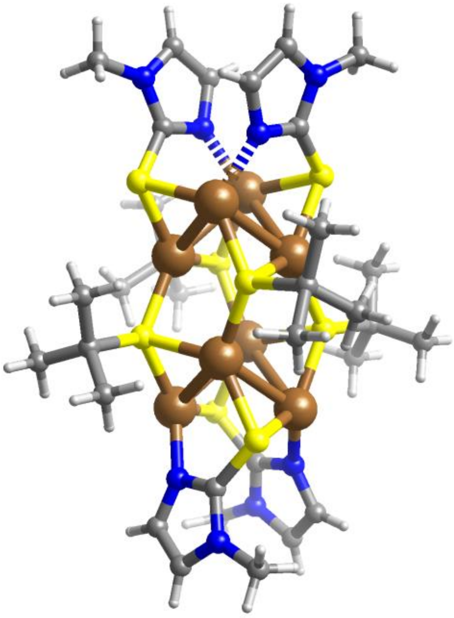

Supplementary Figure 6. Characterization of Cu_8NC. Crystal structure of Cu_8NC (CCDC: 2330282).

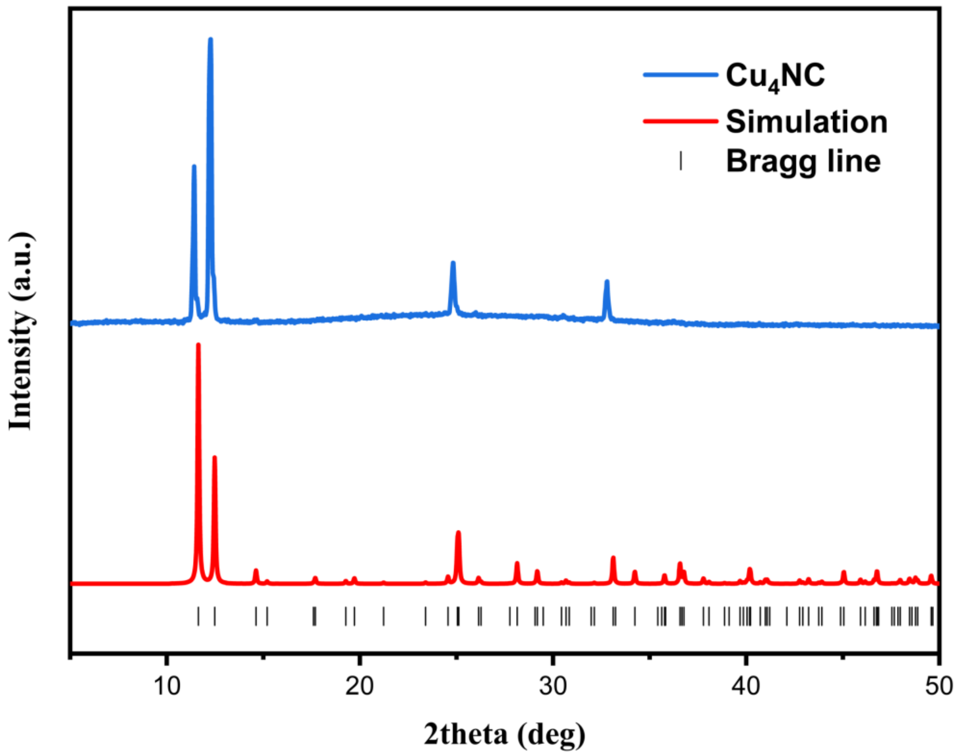

Supplementary Figure 7. Characterization of Cu_4NC. PXRD pattern of microcrystalline Cu_4NC.

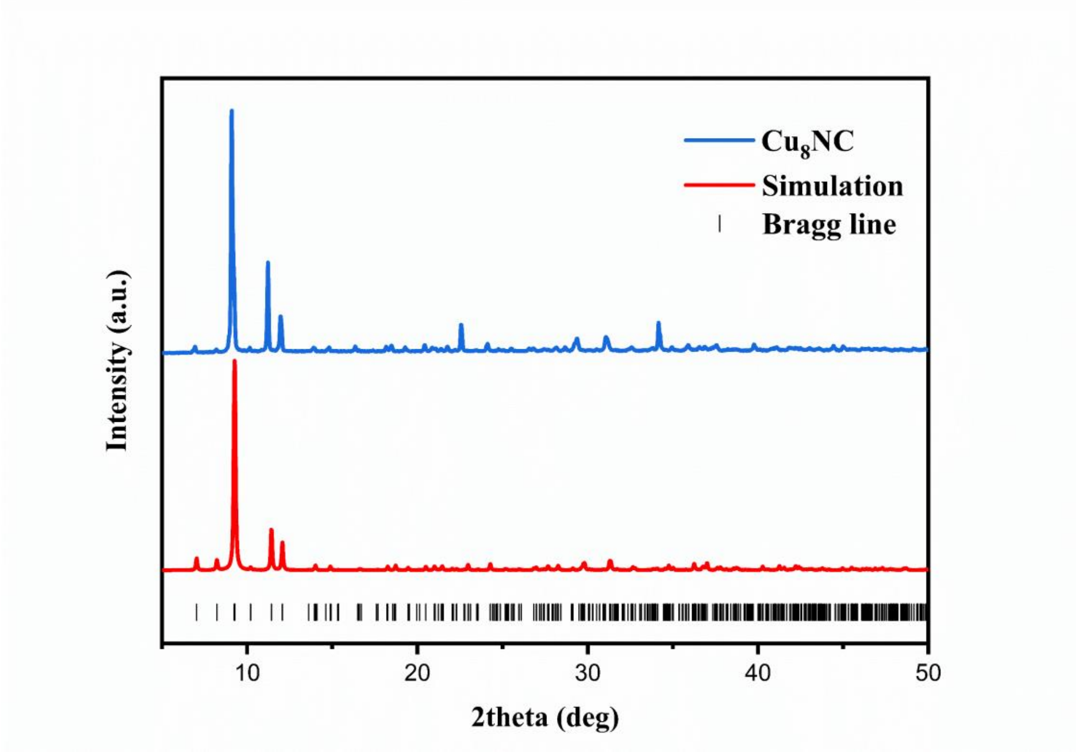

Supplementary Figure 8. Characterization of Cu_8NC. PXRD pattern of microcrystalline Cu_8NC.

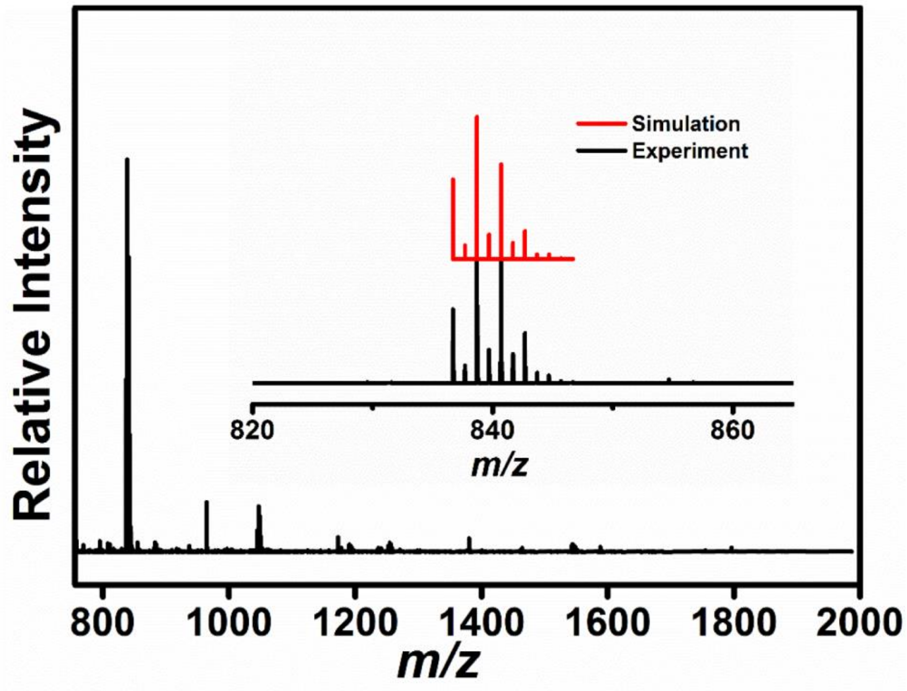

Supplementary Figure 9. ESI–MS spectrum of Cu_4NC. ESI–MS spectrum of Cu_4NC in DMSO solution.

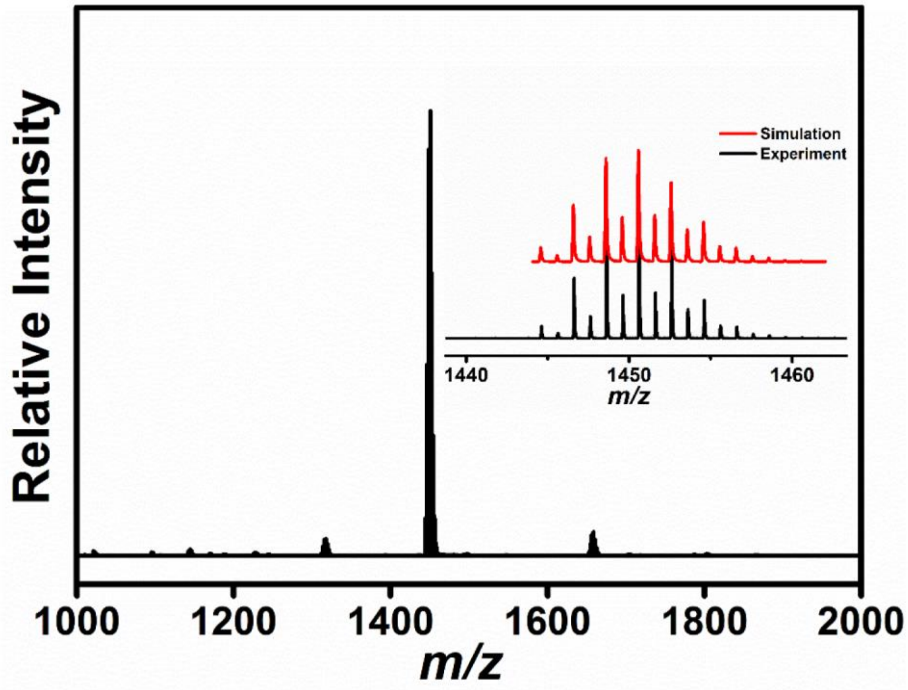

Supplementary Figure 10. ESI–MS spectrum of Cu_8NC. ESI–MS spectrum of Cu_8NC in DMSO solution.

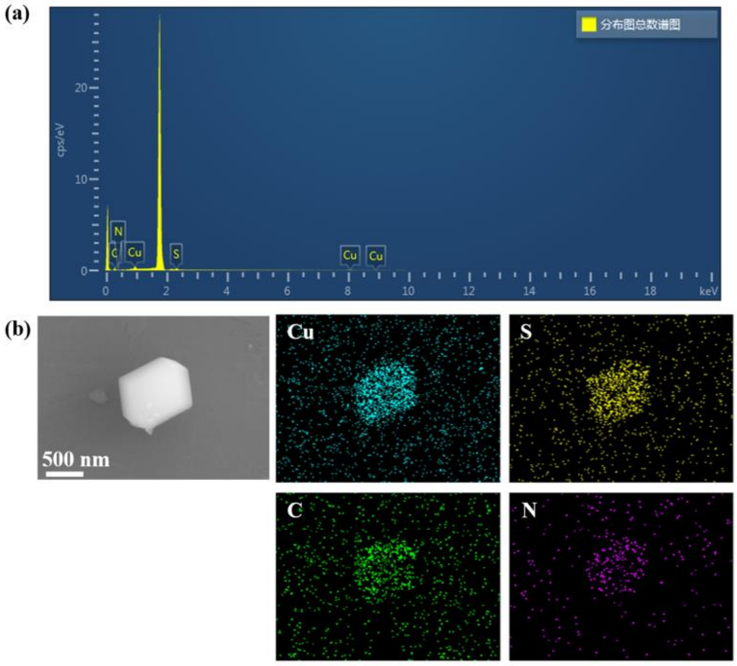

Supplementary Figure 11. Morphology and energy dispersive spectroscopy (EDS) mapping results of microcrystalline Cu_4NC. a. Energy dispersive spectroscopy (EDS) mapping of Cu_4NC. **b.** Mapping test of microcrystalline Cu_4NC.

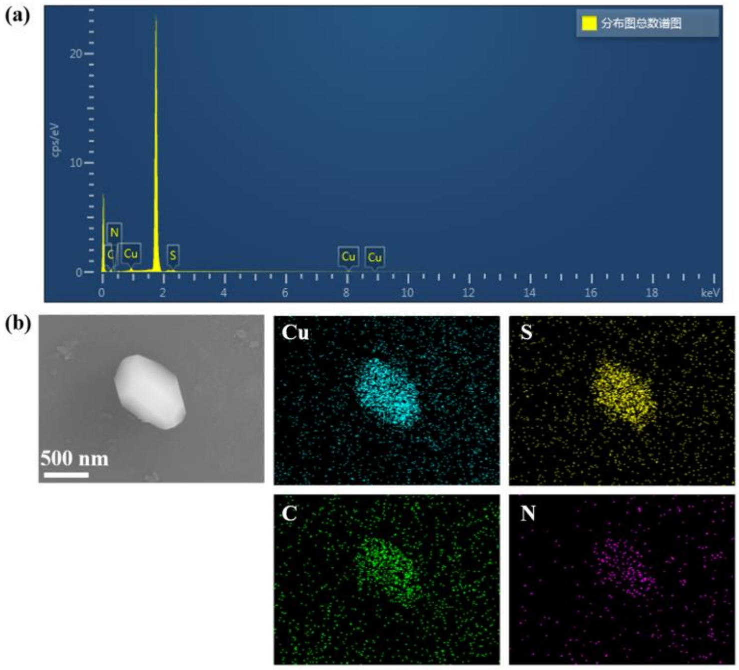

Supplementary Figure 12. Morphology and energy dispersive spectroscopy (EDS) mapping results of microcrystalline Cu_8NC. a. Energy dispersive spectroscopy (EDS) mapping of Cu_8NC. **b.** Mapping test of microcrystalline Cu_8NC.

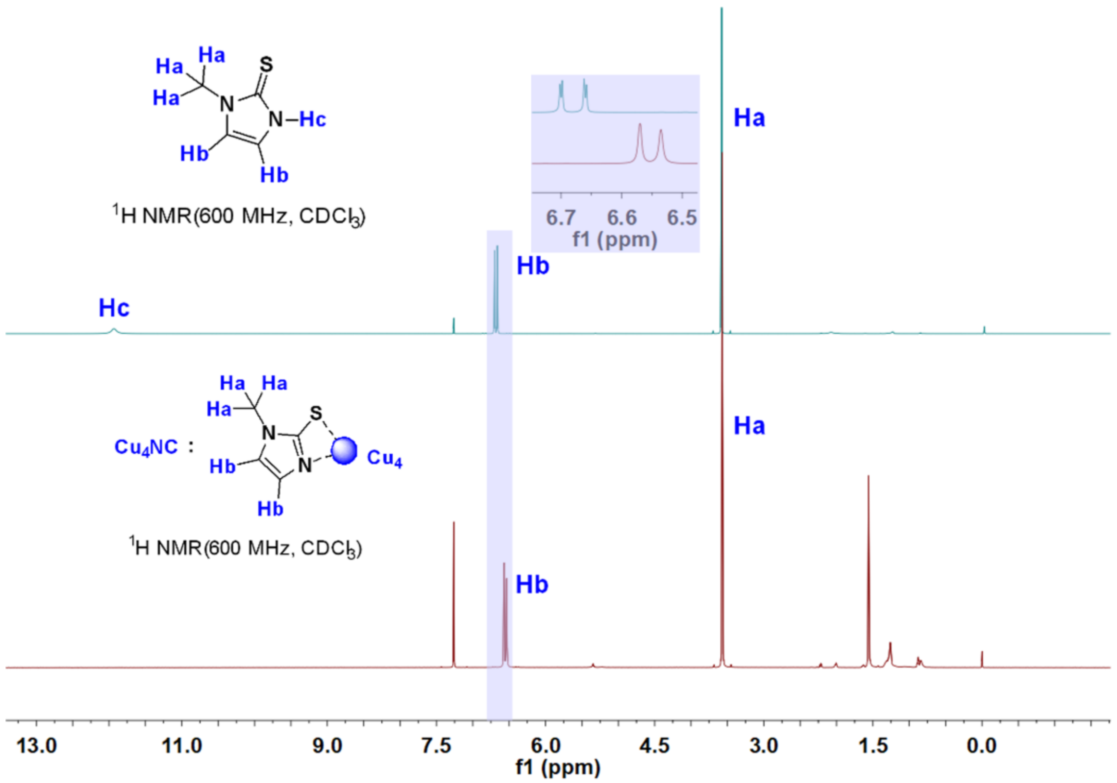

Supplementary Figure 13. 1H NMR spectra of Cu_4NC and ligand. 1H NMR spectra of Cu_4NC and methimazole ligand in $CDCl_3$.

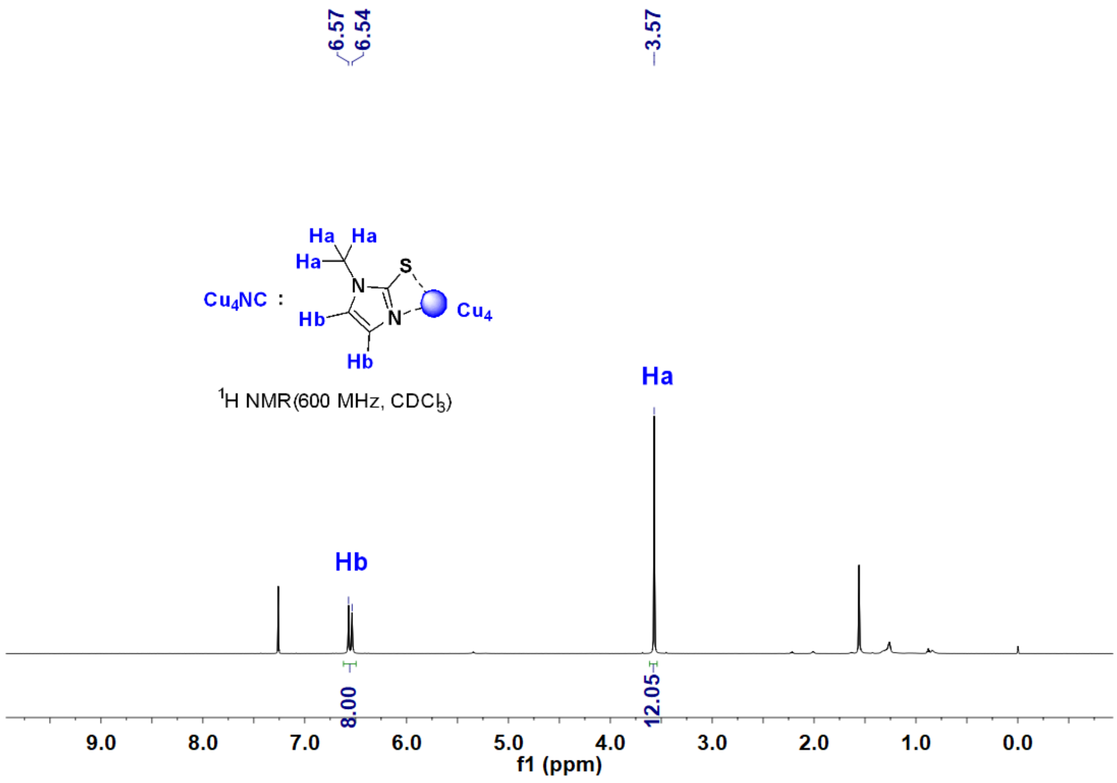

Supplementary Figure 14. 1H NMR spectra of Cu_4NC. 1H NMR spectrum of Cu_4NC in $CDCl_3$.

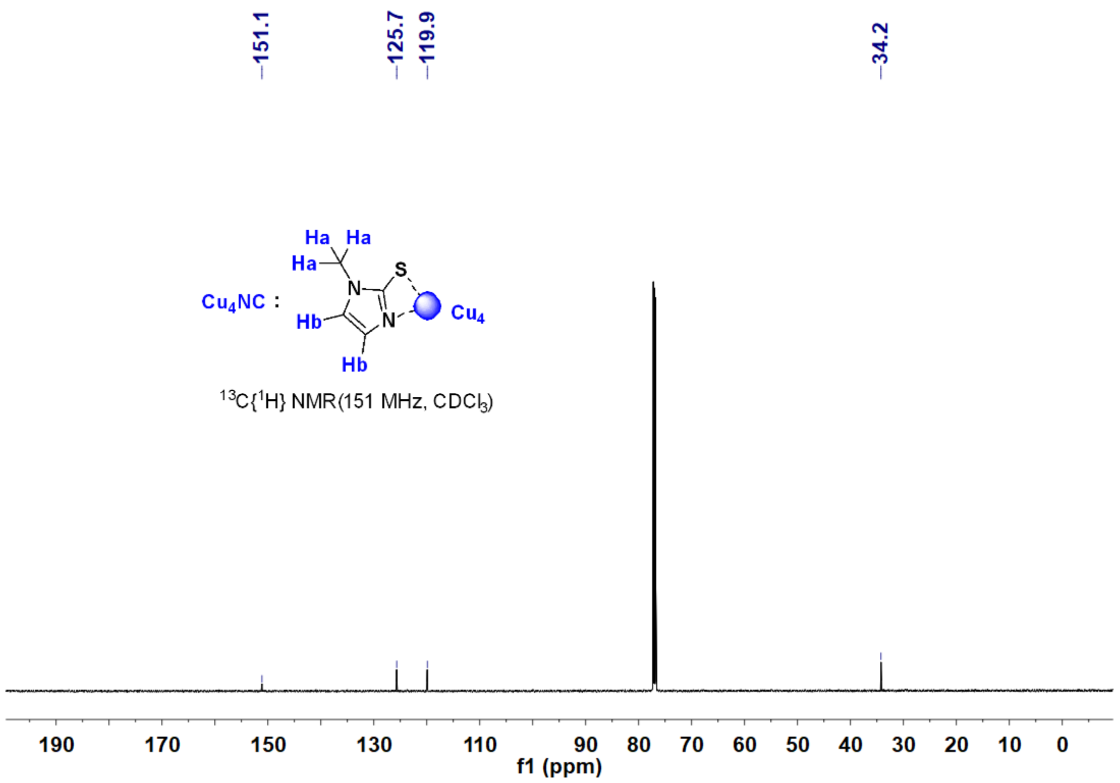

Supplementary Figure 15. $^{13}C\{^{1}H\}$ NMR spectrum of Cu_4NC. $^{13}C\{^{1}H\}$ NMR spectrum of Cu_4NC in $CDCl_3$.

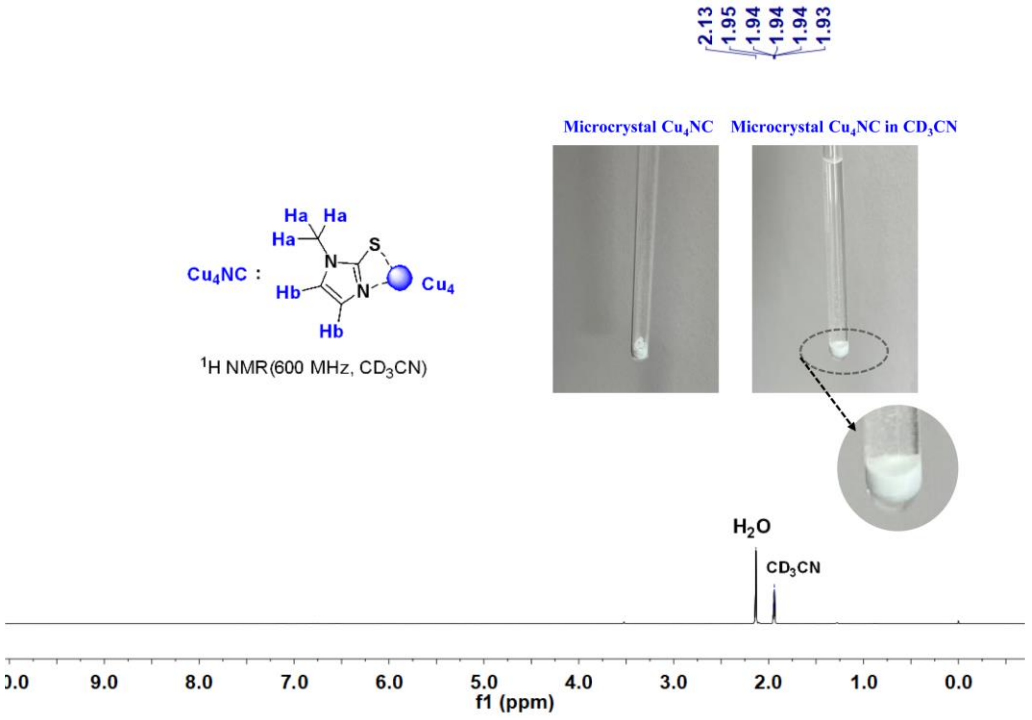

Supplementary Figure 16. ^{1}H NMR spectrum of microcrystalline Cu_4NC in CD_3CN. No characteristic peaks of Cu_4NC are found in ^{1}H NMR spectrum, proving that microcrystalline Cu_4NC is completely insoluble in MeCN solution.

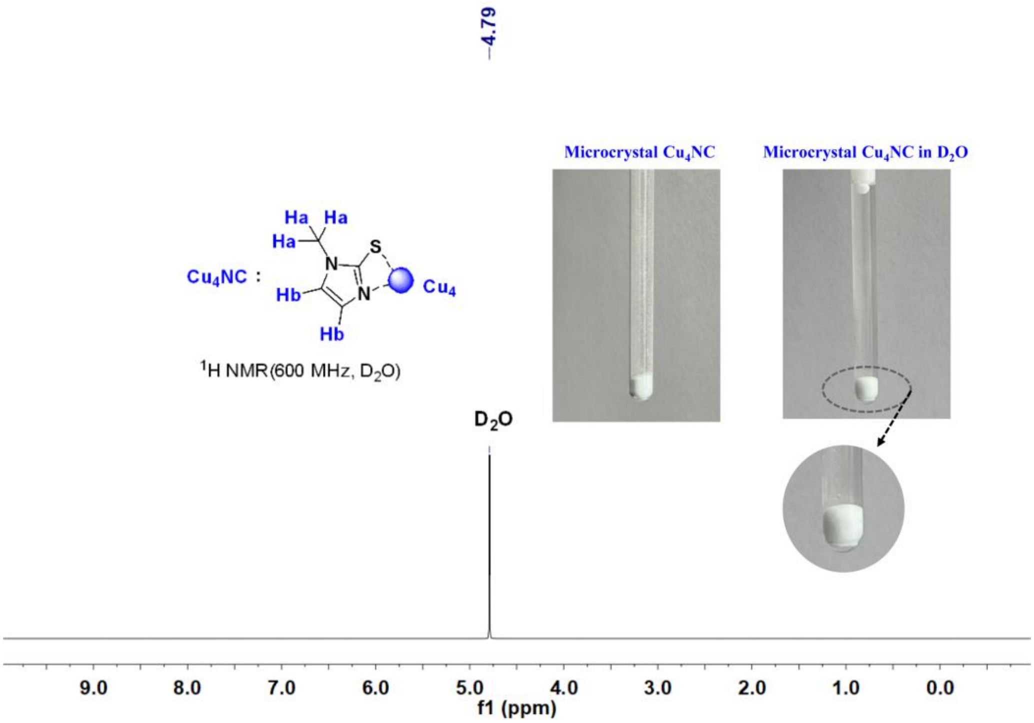

Supplementary Figure 17. ^{1}H NMR spectrum of microcrystalline Cu_4NC in D_2O. No characteristic peaks of Cu_4NC are found in ^{1}H NMR spectrum, proving that microcrystalline Cu_4NC is completely insoluble in H_2O solution.

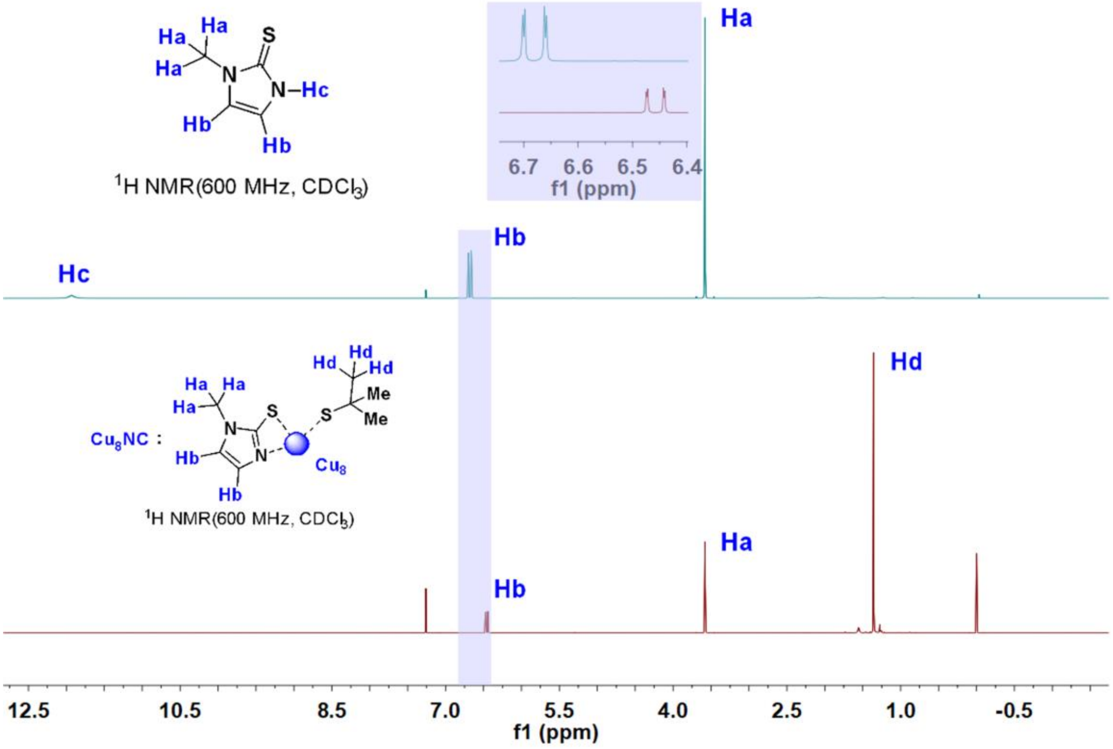

Supplementary Figure 18. ^{1}H NMR spectra of Cu_8NC and ligand. ^{1}H NMR spectra of Cu_8NC and methimazole ligand in $CDCl_3$.

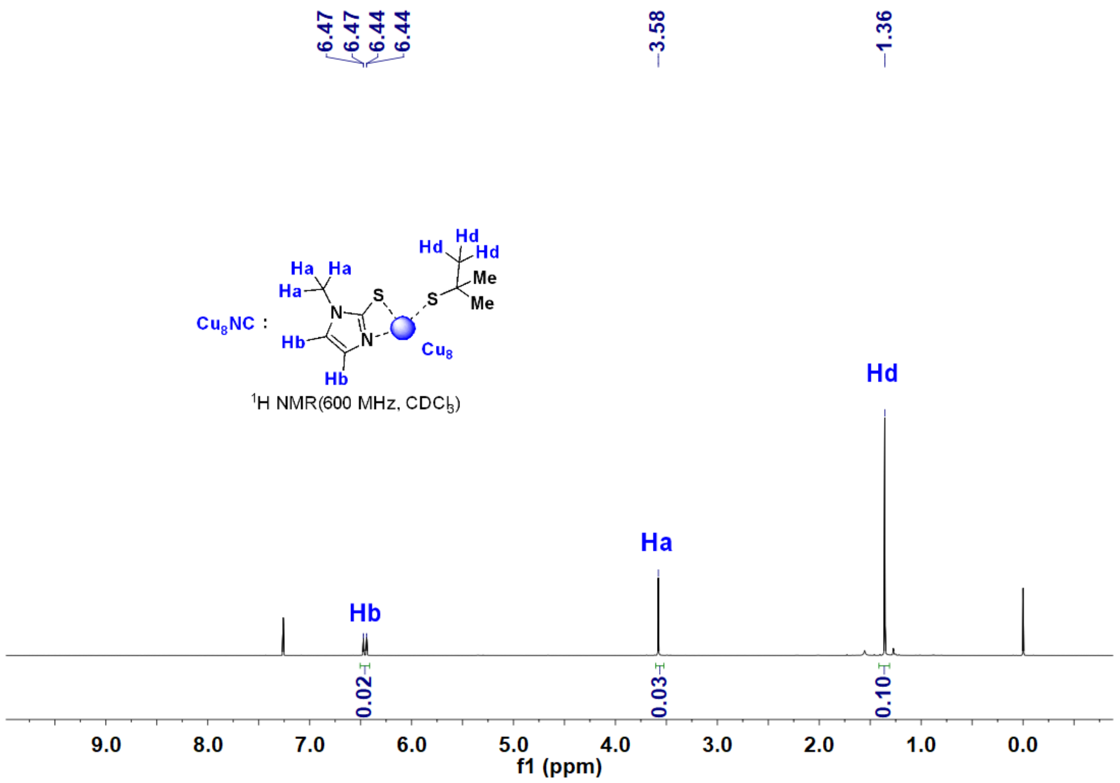

Supplementary Figure 19. ^{1}H NMR spectrum of Cu$_8$NC. ^{1}H NMR spectrum of Cu$_8$NC in $CDCl_3$.

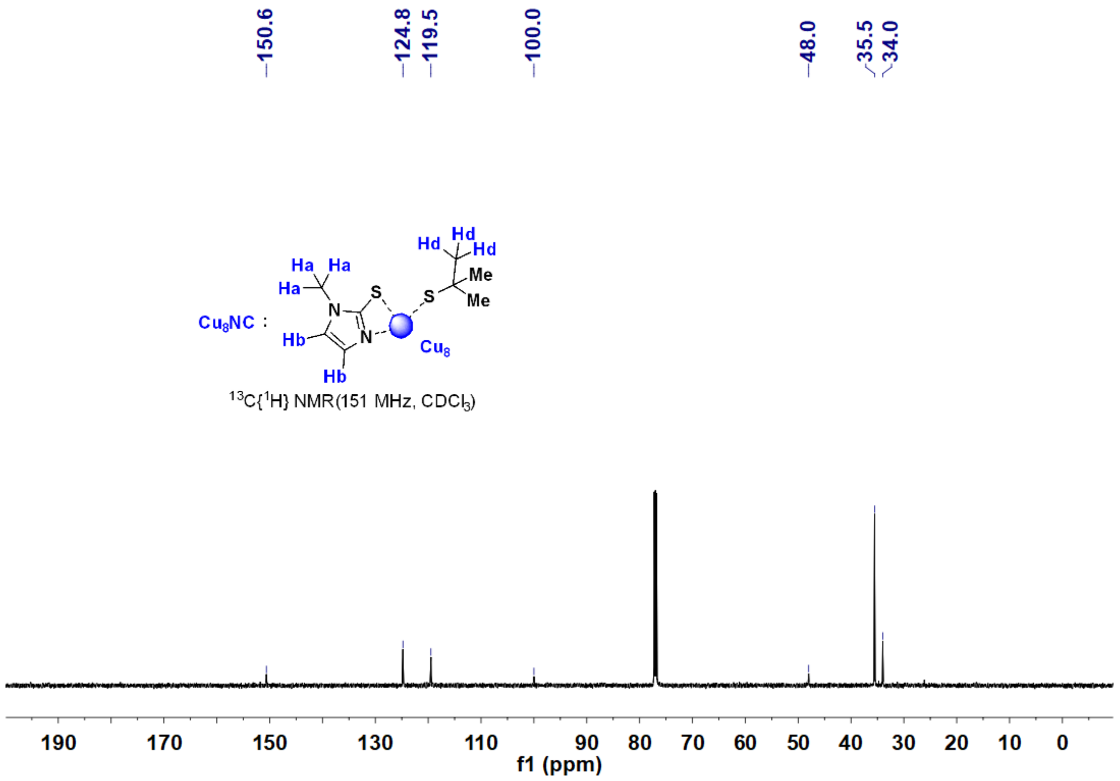

Supplementary Figure 20. ^{13}C{^{1}H} NMR spectrum of Cu$_8$NC. ^{13}C{^{1}H} NMR spectrum of Cu$_8$NC in $CDCl_3$.

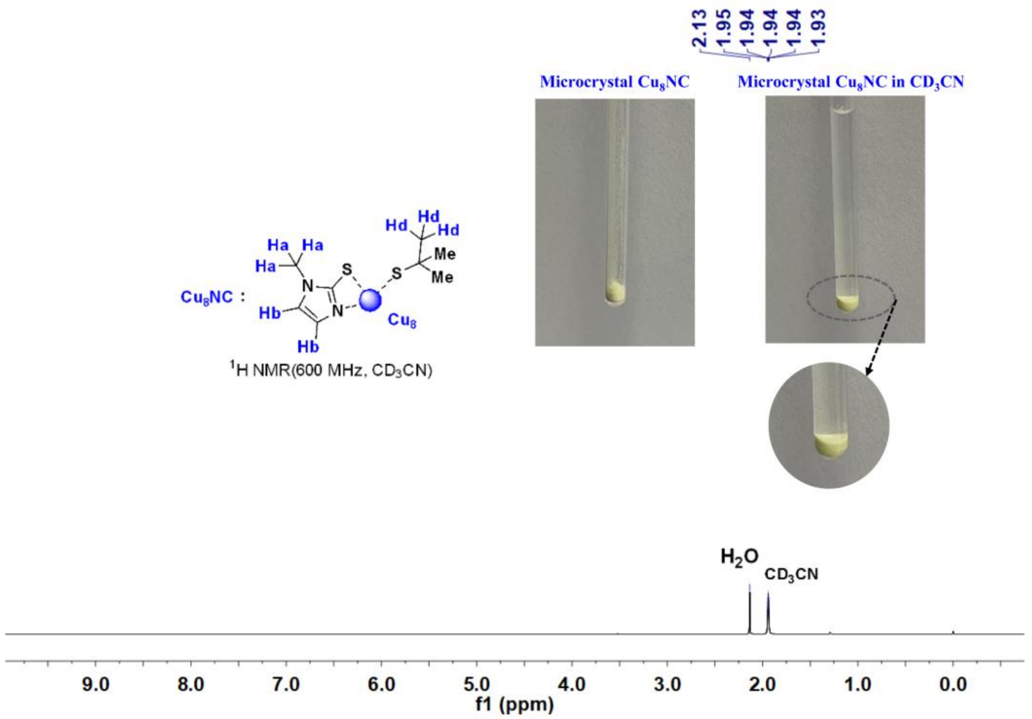

Supplementary Figure 21. ^{1}H NMR spectrum of microcrystalline Cu_8NC in CD_3CN. No characteristic peaks of Cu_8NC are found in ^{1}H NMR spectrum, proving that microcrystalline Cu_8NC is completely insoluble in MeCN solution.

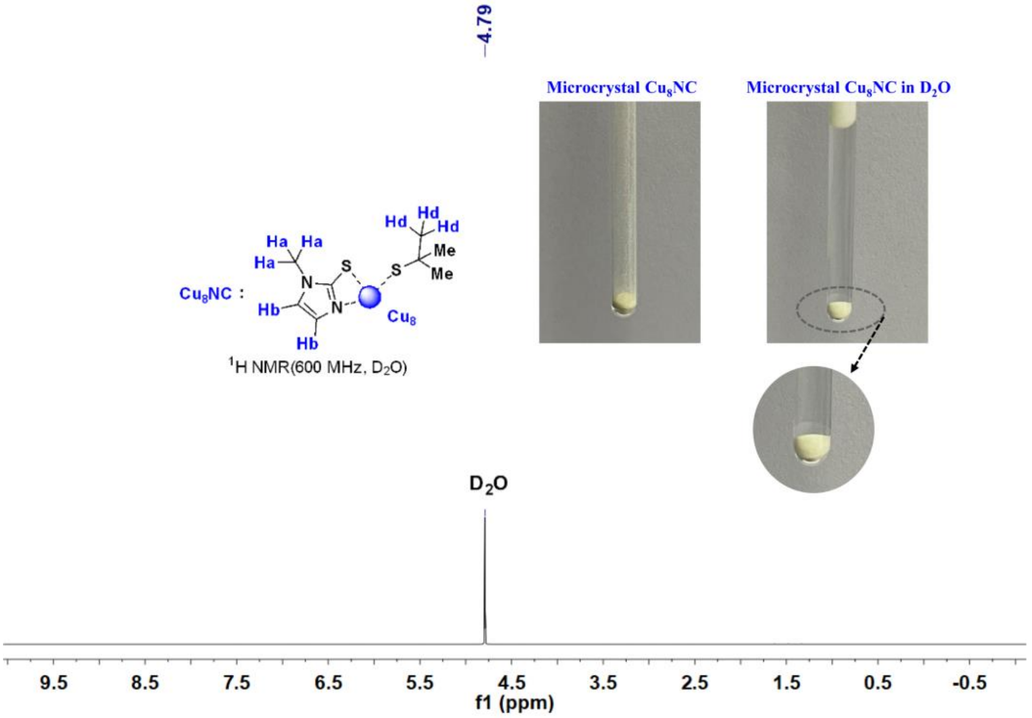

Supplementary Figure 22. ^{1}H NMR spectrum of microcrystalline Cu_8NC in D_2O. No characteristic peaks of Cu_8NC are found in ^{1}H NMR spectrum, proving that microcrystalline Cu_8NC is completely insoluble in H_2O solution.

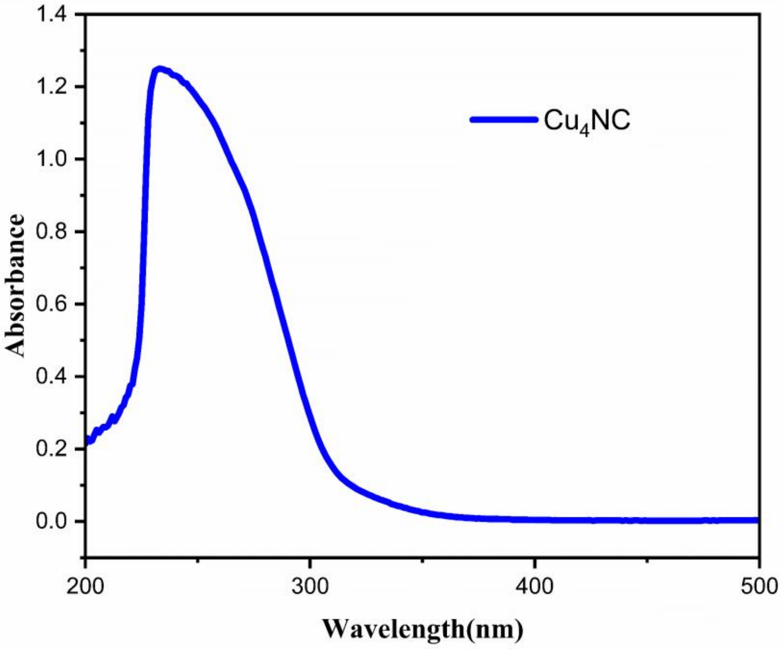

Supplementary Figure 23. Characterization of Cu_4NC. UV–vis spectrum of Cu_4NC in DCM.

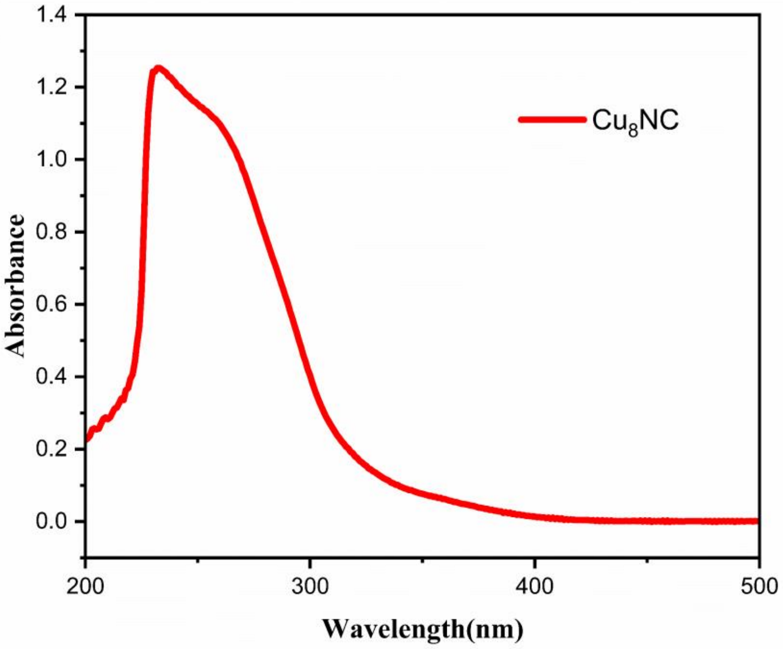

Supplementary Figure 24. Characterization of Cu_8NC. UV–vis spectrum of Cu_8NC in DCM.

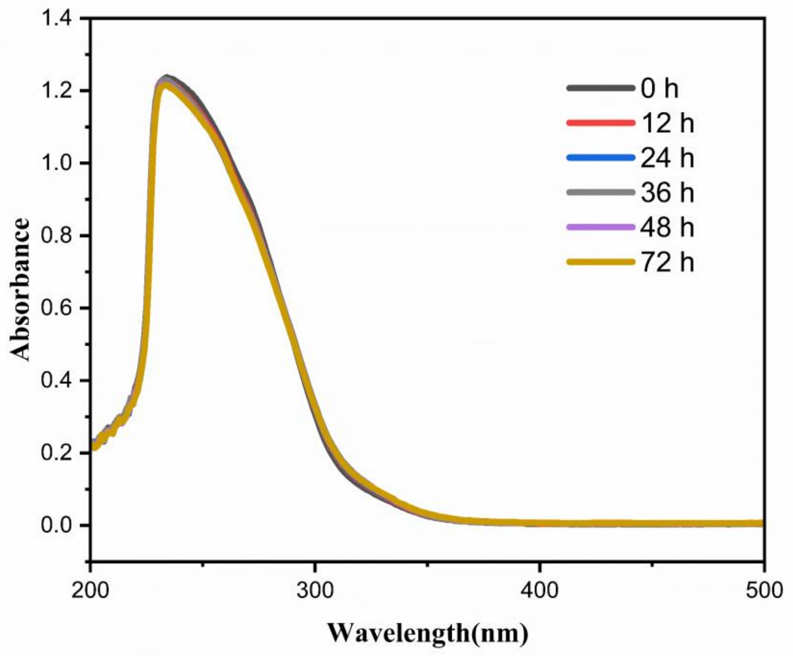

Supplementary Figure 25. Time-dependent UV–vis spectra of Cu_4NC. Time-dependent UV–vis spectra of Cu_4NC in DCM (0-72 h).

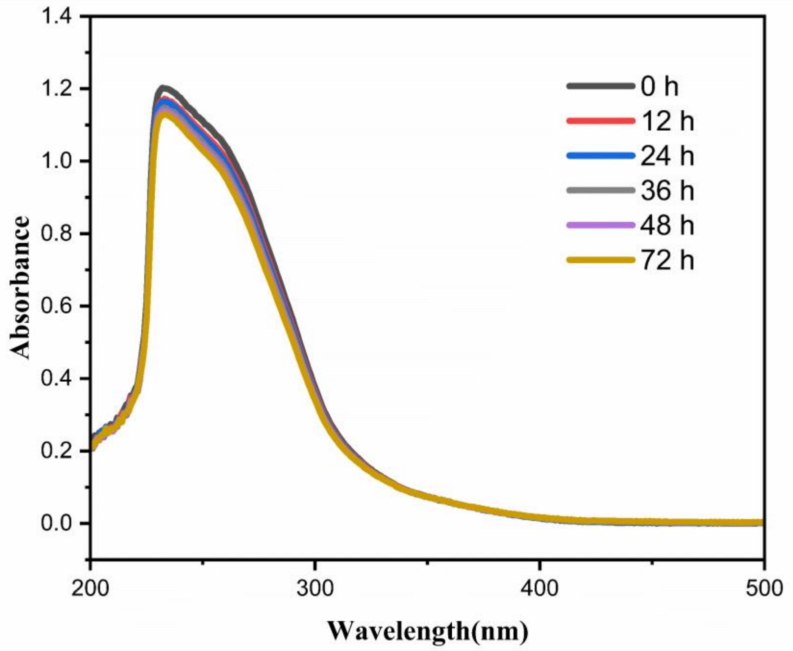

Supplementary Figure 26. Time-dependent UV–vis spectra of Cu_8NC. Time-dependent UV–vis spectra of Cu_8NC in DCM (0-72 h).

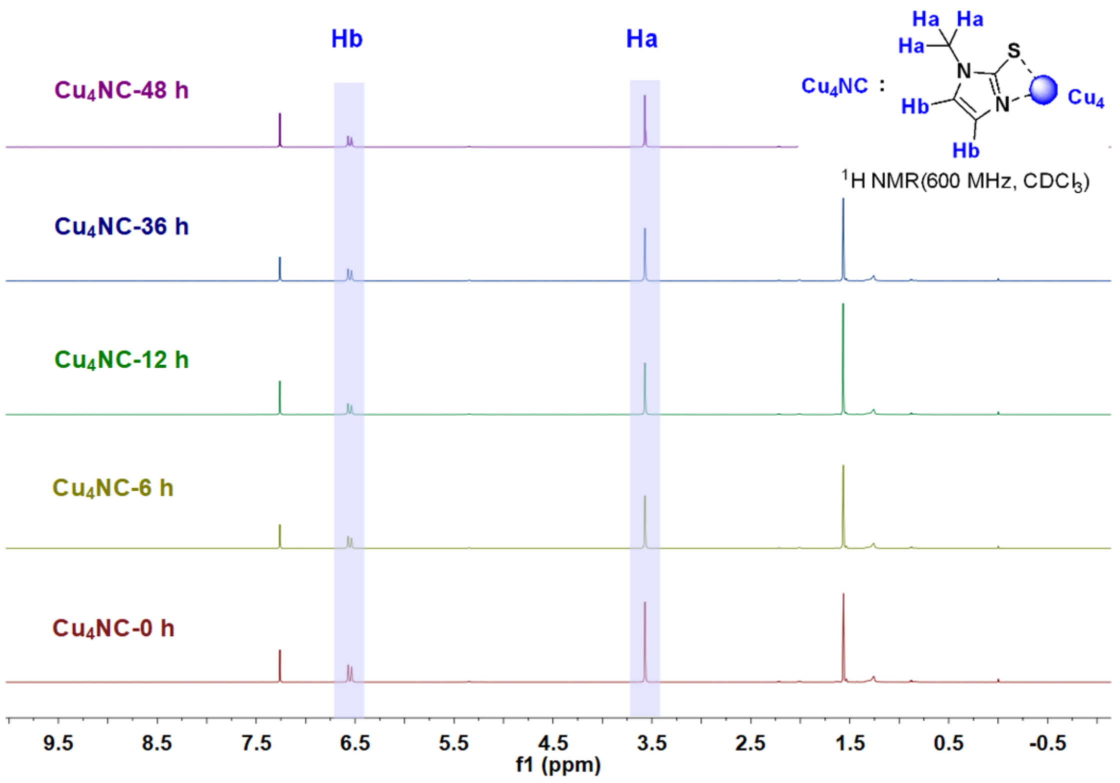

Supplementary Figure 27. ^{1}H NMR spectra tracking of Cu4NC. ^{1}H NMR spectra tracking of Cu4NC in $CDCl_3$ (0-48 h).

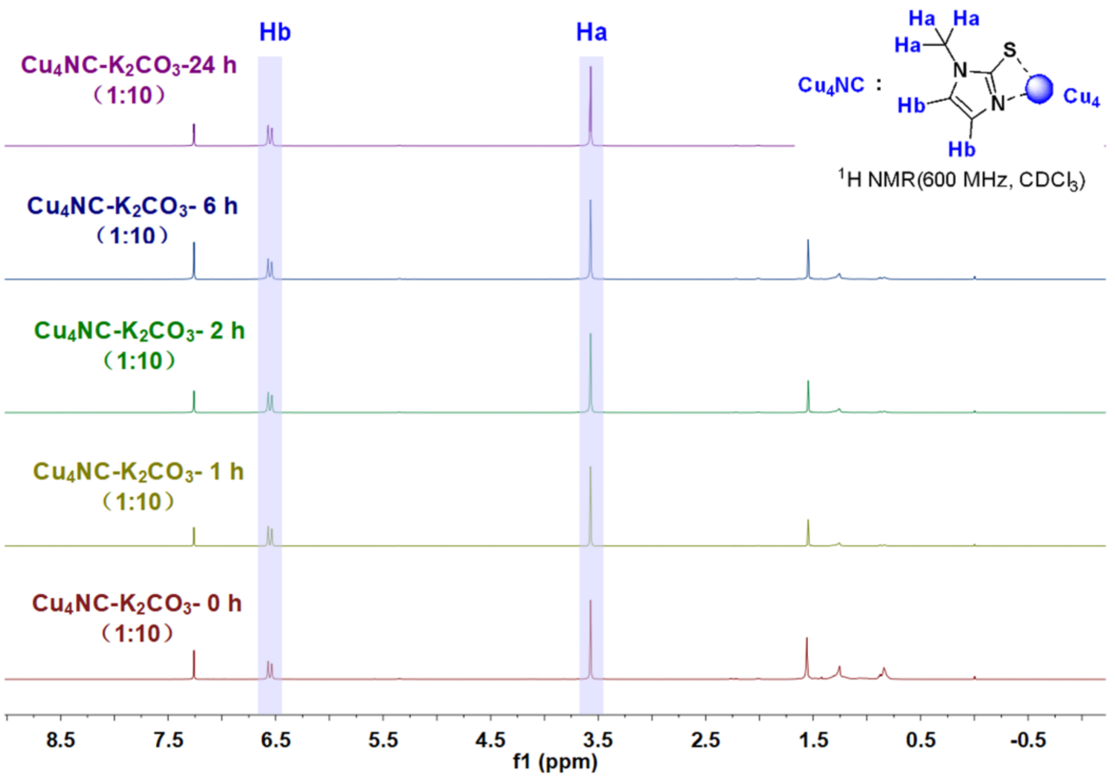

Supplementary Figure 28. ^{1}H NMR spectra tracking of Cu4NC-K_2CO_3. ^{1}H NMR spectra tracking of Cu4NC-K_2CO_3 (1:10) in $CDCl_3$ (0-24 h).

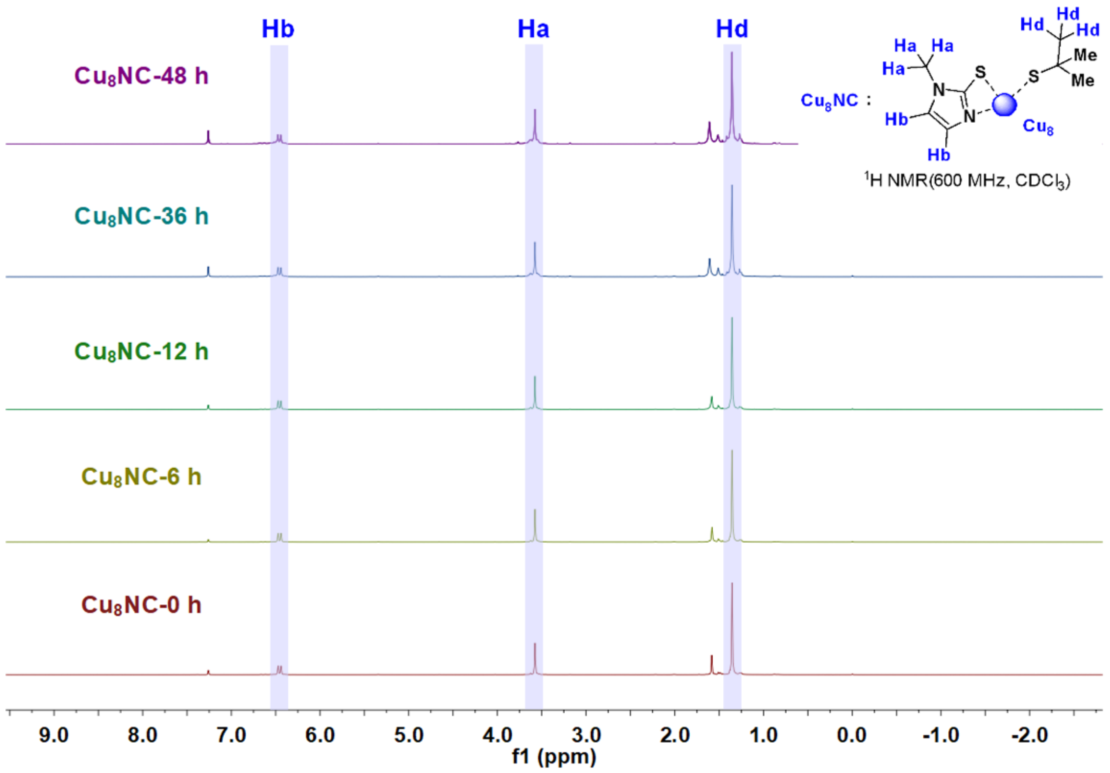

Supplementary Figure 29. ^{1}H NMR spectra tracking of Cu$_8$NC. ^{1}H NMR spectra tracking of Cu$_8$NC in $CDCl_3$ (0-48 h).

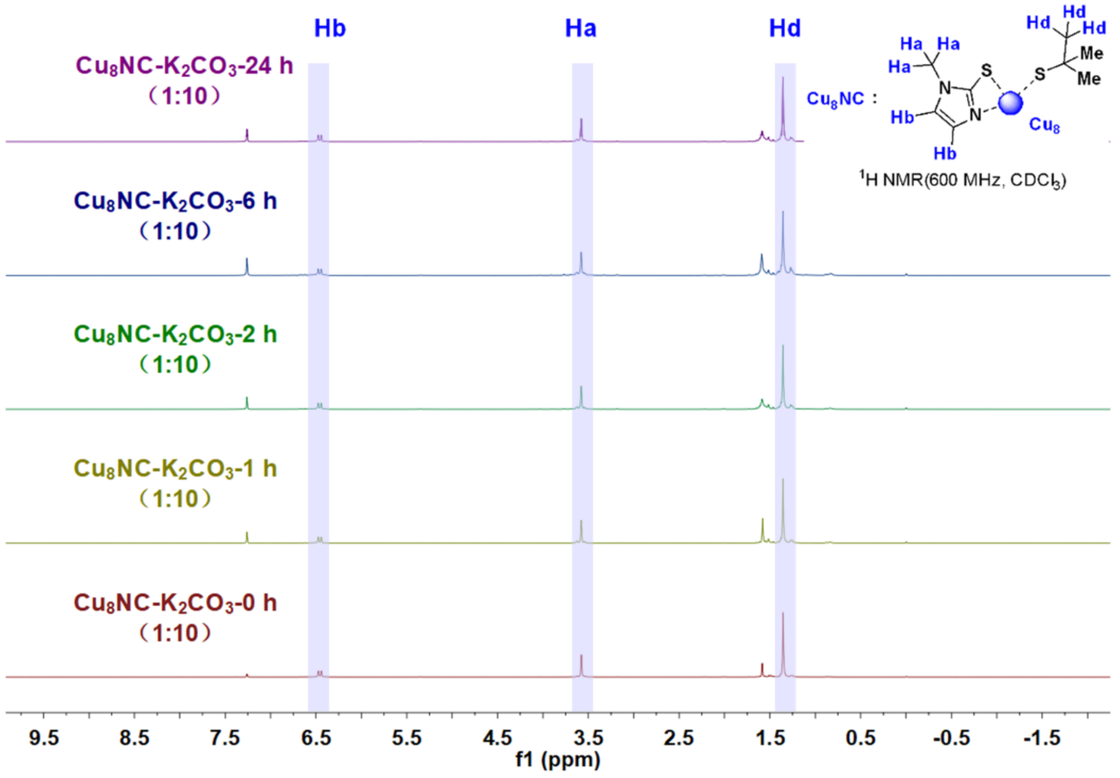

Supplementary Figure 30. ^{1}H NMR spectra tracking of Cu$_8$NC-K_2CO_3. ^{1}H NMR spectra tracking of Cu$_8$NC-K_2CO_3 (1:10) in $CDCl_3$ (0-24 h).

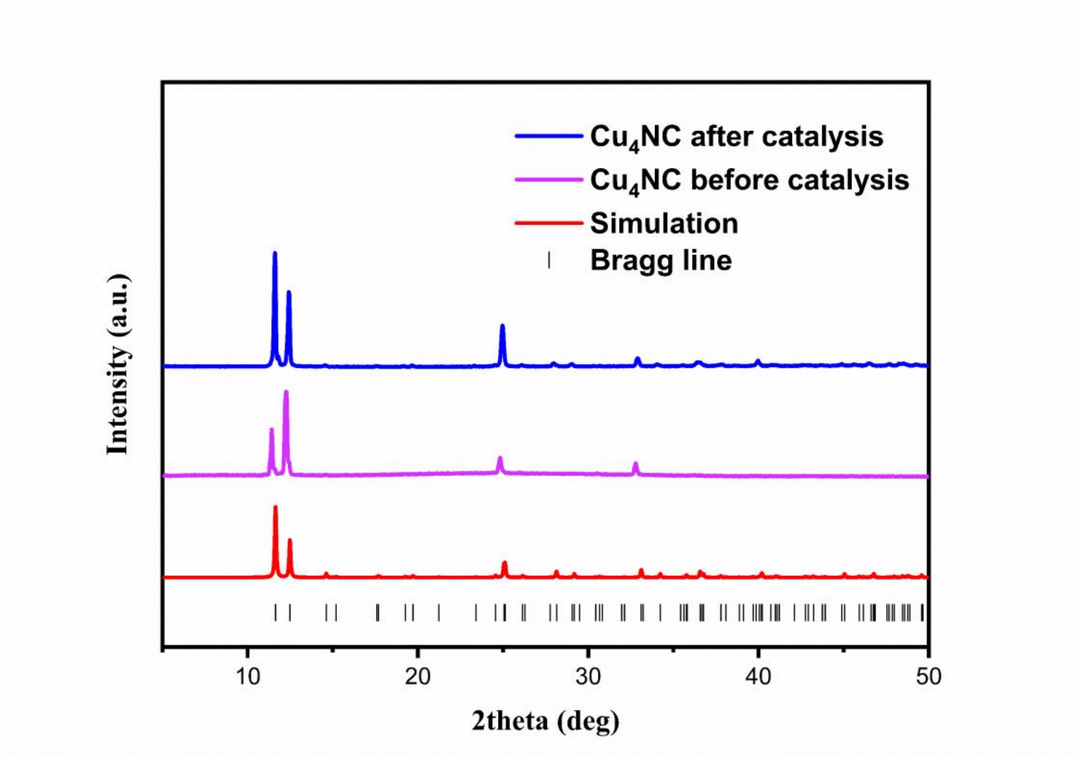

Supplementary Figure 31. PXRD pattern of Cu_4NC. PXRD patterns of microcrystalline Cu_4NC before and after catalysis.

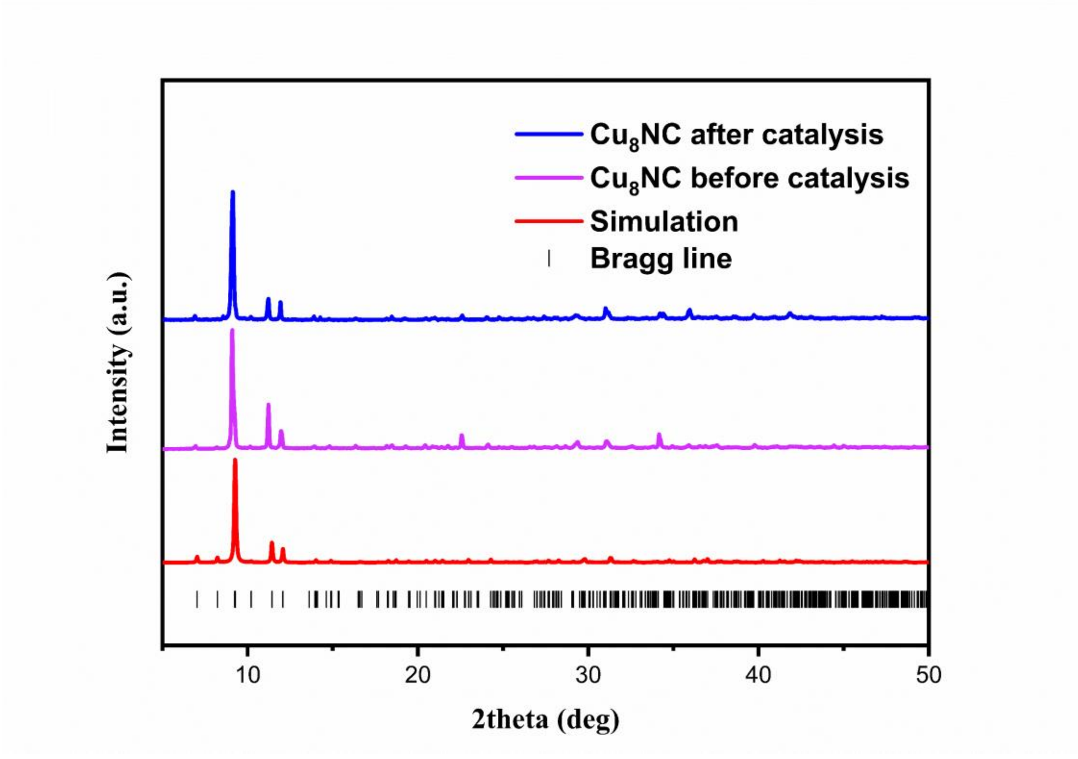

Supplementary Figure 32. PXRD pattern of Cu_8NC. PXRD patterns of microcrystalline Cu_8NC before and after catalysis.

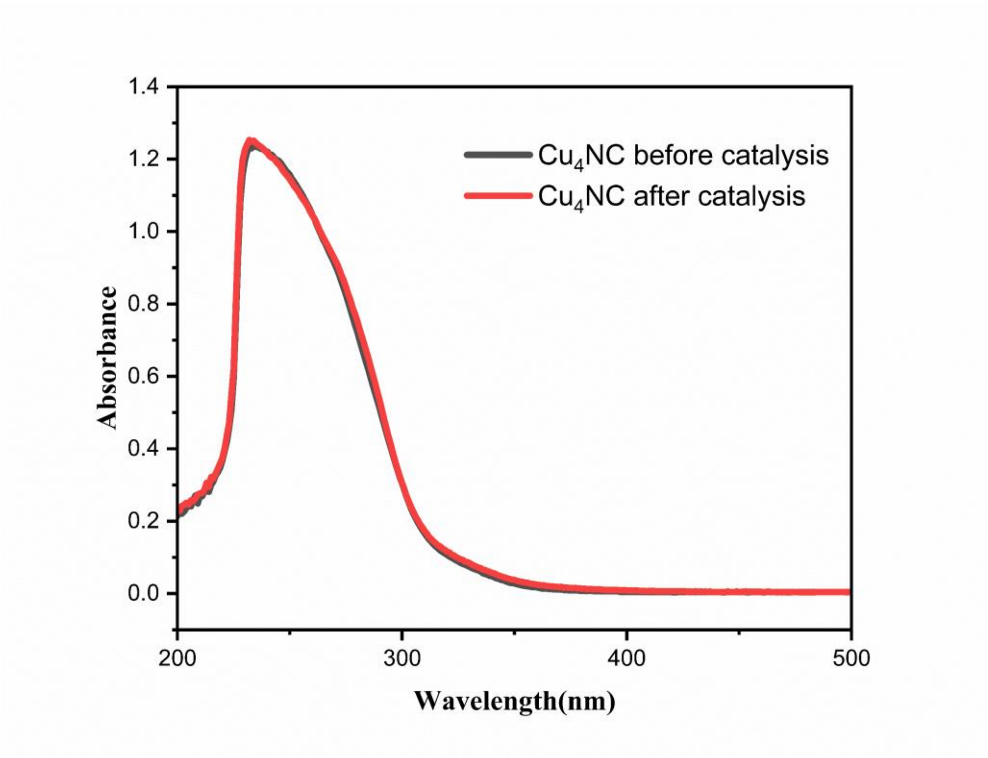

Supplementary Figure 33. Characterization of Cu_4NC before and after catalysis. UV–vis spectra of Cu_4NC before and after catalysis in DCM.

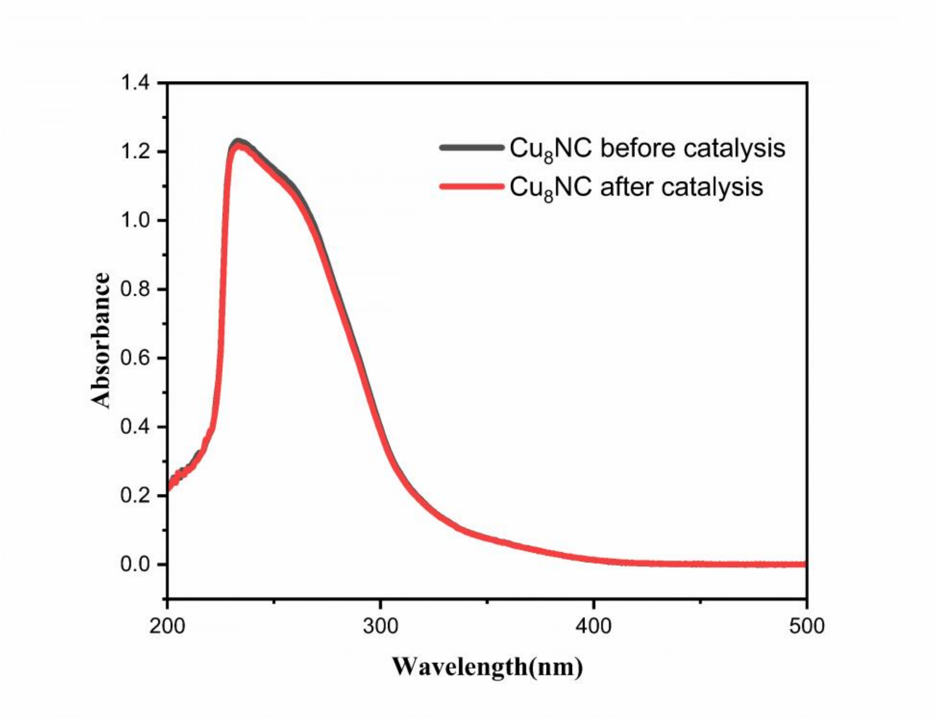

Supplementary Figure 34. Characterization of Cu_8NC before and after catalysis. UV–vis spectra of Cu_8NC before and after catalysis in DCM.

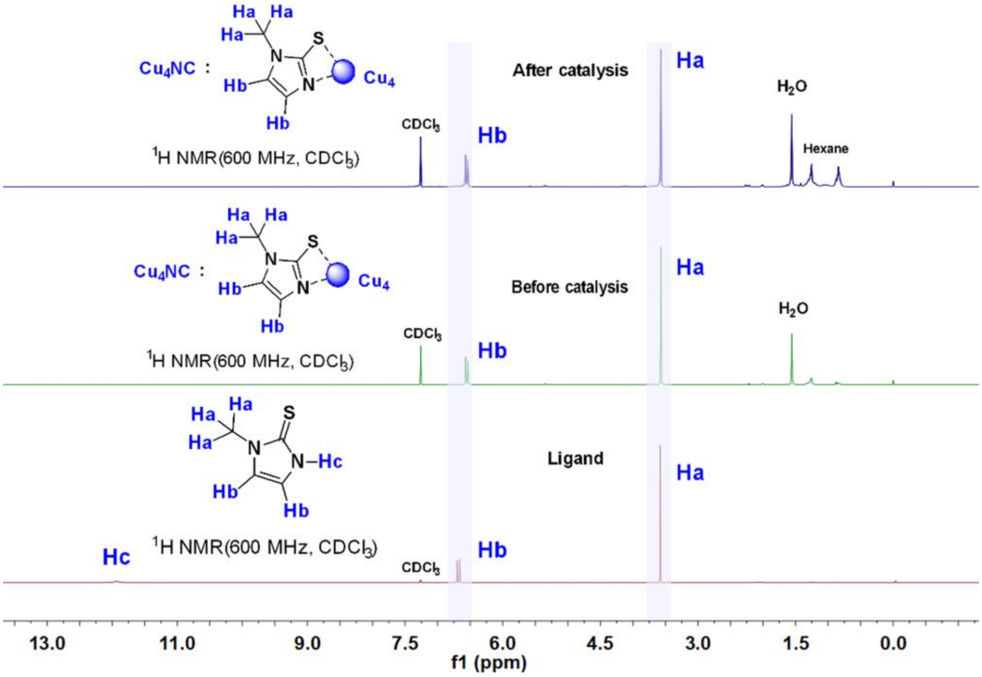

Supplementary Figure 35. ^{1}H NMR spectra of Cu_4NC and methimazole ligand. ^{1}H NMR spectra of Cu_4NC after catalysis (top: recycled microcrystalline Cu_4NC), Cu_4NC before catalysis (middle: new microcrystalline Cu_4NC) and methimazole ligand (bottom) in $CDCl_3$.

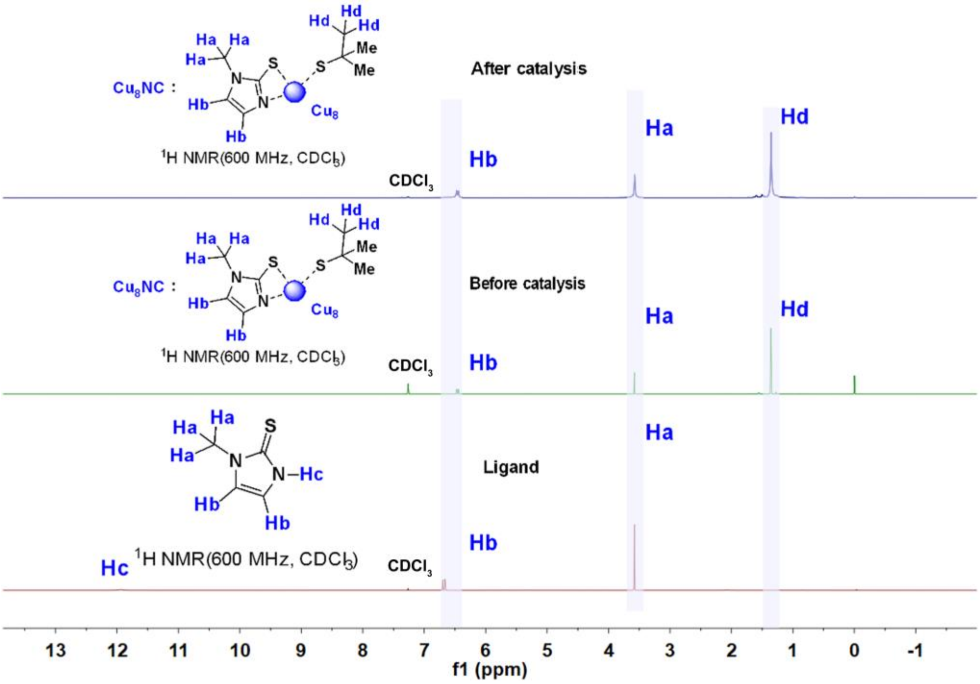

Supplementary Figure 36. ^{1}H NMR spectra of Cu_8NC and methimazole ligand. ^{1}H NMR spectra of Cu_8NC after catalysis (top: recycled microcrystalline Cu_8NC), Cu_8NC before catalysis (middle: new microcrystalline Cu_8NC) and methimazole ligand (bottom) in $CDCl_3$.

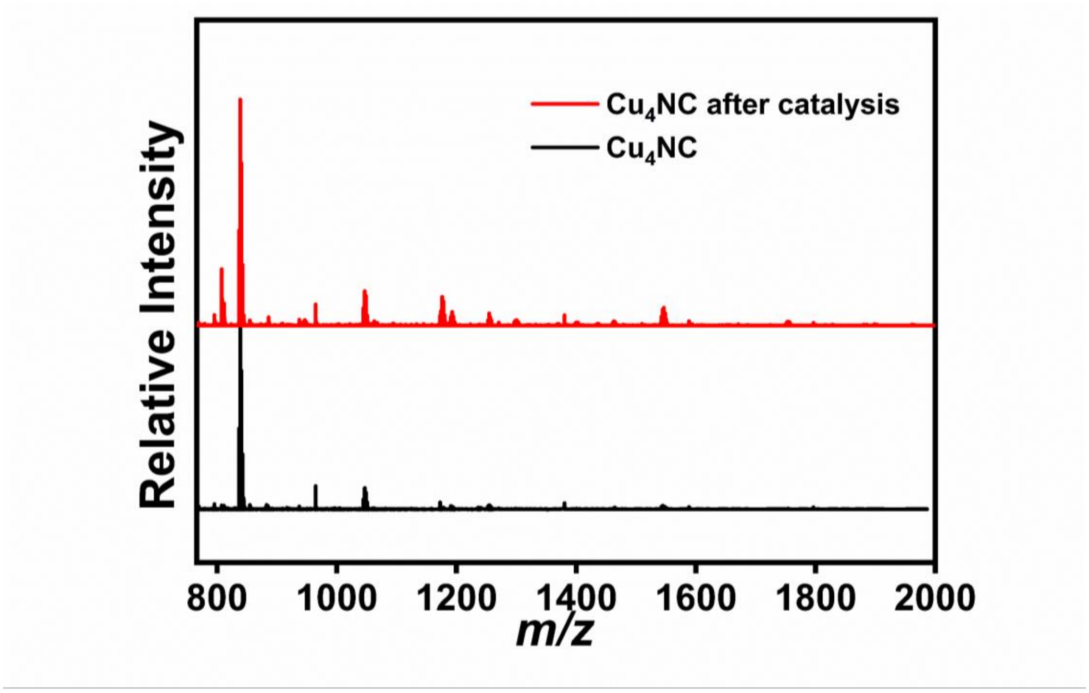

Supplementary Figure 37. ESI–MS spectra of the Cu_4NC. ESI–MS spectra of the Cu_4NC after catalysis (red) and the Cu_4NC before catalysis (black) in the positive mode (DMSO).

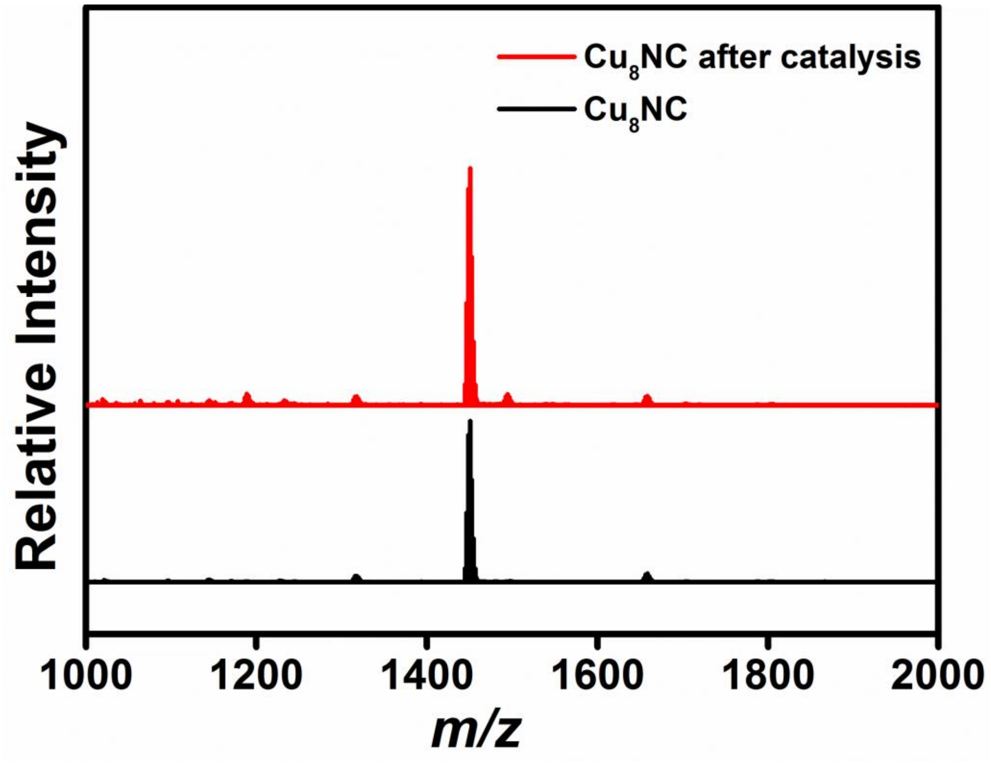

Supplementary Figure 38. ESI–MS spectra of the Cu_8NC. ESI–MS spectra of the Cu_8NC after catalysis (red) and the Cu_8NC before catalysis (black) in the positive mode (DMSO).

General Procedure for Cu_4NC-Catalyzed the Hydroboration Reaction

General Procedure for Cu Catalysts-Catalyzed Hydroboration Reaction (Method A).

Under air atmosphere, alkynes 1 (0.2 mmol, 1.0 eq), B_2Pin_2 2 (0.44 mmol, 2.2 equiv.), Cu catalysts (0 mol%-4.0 mol%), K_2CO_3 (0.44 mmol, 2.2 equiv.) and the mixture solvent (2.0 mL, MeCN-H_2O, v/v 4/1) were added into a tube. The reaction mixture was stirred at room temperature for 1 h. The reactions were monitored by TLC. When alkynes were consumed, the reactions were quenched and concentrated. The crude products were then purified by column chromatography to give the target products.

General Procedure for Cu_4NC-Catalyzed Hydroboration Reaction (Method B).

Under air atmosphere, alkynes 1 (0.2 mmol, 1.0 eq), B_2Pin_2 2 (0.44 mmol, 2.2 equiv.), microcrystalline Cu_4NC catalysts (2.0 mol%), K_2CO_3 (0.44 mmol, 2.2 equiv.) and the mixture solvent (2.0 mL, MeCN-H_2O, v/v 4/1) were added into a tube. The reaction mixture was stirred at room temperature for 1 h or 2 h. The reactions were monitored by TLC. When alkynes were consumed, the reactions were quenched and concentrated. The crude products were then purified by column chromatography to give the target products.

General Procedure for Cu_4NC-Catalyzed Hydroboration Reaction in Dark (Method C).

In dark, alkynes 1 (0.2 mmol, 1.0 eq), B_2Pin_2 2 (0.44 mmol, 2.2 equiv.), microcrystalline Cu_4NC catalysts (2.0 mol%), K_2CO_3 (0.44 mmol, 2.2 equiv.) and the mixture solvent (2.0 mL, MeCN-H_2O, v/v 4/1) were added into a tube under air atmosphere. The reaction mixture was stirred at room temperature for 1 h. The reactions were monitored by TLC. When alkynes were consumed, the reactions were quenched and concentrated. The crude products were then purified by column chromatography to give the target products.

General Procedure for Cu_4NC-Catalyzed Hydroboration Reaction (Method D).

Under N_2 atmosphere, alkynes 1 (0.2 mmol, 1.0 eq), B_2Pin_2 2 (0.44 mmol, 2.2 equiv.), microcrystalline Cu_4NC catalysts (2.0 mol%), K_2CO_3 (0.44 mmol, 2.2 equiv.) and the mixture solvent (2.0 mL, MeCN-H_2O, v/v 4/1) were added into a tube. The reaction mixture was stirred at room temperature for 1 h. The reactions were monitored by TLC. When alkynes were consumed, the reactions were quenched and concentrated. The crude products were then purified by column chromatography to give the target products.

General Procedure for Cu_4NC-Catalyzed Hydroboration Reaction (Method E).

Under air atmosphere, alkynes 1 (0.2 mmol, 1.0 eq), B_2Pin_2 2 (0.44 mmol, 2.2 equiv.), microcrystalline Cu_4NC catalysts (2.0 mol%), base (0 mmol-0.44 mmol, 0 equiv.-2.2 equiv.) and the mixture solvent (2.0 mL, MeCN-H_2O, v/v 4/1) were added into a tube.

The reaction mixture was stirred at room temperature for 1 h. The reactions were monitored by TLC. When alkynes were consumed, the reactions were quenched and concentrated. The crude products were then purified by column chromatography to give the target products.

General Procedure for Cu_4NC-Catalyzed Hydroboration Reaction (Method F).

Under air atmosphere, alkynes 1 (0.2 mmol, 1.0 eq), B_2Pin_2 2 (0.44 mmol, 2.2 equiv.), microcrystalline Cu_4NC catalysts (2.0 mol%), K_2CO_3 (0.44 mmol, 2.2 equiv.) and kinds of solvent (2.0 mL) were added into a tube. The reaction mixture was stirred at room temperature for 1 h. The reactions were monitored by TLC. When alkynes were consumed, the reactions were quenched and concentrated. The crude products were then purified by column chromatography to give the target products.

General Procedure for Cu_4NC-Catalyzed Hydroboration Reaction (Method G).

Under air atmosphere, alkynes 1 (0.1 mmol, 1.0 eq), B_2Pin_2 2 (0.44 mmol, 4.4 equiv.), microcrystalline Cu_4NC catalysts (2.0 mol%), K_2CO_3 (0.44 mmol, 4.4 equiv.) and the mixture solvent (2.0 mL, MeCN-H_2O, v/v 4/1) were added into a tube. The reaction mixture was stirred at room temperature for 1 h. The reactions were monitored by TLC. When alkynes were consumed, the reactions were quenched and concentrated. The crude products were then purified by column chromatography to give products.

General Procedure for Cu_4NC-Catalyzed Hydroboration Reaction (Method H).

Under air atmosphere, alkynes 1 (0.2 mmol, 1.0 eq), alkenes (0.2 mmol, 1.0 eq), B_2Pin_2 2 (0.44 mmol, 2.2 equiv.), microcrystalline Cu_4NC catalysts (2.0 mol%), K_2CO_3 (0.44 mmol, 2.2 equiv.) and the mixture solvent (2.0 mL, MeCN-H_2O, v/v 4/1) were added into a tube. The reaction mixture was stirred at room temperature for 1 h. The reactions were monitored by TLC. When alkynes or alkenes were consumed, the reactions were quenched and concentrated. The crude products were then purified by column chromatography to give the target products.

General Procedure for Cu_4NC-Catalyzed Hydroboration Reaction in 55 mmol Scale.

Under air atmosphere, phenylacetylene **1** (5615.5 mg, 55.0 mmol, 1.0 eq), B_2Pin_2 **2** (30721.9 mg, 121.0 mmol, 2.2 equiv.), microcrystalline Cu_4NC catalysts (0.5 mg, 7×10^{-4} mmol, 1.3×10^{-3} mol%), K_2CO_3 (8361.7 mg, 60.5 mmol, 1.1 equiv.) and the mixture solvent (55.0 mL, MeCN-H_2O, v/v 4/1) were added into a 100mL flask. The reaction mixture was stirred at room temperature for 12.5 h. The reactions were monitored by TLC. When alkynes were consumed, the reactions were quenched and concentrated. The crude products were then purified by column chromatography to give the (E)-4,4,5,5-tetramethyl-2-styryl-1,3,2-dioxaborolane **(E-4a)** products as colorless oil with an overall isolated yield: 98% (12396.8 mg).

Investigation on the Lowest Load of Cu_4NC Catalyst for the Hydroboration Transformation

Supplementary Table 1. Investigation on the lowest load of microcrystalline Cu_4NC catalyst for the hydroboration of phenylacetylene under the different conditions.

Hydroboration of alkyne

Entry	Time	Phenylacetylene	Cat.: Cu_4NC	Yield[k]	TON[l]	TOF (h^{-1})[m]
1[a]	1 h	10 mmol	7×10^{-4} mmol	76%	10857	10857
2[a]	2 h	10 mmol	7×10^{-4} mmol	100%	14286	7143
3[b]	1 h	15 mmol	7×10^{-4} mmol	62%	13286	13286
4[b]	2 h	15 mmol	7×10^{-4} mmol	88%	18857	9429
5[b]	3 h	15 mmol	7×10^{-4} mmol	100%	21429	7143
6[c]	1 h	20 mmol	7×10^{-4} mmol	57%	16286	16286
7[c]	3 h	20 mmol	7×10^{-4} mmol	90%	25714	8571
8[c]	4 h	20 mmol	7×10^{-4} mmol	100%	28571	7143
9[d]	1 h	25 mmol	7×10^{-4} mmol	44%	15714	15714
10[d]	3 h	25 mmol	7×10^{-4} mmol	87%	31071	10357
11[d]	4 h	25 mmol	7×10^{-4} mmol	100%	35714	8929
10[e]	1 h	30 mmol	7×10^{-4} mmol	35%	15000	15000
10[e]	2 h	30 mmol	7×10^{-4} mmol	51%	21857	10929
11[e]	4 h	30 mmol	7×10^{-4} mmol	78%	33429	8357
12[e]	7 h	30 mmol	7×10^{-4} mmol	100%	42857	6122
13[f]	4 h	35 mmol	7×10^{-4} mmol	75%	37500	9375
14[f]	8 h	35 mmol	7×10^{-4} mmol	100%	50000	6250
15[g]	9.2 h	40 mmol	7×10^{-4} mmol	100%	57143	6211
16[h]	10.3 h	45 mmol	7×10^{-4} mmol	100%	64286	6241
17[i]	11.5 h	50 mmol	7×10^{-4} mmol	100%	71429	6211
18[j]	12.5 h	55 mmol	7×10^{-4} mmol	99%	**77786**	6223

.[a]General reaction conditions: Under air atmosphere, alkynes **1** (10 mmol, 1.0 eq), B_2Pin_2 **2** (22.0 mmol, 2.2 equiv.), microcrystalline Cu_4NC catalysts (7×10^{-4} mmol), K_2CO_3 (22.0 mmol, 2.2 equiv.) and the mixture solvent (10.0 mL, MeCN-H_2O, v/v 4/1) were added into a 30 mL vial. The reaction mixture was stirred at room temperature. [b]General reaction conditions: Under air atmosphere, alkynes **1** (15 mmol, 1.0 eq), B_2Pin_2 **2** (33.0 mmol, 2.2 equiv.), microcrystalline Cu_4NC catalysts (7×10^{-4} mmol), K_2CO_3 (33.0 mmol, 2.2 equiv.) and the mixture solvent (30.0 mL, MeCN-H_2O, v/v 4/1) were added into a 50 mL flask. The reaction mixture was stirred at room temperature. [c]General reaction conditions: Under air atmosphere, alkynes **1** (20 mmol, 1.0 eq), B_2Pin_2 **2** (44.0 mmol, 2.2 equiv.), microcrystalline Cu_4NC catalysts (7×10^{-4} mmol), K_2CO_3 (33.0 mmol, 1.65 equiv.) and the mixture solvent (40.0 mL, MeCN-H_2O, v/v 4/1) were added into a 50 mL flask. The reaction mixture was stirred at room temperature. [d]General reaction conditions: Under air atmosphere, alkynes **1** (25 mmol, 1.0 eq), B_2Pin_2 **2** (55.0 mmol, 2.2 equiv.), microcrystalline Cu_4NC catalysts (7×10^{-4} mmol), K_2CO_3 (33.0 mmol, 1.32 equiv.) and the mixture solvent (30.0 mL, MeCN-H_2O, v/v 4/1) were added into a 50 mL flask. The reaction mixture was stirred at room temperature. [e]General reaction conditions: Under air atmosphere, alkynes **1** (30 mmol, 1.0 eq), B_2Pin_2 **2** (66.0 mmol, 2.2 equiv.), microcrystalline Cu_4NC catalysts (7×10^{-4} mmol), K_2CO_3 (33.0 mmol, 1.1 equiv.) and the mixture solvent (30.0 mL, MeCN-H_2O, v/v 4/1) were added into a 50 mL flask. The reaction mixture was stirred at room temperature. [f]General reaction conditions: Under air atmosphere, alkynes **1** (35 mmol, 1.0 eq), B_2Pin_2 **2** (77.0 mmol, 2.2 equiv.),

microcrystalline Cu_4NC catalysts (7×10^{-4} mmol), K_2CO_3 (38.5 mmol, 1.1 equiv.) and the mixture solvent (40.0 mL, MeCN-H_2O, v/v 4/1) were added into a 100 mL flask. The reaction mixture was stirred at room temperature. [g]Yield was determined by ^{1}H NMR using 1,3,5-trimethoxybenzene as an internal standard. [g]General reaction conditions: Under air atmosphere, alkynes **1** (40 mmol, 1.0 eq), B_2Pin_2 **2** (88.0 mmol, 2.2 equiv.), microcrystalline Cu_4NC catalysts (7×10^{-4} mmol), K_2CO_3 (44.0 mmol, 1.1 equiv.) and the mixture solvent (40.0 mL, MeCN-H_2O, v/v 4/1) were added into a 100 mL flask. The reaction mixture was stirred at room temperature. [h]General reaction conditions: Under air atmosphere, alkynes **1** (45 mmol, 1.0 eq), B_2Pin_2 **2** (99.0 mmol, 2.2 equiv.), microcrystalline Cu_4NC catalysts (7×10^{-4} mmol), K_2CO_3 (49.5 mmol, 1.1 equiv.) and the mixture solvent (45.0 mL, MeCN-H_2O, v/v 4/1) were added into a 100 mL flask. The reaction mixture was stirred at room temperature. [i]General reaction conditions: Under air atmosphere, alkynes **1** (50 mmol, 1.0 eq), B_2Pin_2 **2** (110.0 mmol, 2.2 equiv.), microcrystalline Cu_4NC catalysts (7×10^{-4} mmol), K_2CO_3 (55.0 mmol, 1.1 equiv.) and the mixture solvent (50.0 mL, MeCN-H_2O, v/v 4/1) were added into a 100 mL flask. The reaction mixture was stirred at room temperature. [j]General reaction conditions: Under air atmosphere, alkynes **1** (55 mmol, 1.0 eq), B_2Pin_2 **2** (121.0 mmol, 2.2 equiv.), microcrystalline Cu_4NC catalysts (7×10^{-4} mmol), K_2CO_3 (60.5 mmol, 1.1 equiv.) and the mixture solvent (55.0 mL, MeCN-H_2O, v/v 4/1) were added into a 100 mL flask. The reaction mixture was stirred at room temperature. [k]Yield was determined by ^{1}H NMR using 1,3,5-trimethoxybenzene as an internal standard. [l]TON = (Moles of reactants) × (Yield %) / (Moles of catalysts). [m]TOF = TON / Time (h).

Catalytic Mechanism Studies by a Series of Control Experiments

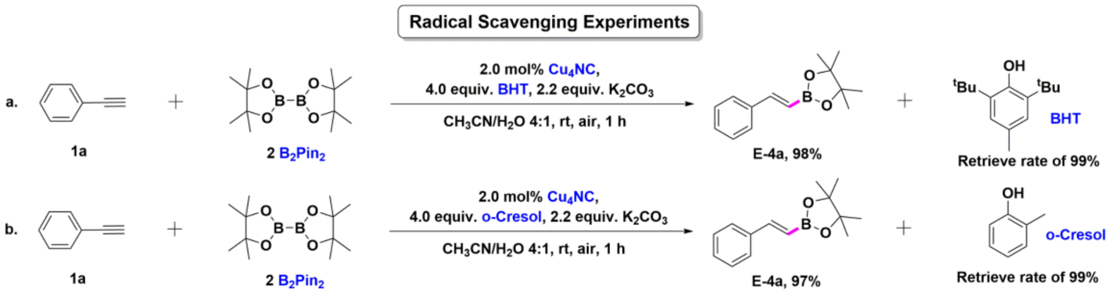

Supplementary Figure 39. Radical scavenging experiments of microcrystalline Cu_4NC-catalyzed hydroboration reaction. a. The BHT as radical scavenger in radical scavenging experiment. **b.** The *o*-cresol as radical scavenger in radical scavenging experiment.

Radical scavenging experiment a. Under air atmosphere, phenylacetylene **1a** (20.4 mg, 0.2 mmol, 1.0 eq), BHT (176.3 mg, 0.8 mmol, 4.0 eq), B_2Pin_2 **2** (111.8 mg, 0.44 mmol, 2.2 equiv.), microcrystalline Cu_4NC catalysts (2.8 mg, 2.0 mol%), K_2CO_3 (60.7 mg, 0.44 mmol, 2.2 equiv.) and the mixture solvent (2.0 mL, CD_3CN-H_2O, v/v 4/1) were added into a tube. The reaction mixture was stirred at room temperature for 1 h. The reactions were monitored by TLC. When alkynes were consumed, the reactions were quenched and concentrated. Conversion and yield were determined by ^{1}H NMR using 1,3,5-trimethoxybenzene as an internal standard. The crude products were then purified by column chromatography (PE-EA, v/v 20/1) to give the target product **E-4a** as a colorless oil with an overall isolated yield: 98%. The retrieve rate of BHT is 99%.

Radical scavenging experiment b. Under air atmosphere, phenylacetylene **1a** (20.4 mg, 0.2 mmol, 1.0 eq), *o*-cresol (86.5 mg, 0.8 mmol, 4.0 eq), B_2Pin_2 2 (111.8 mg, 0.44 mmol, 2.2 equiv.), microcrystalline Cu_4NC catalysts (2.8 mg, 2.0 mol%), K_2CO_3 (60.7

mg, 0.44 mmol, 2.2 equiv.) and the mixture solvent (2.0 mL, CD_3CN-H_2O, v/v 4/1) were added into a tube. The reaction mixture was stirred at room temperature for 1 h. The reactions were monitored by TLC. When alkynes were consumed, the reactions were quenched and concentrated. Conversion and yield were determined by ^{1}H NMR using 1,3,5-trimethoxybenzene as an internal standard. The crude products were then purified by column chromatography (PE-EA, v/v 20/1) to give the target product **E-4a** as a colorless oil with an overall isolated yield: 97%. The retrieve rate of o-cresol is 99%.

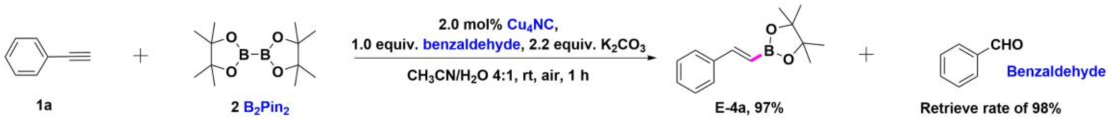

Supplementary Figure 40. The control experiment. The control experiment of microcrystalline Cu_4NC-catalyzed the hydroboration reaction.

Under air atmosphere, phenylacetylene **1a** (20.4 mg, 0.2 mmol, 1.0 eq), benzaldehyde (21.2 mg, 0.2 mmol, 1.0 eq), B_2Pin_2 **2** (111.8 mg, 0.44 mmol, 2.2 equiv.), microcrystalline Cu_4NC catalysts (2.8 mg, 2.0 mol%), K_2CO_3 (60.7 mg, 0.44 mmol, 2.2 equiv.) and the mixture solvent (2.0 mL, CD_3CN-H_2O, v/v 4/1) were added into a tube. The reaction mixture was stirred at room temperature for 1 h. The reactions were monitored by TLC. When alkynes were consumed, the reactions were quenched and concentrated. Conversion and yield were determined by ^{1}H NMR using 1,3,5-trimethoxybenzene as an internal standard. The crude products were then purified by column chromatography (PE-EA, v/v 20/1) to give the target product **E-4a** as a colorless oil with an overall isolated yield: 97%. The retrieve rate of benzaldehyde is 98%.

To prove that the carbonyl of benzaldehyde remains intact in our catalytic system, we carried out the NMR experiment. As reviewer suggested, 2.0 mol% microcrystalline Cu_4NC, 1.0 eq. *p*-tolualdehyde, 2.2 eq. B_2Pin_2 and 2.2 eq. K_2CO_3 were added into the mixture solvent (CH_3CN-H_2O). The *in situ* ^{1}H NMR was monitored at the 0 minute, the 3rd minute, 5th minute, the 10th minute, 15th minute, the 20th minute, 30th minute, the 40th minute, 50th minute and the 60th minute, showing that characteristic peaks of *p*-tolualdehyde have no change (Supplementary Figure 41). The result illustrated that *p*-tolualdehyde is inert substrate to our catalytic system, indicating that the carbonyl remains intact our catalytic system.

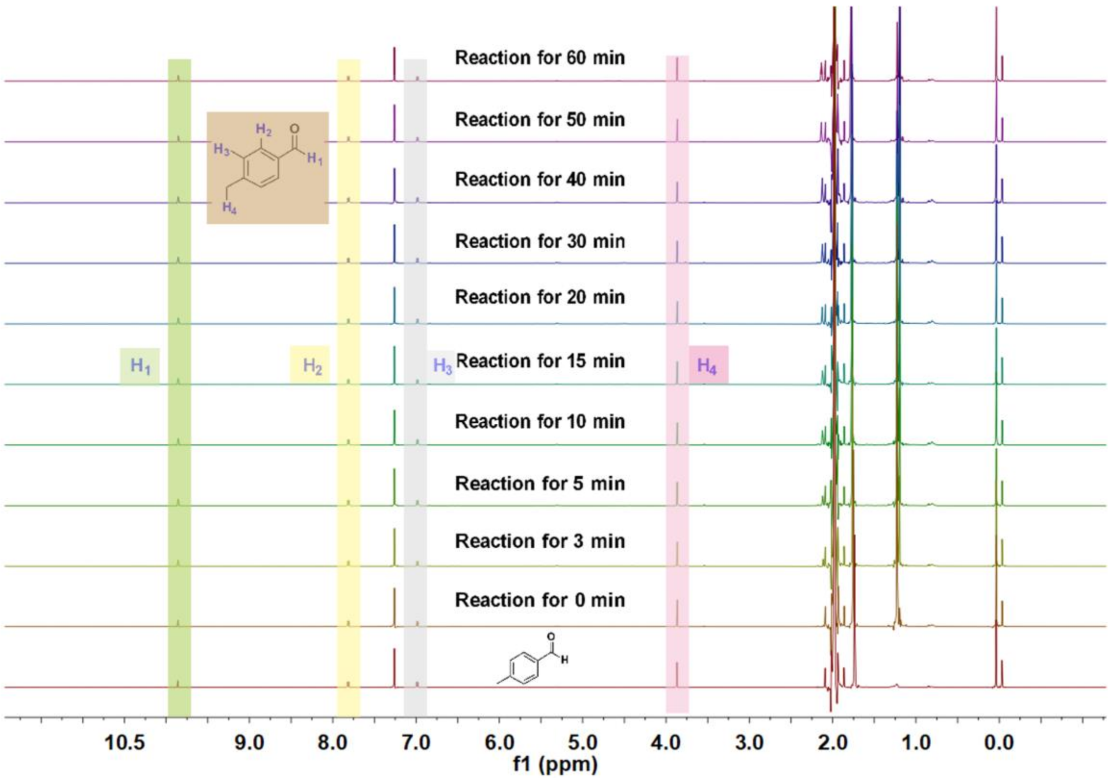

Supplementary Figure 41. Time-dependent *in situ* ^{1}H NMR spectra of the reaction catalyzed by microcrystalline Cu4NC catalyst. Reaction conditions: *p*-tolualdehyde (0.2 mmol, 1.0 equiv.), B_2Pin_2 2 (0.44 mmol, 2.2 equiv.), Cu4NC catal. (0.004 mmol, 2.0 mol%) and K_2CO_3 (0.44 mmol, 2.2 equiv.) were added to the mixture solvent (2.0 mL, MeCN/H_2O 4:1) under air atmosphere at room temperature and allowed to react for 1 h.

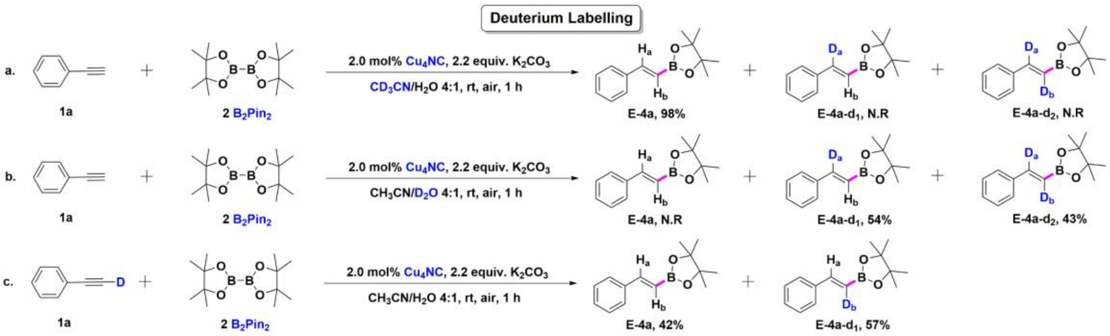

Supplementary Figure 42. Deuterium experiments of microcrystalline Cu4NC-catalyzed hydroboration reaction. a. The CD_3CN as deuterium source in deuterium experiment. **b.** The D_2O as deuterium source in deuterium experiment. **c.** The phenylacetylene-d1 as deuterium source in deuterium experiment.

General Procedure for deuterium experiment a. Under air atmosphere, phenylacetylene **1a** (20.4 mg, 0.2 mmol, 1.0 eq), B_2Pin_2 **2** (111.8 mg, 0.44 mmol, 2.2 equiv.), microcrystalline Cu4NC catalysts (2.8 mg, 2.0 mol%), K_2CO_3 (60.7 mg, 0.44 mmol, 2.2 equiv.) and the mixture solvent (2.0 mL, CD_3CN-H_2O, v/v 4/1) were added

into a tube. The reaction mixture was stirred at room temperature for 1 h. The reactions were monitored by TLC. When alkynes were consumed, the reactions were quenched and concentrated. Conversion and yield were determined by ^{1}H NMR using 1,3,5-trimethoxybenzene as an internal standard. The crude products were then purified by column chromatography (PE-EA, v/v 20/1) to give the target product **E-4a** as a colorless oil. **^{1}H NMR** (600 MHz, $CDCl_3$) δ 7.49 (d, *J* = 7.2 Hz, 2H), 7.40 (d, *J* = 18.4 Hz, 1H), 7.34 (t, *J* = 7.4 Hz, 2H), 7.29 (ddd, *J* = 7.3, 3.6, 1.2 Hz, 1H), 6.17 (d, *J* = 18.4 Hz, 1H), 1.32 (s, 12H).

General Procedure for deuterium experiment b. Under air atmosphere, phenylacetylene 1a (20.4 mg, 0.2 mmol, 1.0 eq), B_2Pin_2 2 (111.8 mg, 0.44 mmol, 2.2 equiv.), microcrystalline Cu_4NC catalysts (2.8 mg, 2.0 mol%), K_2CO_3 (60.7 mg, 0.44 mmol, 2.2 equiv.) and the mixture solvent (2.0 mL, CH_3CN-D_2O, v/v 4/1) were added into a tube. The reaction mixture was stirred at room temperature for 1 h. The reactions were monitored by TLC. When alkynes were consumed, the reactions were quenched and concentrated. Conversion and yield were determined by ^{1}H NMR using 1,3,5-trimethoxybenzene as an internal standard. The crude products were then purified by column chromatography to give the target products **E-4a-d₁** and **E-4a-d₂** as colorless oils, which are inseparable mixture of products with an isolated ratio of **E-4a-d₁** to **E-4a-d₂** of 58:42, compared to the original ratio of 54:43. ^{1}H NMR (600 MHz, $CDCl_3$) δ 7.51 – 7.48 (m, 2H), 7.36 – 7.32 (m, 2H), 7.31 – 7.28 (m, 1H), 6.18 – 6.16 (m, 0.58H), 1.32 (s, 12H). ^{2}H NMR (92 MHz, $CDCl_3$) δ 7.40 (s, 1H), 6.17 (s, 0.42H).

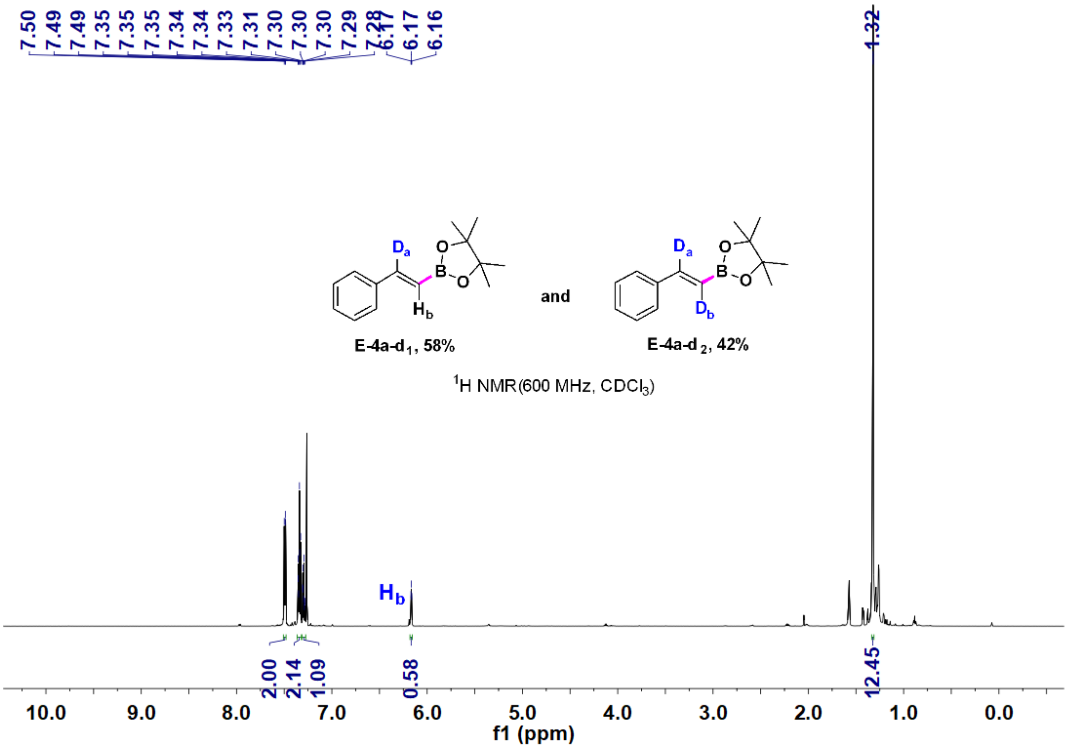

Supplementary Figure 43. ^{1}H NMR spectrum of inseparable compounds E-4a-d₁ and E-4a-d₂ in $CDCl_3$. The control experiment was catalyzed by microcrystalline Cu_4NC in the mixture solvent of CH_3CN-D_2O.

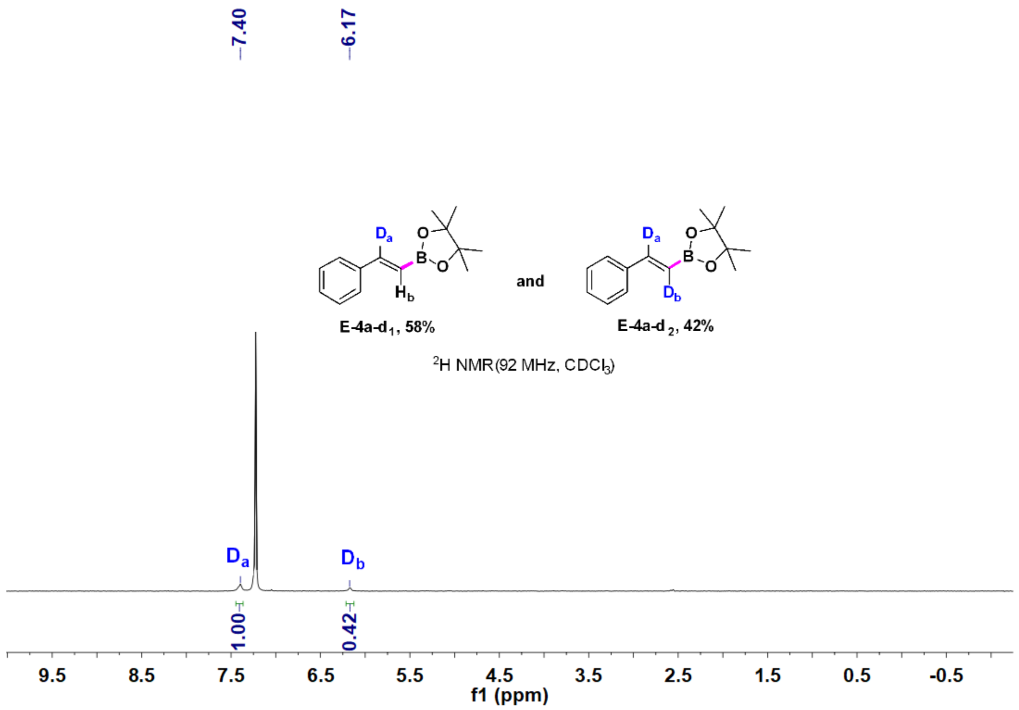

Supplementary Figure 44. ^{2}H NMR spectrum of inseparable compounds E-4a-d$_1$ and E-4a-d$_2$ in $CDCl_3$-DCM. The control experiment was catalyzed by microcrystalline Cu_4NC in the mixture solvent of CH_3CN-D_2O.

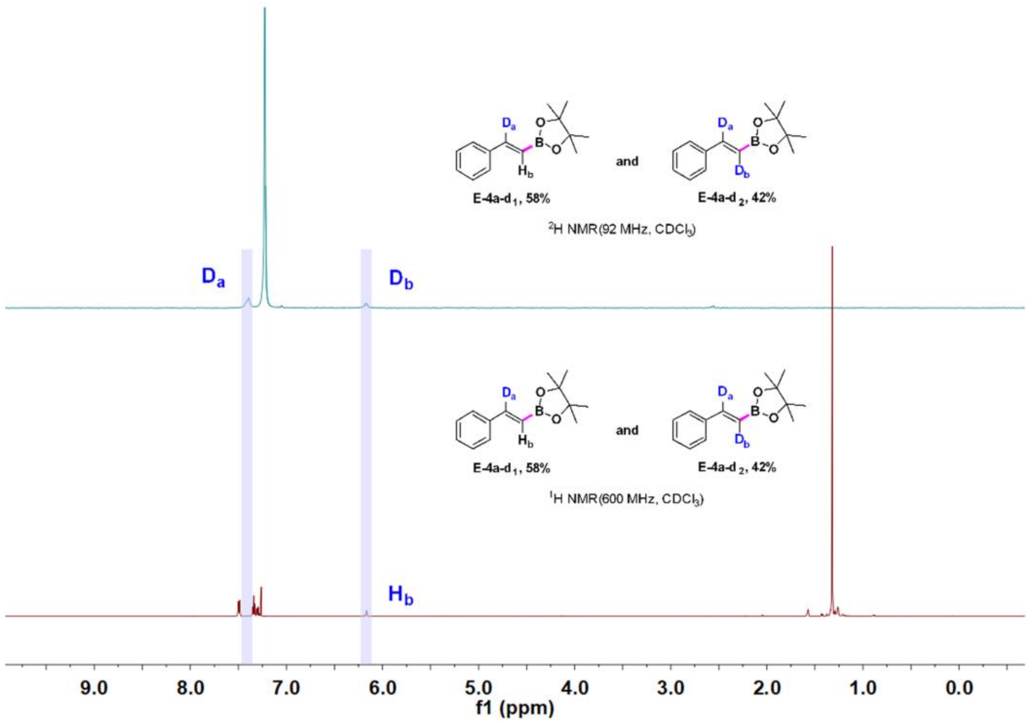

Supplementary Figure 45. ^{2}H NMR spectrum of inseparable compounds E-4a-d$_1$ and E-4a-d$_2$ in $CDCl_3$-DCM (top) and ^{1}H NMR spectrum of inseparable compounds E-4a-d$_1$ and E-4a-d$_2$ in $CDCl_3$ (bottom). The control experiment was catalyzed by microcrystalline Cu_4NC in the mixture solvent of CH_3CN-D_2O.

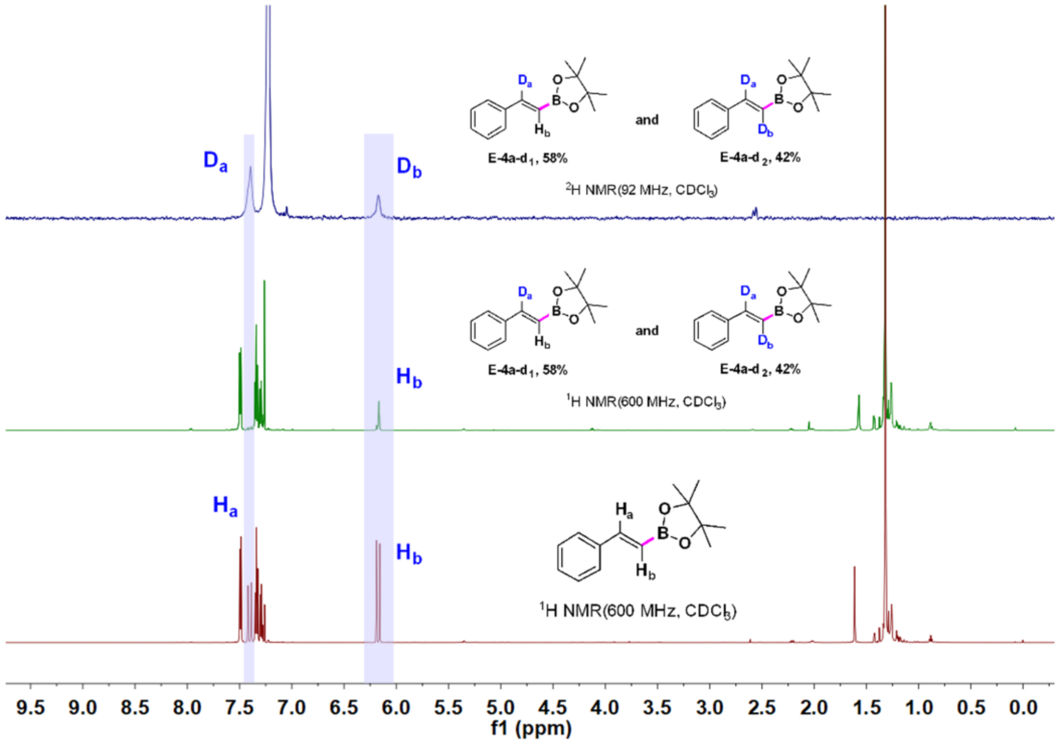

Supplementary Figure 46. ^{1}H NMR spectra and ^{2}H NMR spectra of compounds E-4a, E-4a-d$_1$ and E-4a-d$_2$. ^{2}H NMR spectrum of inseparable compounds E-4a-d$_1$ and E-4a-d$_2$ (top) in $CDCl_3$-DCM, ^{1}H NMR spectra of inseparable compounds E-4a-d$_1$ and E-4a-d$_2$ (middle) and compound E-4a (bottom) in $CDCl_3$. The control experiment was catalyzed by microcrystalline Cu_4NC in the mixture solvent of CH_3CN-D_2O.

General Procedure for deuterium experiment c. Under air atmosphere, phenylacetylene-d$_1$ **1a** (20.6 mg, 0.2 mmol, 1.0 eq), B_2Pin_2 **2** (111.8 mg, 0.44 mmol, 2.2 equiv.), microcrystalline Cu_4NC catalysts (2.8 mg, 2.0 mol%), K_2CO_3 (60.7 mg, 0.44 mmol, 2.2 equiv.) and the mixture solvent (2.0 mL, CH_3CN-H_2O, v/v 4/1) were added into a tube. The reaction mixture was stirred at room temperature for 1 h. The reactions were monitored by TLC. When alkynes were consumed, the reactions were quenched and concentrated. Conversion and yield were determined by ^{1}H NMR using 1,3,5-trimethoxybenzene as an internal standard. The crude products were then purified by column chromatography to give the target products **E-4a** and **E-4a-d$_1$** as colorless oils, which are inseparable mixture of products with an isolated ratio of **E-4a** to **E-4a-d$_1$** of 35:65, compared to the original ratio of 42:57. ^{1}H NMR (600 MHz, $CDCl_3$) δ 7.49 (d, J = 7.9 Hz, 2H), 7.41 (d, J = 14.2 Hz, 1H), 7.34 (t, J = 7.4 Hz, 2H), 7.29 (t, J = 7.2 Hz, 1H), 6.17 (d, J = 18.4 Hz, 0.35H), 1.32 (s, 12H). ^{2}H NMR (92 MHz, $CDCl_3$) δ 6.17 (d, J = 2.2 Hz, 0.65H).

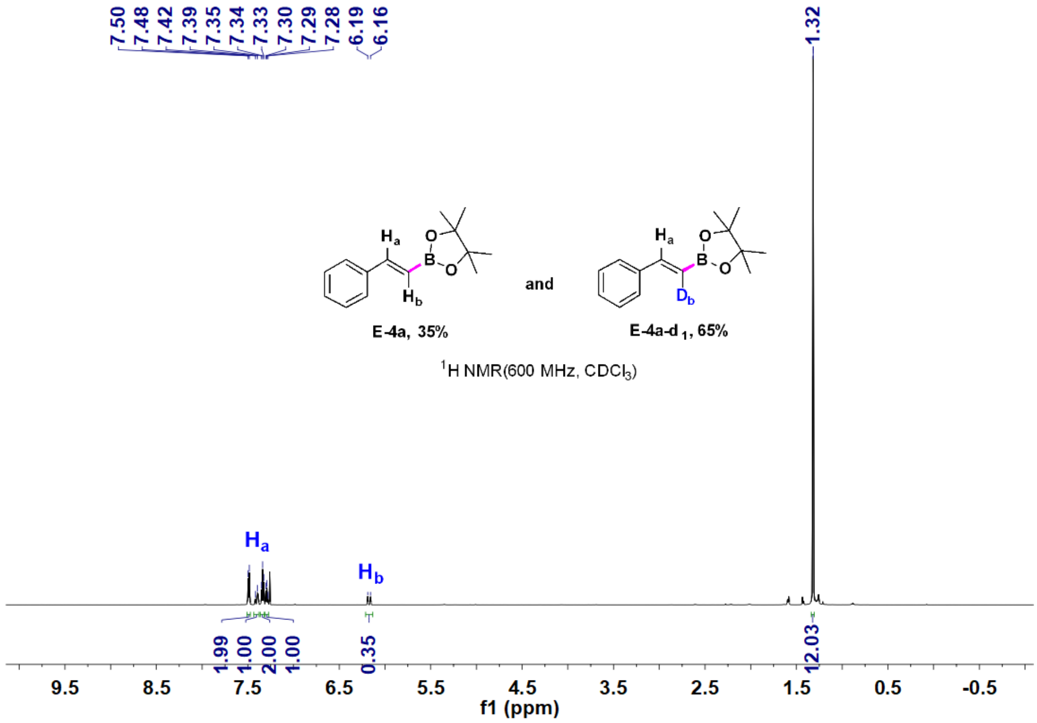

Supplementary Figure 47. ^{1}H NMR spectrum of inseparable compounds E-4a and E-4a-d$_1$ in CDCl$_3$. The phenylacetylene-d$_1$ was used in control experiment catalyzed by microcrystalline Cu$_4$NC.

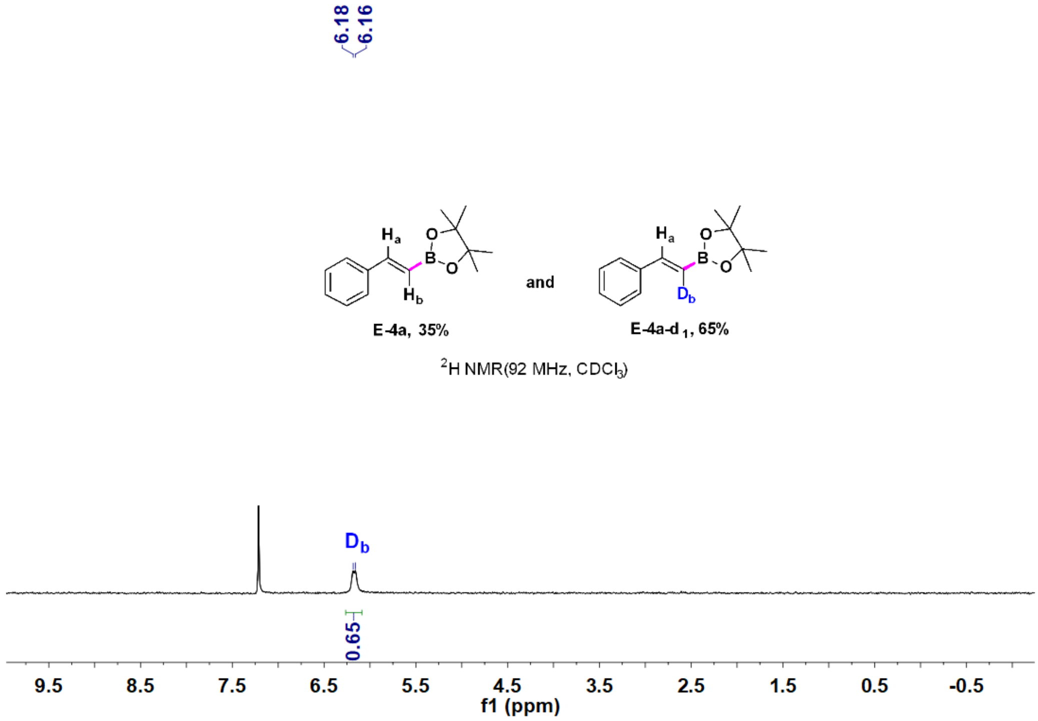

Supplementary Figure 48. ^{2}H NMR spectrum of inseparable compounds E-4a and E-4a-d$_1$ in CDCl$_3$-DCM. The phenylacetylene-d$_1$ was used in control experiment catalyzed by microcrystalline Cu$_4$NC.

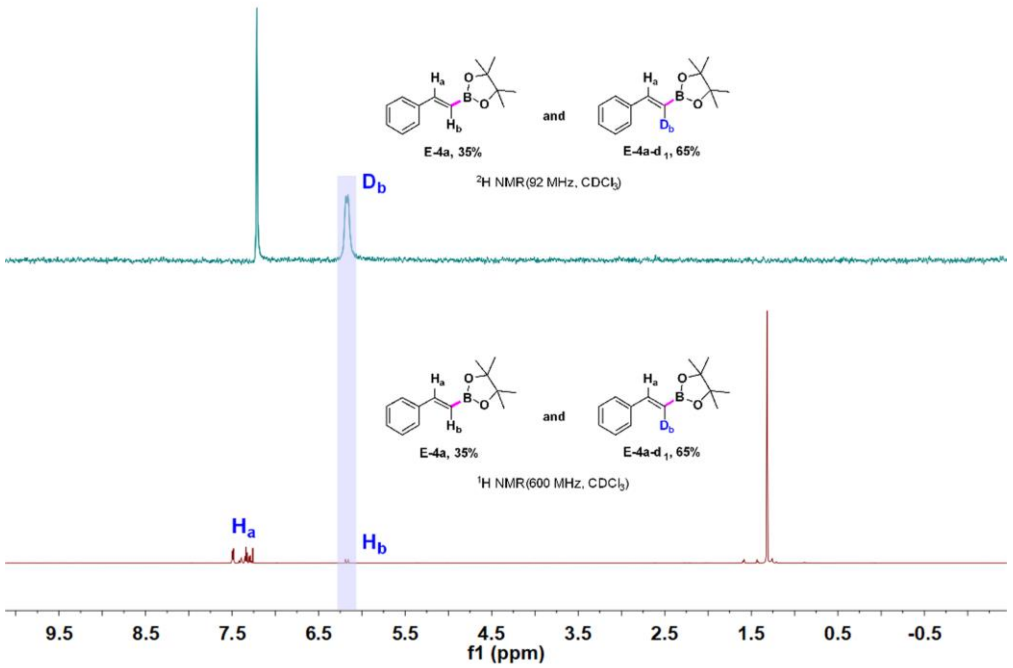

Supplementary Figure 49. ^{2}H NMR spectrum of inseparable compounds E-4a and E-4a-d$_1$ in $CDCl_3$-DCM (top) and ^{1}H NMR spectrum of inseparable compounds E-4a and E-4a-d$_1$ in $CDCl_3$ (bottom). The phenylacetylene-d$_1$ was used in control experiment catalyzed by microcrystalline Cu_4NC.

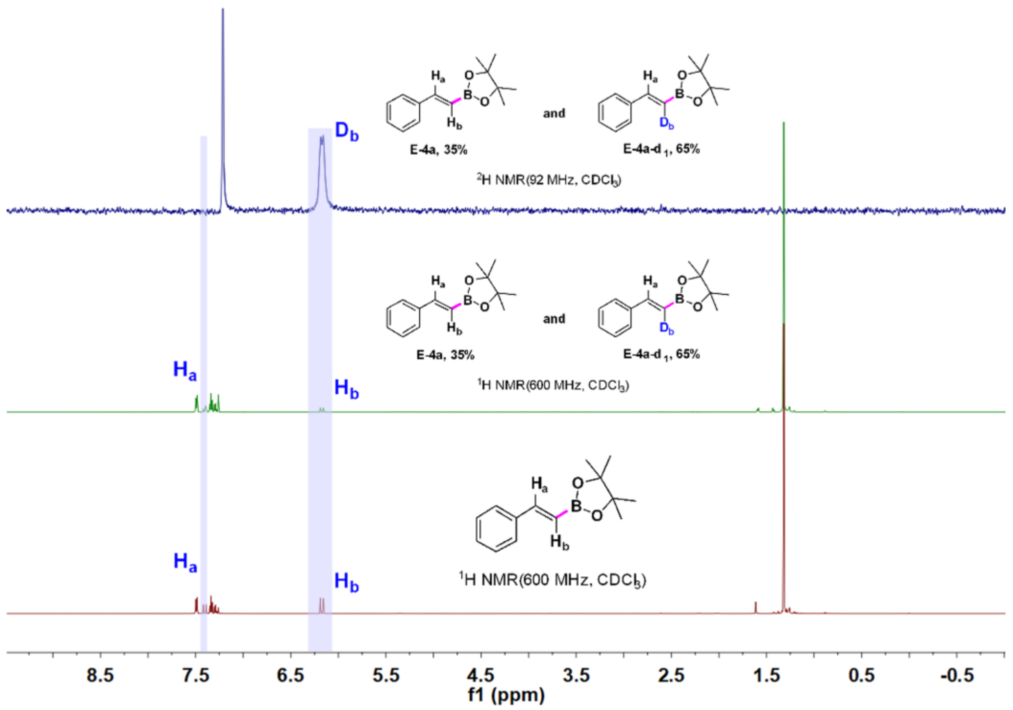

Supplementary Figure 50. ^{1}H NMR spectra and ^{2}H NMR spectra of compounds E-4a and E-4a-d$_1$. ^{2}H NMR spectrum of inseparable compounds E-4a and E-4a-d$_1$ (top) in $CDCl_3$-DCM, ^{1}H NMR spectra of inseparable compounds E-4a and E-4a-d$_1$ (middle) and compound E-4a (bottom) in $CDCl_3$. The phenylacetylene-d$_1$ was used in control experiment catalyzed by microcrystalline Cu_4NC.

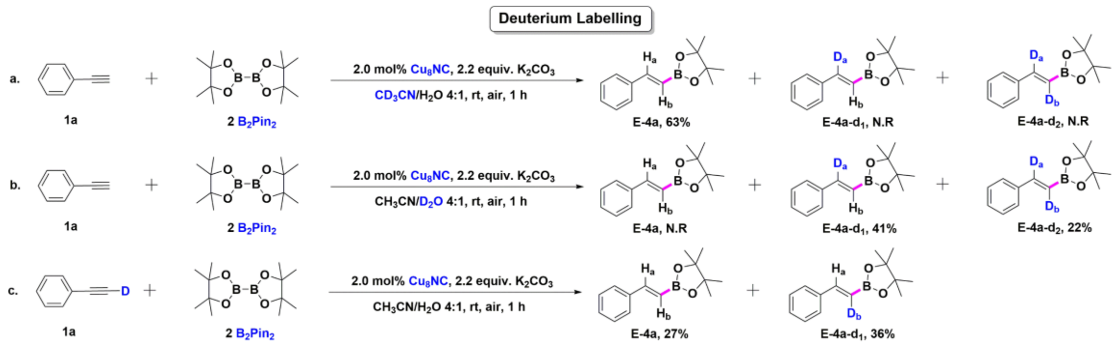

Supplementary Figure 51. Deuterium experiments of microcrystalline Cu_8NC-catalyzed hydroboration reaction. a. The CD_3CN as deuterium source in deuterium experiment. **b.** The D_2O as deuterium source in deuterium experiment. **c.** The phenylacetylene-d_1 as deuterium source in deuterium experiment.

General Procedure for deuterium experiment a. Under air atmosphere, phenylacetylene **1a** (20.4 mg, 0.2 mmol, 1.0 eq), B_2Pin_2 **2** (111.8 mg, 0.44 mmol, 2.2 equiv.), microcrystalline Cu_8NC catalysts (5.3 mg, 2.0 mol%), K_2CO_3 (60.7 mg, 0.44 mmol, 2.2 equiv.) and the mixture solvent (2.0 mL, CD_3CN-H_2O, v/v 4/1) were added into a tube. The reaction mixture was stirred at room temperature for 1 h. The reactions were monitored by TLC. When alkynes were consumed, the reactions were quenched and concentrated. Conversion and yield were determined by ^{1}H NMR using 1,3,5-trimethoxybenzene as an internal standard. The crude products were then purified by column chromatography to give the target product **E-4a** as a colorless oil. **^{1}H NMR** (600 MHz, $CDCl_3$) δ 7.49 (d, J = 7.2 Hz, 2H), 7.40 (d, J = 18.4 Hz, 1H), 7.34 (t, J = 7.4 Hz, 2H), 7.29 (ddd, J = 7.3, 3.6, 1.2 Hz, 1H), 6.17 (d, J = 18.4 Hz, 1H), 1.32 (s, 12H).

General Procedure for deuterium experiment b. Under air atmosphere, phenylacetylene **1a** (20.4 mg, 0.2 mmol, 1.0 eq), B_2Pin_2 **2** (111.8 mg, 0.44 mmol, 2.2 equiv.), microcrystalline Cu_8NC catalysts (5.3 mg, 2.0 mol%), K_2CO_3 (60.7 mg, 0.44 mmol, 2.2 equiv.) and the mixture solvent (2.0 mL, CH_3CN-D_2O, v/v 4/1) were added into a tube. The reaction mixture was stirred at room temperature for 1 h. The reactions were monitored by TLC. When alkynes were consumed, the reactions were quenched and concentrated. Conversion and yield were determined by ^{1}H NMR using 1,3,5-trimethoxybenzene as an internal standard. The crude products were then purified by column chromatography to give the target products **E-4a-d$_1$** and **E-4a-d$_2$** as colorless oils, which are inseparable mixture of products with an isolated ratio of **E-4a-d$_1$** to **E-4a-d$_2$** of 67:33, compared to the original ratio of 41:22. ^{1}H NMR (600 MHz, $CDCl_3$) δ 7.50 (dd, J = 8.2, 1.0 Hz, 2H), 7.34 (dd, J = 10.1, 4.6 Hz, 2H), 7.32 – 7.27 (m, 1H), 6.18 – 6.16 (m, 0.67H), 1.32 (s, 12H). ^{2}H NMR (92 MHz, $CDCl_3$) δ 7.39 (s, 1H), 6.17 (s, 0.33H).

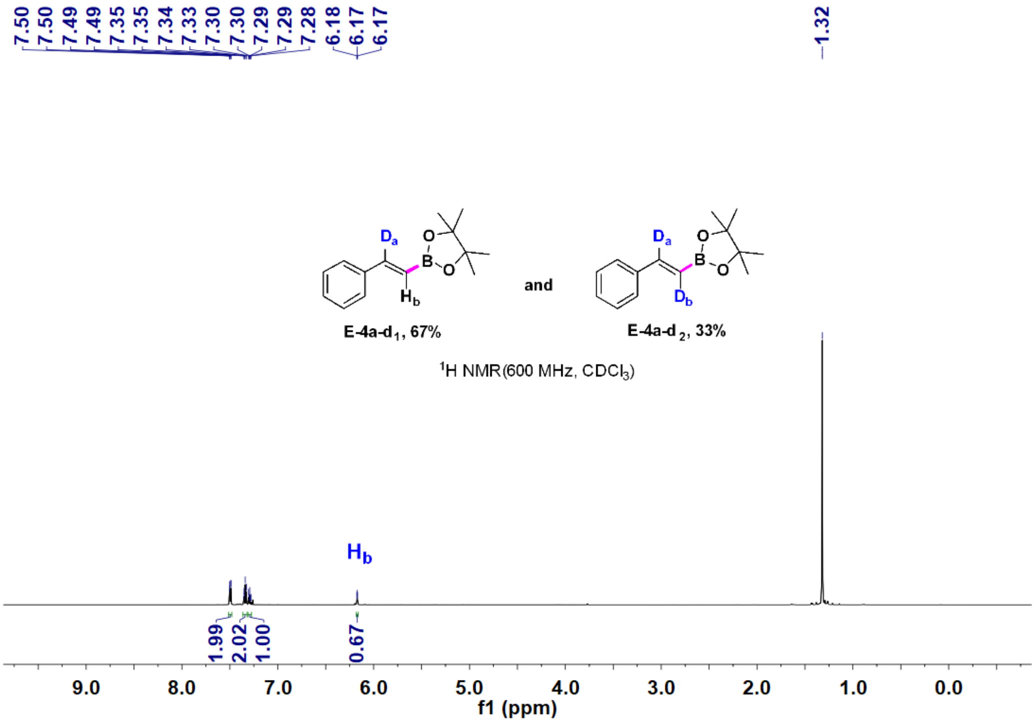

Supplementary Figure 52. ^{1}H NMR spectrum of inseparable compounds E-4a-d$_1$ and E-4a-d$_2$ in $CDCl_3$. The control experiment was catalyzed by microcrystalline Cu_8NC in the mixture solvent of CH_3CN-D_2O.

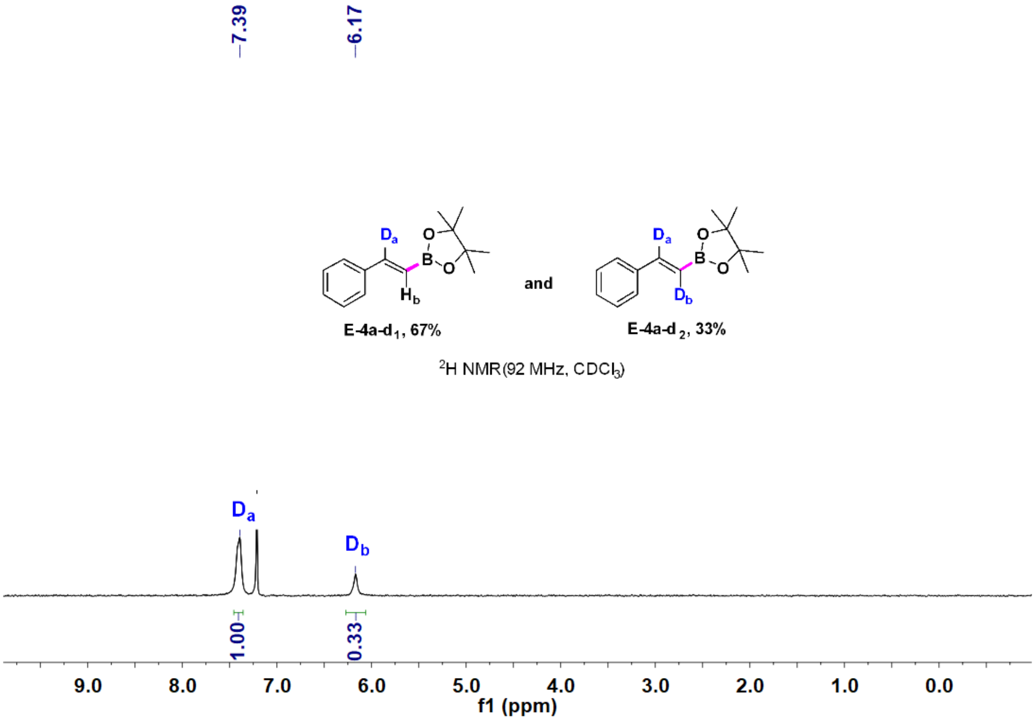

Supplementary Figure 53. ^{2}H NMR spectrum of inseparable compounds E-4a-d$_1$ and E-4a-d$_2$ in $CDCl_3$-DCM. The control experiment was catalyzed by microcrystalline Cu_8NC in the mixture solvent of CH_3CN-D_2O.

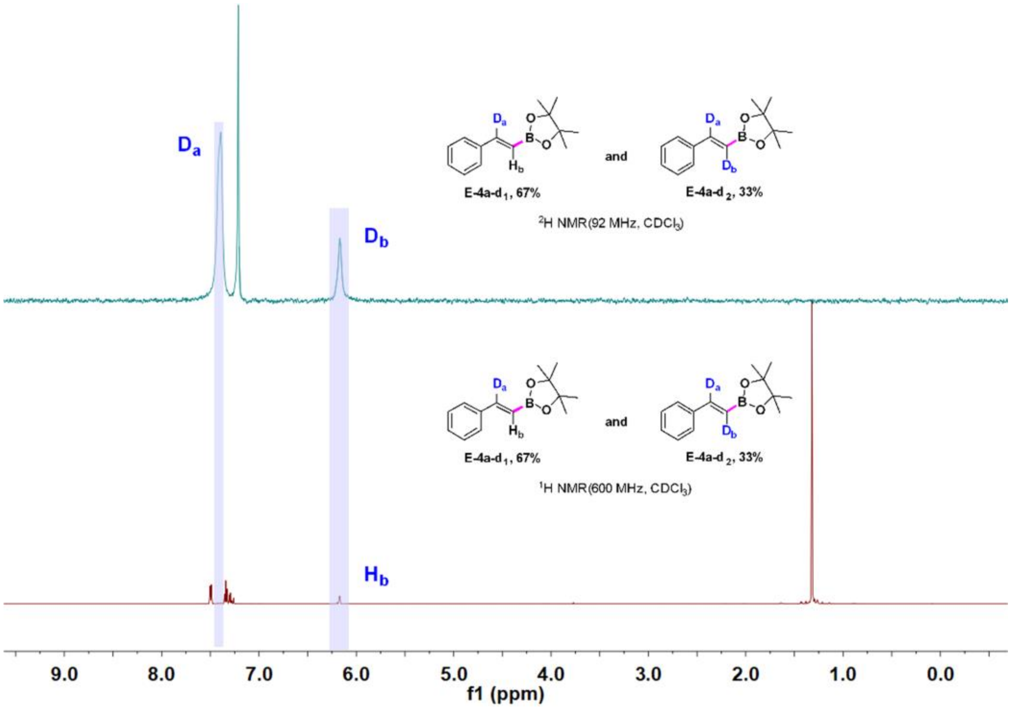

Supplementary Figure 54. ^{2}H NMR spectrum of inseparable compounds E-4a-d_1 and E-4a-d_2 in $CDCl_3$-DCM (top) and ^{1}H NMR spectrum of inseparable compounds E-4a-d_1 and E-4a-d_2 in $CDCl_3$ (bottom). The control experiment was catalyzed by microcrystalline Cu_8NC in the mixture solvent of CH_3CN-D_2O.

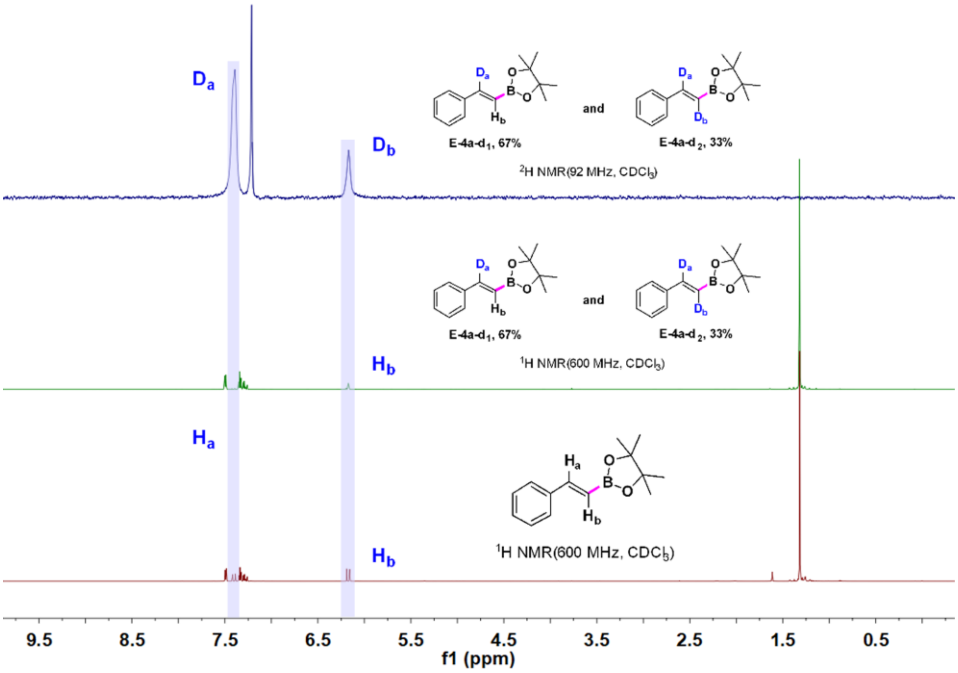

Supplementary Figure 55. ^{1}H NMR spectra and ^{2}H NMR spectra of compounds E-4a, E-4a-d_1 and E-4a-d_2. ^{2}H NMR spectrum of inseparable compounds E-4a-d_1 and E-4a-d_2 (top) in $CDCl_3$-DCM, ^{1}H NMR spectra of inseparable compounds E-4a-d_1 and E-4a-d_2 (middle) and compound E-4a (bottom) in $CDCl_3$. The control experiment was catalyzed by microcrystalline Cu_8NC in the mixture solvent of CH_3CN-D_2O.

General Procedure for deuterium experiment c. Under air atmosphere, phenylacetylene-d_1 **1a** (20.6 mg, 0.2 mmol, 1.0 eq), B_2Pin_2 **2** (111.8 mg, 0.44 mmol, 2.2 equiv.), microcrystalline Cu_8NC catalysts (5.3 mg, 2.0 mol%), K_2CO_3 (60.7 mg, 0.44 mmol, 2.2 equiv.) and the mixture solvent (2.0 mL, CH_3CN-H_2O, v/v 4/1) were added into a tube. The reaction mixture was stirred at room temperature for 1 h. The reactions were monitored by TLC. When alkynes were consumed, the reactions were quenched and concentrated. Conversion and yield were determined by ^1H NMR using 1,3,5-trimethoxybenzene as an internal standard. The crude products were then purified by column chromatography to give the target products **E-4a** and **E-4a-d₁** as colorless oils, which are inseparable mixture of products with an isolated ratio of **E-4a** to **E-4a-d₁** of 28:72, compared to the original ratio of 27:36. ^1H NMR (600 MHz, $CDCl_3$) δ 7.50 (d, J = 7.4 Hz, 2H), 7.44 – 7.38 (m, 1H), 7.34 (t, J = 7.4 Hz, 2H), 7.29 (t, J = 7.2 Hz, 1H), 6.19 (dd, J = 18.4, 5.0 Hz, 0.28H), 1.32 (s, 12H). ^2H NMR (92 MHz, $CDCl_3$) δ 6.18 (s, 0.72H).

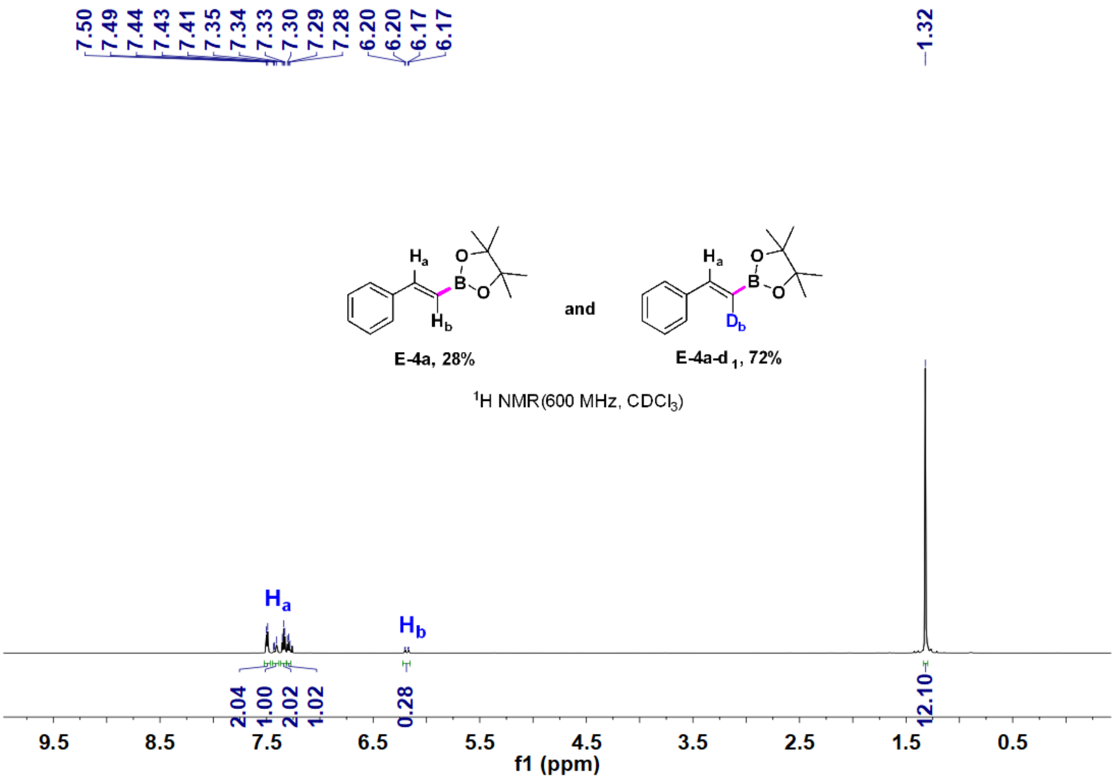

Supplementary Figure 56. ^1H NMR spectrum of inseparable compounds E-4a and E-4a-d_1 in $CDCl_3$. The phenylacetylene-d_1 was used in control experiment catalyzed by microcrystalline Cu_8NC.

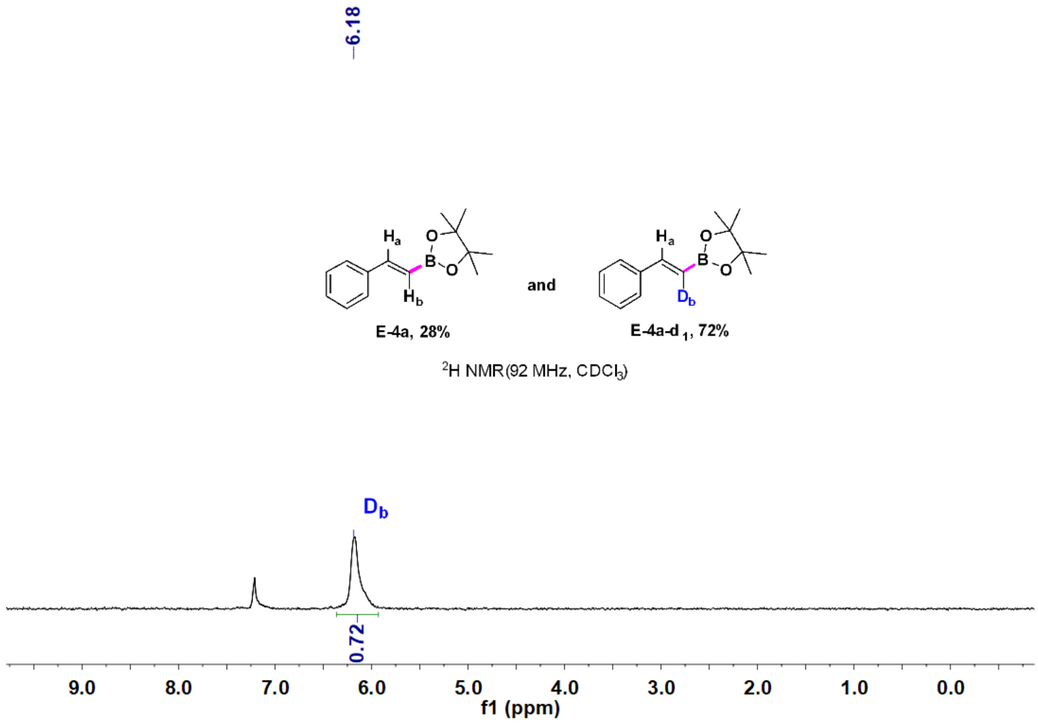

Supplementary Figure 57. ^{2}H NMR spectrum of inseparable compounds E-4a and E-4a-d$_1$ in $CDCl_3$-DCM. The phenylacetylene-d$_1$ was used in control experiment catalyzed by microcrystalline Cu_8NC.

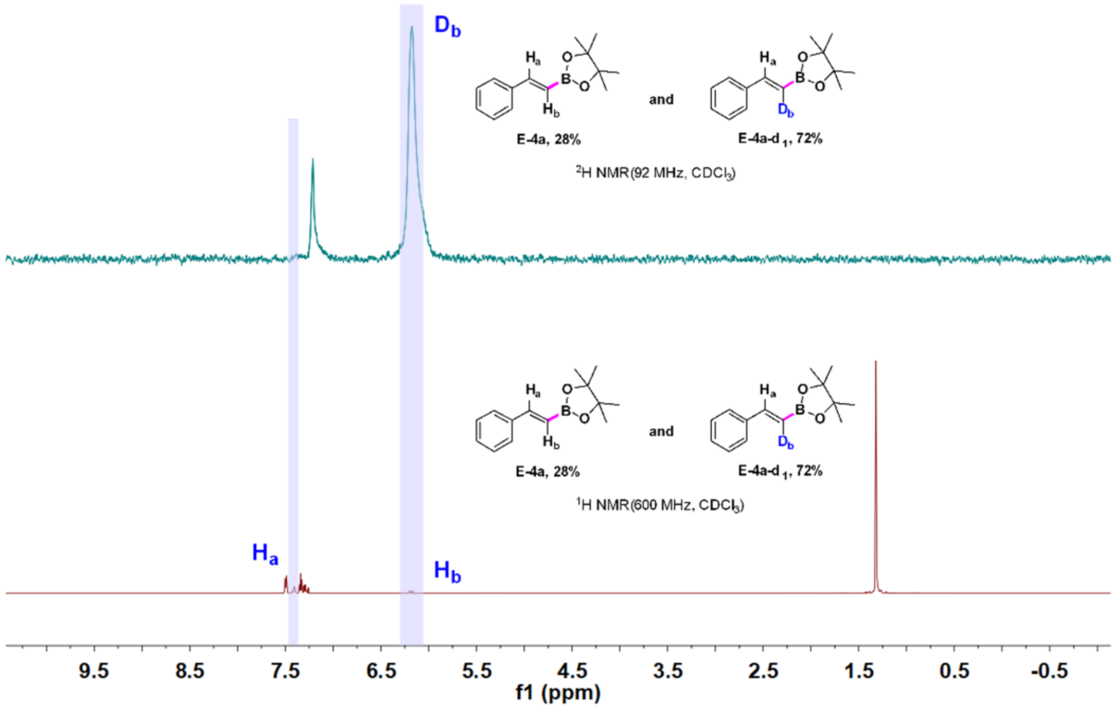

Supplementary Figure 58. ^{2}H NMR spectrum of inseparable compounds E-4a and E-4a-d$_1$ in $CDCl_3$-DCM (top) and ^{1}H NMR spectrum of inseparable compounds E-4a and E-4a-d$_1$ in $CDCl_3$ (bottom). The phenylacetylene-d$_1$ was used in control experiment catalyzed by microcrystalline Cu_8NC.

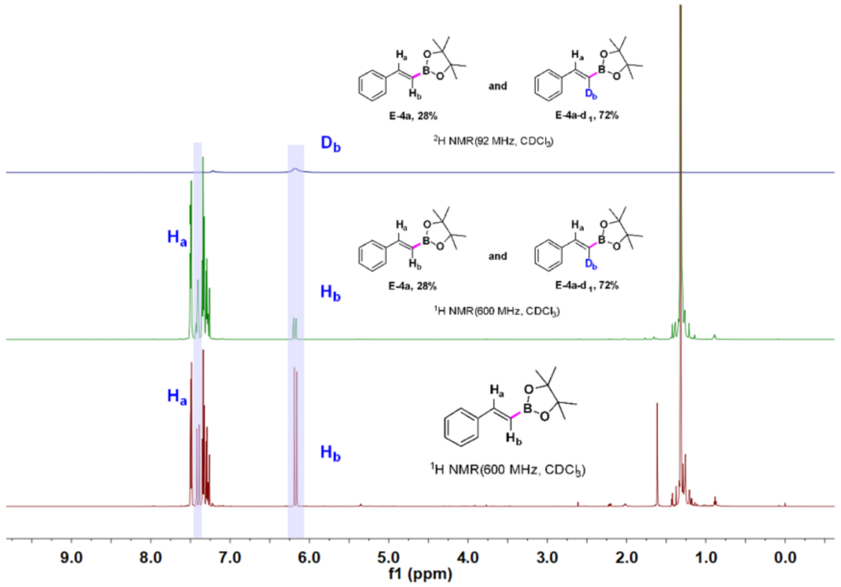

Supplementary Figure 59. ^{1}H NMR spectra and ^{2}H NMR spectra of compounds E-4a and E-4a-d$_1$. ^{2}H NMR spectrum of inseparable compounds E-4a and E-4a-d$_1$ (top) in $CDCl_3$-DCM, ^{1}H NMR spectra of inseparable compounds E-4a and E-4a-d$_1$ (middle) and compound E-4a (bottom) in $CDCl_3$. The phenylacetylene-d$_1$ was used in control experiment catalyzed by microcrystalline Cu_8NC.

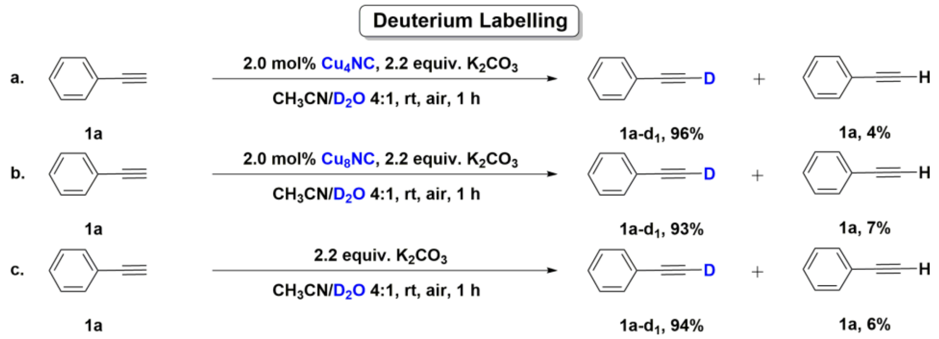

Supplementary Figure 60. Deuterium experiments of the deuteration method of phenylacetylene. a. The Cu_4NC as catalyst in deuterium experiment. **b.** The Cu_8NC as catalyst in deuterium experiment. **c.** No catalyst in deuterium experiment.

General Procedure for deuterium experiment a. Under air atmosphere, phenylacetylene **1a** (20.4 mg, 0.2 mmol, 1.0 eq), microcrystalline Cu_4NC catalysts (2.8 mg, 2.0 mol%), K_2CO_3 (60.7 mg, 0.44 mmol, 2.2 equiv.) and the mixture solvent (2.0 mL, CH_3CN-D_2O, v/v 4/1) were added into a tube. The reaction mixture was stirred at room temperature for 1 h. The reactions were monitored by TLC. Conversion and yield were determined by ^{1}H NMR using 1,3,5-trimethoxybenzene as an internal standard. The reaction solution was filtrated through celite and concentrated to give the target products **1a** and **1a-d$_1$** as a colorless oil, which are inseparable mixture of products with a ratio of **1a** to **1a-d$_1$** of 96:4. ^{1}H NMR (600 MHz, $CDCl_3$) δ 7.53 (ddd, J = 2.2, 1.8, 0.9 Hz, 2H), 7.40 – 7.31 (m, 3H), 3.10 (s, 0.4H). ^{2}H NMR (92 MHz, $CDCl_3$) δ 3.10 (s, 0.96H).

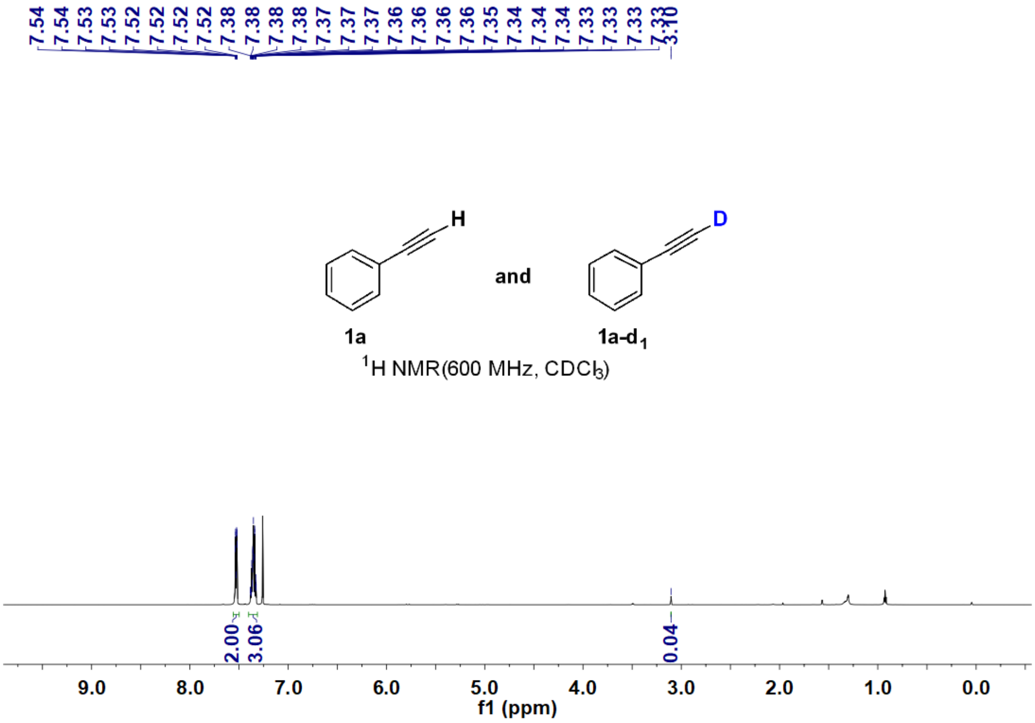

Supplementary Figure 61. ^{1}H NMR spectrum of inseparable compounds 1a and 1a-d$_1$ in CDCl$_3$. The phenylacetylene was deuterated in control experiment catalyzed by microcrystalline Cu$_4$NC.

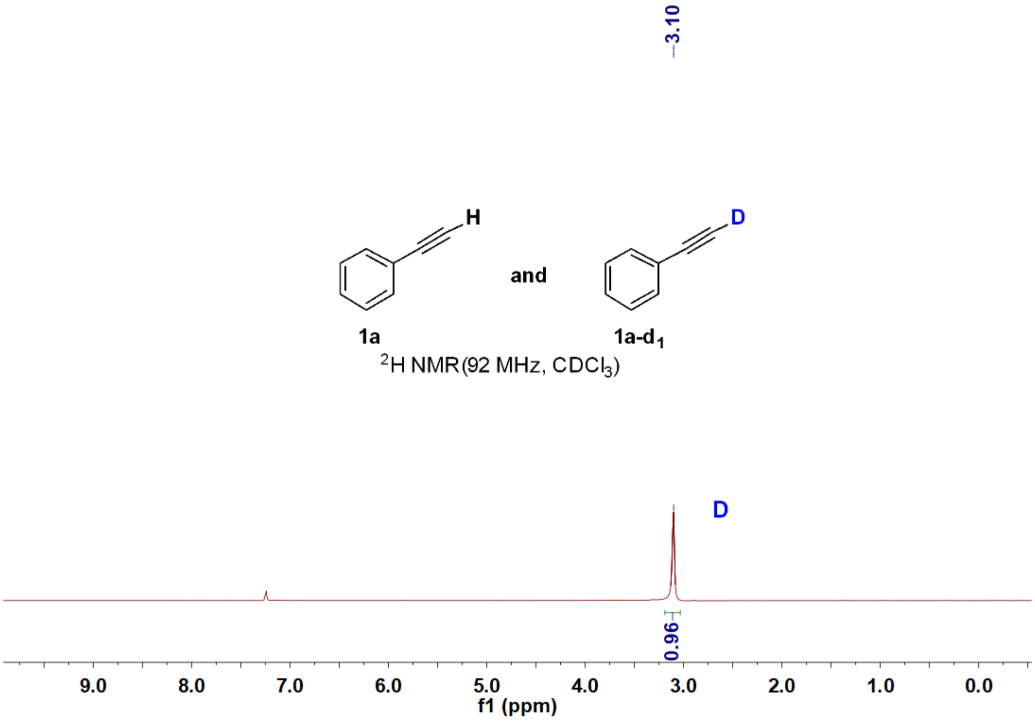

Supplementary Figure 62. ^{2}H NMR spectrum of inseparable compounds 1a and 1a-d$_1$ in CDCl$_3$-DCM. The phenylacetylene was deuterated in control experiment catalyzed by microcrystalline Cu$_4$NC.

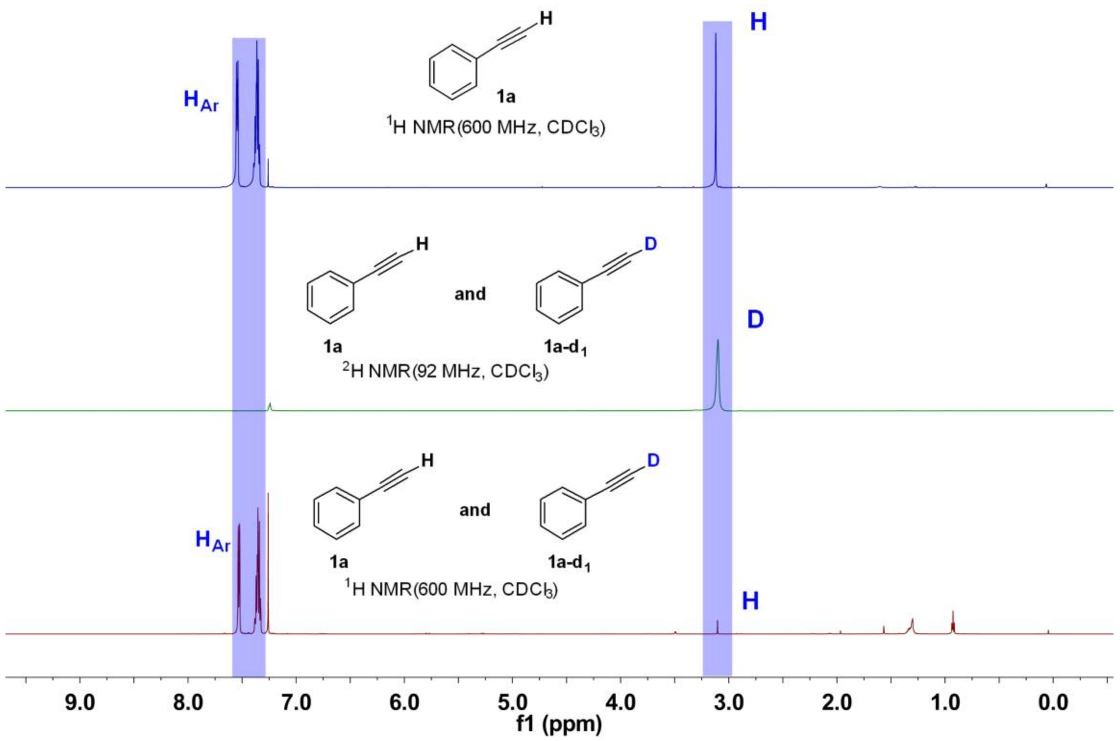

Supplementary Figure 63. ^{1}H NMR spectra and ^{2}H NMR spectra of compounds 1a and 1a-d$_1$. ^{1}H NMR spectrum of compounds 1a (top), ^{2}H NMR spectrum of inseparable compounds 1a and 1a-d$_1$ in $CDCl_3$-DCM (middle) and ^{1}H NMR spectrum of inseparable compounds 1a and 1a-d$_1$ in $CDCl_3$ (bottom). The phenylacetylene was deuterated in control experiment catalyzed by microcrystalline Cu_4NC.

General Procedure for deuterium experiment b. Under air atmosphere, phenylacetylene **1a** (20.4 mg, 0.2 mmol, 1.0 eq), microcrystalline Cu_8NC catalysts (5.3 mg, 2.0 mol%), K_2CO_3 (60.7 mg, 0.44 mmol, 2.2 equiv.) and the mixture solvent (2.0 mL, CH_3CN-D_2O, v/v 4/1) were added into a tube. The reaction mixture was stirred at room temperature for 1 h. The reactions were monitored by TLC. Conversion and yield were determined by ^{1}H NMR using 1,3,5-trimethoxybenzene as an internal standard. The reaction solution was filtrated through celite and concentrated to give the target products **1a** and **1a-d$_1$** as colorless oils, which are inseparable mixture of products with a ratio of **1a** to **1a-d$_1$** of 93:7. ^{1}H NMR (600 MHz, $CDCl_3$) δ 7.52 (dd, *J* = 6.6, 1.5 Hz, 2H), 7.40 – 7.31 (m, 3H), 3.10 (s, 0.07H). ^{2}H NMR (92 MHz, $CDCl_3$) δ 3.10 (s, 0.93H).

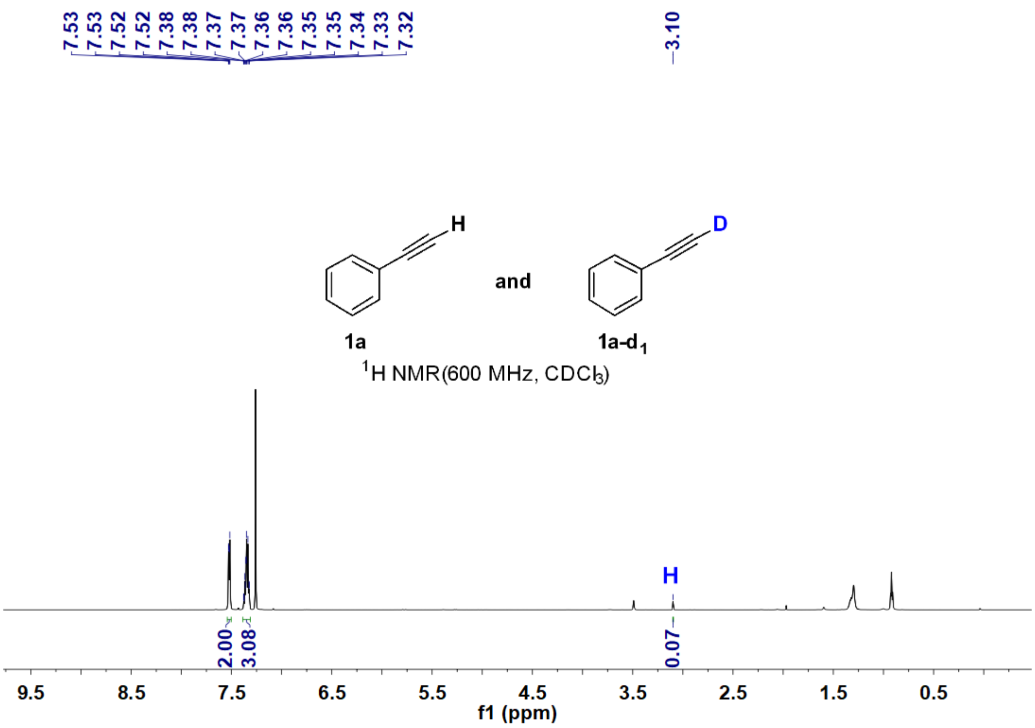

Supplementary Figure 64. ^{1}H NMR spectrum of inseparable compounds 1a and 1a-d$_1$ in $CDCl_3$. The phenylacetylene was deuterated in control experiment catalyzed by microcrystalline Cu_8NC.

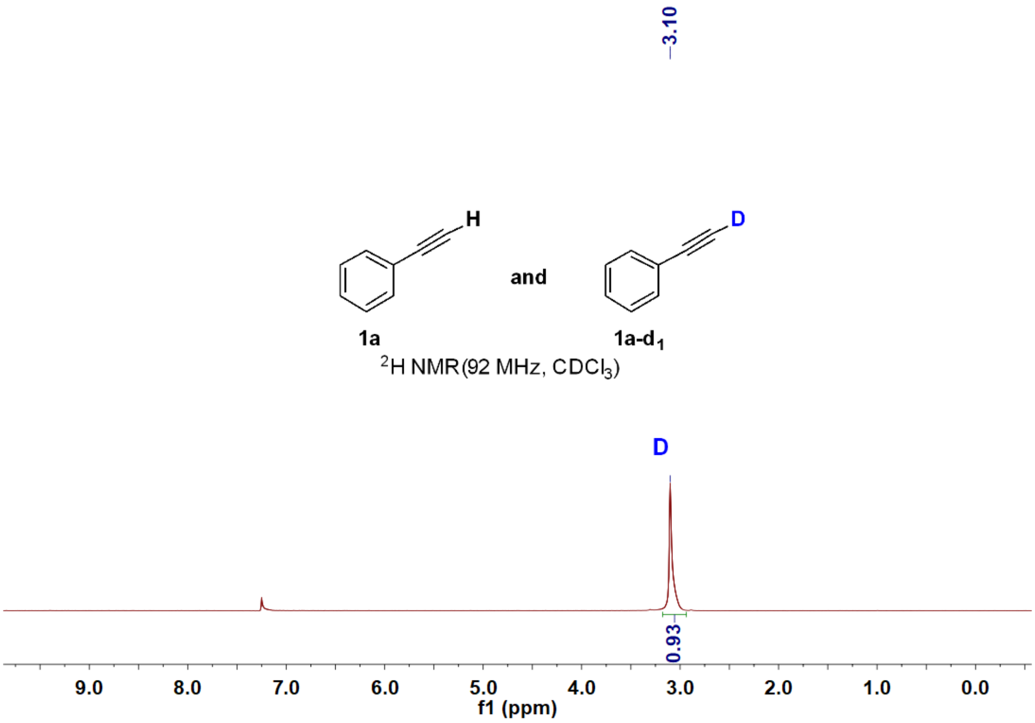

Supplementary Figure 65. ^{2}H NMR spectrum of inseparable compounds 1a and 1a-d$_1$ in $CDCl_3$-DCM. The phenylacetylene was deuterated in control experiment catalyzed by microcrystalline Cu_8NC.

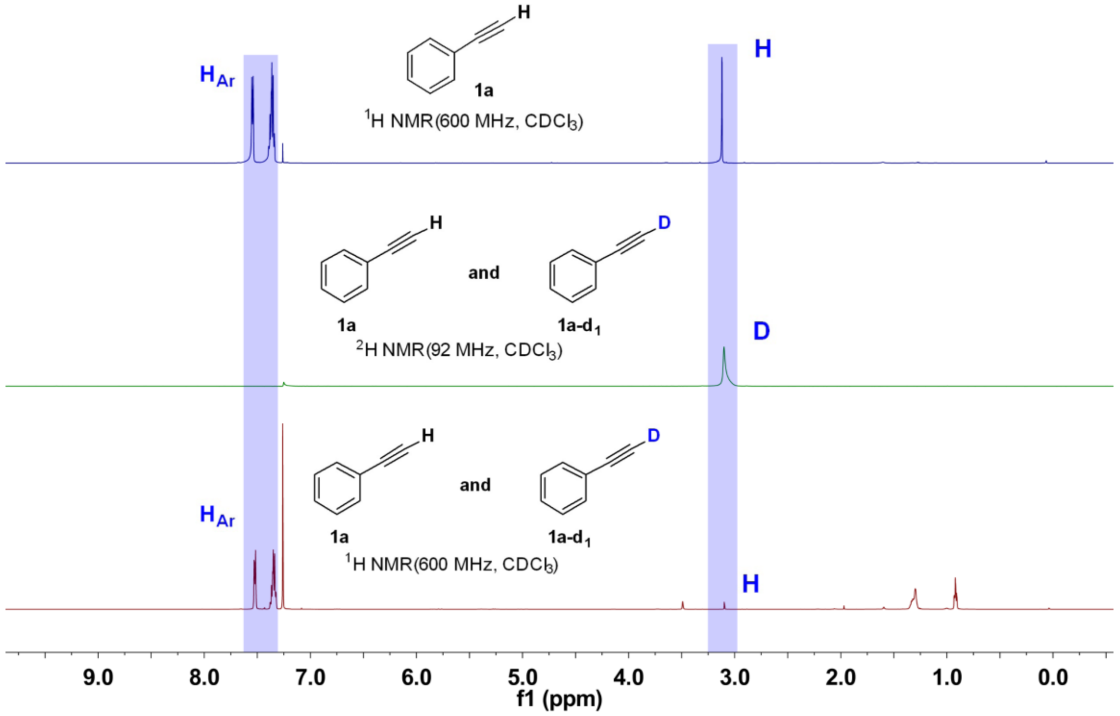

Supplementary Figure 66. ^{1}H NMR spectra and ^{2}H NMR spectra of compounds 1a and 1a-d$_1$. ^{1}H NMR spectrum of compound 1a (top), ^{2}H NMR spectrum of inseparable compounds 1a and 1a-d$_1$ in $CDCl_3$-DCM (middle), ^{1}H NMR spectrum of inseparable compounds 1a and 1a-d$_1$ in $CDCl_3$ (bottom). The phenylacetylene was deuterated in control experiment catalyzed by microcrystalline Cu_8NC.

General Procedure for deuterium experiment c. Under air atmosphere, phenylacetylene **1a** (20.4 mg, 0.2 mmol, 1.0 eq), K_2CO_3 (60.7 mg, 0.44 mmol, 2.2 equiv.) and the mixture solvent (2.0 mL, CH_3CN-D_2O, v/v 4/1) were added into a tube. The reaction mixture was stirred at room temperature for 1 h. The reactions were monitored by TLC. Conversion and yield were determined by ^{1}H NMR using 1,3,5-trimethoxybenzene as an internal standard. The reaction solution was filtrated through celite and concentrated to give the target products **1a** and **1a-d$_1$** as colorless oils, which are inseparable mixture of products with a ratio of **1a** to **1a-d$_1$** of 94:6. ^{1}H NMR (600 MHz, $CDCl_3$) δ 7.56 – 7.50 (m, 2H), 7.42 – 7.31 (m, 3H), 3.10 (s, 0.06H). ^{2}H NMR (92 MHz, $CDCl_3$) δ 3.10 (s, 0.94H).

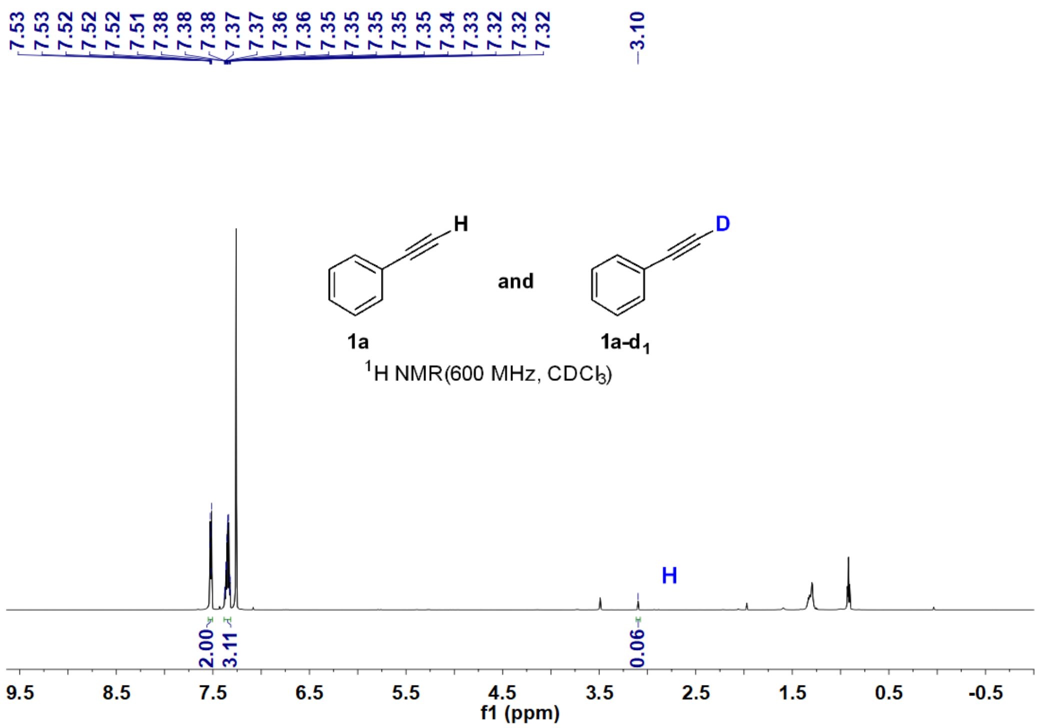

Supplementary Figure 67. ^{1}H NMR spectrum of inseparable compounds 1a and 1a-d$_1$ in $CDCl_3$. The phenylacetylene was deuterated in control experiment without copper catalyst.

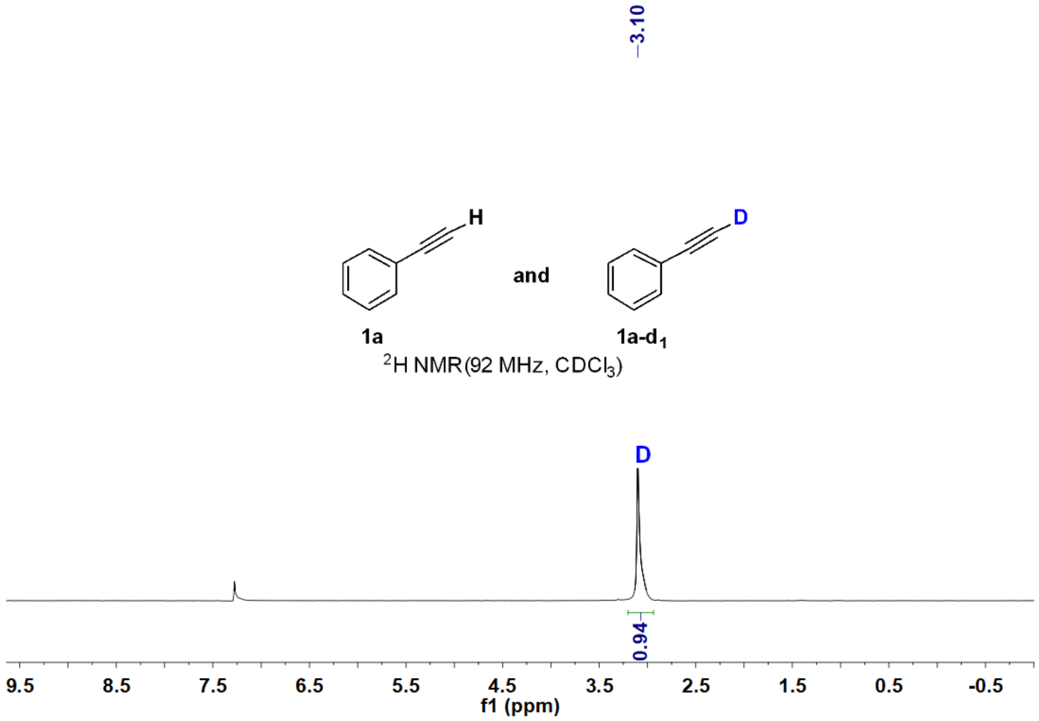

Supplementary Figure 68. ^{2}H NMR spectrum of inseparable compounds 1a and 1a-d$_1$ in $CDCl_3$-DCM. The phenylacetylene was deuterated in control experiment without copper catalyst.

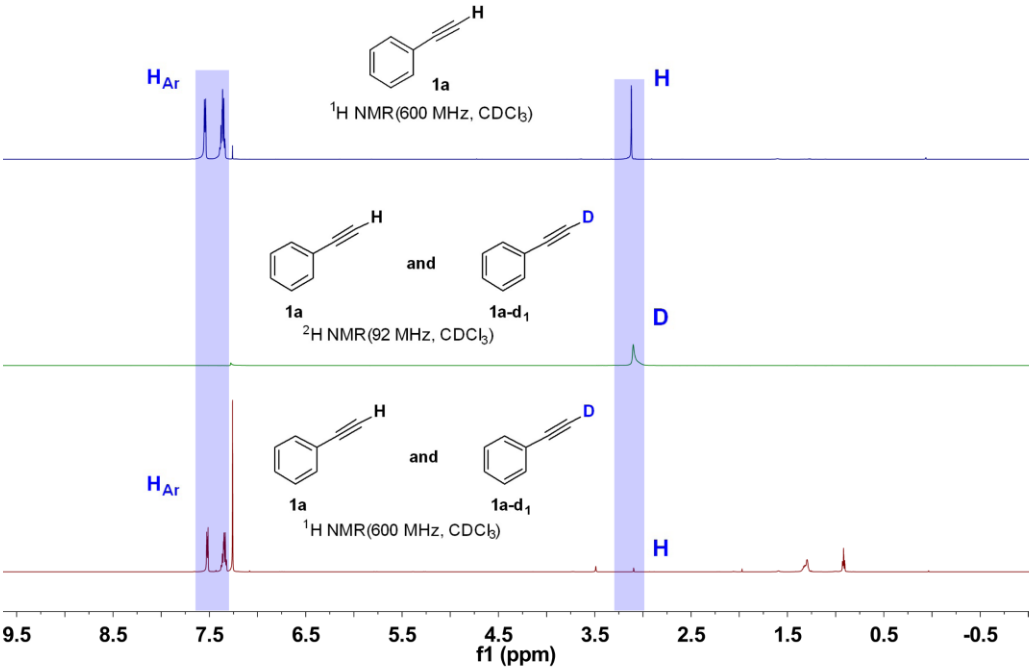

Supplementary Figure 69. ^{1}H NMR spectra and ^{2}H NMR spectra of compounds 1a and 1a-d$_1$. ^{1}H NMR spectrum of compound 1a (top), ^{2}H NMR spectrum of inseparable compounds 1a and 1a-d$_1$ in $CDCl_3$-DCM (middle), ^{1}H NMR spectrum of inseparable compounds 1a and 1a-d$_1$ in $CDCl_3$ (bottom). The phenylacetylene was deuterated in control experiment without copper catalyst.

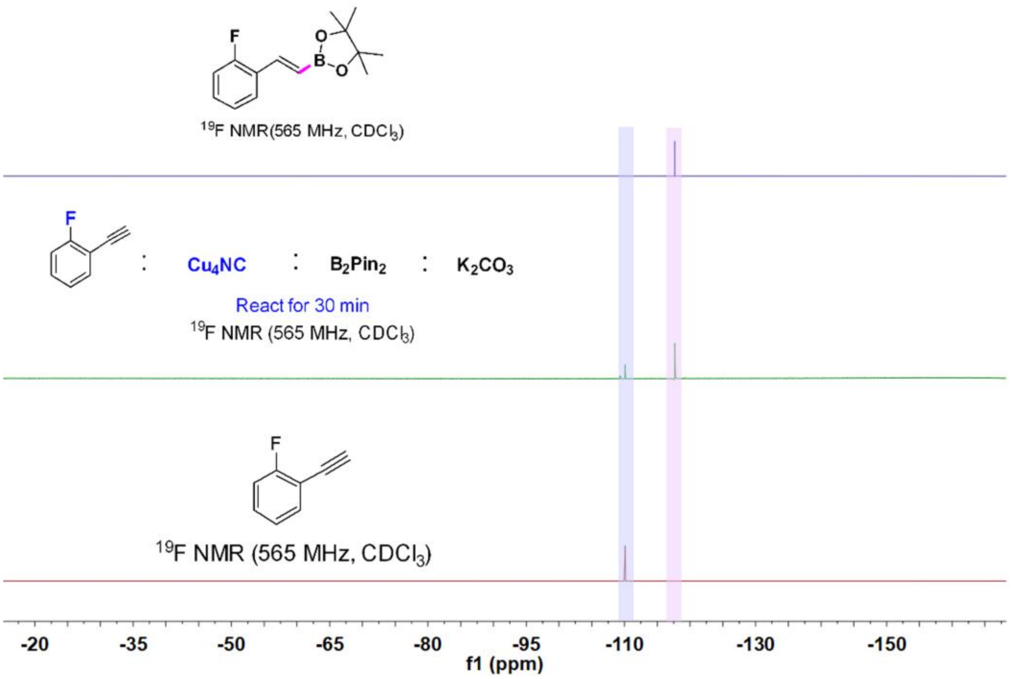

Supplementary Figure 70. ^{19}F NMR spectra tracking of hydroboration. ^{19}F NMR spectra of (E)-2-(2-fluorostyryl)-4,4,5,5-tetramethyl-1,3,2-dioxaborolane (E-4g) (top), the Cu$_4$NC catalyzed hydroboration for 30 min (middle) and 2-fluorophenylacetylene (bottom) in $CDCl_3$.

Catalytic Mechanism Studies by Density Functional Theory (DFT) Calculations

Density functional theory (DFT) calculations at the PBE0 level of theory were performed to understand the reaction mechanism(7,8). The def2svp basis set was employed for C, O, H, B, N, S, and Cu atoms(9-12). Solvation effects were modeled using the Solvation Model based on Density (SMD) with acenitrile as the solvent(13). Additional dispersion corrections were applied using the Grimme's D3 method(14,15). Vibrational frequency calculations were performed to ensure that a transition state has only one imaginary frequency and a local minimum has no imaginary frequency. Transition states connecting relevant minima were further examined by carrying out intrinsic reaction coordinate (IRC) calculations(16-18). All the calculations were performed using Gaussian 16 software(19).

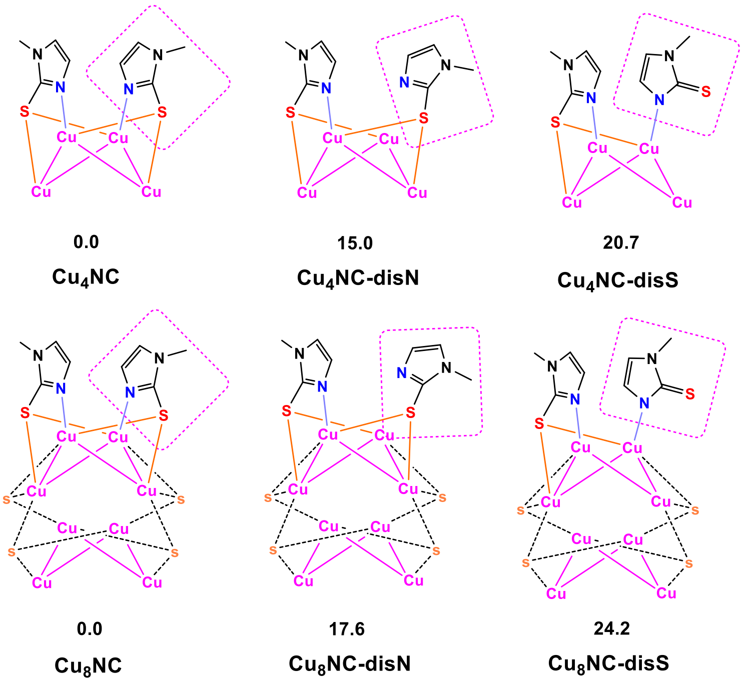

Supplementary Figure 71. DFT-calculated the binding energies of Cu-S bonds and Cu-N bonds. DFT-calculated the binding energies of Cu-S bonds and Cu-N bonds in Cu_4NC and Cu_8NC (ΔG in kcal/mol).

In our experiment, we used K_2CO_3 as the base. Since K_2CO_3 can react with water to form KOH, we simplified our calculations by using KOH as the base. In the Cu_4NC, KOH undergoes a ligand substitution reaction to form intermediate A via an association mechanism. As shown in Supplementary Figure 72, the energy barrier leading to intermediate A is only 2.9 kcal/mol and the energy of intermediate A is -19.3 kcal/mol, indicating that this process is both kinetically and thermodynamically favorable. This suggests that the formation of intermediate A from the Cu_4NC is feasible and that intermediate A serves as a suitable starting point for this reaction. Similarly, formation of Cu_8NC-OH intermediate B from Cu_8NC is also kinetically and thermodynamically favorable, suggesting intermediate B is a suitable starting point (Supplementary Figure 73).

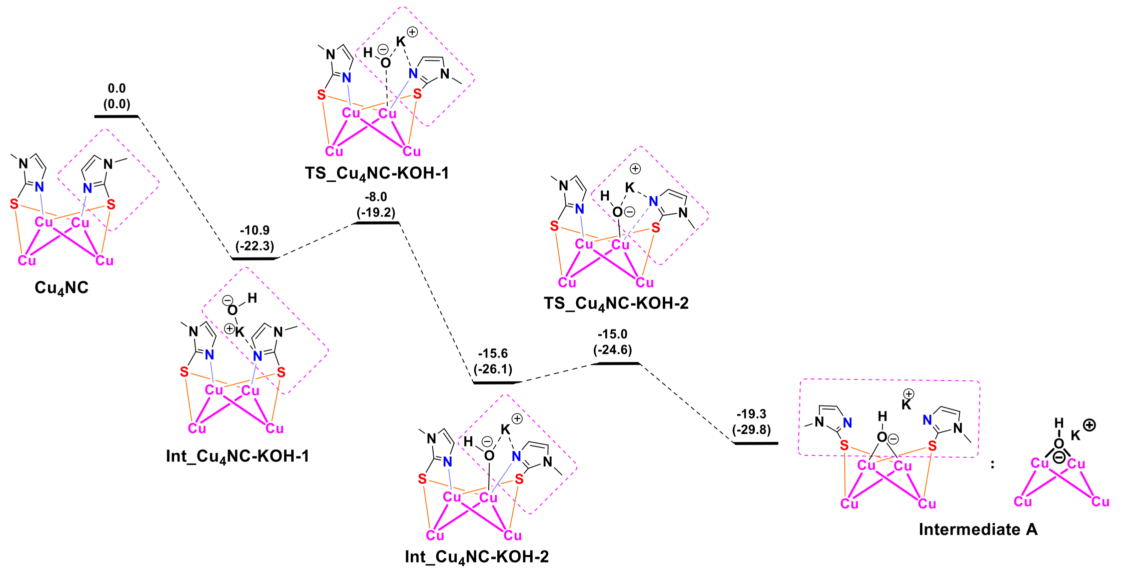

Supplementary Figure 72. DFT-computed Gibbs free energy profile for formation of Intermediate A. The relative Gibbs energies and electronic energies (in parentheses) are given in kcal/mol.

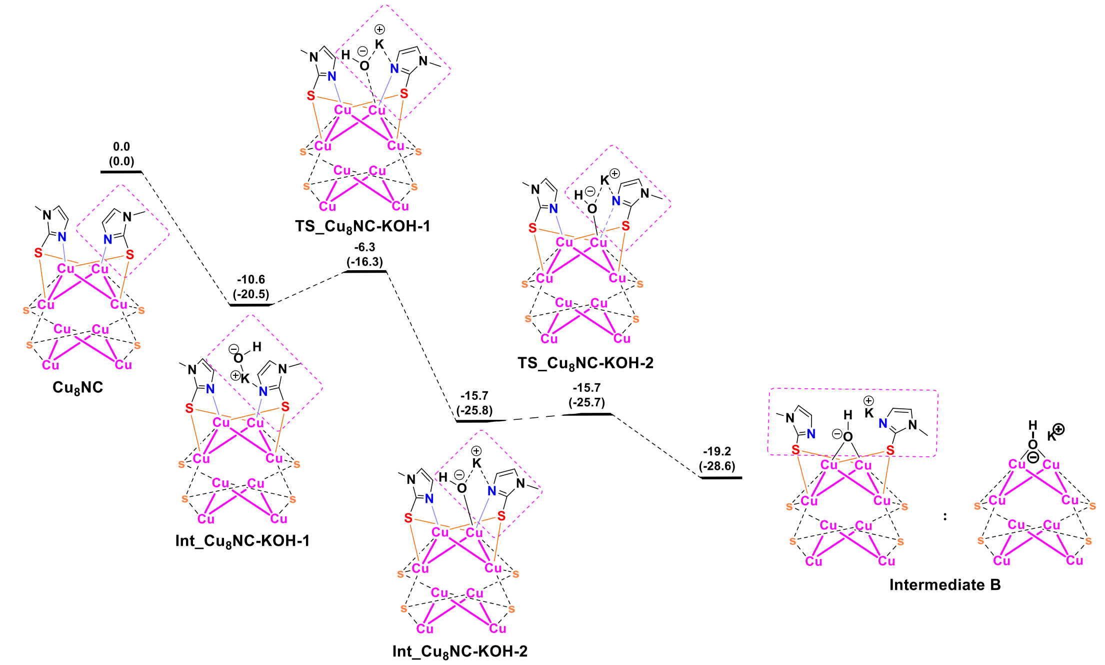

Supplementary Figure 73. DFT-computed Gibbs free energy profile for formation of Intermediate B. The relative Gibbs energies and electronic energies (in parentheses) are given in kcal/mol.

The schematic illustration of the energy profile of the microcrystalline Cu_4NC-catalyzed hydroboration reaction is depicted in Figure 5, Supplementary Figure 74 and Supplementary Figure 75. The $TS_{4\text{-}5}$ represent rate-determining transition state. The Int2 represent rate-determining intermediate. So the overall barrier could be calculated by $\Delta G_1 + \Delta G_2$. And we got $\Delta G_1 = 18.2$ kcal mol^{-1} and $\Delta G_2 = 4.7$ kcal mol^{-1} from the Figure 5 and Supplementary Figure 74.

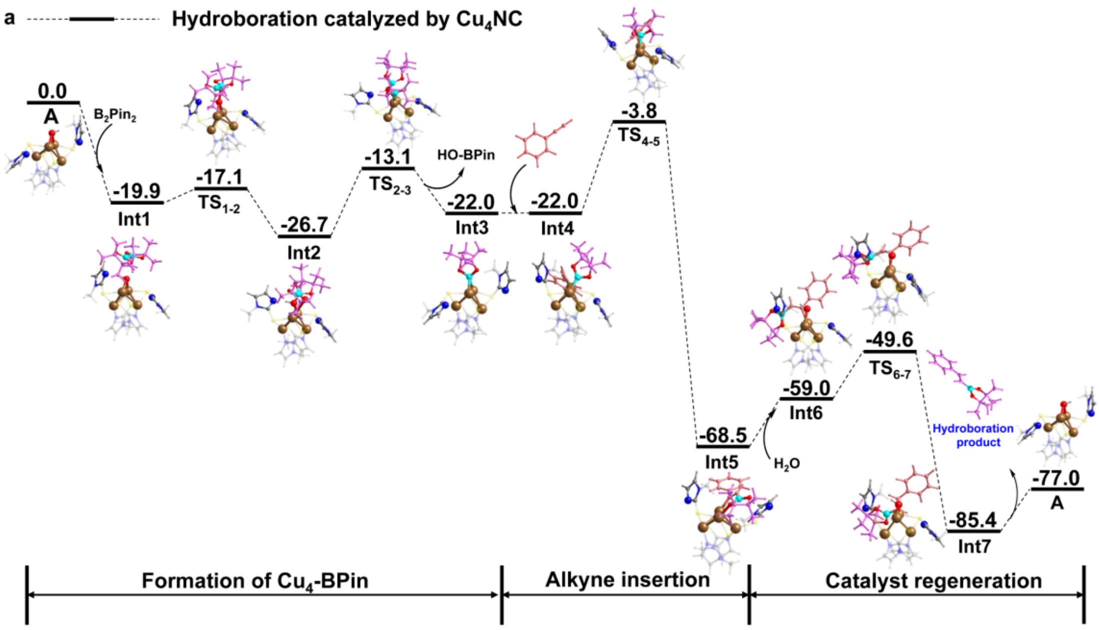

Supplementary Figure 74. DFT-computed Gibbs free energy profile. DFT-computed Gibbs free energy profile (ΔG in kcal/mol) for the hydroboration catalyzed by microcrystalline Cu_4NC.

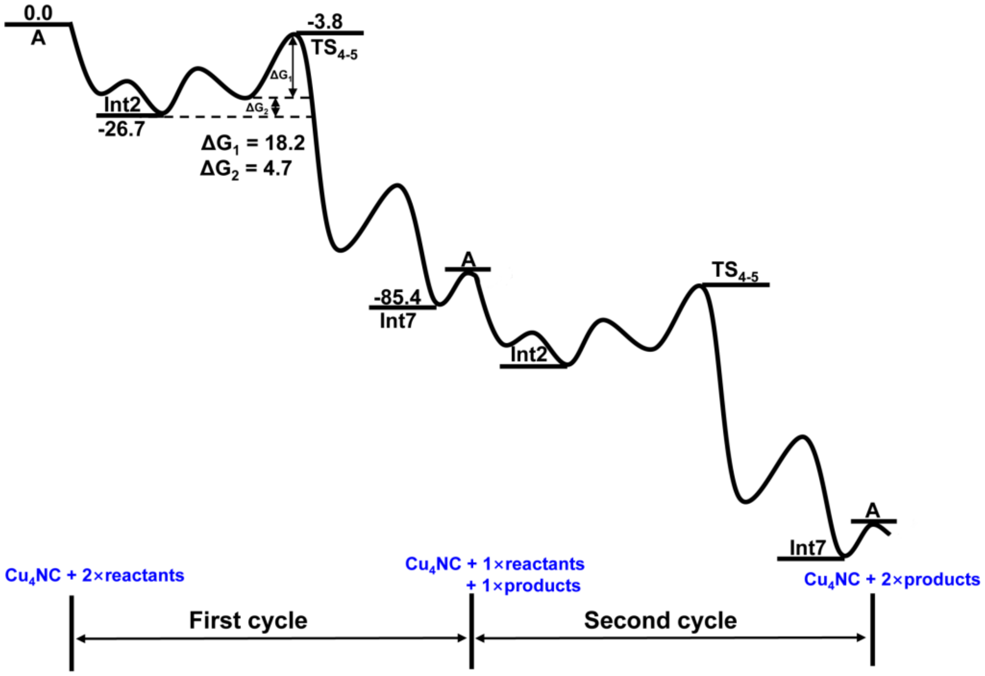

Supplementary Figure 75. Schematic illustration of the energy profile. Schematic illustration of the energy profile for an additional catalytic cycle (phenylacetylene as the alkyne substrate), which includes energy profile given the microcrystalline Cu_4NC catalyzed hydroboration reaction in Figure 5. The relative Gibbs energies (in black) are given in kcal/mol.

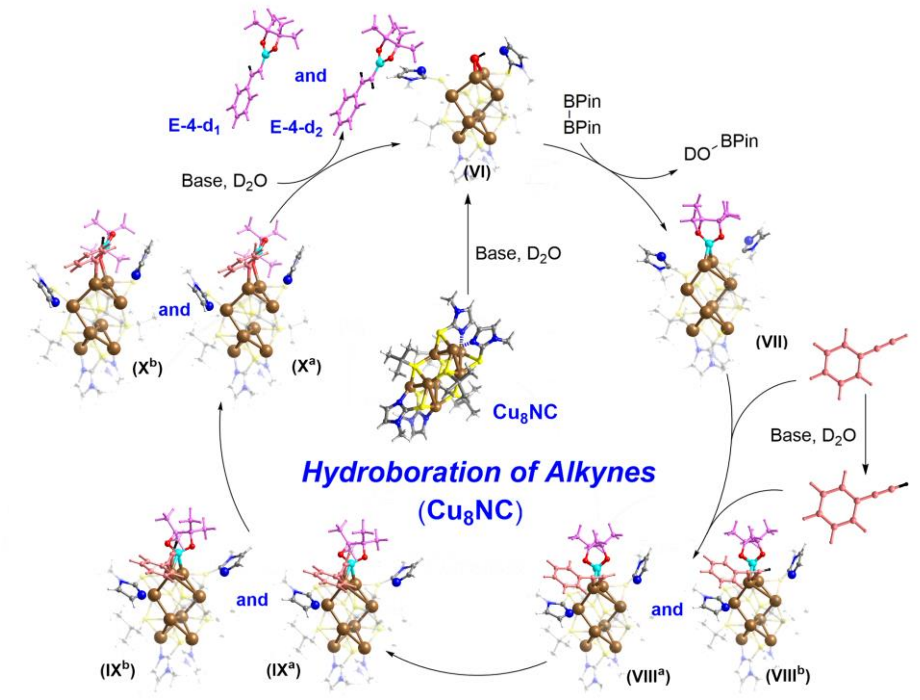

Supplementary Figure 76. A Proposed reaction mechanism. The catalytic mechanism for the formation of the vinylboronate esters catalyzed by microcrystalline Cu_8NC.

The catalytic mechanism for the formation of the vinylboronate esters catalyzed by DRDS Cu_8NC is depicted in Supplementary Figure 76. The schematic illustration of the energy profile of the microcrystalline Cu_8NC-catalyzed hydroboration reaction is depicted in Supplementary Figures 77–79. The $TS_{11\text{-}12}$ represent rate-determining transition state and the Int10 represent rate-determining intermediate. Therefore, the overall barrier could be calculated by ΔG_3. And we got ΔG_3 = 24.7 kcal mol^{-1} from Supplementary Figures 77 and 78.

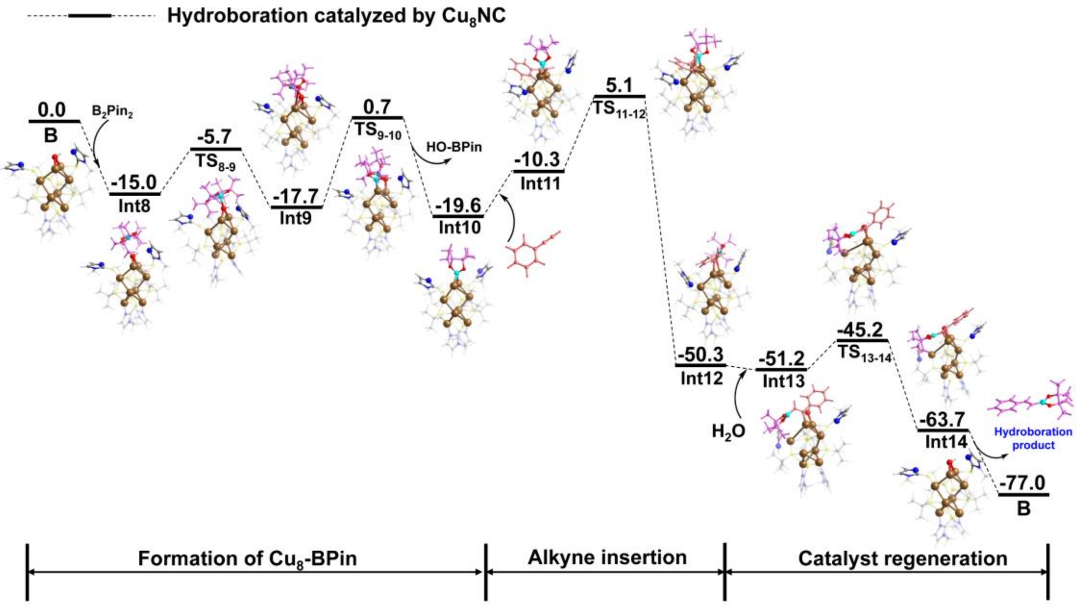

Supplementary Figure 77. DFT-computed Gibbs free energy profile. DFT-computed Gibbs free energy profile (ΔG in kcal/mol) for the hydroboration catalyzed by microcrystalline Cu$_8$NC.

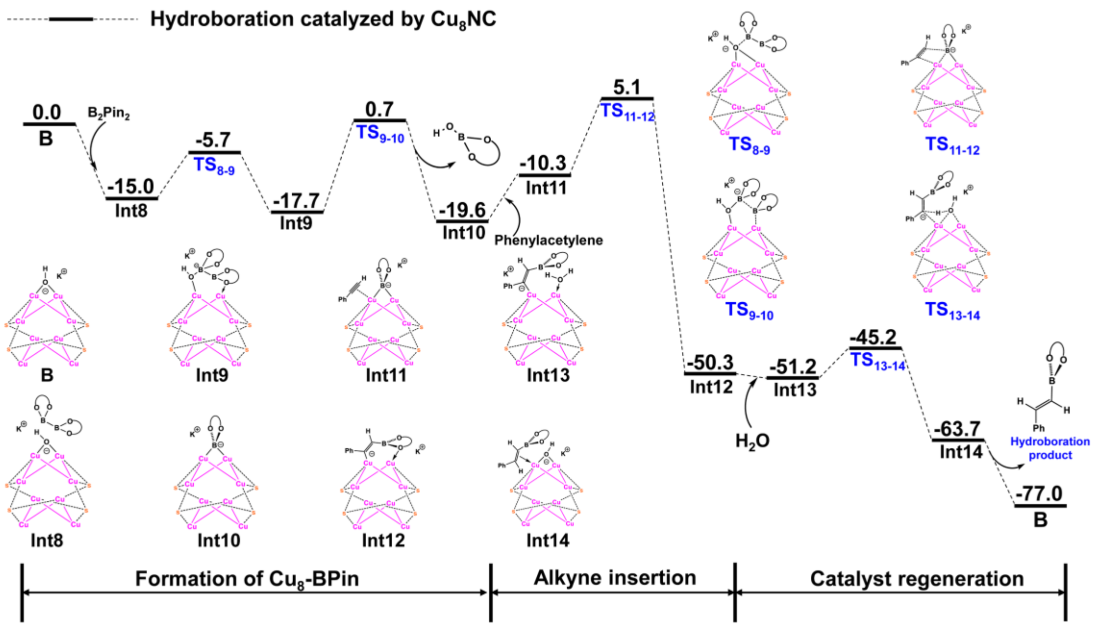

Supplementary Figure 78. Energy profile calculated for the microcrystalline Cu$_8$NC catalyzed hydroboration reaction. The relative free energies are given in kcal/mol.

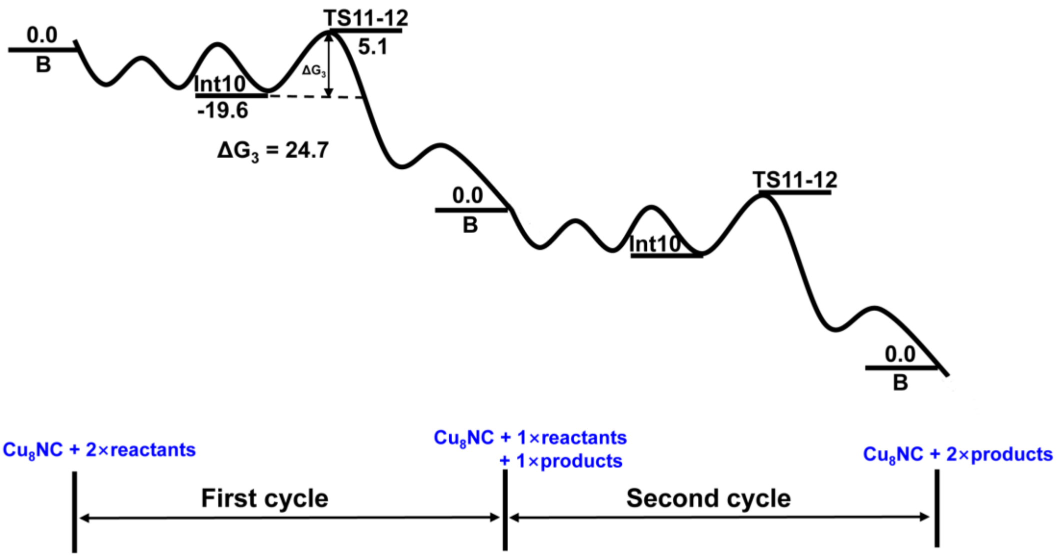

Supplementary Figure 79. Schematic illustration of the energy profile. Schematic illustration of the energy profile for an additional catalytic cycle (phenylacetylene as the alkyne substrate), which includes energy profile given the microcrystalline Cu_8NC catalyzed hydroboration reaction in Supplementary Figure 78. The relative Gibbs energies (in black) are given in kcal/mol.

Energy difference between the barrier of Cu_4NC and Cu_8NC

As shown in Supplementary Table 2, we tested some popular functionals in Supplementary Table 2, the golden standard M06 functional enlarge the energy difference to 12.8 kcal/mol, (7.9 for dispersion corrected M06). B3LYP functional gives similar energy difference with PBE0 functional. In terms of basis set, 6-311+G** functional gives a larger difference of barrier difference. The above method can well fit our experiment (Supplementary Table 2).

Supplementary Table 2. Energy difference between the barrier of Cu_4NC and Cu_8NC tested for different functionals and basis set.

Method	Energy difference of barrier
PBE0-D3/def2svp	1.8 kcal/mol
M06-D3/def2svp	7.9 kcal/mol
M06/def2svp	12.8 kcal/mol
B3LYP-D3/def2svp	1.6 kcal/mol
PBE0/6-311+G**	8.4 kcal/mol

Supplementary Table 3. Theoretical calculations regarding rate-determining intermediates and the rate-determining transition states for the hydroboration catalyzed by Cu_4NC monomer, dimer and trimer.

States	The barrier (kcal/mol)	Structures of TS_{4-5}
Cu_4NC monomer	22.9 kcal/mol	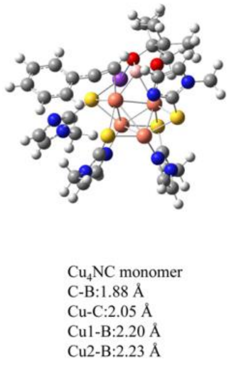
Cu_4NC dimer	20.6 kcal/mol	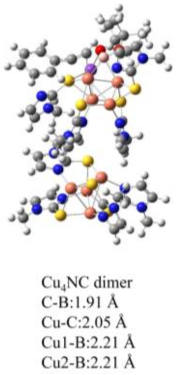
Cu_4NC trimer	22.0 kcal/mol	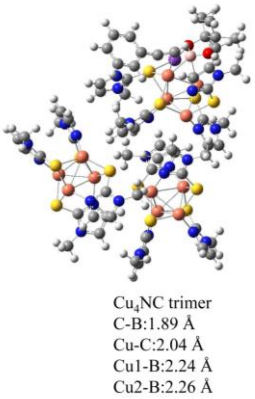

The Preliminary Kinetic Studies of the Hydroboration Reaction

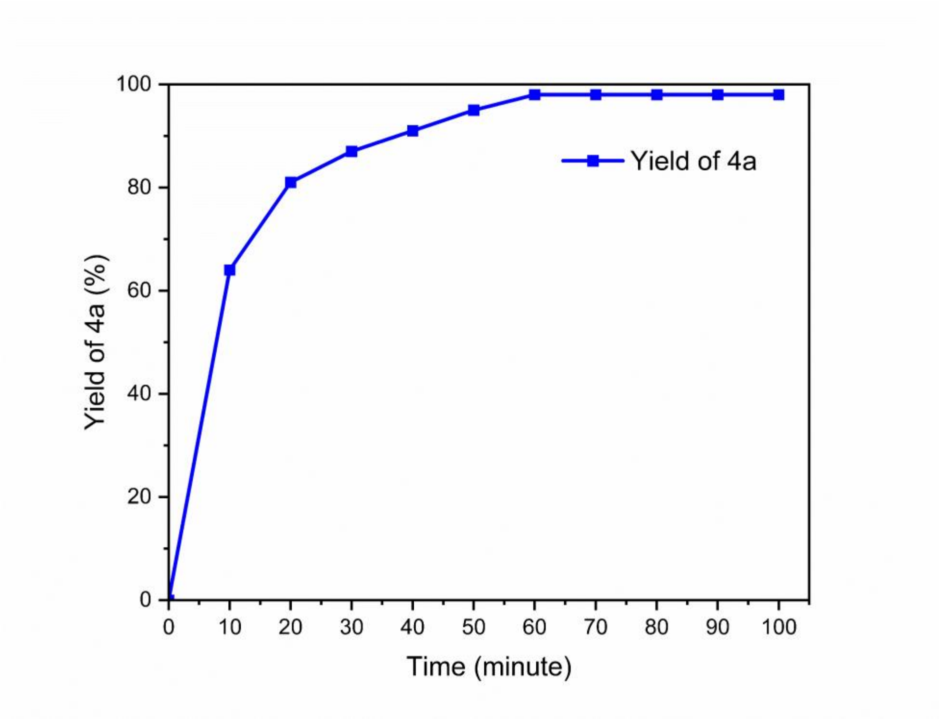

Supplementary Figure 80. Time-rate curve of hydroboration. Catalytic performances of 2.0 mol% Cu_4NC in hydroboration reaction of phenylacetylene at room temperature under air atmosphere.

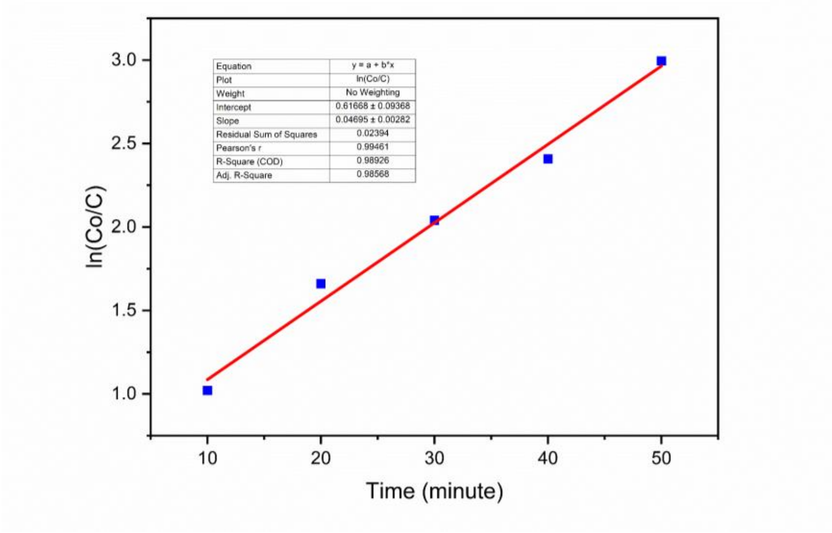

Supplementary Figure 81. Kinetic curve of hydroboration. Kinetic curve ($\ln(C_0/C)$ vs. t) of hydroboration reaction of phenylacetylene by using 2.0 mol% Cu_4NC as catalyst. C_0 and C are the phenylacetylene concentrations before the reaction and at a reaction time of t, respectively.

The *in situ* Raman Experiment

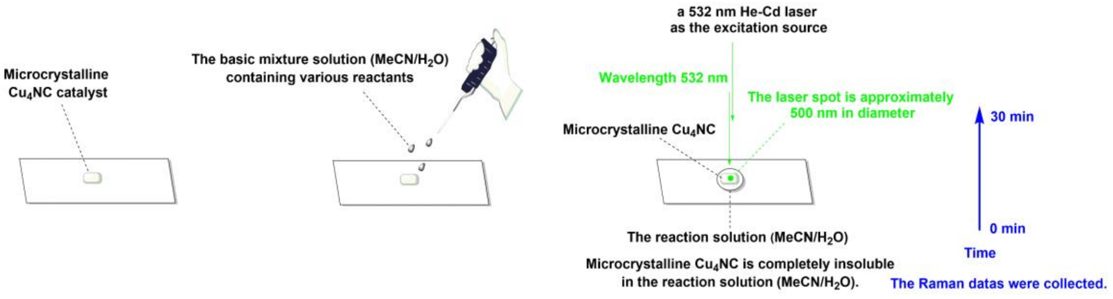

Supplementary Figure 82. The test method. The test method of the Raman spectra.

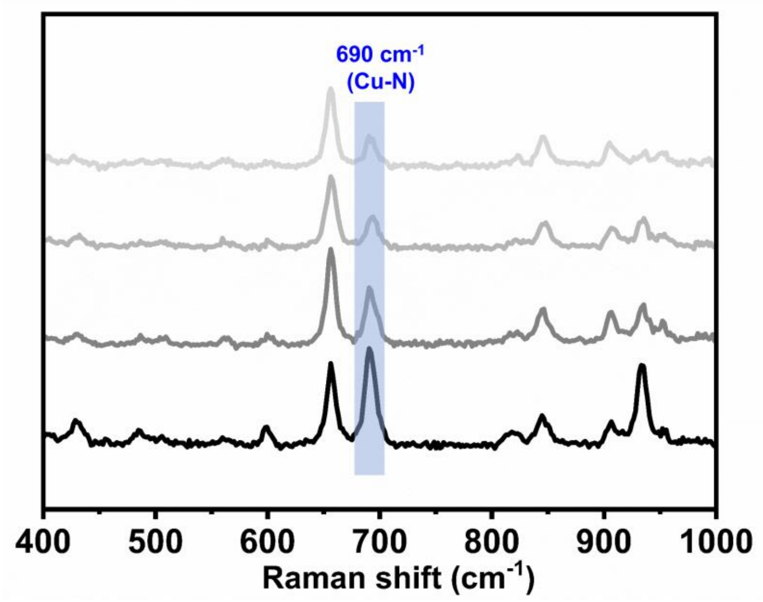

Supplementary Figure 83. Time-dependent *in situ* Raman spectra of the hydroboration reaction catalyzed by microcrystalline Cu_4NC catalyst (from bottom to top: 1min, 10min, 15min, 20min). Reaction conditions: the basic mixture solution ($MeCN/H_2O$) containing various reactants was dropped onto microcrystalline Cu_4NC catalyst to react under air atmosphere at room temperature.

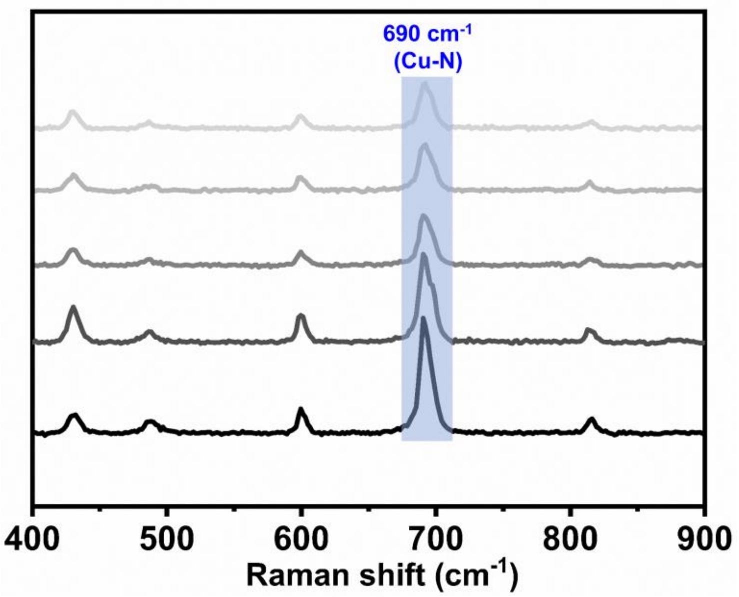

Supplementary Figure 84. Time-dependent *in situ* Raman spectra of the Cu_4NC + K_2CO_3 (from bottom to top: 1 min, 10 min, 15 min, 20 min, 30 min). Reaction conditions: the mixture solution (MeCN/H_2O) of K_2CO_3 was dropped onto microcrystalline Cu_4NC catalyst to react under air atmosphere at room temperature.

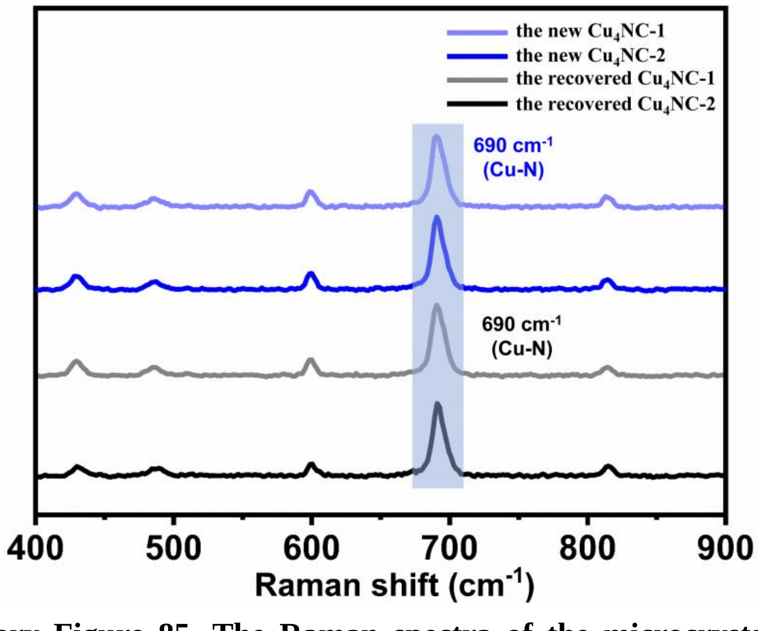

Supplementary Figure 85. The Raman spectra of the microcrystalline Cu_4NC before and after catalysis. The new Cu_4NC-1 and the new Cu_4NC-2 represent the Raman spectra of microcrystalline Cu_4NC catalysts prepared from different batches. The recovered Cu_4NC-1 and the recovered Cu_4NC-2 represent the Raman spectra of microcrystalline Cu_4NC catalysts recovered from different batches.

The *in situ* Fourier Transform Infrared (FT-IR) Experiment

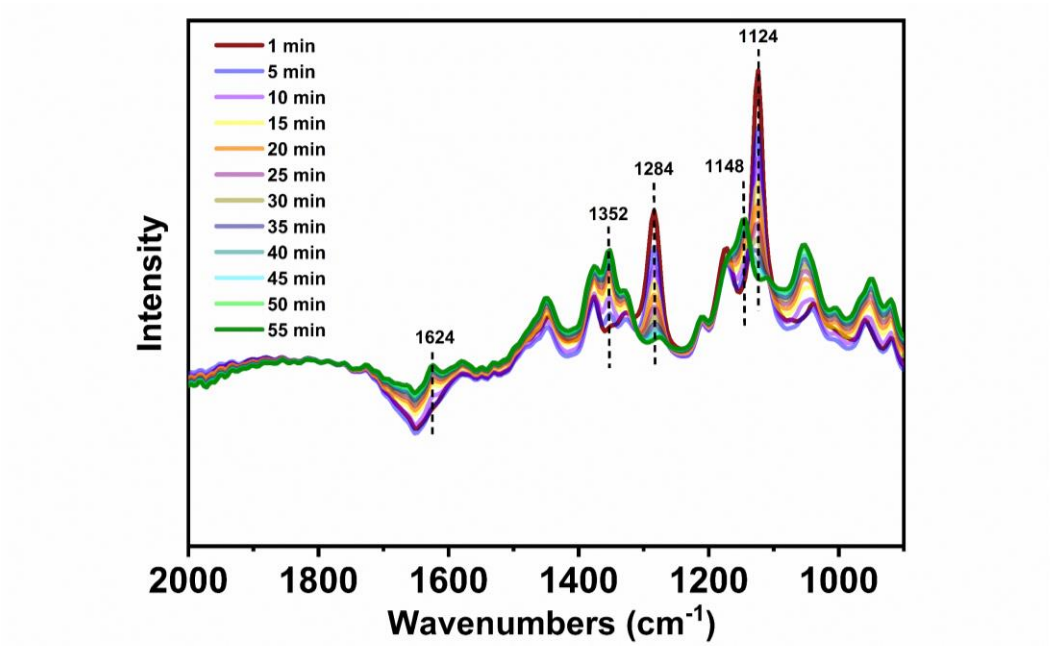

Supplementary Figure 86. Time-dependent *in situ* FT-IR spectra of the hydroboration reaction catalyzed by microcrystalline Cu_4NC catalyst. Reaction conditions: Phenylacetylene 1 (0.2 mmol, 1.0 equiv.), B_2Pin_2 2 (0.44 mmol, 2.2 equiv.), Cu_4NC catal. (0.004 mmol, 2.0 mol%) and K_2CO_3 (0.44 mmol, 2.2 equiv.) were added to the mixture solvent (2.0 mL, MeCN/H_2O 4:1) under air atmosphere at room temperature and allowed to react for 1 h.

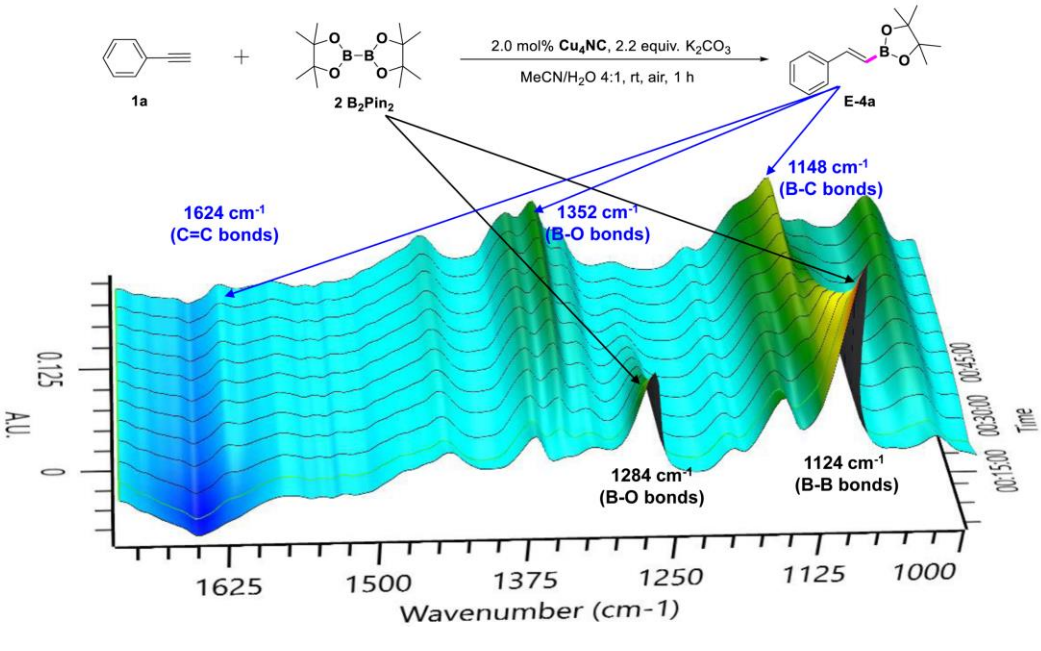

Supplementary Figure 87. The *in situ* FTIR spectra for hydroboration. The top view of the three-dimensional surface of the *in situ* FTIR spectra for hydroboration process catalyzed by microcrystalline Cu_4NC catalyst.

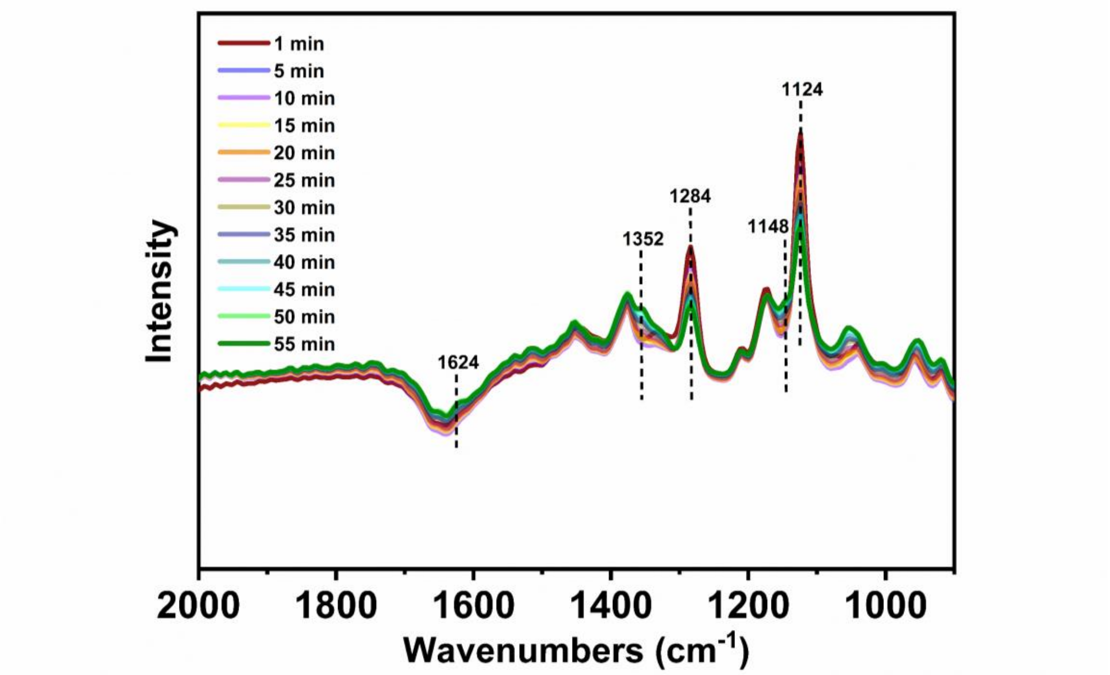

Supplementary Figure 88. Time-dependent *in situ* FT-IR spectra of the hydroboration reaction catalyzed by microcrystalline Cu_8NC catalyst. Reaction conditions: Phenylacetylene 1 (0.2 mmol, 1.0 equiv.), B_2Pin_2 2 (0.44 mmol, 2.2 equiv.), Cu_8NC catal. (0.004 mmol, 2.0 mol%) and K_2CO_3 (0.44 mmol, 2.2 equiv.) were added to the mixture solvent (2.0 mL, MeCN/H_2O 4:1) under air atmosphere at room temperature and allowed to react for 1 h.

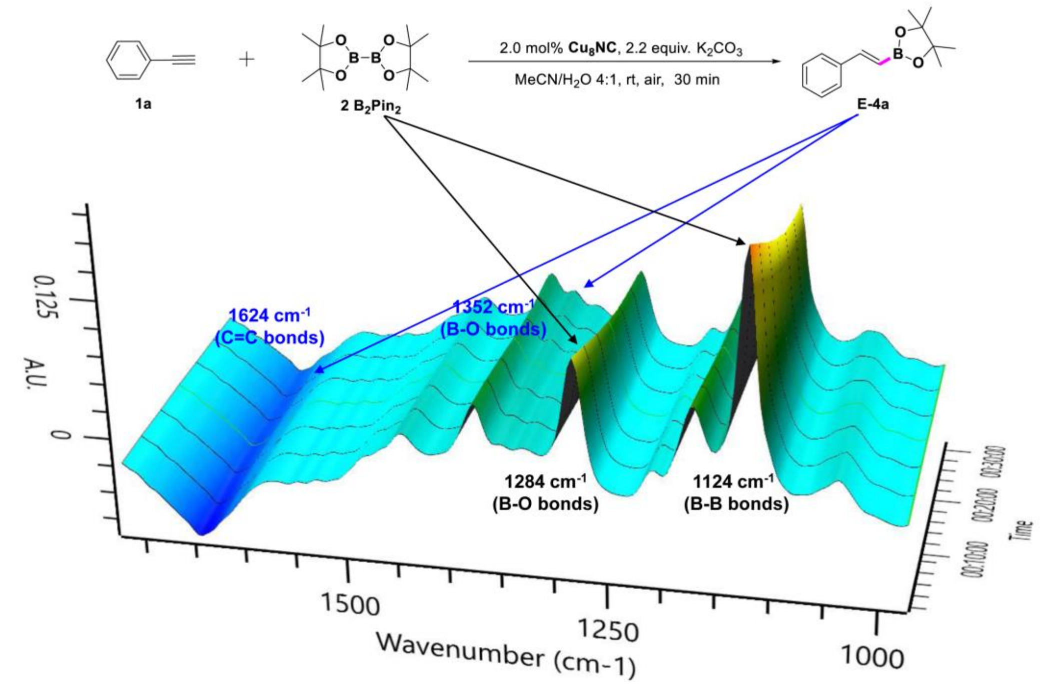

Supplementary Figure 89. The *in situ* FTIR spectra for hydroboration. The top view of the three-dimensional surface of the *in situ* FTIR spectra for hydroboration process catalyzed by microcrystalline Cu_8NC catalyst.

Deprotection of 1,3,5-Trimethoxybenzene Catalyzed by Microcrystalline Cu Clusters

To further prove that 1,3,5-trimethoxybenzene can be utilized as an internal standard, a control experiments was carried out that the 1,3,5-trimethoxybenzene, which replaced phenylacetylene as substrate, was been added into our copper cluster catalytic system. When the reaction time reaches 1 hour, the mesitylene as the standard was added into the reaction system. The reaction result was monitored by the ^{1}H NMR spectrum, showing that the content of 1,3,5-trimethoxybenzene has not decreased (Supplementary Figures 87–90). It further illustrated that 1,3,5-trimethoxybenzene is an inert substrate in our catalytic system. The experiment shows that 1,3,5-trimethoxybenzene can be utilized as an internal standard in our catalytic system.

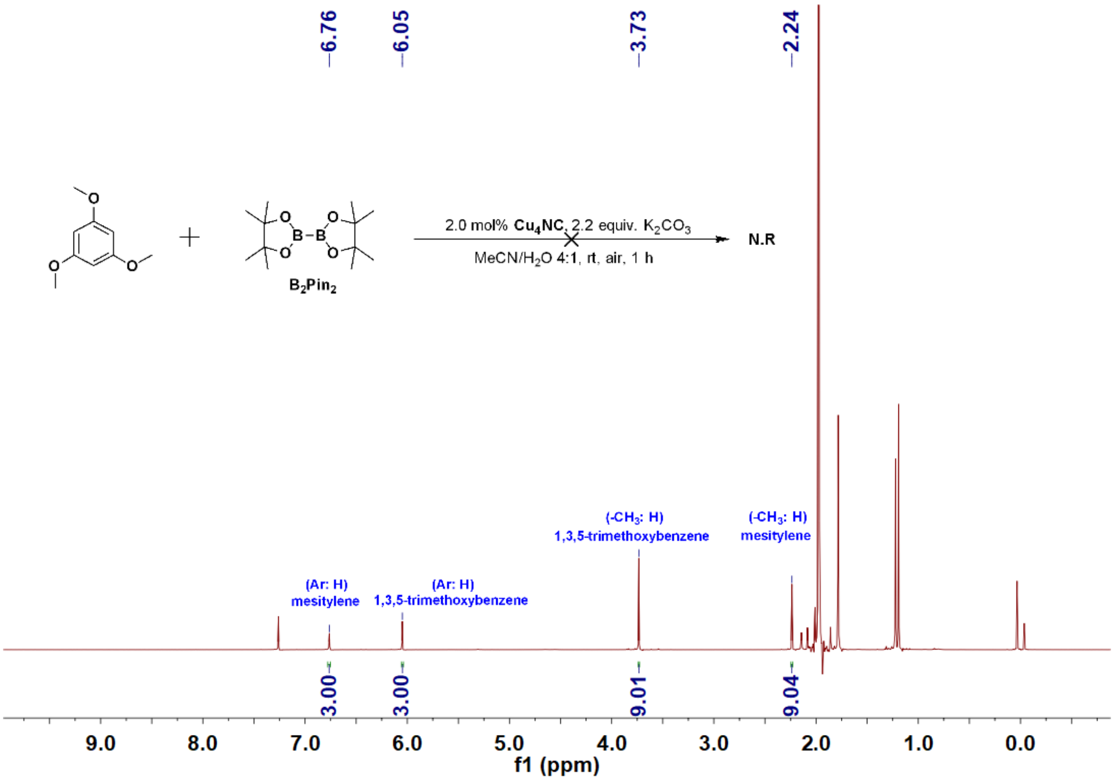

Supplementary Figure 90. The *in situ* ^{1}H NMR spectrum of deprotection of 1,3,5-trimethoxybenzene. The *in situ* ^{1}H NMR spectrum of deprotection of 1,3,5-trimethoxybenzene catalyzed by microcrystalline Cu_4NC catalyst.

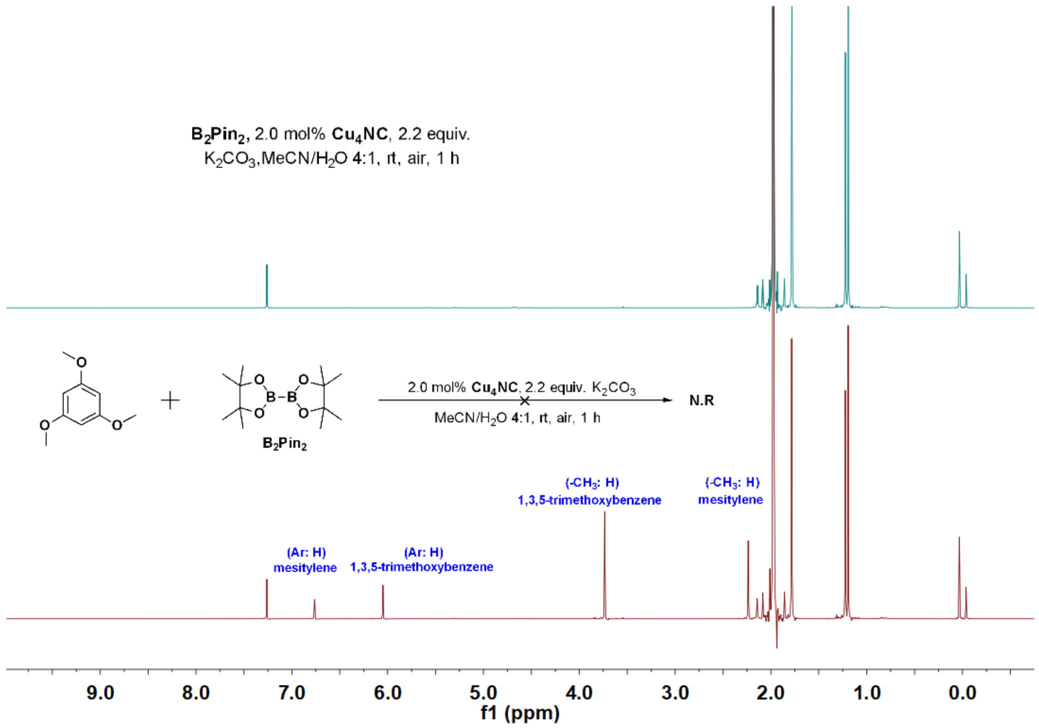

Supplementary Figure 91. The *in situ* ^{1}H NMR spectra of deprotection of 1,3,5-trimethoxybenzene. The *in situ* ^{1}H NMR spectra of deprotection of 1,3,5-trimethoxybenzene catalyzed by microcrystalline Cu_4NC catalyst. Top: the control group. Bottom: the experimental group.

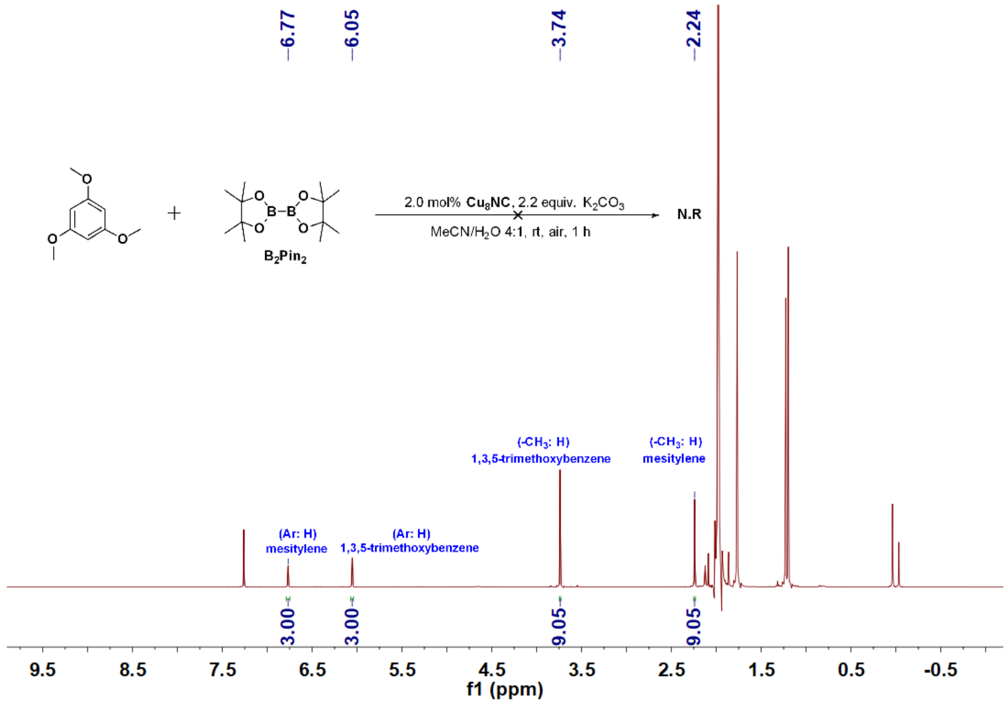

Supplementary Figure 92. The *in situ* ^{1}H NMR spectrum of deprotection of 1,3,5-trimethoxybenzene. The *in situ* ^{1}H NMR spectrum of deprotection of 1,3,5-trimethoxybenzene catalyzed by microcrystalline Cu_8NC catalyst.

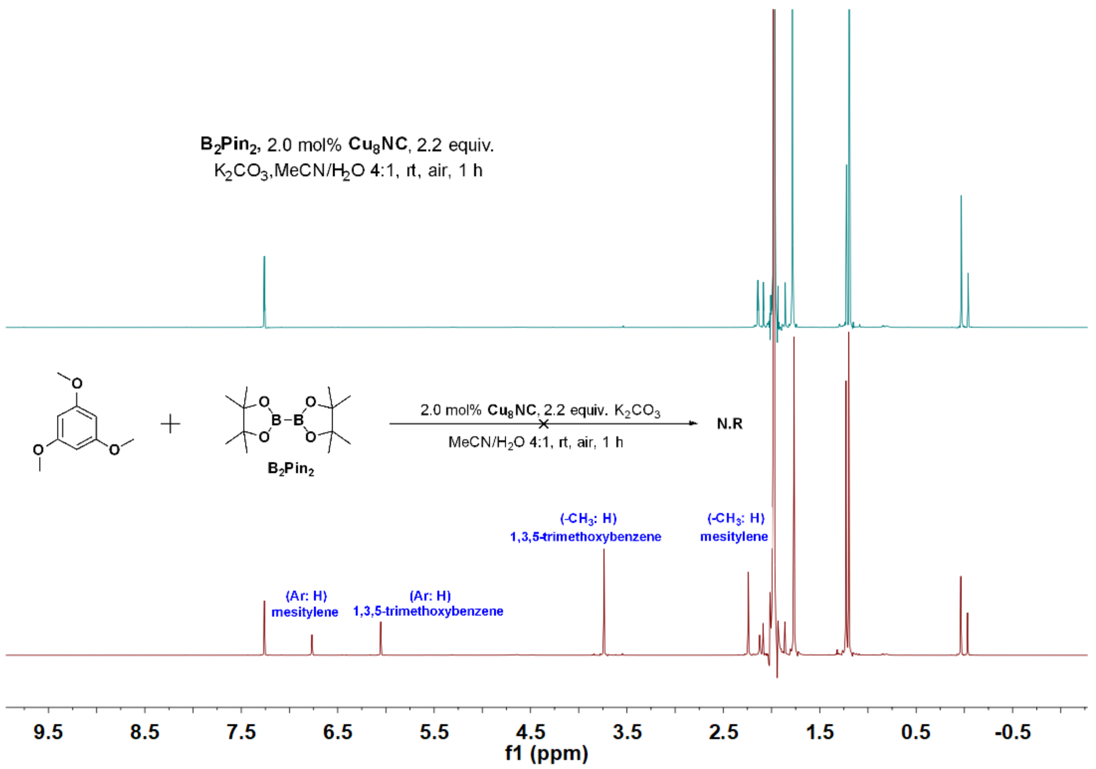

Supplementary Figure 93. The *in situ* ^{1}H NMR spectra of deprotection of 1,3,5-trimethoxybenzene. The *in situ* ^{1}H NMR spectra of deprotection of 1,3,5-trimethoxybenzene catalyzed by microcrystalline Cu_8NC catalyst. Top: the control group. Bottom: the experimental group.

Recyclability of the Cu_4NC Catalyzed Hydroboration Reaction

Recycling Procedure for Cu_4NC-Catalyzed the Hydroboration Reaction.

Under air atmosphere, phenylacetylene **1** (20.4 mg, 0.2 mmol, 1.0 eq), B_2Pin_2 **2** (111.8 mg, 0.44 mmol, 2.2 equiv.), Cu_4NC catalysts (2.8 mg, 2.0 mol%), K_2CO_3 (60.7 mg, 0.44 mmol, 2.2 equiv.) and the mixture solvent (2.0 mL, MeCN-H_2O, v/v 4/1) were added into a 10 mL flask. The reaction mixture was stirred at room temperature for 1 h. Conversions and yields were determined by ^{1}H NMR using 1,3,5-trimethoxybenzene as an internal standard. After the reaction is completed, the reaction mixture was centrifuged at 8000 rpm for 5 minutes. The microcrystalline Cu_4NC catalyst was filtered, washed with hexane and then dried in vacuum to recycle the microcrystalline Cu_4NC. The recycled microcrystalline Cu_4NC catalyst was used again in the next recycle. In this way, the hydroboration reaction of alkynes was conducted for 5 cycles with the microcrystalline Cu_4NC catalyst (Supplementary Figure 94).

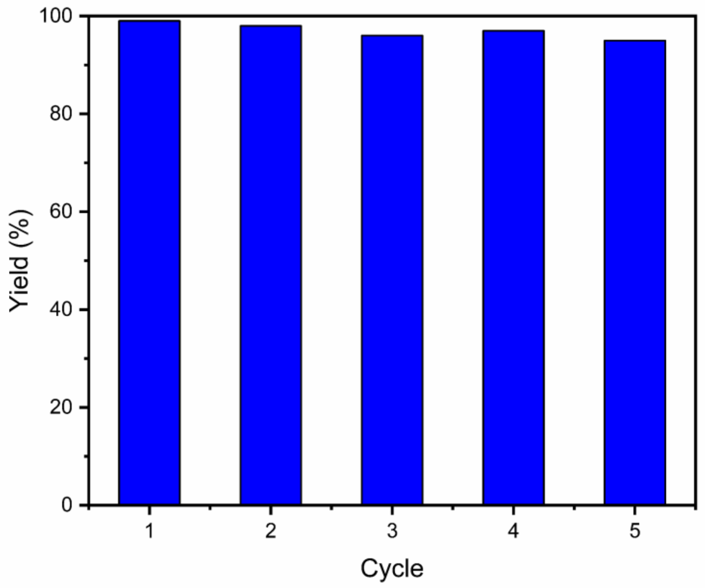

Supplementary Figure 94. Cyclic experiment. Recyclability of the microcrystalline Cu_4NC catalyzed hydroboration reaction in term of yield (1 h).

When we studied the recovery experiments, we found that the weak interaction between recycled microcrystalline Cu_4NC catalyst and filter paper can lead to unnecessary loss of microcrystalline Cu_4NC catalyst, thereby limiting the number of cycles of catalysis. To eliminate the influence of the weak interaction, the repeated dosing projects were carried out. In this way, the hydroboration reaction of alkynes was conducted for more than 20 cycles (Supplementary Figure 95). The repeated dosing experiments were conducted to further demonstrate that microcrystalline Cu_4NC has excellent catalytic activity and stability.

Under air atmosphere, phenylacetylene **1** (20.4 mg, 0.2 mmol, 1.0 eq), B_2Pin_2 **2** (111.8 mg, 0.44 mmol, 2.2 equiv.), Cu_4NC catalysts (2.8 mg, 2.0 mol%), K_2CO_3 (60.7 mg, 0.44 mmol, 2.2 equiv.) and the mixture solvent (2.0 mL, MeCN-H_2O, v/v 4/1) were added into a 50 mL flask. The reaction mixture was stirred at room temperature for 1 h. Conversions and yields were determined by ^1H NMR using 1,3,5-trimethoxybenzene as an internal standard. Then the reactants [phenylacetylene **1** (20.4 mg, 0.2 mmol, 1.0 eq), B_2Pin_2 2 (111.8 mg, 0.44 mmol, 2.2 equiv.), K_2CO_3 (60.7 mg, 0.44 mmol, 2.2 equiv.) and the mixture solvent (1.0 mL, MeCN-H_2O, v/v 4/1)] were injected to the reaction system twenty-one consecutive times. The conversion and yield were determined by ^1H NMR using 1,3,5-trimethoxybenzene as an internal standard.

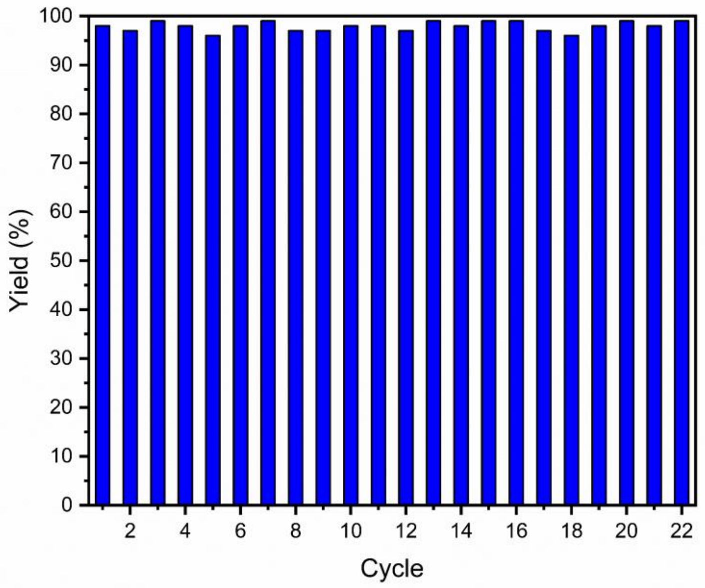

Supplementary Figure 95. The repeated dosing experiment. The repeated dosing of the microcrystalline Cu_4NC catalyzed hydroboration reaction in term of yield (1 h).

Monitor the State of the Microcrystalline Cu_4NC during the Catalytic Process Using PXRD

To verify the stability of the catalyst and to verify that the crystal state of microcrystalline Cu_4NC has been maintained in hydroboration, three parallel experiments of hydroboration catalyzed by microcrystalline Cu_4NC also were carried out. The parallel experiments will be stopped at the 10th minute, the 30th minute and the 60th minute. The reaction mixture was centrifuged at 8000 rpm for 5 minutes. The microcrystalline Cu_4NC catalyst was filtered, washed with hexane and then dried in vacuum to recycle the microcrystalline Cu_4NC. Recycled microcrystalline Cu_4NC catalyst was further characterized by PXRD (Supplementary Figure 96). It showed that the characteristic peaks of the catalyzed microcrystalline Cu_4NC at different time (the 10th minute, the 30th minute and the 60th minute) matched well with the characteristic peaks of the new microcrystalline Cu_4NC. In a word, the results illustrated that the hydroboration happens on the surface of microcrystalline Cu_4NC, and microcrystalline Cu_4NC is very stable in hydroboration process.

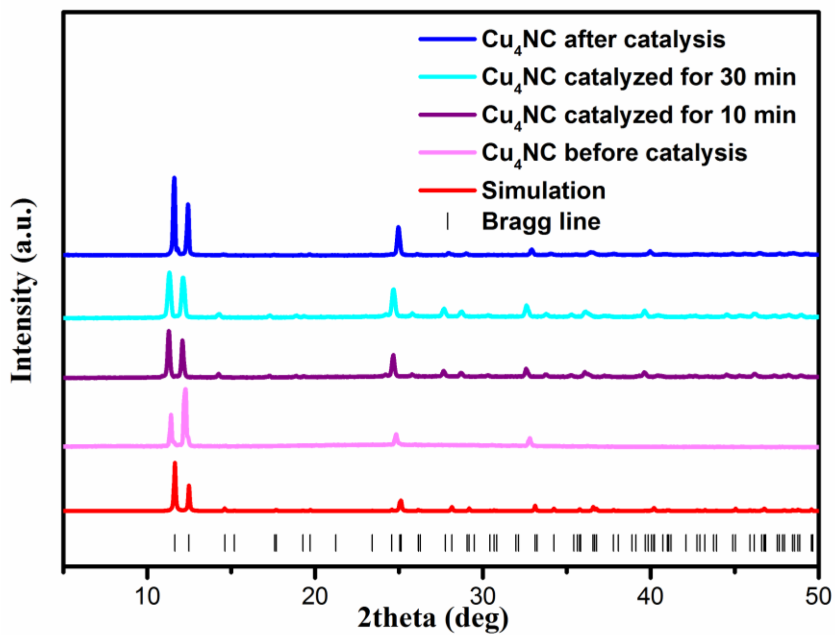

Supplementary Figure 96. PXRD patterns of Cu_4NC before catalysis and after catalysis. PXRD patterns of Cu_4NC before catalysis and after the 10th minute, the 30th minute and the 60th minute catalysis.

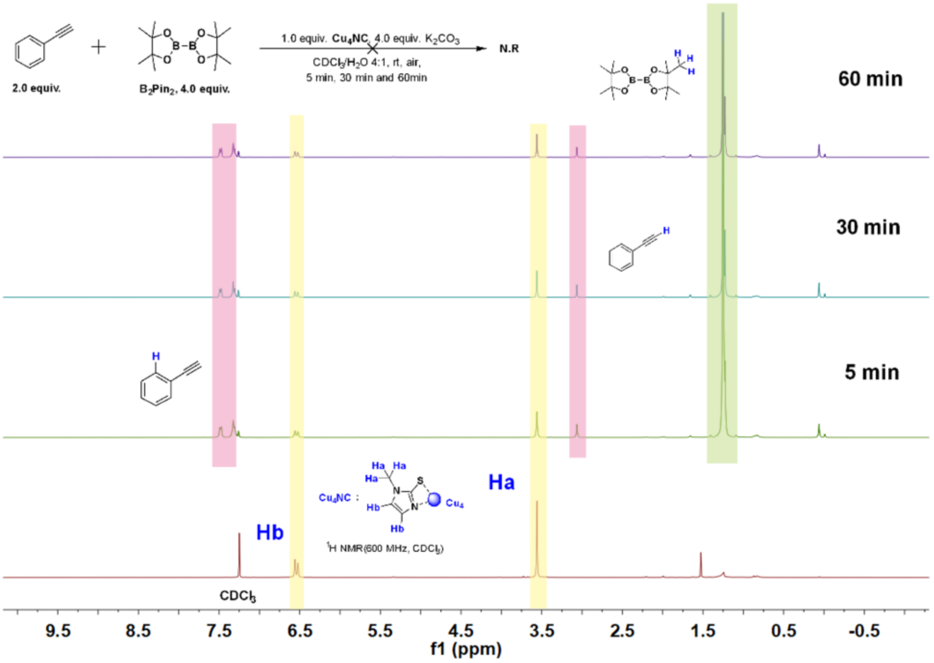

Supplementary Figure 97. Time-dependent *in situ* ^{1}H NMR spectra of the hydroboration reaction. Time-dependent *in situ* ^{1}H NMR spectra of the hydroboration reaction catalyzed by microcrystalline Cu_4NC catalyst in the mixture solvent ($CDCl_3$-H_2O). Reaction conditions: Phenylacetylene 1 (2.0 equiv.), B_2Pin_2 2 (4.0 equiv.), Cu_4NC catal. (1.0 equiv.) and K_2CO_3 (4.0 equiv.) were added to the mixture solvent (2.0 mL, $CDCl_3$-H_2O) under air atmosphere at room temperature and allowed to react for 1 h. No product was found.

Monitor the Catalytic Ability of the Supernatant Using *in situ* ^{1}H NMR

To verify that the supernatant lacks any catalytic ability, corresponding experiments and characterization tests were carried out. First, the hydroboration reaction catalyzed by microcrystalline Cu_4NC was conducted. After 5 minutes, the microcrystalline Cu_4NC was removed from the catalytic system. Continuous stirring of supernatant was further monitored by the *in situ* ^{1}H NMR at the 5th minute, 20th minute, 30th minute and 60th minute (Supplementary Figure 98), showing that no further conversion was observed within this solution. Second, the other control experiment was carried out. When the reaction was completed, the microcrystalline Cu_4NC was removed from the catalytic system. The additional aliquot of the substrates was added to the supernatant, which was further monitored by the *in situ* ^{1}H NMR at the 1th minute, 20th minute, 30th minute and 60th minute (Supplementary Figure 99). The result showed that no conversion of the added material was observed. All of these observations and experimental results suggested that the supernatant lacks any catalytic ability. The possibility of forming a soluble, catalytically competent, and copper-free species under reaction conditions has been precluded. At the same time, the supernatants were characterized by inductively coupled plasma mass spectrometric (ICP-MS) measurement, showing that no Cu^+ ions was found in the reaction supernatant, suggesting that Cu^+ ions were not leached from the metal cluster catalyst into solution over the course of the reaction. These results also suggested that key intermediate A is completely insoluble in the reaction solvent system (MeCN-H_2O).

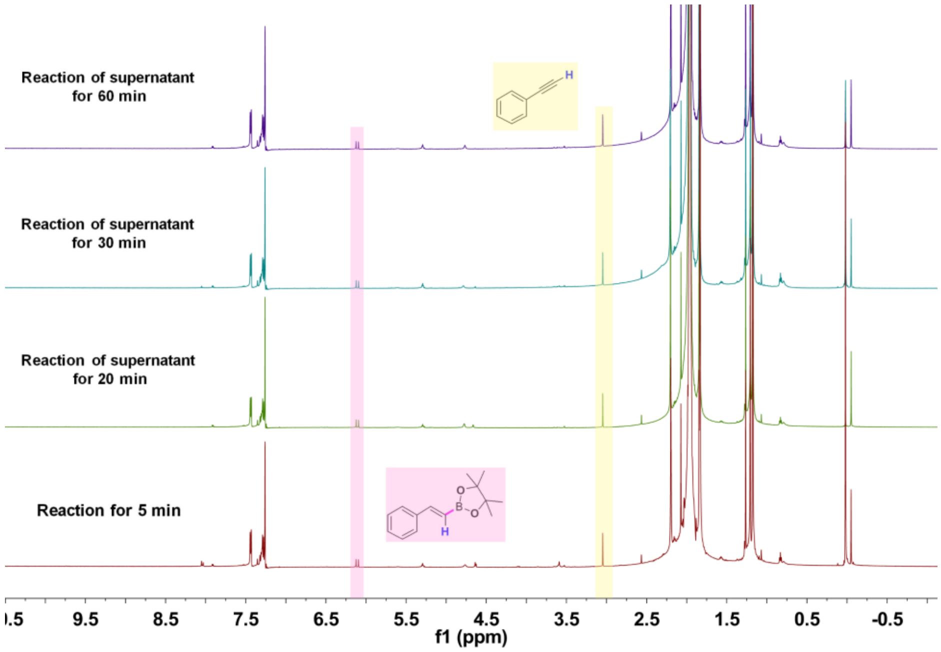

Supplementary Figure 98. Time-dependent *in situ* ^{1}H NMR spectra of the hydroboration reaction. Reaction conditions: Phenylacetylene 1 (1.0 equiv.), B_2Pin_2 2 (2.2 equiv.), Cu_4NC catal. (10% mol) and K_2CO_3 (2.2 equiv.) were added to the mixture solvent (2.0 mL, CD_3CN-H_2O) under air atmosphere at room temperature and allowed to react for 5 min. The catalyst was separated from the supernatant of the reaction system by filtration. The supernatant was stirred for 1 h.

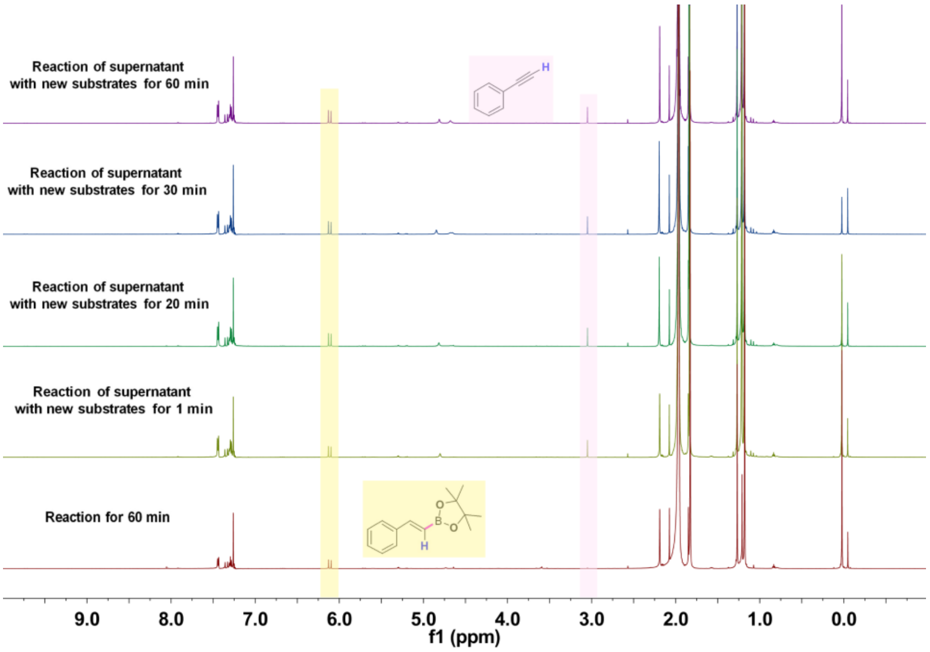

Supplementary Figure 99. Time-dependent *in situ* ^{1}H NMR spectra of the hydroboration reaction. Reaction conditions: Phenylacetylene 1 (1.0 equiv.), B_2Pin_2 2 (2.2 equiv.), Cu_4NC catal. (10% mol) and K_2CO_3 (2.2 equiv.) were added to the mixture solvent (2.0 mL, CD_3CN-H_2O) under air atmosphere at room temperature and allowed to react for 1 h. The catalyst was separated from the supernatant of the reaction system by filtration. Phenylacetylene 1 (1.0 equiv.), B_2Pin_2 2 (2.2 equiv.) and K_2CO_3 (2.2 equiv.) were added to the supernatant. The reaction was stirred for 1 h.

Derivatization of the Products from Hydroboration of Alkynes

(E)-(2-bromovinyl) benzene (7a)

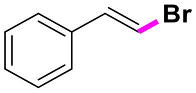

E-7a

Under air atmosphere, the copper salt $CuBr_2$ (134.0 mg, 0.6 mmol), **E-4a** compound (46.0 mg, 0.2 mmol) and the mixture solvent (3.0 mL, EtOH-H_2O, v/v 1/1) were added into 25 mL Schlenk tube to react at 100 °C for 24 h. The reactions were monitored by TLC. When **E-4a** compound was consumed, the reaction was cooled to room temperature, then quenched and concentrated. The crude products were then purified by column chromatography (PE-EA, v/v 1/0) to give the target products as a colorless oil with an overall isolated yield: 81% (29.4 mg). **^{1}H NMR** (600 MHz, $CDCl_3$) δ 7.36 – 7.28 (m, 5H), 7.12 (d, *J* = 14.0 Hz, 1H), 6.78 (d, *J* = 14.0 Hz, 1H). **^{13}C{^{1}H} NMR** (151 MHz, $CDCl_3$) δ 137.2, 135.9, 128.8, 128.3, 126.1, 106.5.

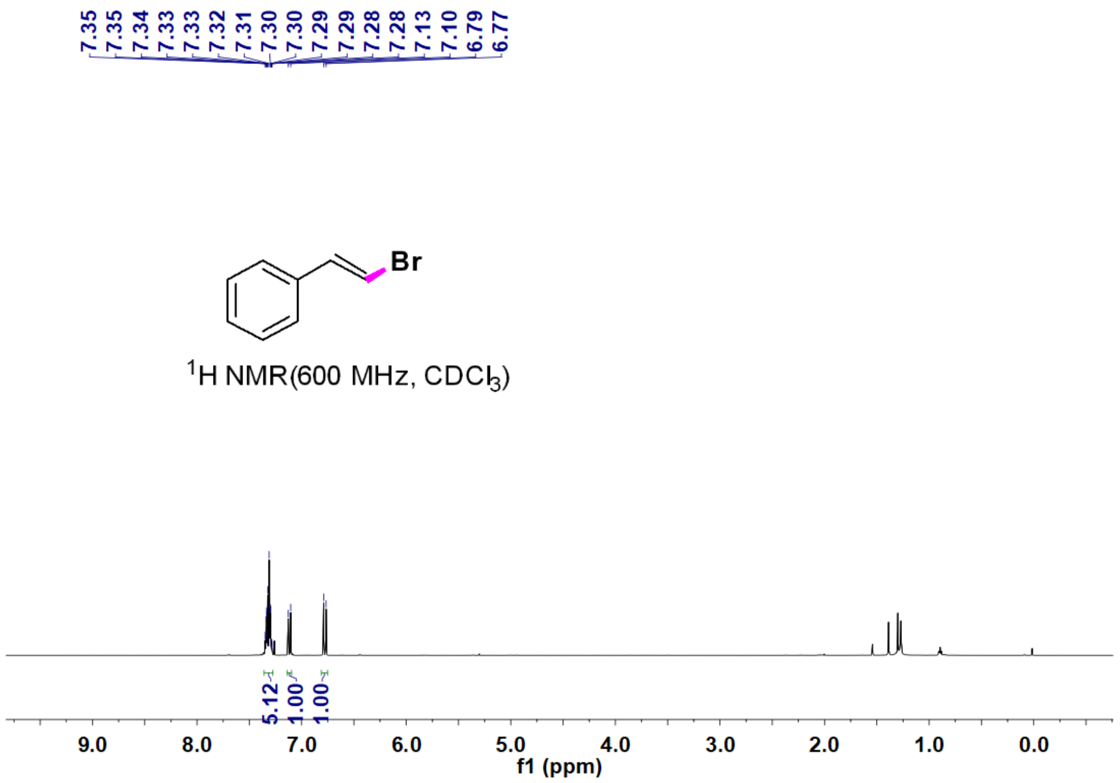

Supplementary Figure 100. Characterization of compound 7a. ^{1}H NMR spectrum of compound **7a** in $CDCl_3$.

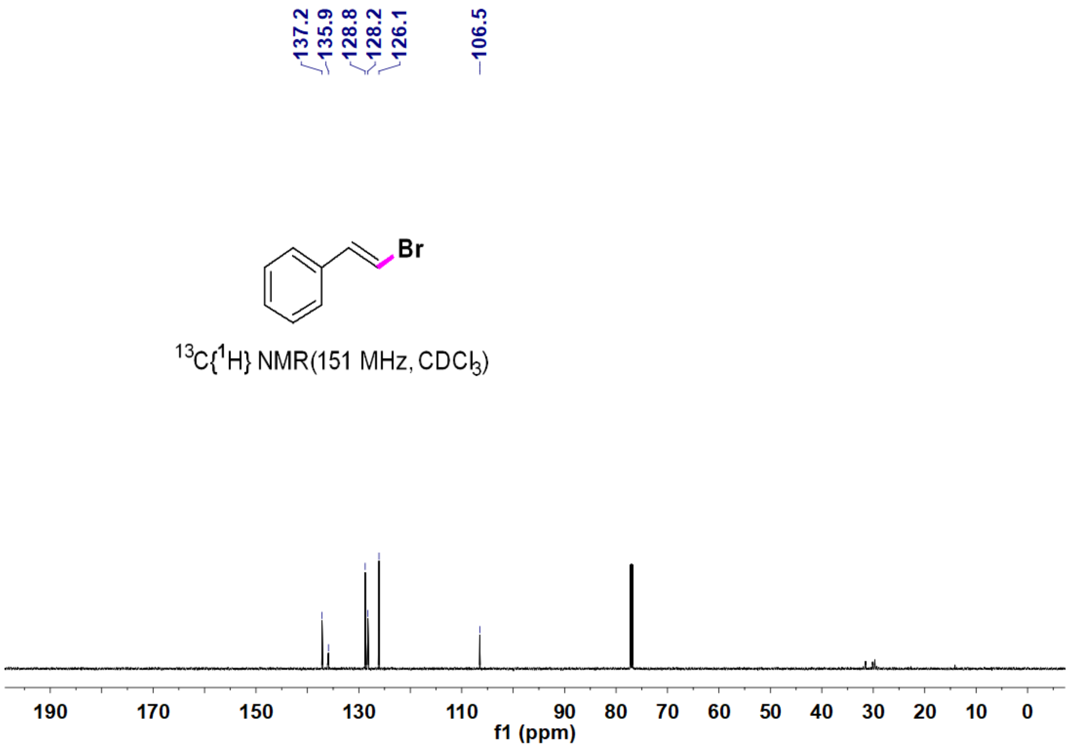

Supplementary Figure 101. Characterization of compound 7a. ^{13}C{^{1}H} NMR spectrum of compound **7a** in $CDCl_3$.

(E)-(2-iodovinyl) benzene (8a)

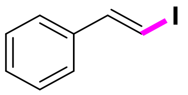

E-8a

Under air atmosphere, the copper salts CuI (3.8 mg, 0.02 mmol), o-phenanthroline (7.2 mg, 0.04 mmol) and the mixture solvent (2.0 mL, MeOH-H_2O, v/v 4/1) were added into 25 mL Schlenk tube to react at room temperature for 30 min. Then KI (49.8 mg, 0.3 mmol) and **E-4a** compound (46.0 mg, 0.2 mmol) were added in the reaction system to react at 80 °C for 12 h. The reactions were monitored by TLC. When **E-4a** compound was consumed, the reaction was cooled to room temperature, then quenched and concentrated. The crude products were then purified by column chromatography (PE-EA, v/v 1/0) to give the target products as a colorless oil with an overall isolated yield: 51% (23.5 mg). **^{1}H NMR** (600 MHz, $CDCl_3$) δ 7.44 (d, *J* = 14.9 Hz, 1H), 7.35 – 7.27 (m, 5H), 6.83 (d, *J* = 14.9 Hz, 1H). **^{13}C{^{1}H} NMR** (151 MHz, $CDCl_3$) δ 145.0, 137.7, 128.7, 128.4, 126.0, 76.6.

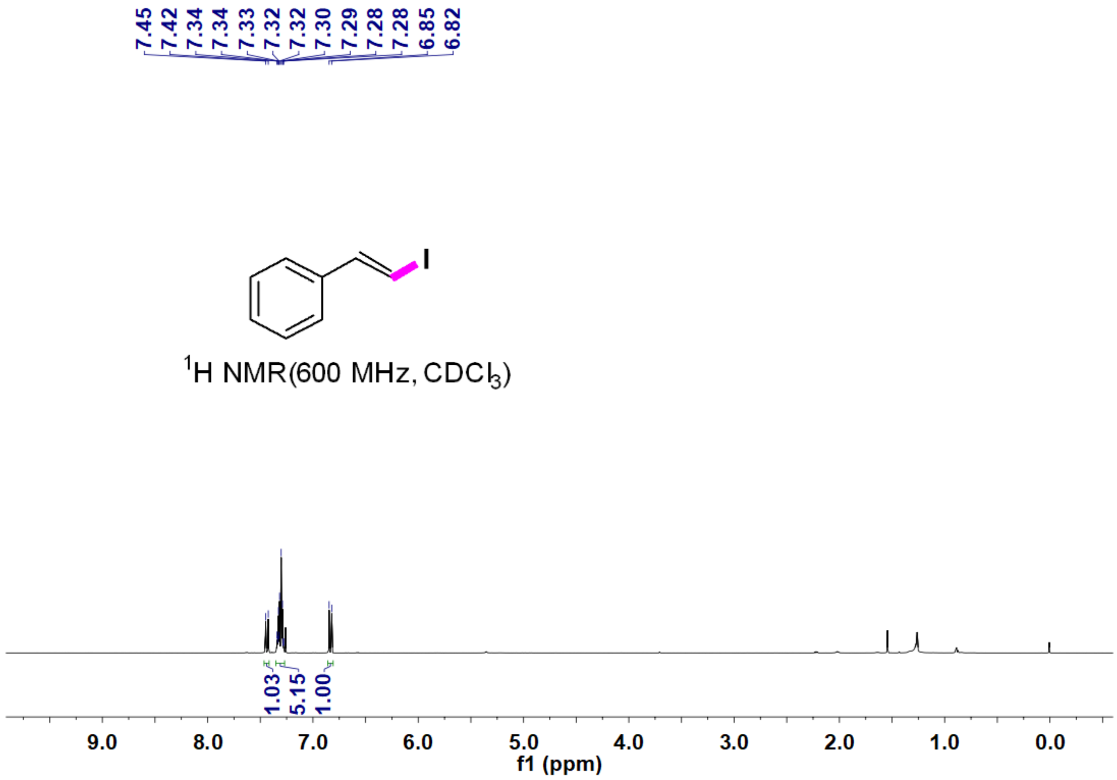

Supplementary Figure 102. Characterization of compound 8a. ^{1}H NMR spectrum of compound **8a** in $CDCl_3$

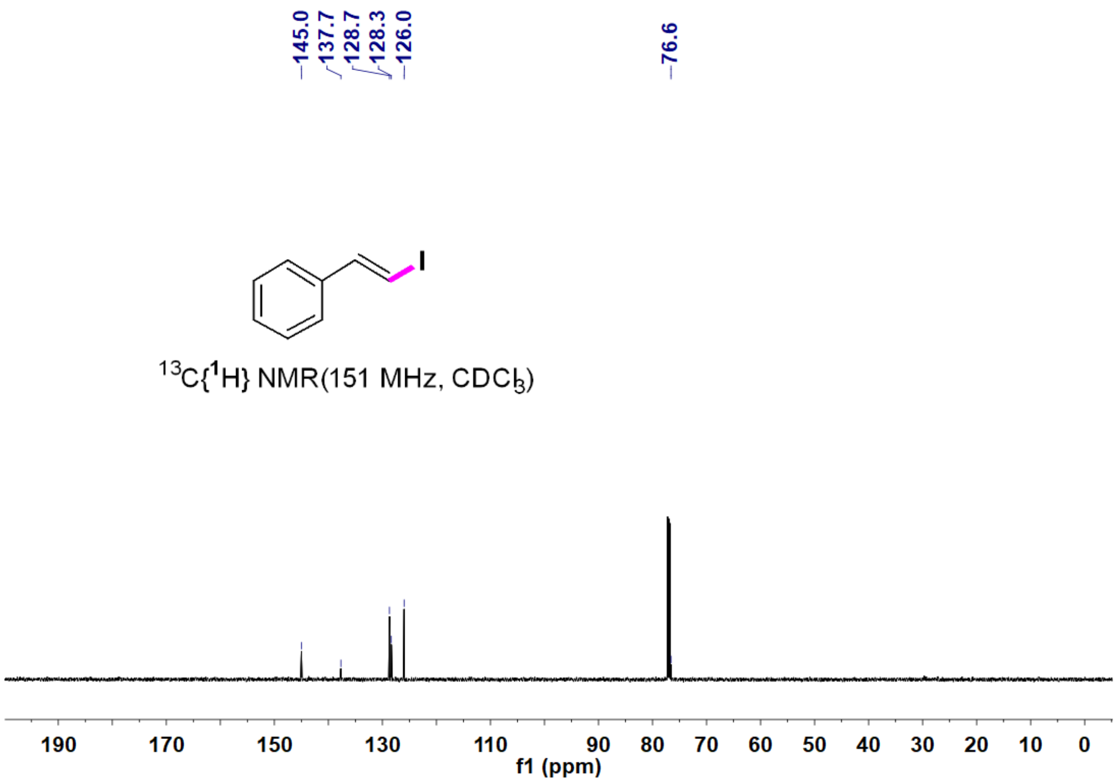

Supplementary Figure 103. Characterization of compound 8a. $^{13}C\{^1H\}$ NMR spectrum of compound **8a** in $CDCl_3$.

(E)-1-methyl-4-styrylbenzene (9a)

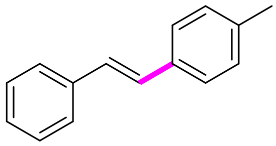

E-9a

Under N_2 atmosphere, the $Pd(PPh_3)_4$ (11.6 mg, 5.0 mol%), **E-4a** compound (46.0 mg, 0.2 mmol), Cs_2CO_3 (391.0 mg, 1.2 mmol), 4-iodotoluene (43.6 mg, 0.2 mmol) and the 4.0 mL THF solvent were added into 25 mL Schlenk tube to react at 60 °C overnight. The reactions were monitored by TLC. When **E-4a** compound was consumed, the reaction was cooled to room temperature, then quenched and concentrated. The crude products were then purified by column chromatography (PE-EA, v/v 40/1) to give the target products as a white solid with an overall isolated yield: 95% (36.9 mg). **^{1}H NMR** (600 MHz, $CDCl_3$) δ 7.51 – 7.47 (m, 2H), 7.41 (d, J = 8.1 Hz, 2H), 7.34 (t, J = 7.7 Hz, 2H), 7.26 – 7.22 (m, 1H), 7.16 (d, J = 7.9 Hz, 2H), 7.10 – 7.03 (m, 2H), 2.35 (s, 3H). **^{13}C{^{1}H} NMR** (151 MHz, $CDCl_3$) δ 137.5, 137.5, 134.6, 129.4, 128.6, 127.7, 127.4, 126.4, 126.4, 21.2.

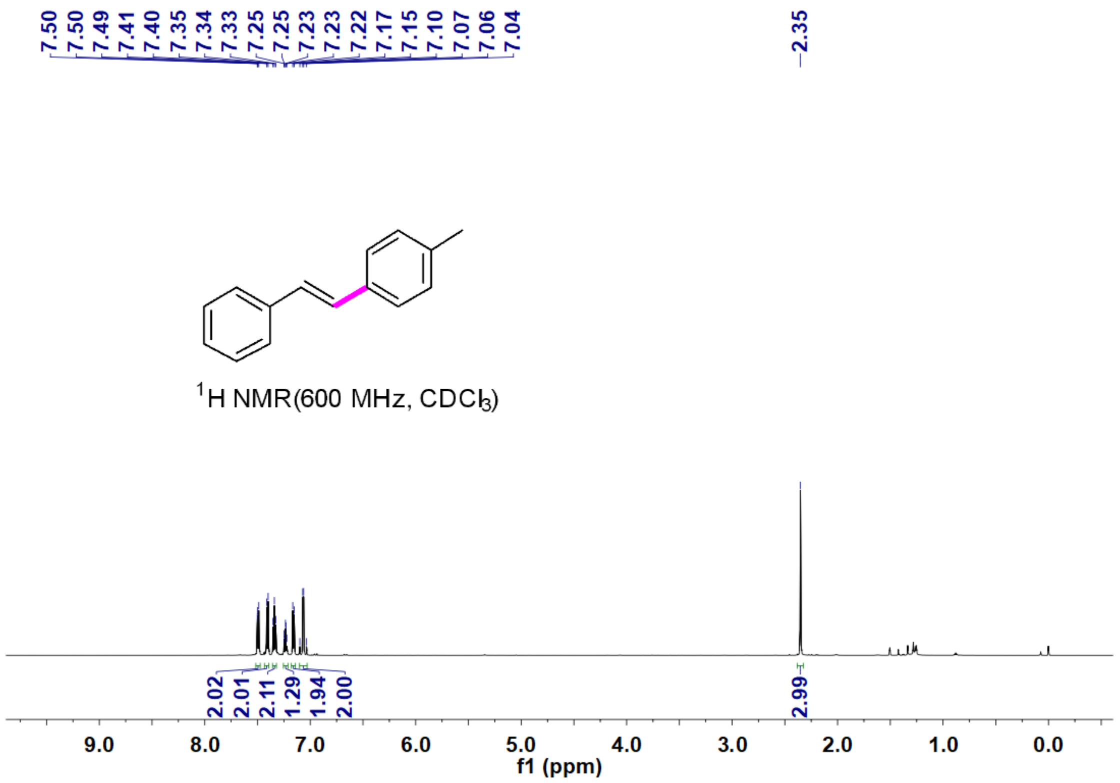

Supplementary Figure 104. Characterization of compound 9a. ^{1}H NMR spectrum of compound **9a** in $CDCl_3$.

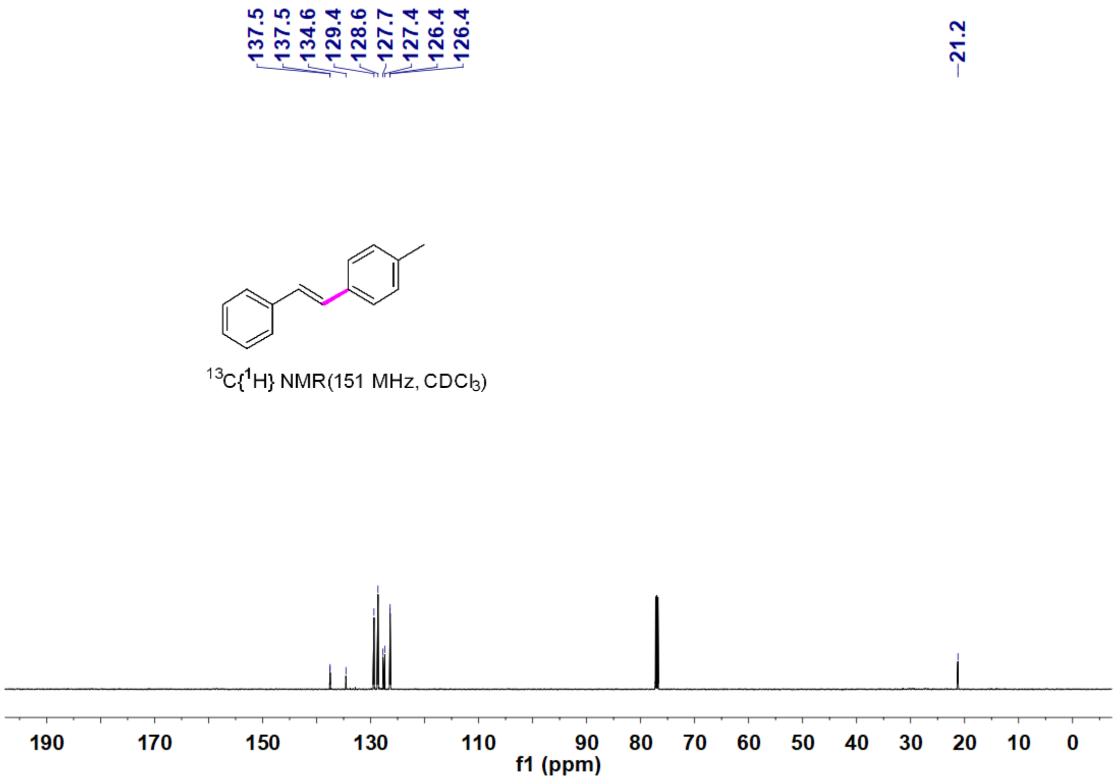

Supplementary Figure 105. Characterization of compound 9a. ^{13}C{^{1}H} NMR spectrum of compound **9a** in $CDCl_3$.

(E)-(2-azidovinyl)benzene (10a)

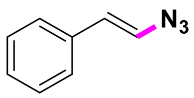

E-10a

Under air atmosphere, the copper salts $CuSO_4{\cdot}5H_2O$ (30.0 mg, 0.12 mmol), **E-4a** compound (46.0 mg, 0.2 mmol), NaN_3 (19.5 mg, 0.3 mmol) and the 0.8 mL MeOH solvent were added into 25 mL round-bottom flask to react at room temperature overnight. The reactions were monitored by TLC. When **E-4a** compound was consumed, the reaction was quenched and concentrated. The crude products were then purified by column chromatography (PE-EA, v/v 1/0) to give the target products as a yellow oil with an overall isolated yield: 92% (26.7 mg). **^{1}H NMR** (600 MHz, $CDCl_3$) δ 7.34 – 7.27 (m, 4H), 7.25 – 7.21 (m, 1H), 6.62 (d, J = 13.8 Hz, 1H), 6.28 (d, J = 13.8 Hz, 1H). **^{13}C{^{1}H} NMR** (151 MHz, $CDCl_3$) δ 135.0, 128.7, 127.4, 126.7, 125.8, 119.8.

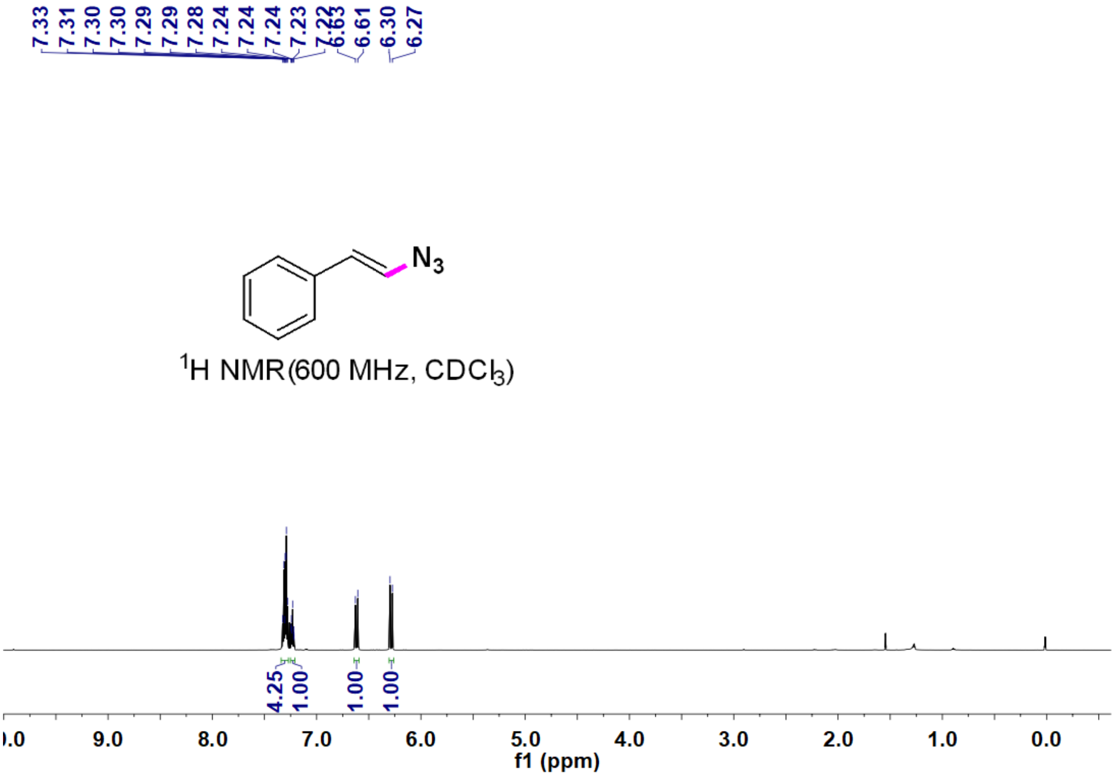

Supplementary Figure 106. Characterization of compound 10a. ^{1}H NMR spectrum of compound **10a** in $CDCl_3$.

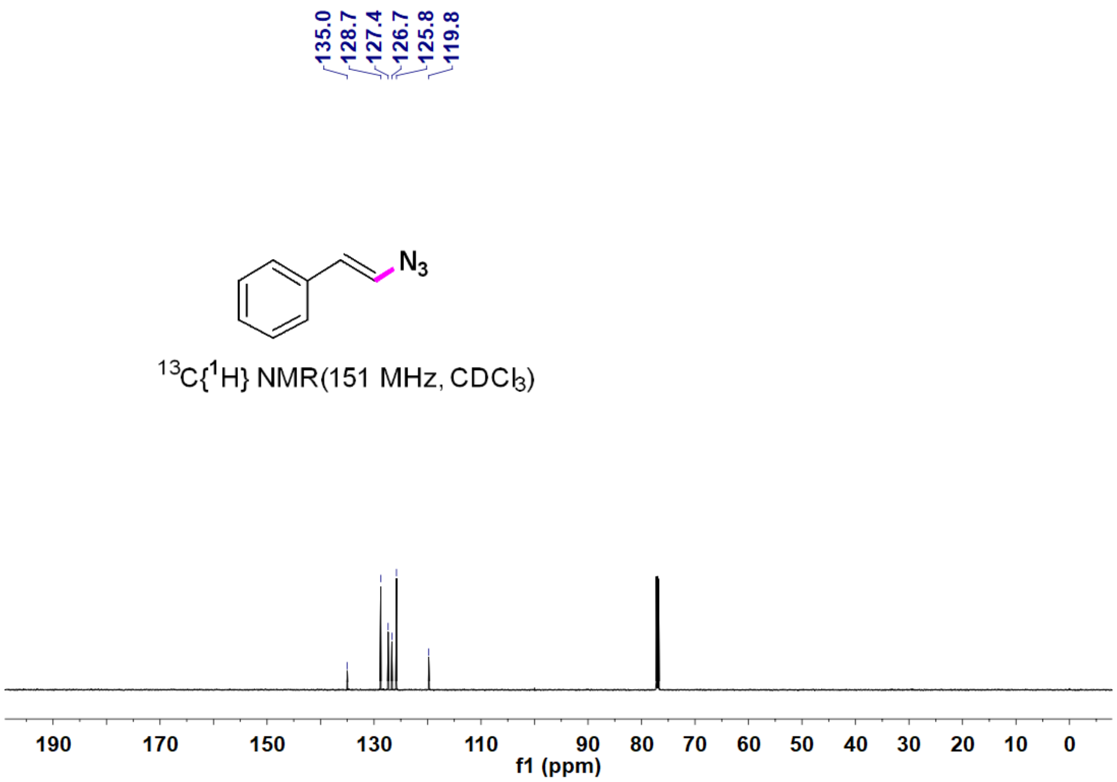

Supplementary Figure 107. Characterization of compound 10a. $^{13}C\{^{1}H\}$ NMR spectrum of compound **10a** in $CDCl_3$.

(E)-4-styrylbenzaldehyde (11a)

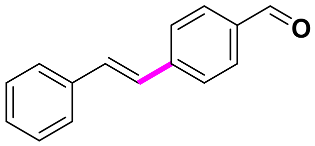

E-11a

Under N_2 atmosphere, the $Pd(PPh_3)_4$ (11.6 mg, 5.0 mol%), **E-4a** compound (46.0 mg, 0.2 mmol), Cs_2CO_3 (391.0 mg, 1.2 mmol), 4-bromobenzaldehyde (37.0 mg, 0.2 mmol) and the 4.0 mL THF solvent were added into 25 mL Schlenk tube to react at 60 °C overnight. The reactions were monitored by TLC. When **E-4a** compound was consumed, the reaction was cooled to room temperature, then quenched and concentrated. The crude products were then purified by column chromatography (PE-EA, v/v 20/1) to give the target products as a white solid with an overall isolated yield: 93% (38.7 mg). **^{1}H NMR** (600 MHz, $CDCl_3$) δ 10.00 (s, 1H), 7.87 (d, J = 8.1 Hz, 2H), 7.65 (d, J = 7.9 Hz, 2H), 7.55 (d, J = 7.5 Hz, 2H), 7.39 (t, J = 7.6 Hz, 2H), 7.32 (t, J = 7.3 Hz, 1H), 7.26 (d, J = 16.3 Hz, 1H), 7.14 (d, J = 16.3 Hz, 1H). **$^{13}C\{^{1}H\}$ NMR** (151 MHz, $CDCl_3$) δ 191.5, 143.4, 136.5, 135.3, 132.2, 130.2, 128.8, 128.5, 127.3, 126.9.

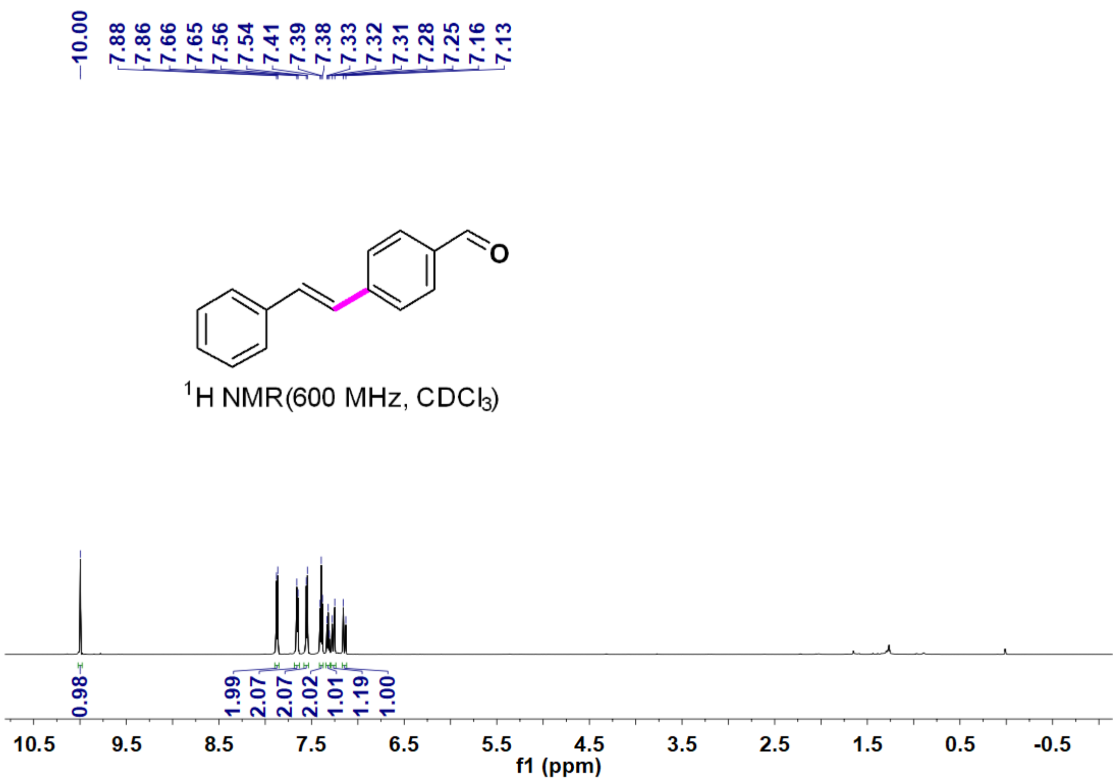

Supplementary Figure 108. Characterization of compound 11a. ^{1}H NMR spectrum of compound **11a** in $CDCl_3$.

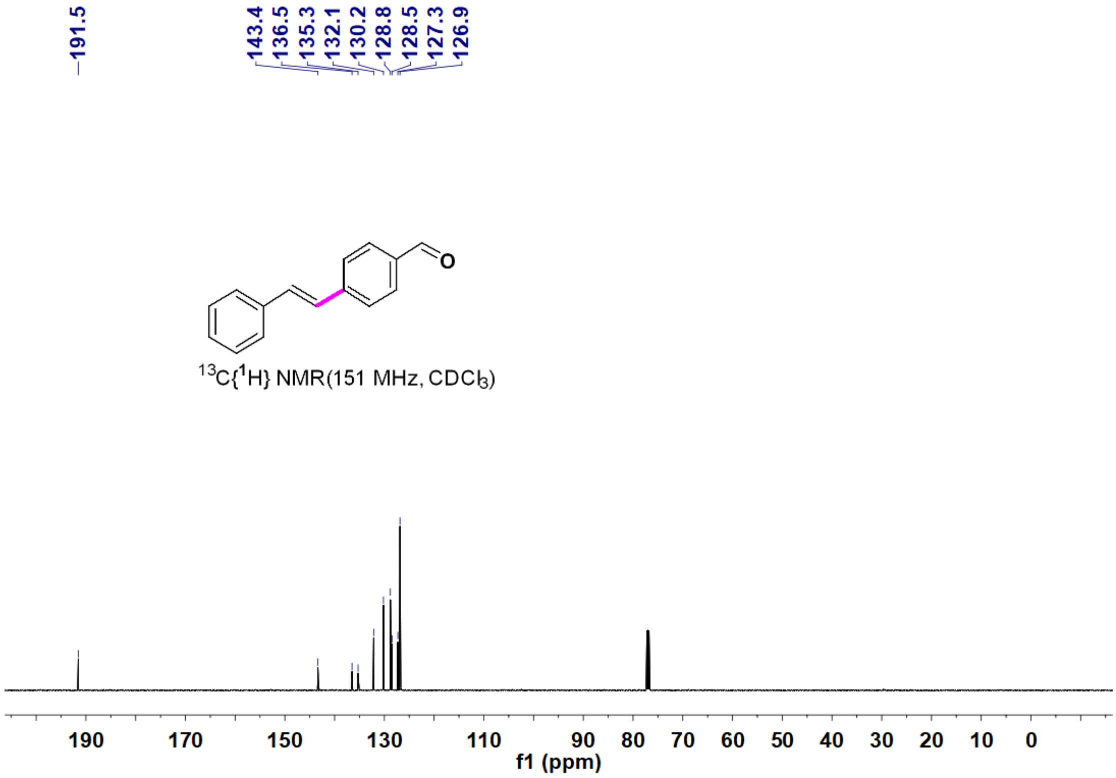

Supplementary Figure 109. Characterization of compound 11a. $^{13}C\{^{1}H\}$ NMR spectrum of compound **11a** in $CDCl_3$.

Tables of Crystal Data and Structure Refinements

Supplementary Table 4. Crystal datum and structure refinements for **Cu_4NC** and **Cu_8NC**.

	**Cu_4NC**	**Cu_8NC**
**Empirical formula**	$C_{16}H_{20}Cu_4N_8S_4$	$C_{32}H_{56}Cu_8N_8S_8$
**Formula weight**	706.80	3717.21
**Temperature/K**	200	200
**Crystal system**	tetragonal	monoclinic
**Space group**	$P4_2/n$	$C2/c$
***a*/Å**	10.01930(10)	15.47190(10)
***b*/Å**	10.01930(10)	21.4708(2)
***c*/Å**	11.6512(2)	14.65210(10)
***α*/°**	90	90
***β*/°**	90	90.3710(10)
***γ*/°**	90	90
**Volume/Å^3**	1169.62(3)	4867.24(6)
**Z**	2	4
***ρcalc*g/cm^3**	2.007	1.798
***μ*/mm^{-1}**	7.626	7.217
**F(000)**	704.0	2656.0
**Crystal size/mm^3**	0.13 × 0.12 × 0.1	0.08 × 0.07 × 0.06
**Radiation**	CuK*α* (*λ* = 1.54184 Å)	CuK*α* (*λ* = 1.54184 Å)
**2θrange for data collection/°**	11.648 to 147.848	7.042 to 147.194
**Index ranges**	-6 ≤ h ≤ 11, -11 ≤ k ≤ 12, -7 ≤ l ≤ 14	-18 ≤ h ≤ 17, -22 ≤ k ≤ 26, -17 ≤ l ≤ 18
**Reflections collected**	2629	12406
**Independent reflections**	1144 [R_{int} = 0.0166, R_{sigma} = 0.0218]	4687 [R_{int} = 0.0228, R_{sigma} = 0.0237]
**Data/restraints/parameters**	1144/0/74	4687/63/292
**Goodness-of-fit on F^2**	1.070	1.041
**Final R indexes [I>=2*σ* (I)]**	R_1 = 0.0331, wR_2 = 0.0946	R_1 = 0.0293, wR_2 = 0.0800
**CCDC number**	2330281	2330282

Compounds Characterization

(E)-4,4,5,5-tetramethyl-2-styryl-1,3,2-dioxaborolane (4a)[(28)]

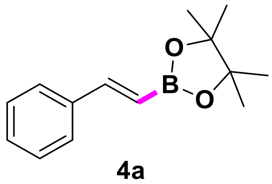

4a

Prepared according to general procedure (B), under air atmosphere, phenylacetylene **1a** (21.0 mg, 0.2 mmol, 1.0 eq), B_2Pin_2 **2** (111.8 mg, 0.44 mmol, 2.2 equiv.), microcrystalline Cu_4NC catalysts (2.8 mg, 2.0 mol%), K_2CO_3 (60.7 mg, 0.44 mmol, 2.2 equiv.) and the mixture solvent (2.0 mL, MeCN-H_2O, v/v 4/1) were added into a tube. The reaction mixture was stirred at room temperature for 1 h. The reactions were monitored by TLC (PE-EA, v/v 10/1). When alkynes were consumed, the reactions were quenched and concentrated. The crude product was then purified by column chromatography (PE-EA, v/v 20/1) to give the target product **4a** as a colorless oil with an overall isolated yield: 97% (44.6 mg). **^{1}H NMR** (600 MHz, $CDCl_3$) δ 7.49 (d, J = 7.2 Hz, 2H), 7.40 (d, J = 18.4 Hz, 1H), 7.34 (t, J = 7.4 Hz, 2H), 7.29 (ddd, J = 7.3, 3.6, 1.2 Hz, 1H), 6.17 (d, J = 18.4 Hz, 1H), 1.32 (s, 12H). **^{13}C{^{1}H} NMR** (151 MHz, $CDCl_3$) δ 149.5, 137.5, 128.9, 128.5, 127.0, 83.3, 24.8. The carbon signal attached to B was not observed due to low intensity.

(E)-4,4,5,5-tetramethyl-2-(4-methylstyryl)-1,3,2-dioxaborolane (4b)[(28)]

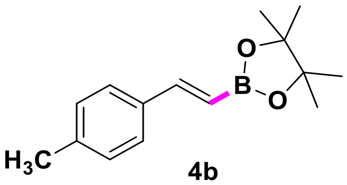

4b

Prepared according to general procedure (B), under air atmosphere, 4-ethynyltoluene **1b** (23.2 mg, 0.2 mmol, 1.0 eq), B_2Pin_2 **2** (111.8 mg, 0.44 mmol, 2.2 equiv.), microcrystalline Cu_4NC catalysts (2.8 mg, 2.0 mol%), K_2CO_3 (60.7 mg, 0.44 mmol, 2.2 equiv.) and the mixture solvent (2.0 mL, MeCN-H_2O, v/v 4/1) were added into a tube. The reaction mixture was stirred at room temperature for 1 h. The reactions were monitored by TLC (PE-EA, v/v 10/1). When alkynes were consumed, the reactions were quenched and concentrated. The crude product was then purified by column chromatography (PE-EA, v/v 20/1) to give the target product **4b** as a colorless oil with an overall isolated yield: 92% (44.8 mg). **^{1}H NMR** (600 MHz, $CDCl_3$) δ 7.38 (dd, J = 13.3, 7.1 Hz, 3H), 7.14 (d, J = 8.0 Hz, 2H), 6.11 (d, J = 18.4 Hz, 1H), 2.34 (s, 3H), 1.31 (s, 12H). **^{13}C{^{1}H} NMR** (151 MHz, $CDCl_3$) δ 149.5, 138.9, 134.8, 129.3, 127.0, 83.3, 24.8, 21.3. The carbon signal attached to B was not observed due to low intensity.

(E)-4,4,5,5-tetramethyl-2-(3-methylstyryl)-1,3,2-dioxaborolane (4c)[28]

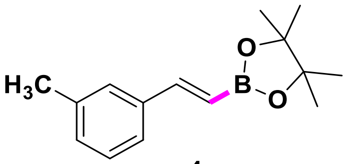

4c

Prepared according to general procedure (B), under air atmosphere, 3-ethynyltoluene **1c** (23.2 mg, 0.2 mmol, 1.0 eq), B_2Pin_2 **2** (111.8 mg, 0.44 mmol, 2.2 equiv.), microcrystalline Cu_4NC catalysts (2.8 mg, 2.0 mol%), K_2CO_3 (60.7 mg, 0.44 mmol, 2.2 equiv.) and the mixture solvent (2.0 mL, MeCN-H_2O, v/v 4/1) were added into a tube. The reaction mixture was stirred at room temperature for 1 h. The reactions were monitored by TLC (PE-EA, v/v 10/1). When alkynes were consumed, the reactions were quenched and concentrated. The crude product was then purified by column chromatography (PE-EA, v/v 20/1) to give the target product **4c** as a colorless oil with an overall isolated yield: 93% (45.3 mg). **^{1}H NMR** (600 MHz, $CDCl_3$) δ 7.37 (d, *J* = 18.4 Hz, 1H), 7.29 (d, *J* = 6.9 Hz, 2H), 7.22 (t, *J* = 7.9 Hz, 1H), 7.11 (d, *J* = 7.5 Hz, 1H), 6.15 (d, *J* = 18.4 Hz, 1H), 2.35 (s, 3H), 1.31 (s, 12H). **^{13}C{^{1}H} NMR** (151 MHz, $CDCl_3$) δ 149.7, 138.1, 137.5, 129.7, 128.4, 127.8, 124.2, 83.3, 24.8, 21.4. The carbon signal attached to B was not observed due to low intensity.

(E)-2-(2-methoxystyryl)-4,4,5,5-tetramethyl-1,3,2-dioxaborolane (4d)[26]

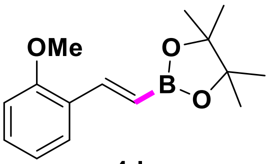

4d

Prepared according to general procedure (B), under air atmosphere, 1-ethynyl-2-methoxybenzene **1d** (26.4 mg, 0.2 mmol, 1.0 eq), B_2Pin_2 **2** (111.8 mg, 0.44 mmol, 2.2 equiv.), microcrystalline Cu_4NC catalysts (2.8 mg, 2.0 mol%), K_2CO_3 (60.7 mg, 0.44 mmol, 2.2 equiv.) and the mixture solvent (2.0 mL, MeCN-H_2O, v/v 4/1) were added into a tube. The reaction mixture was stirred at room temperature for 2 h. The reactions were monitored by TLC (PE-EA, v/v 20/1). When alkynes were consumed, the reactions were quenched and concentrated. The crude product was then purified by column chromatography (PE-EA, v/v 40/1) to give the target product **4d** as a colorless oil with an overall isolated yield: 90% (46.7 mg). **^{1}H NMR** (600 MHz, $CDCl_3$) δ 7.77 (d, *J* = 18.6 Hz, 1H), 7.55 (d, *J* = 7.6 Hz, 1H), 7.29 – 7.24 (m, 1H), 6.93 (t, *J* = 7.5 Hz, 1H), 6.87 (d, *J* = 8.3 Hz, 1H), 6.18 (d, *J* = 18.6 Hz, 1H), 3.85 (s, 3H), 1.31 (s, 12H). **^{13}C{^{1}H} NMR** (151 MHz, $CDCl_3$) δ 157.3, 144.1, 129.9, 127.1, 126.6, 120.6, 110.9, 83.2, 55.4, 24.8. The carbon signal attached to B was not observed due to low intensity.

(E)-2-(4-methoxystyryl)-4,4,5,5-tetramethyl-1,3,2-dioxaborolane (4e)[22]

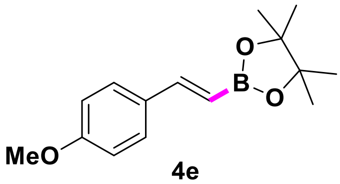

4e

Prepared according to general procedure (B), under air atmosphere, 1-ethynyl-4-methoxybenzene **1e** (26.4 mg, 0.2 mmol, 1.0 eq), B_2Pin_2 **2** (111.8 mg, 0.44 mmol, 2.2 equiv.), microcrystalline Cu_4NC catalysts (2.8 mg, 2.0 mol%), K_2CO_3 (60.7 mg, 0.44 mmol, 2.2 equiv.) and the mixture solvent (2.0 mL, MeCN-H_2O, v/v 4/1) were added into a tube. The reaction mixture was stirred at room temperature for 2 h. The reactions were monitored by TLC (PE-EA, v/v 20/1). When alkynes were consumed, the reactions were quenched and concentrated. The crude product was then purified by column chromatography (PE-EA, v/v 40/1) to give the target product **4e** as a colorless oil with an overall isolated yield: 93% (48.3 mg). **^{1}H NMR** (600 MHz, $CDCl_3$) δ 7.43 (d, J = 8.6 Hz, 2H), 7.35 (d, J = 18.4 Hz, 1H), 6.86 (d, J = 8.6 Hz, 2H), 6.01 (d, J = 18.4 Hz, 1H), 3.81 (s, 3H), 1.31 (s, 12H). **^{13}C{^{1}H} NMR** (151 MHz, $CDCl_3$) δ 160.3, 149.1, 130.4, 128.5, 114.0, 83.2, 55.3, 24.8. The carbon signal attached to B was not observed due to low intensity.

(E)-2-(4-(tert-butyl)styryl)-4,4,5,5-tetramethyl-1,3,2-dioxaborolane (4f)[24]

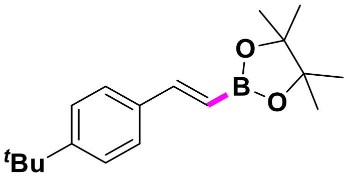

4f

Prepared according to general procedure (B), under air atmosphere, 1-(*tert*-butyl)-4-ethynylbenzene **1f** (31.6 mg, 0.2 mmol, 1.0 eq), B_2Pin_2 **2** (111.8 mg, 0.44 mmol, 2.2 equiv.), microcrystalline Cu_4NC catalysts (2.8 mg, 2.0 mol%), K_2CO_3 (60.7 mg, 0.44 mmol, 2.2 equiv.) and the mixture solvent (2.0 mL, MeCN-H_2O, v/v 4/1) were added into a tube. The reaction mixture was stirred at room temperature for 1 h. The reactions were monitored by TLC (PE-EA, v/v 20/1). When alkynes were consumed, the reactions were quenched and concentrated. The crude product was then purified by column chromatography (PE-EA, v/v 40/1) to give the target product **4f** as a white solid with an overall isolated yield: 92% (52.5 mg). **^{1}H NMR** (600 MHz, $CDCl_3$) δ 7.45 – 7.34 (m, 5H), 6.12 (d, J = 18.4 Hz, 1H), 1.31 (s, 21H). **^{13}C{^{1}H} NMR** (151 MHz, $CDCl_3$) δ 152.1, 149.4, 134.8, 126.8, 125.5, 83.3, 34.7, 31.2, 24.8. The carbon signal attached to B was not observed due to low intensity.

(E)-2-(2-fluorostyryl)-4,4,5,5-tetramethyl-1,3,2-dioxaborolane (4g)(26)

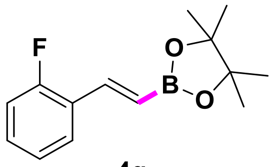

4g

Prepared according to general procedure (B), under air atmosphere, 1-ethynyl-2-fluorobenzene **1g** (24.0 mg, 0.2 mmol, 1.0 eq), B_2Pin_2 **2** (111.8 mg, 0.44 mmol, 2.2 equiv.), microcrystalline Cu_4NC catalysts (2.8 mg, 2.0 mol%), K_2CO_3 (60.7 mg, 0.44 mmol, 2.2 equiv.) and the mixture solvent (2.0 mL, MeCN-H_2O, v/v 4/1) were added into a tube. The reaction mixture was stirred at room temperature for 1 h. The reactions were monitored by TLC (PE-EA, v/v 10/1). When alkynes were consumed, the reactions were quenched and concentrated. The crude product was then purified by column chromatography (PE-EA, v/v 20/1) to give the target product **4g** as a colorless oil with an overall isolated yield: 93% (46.0 mg). **^{1}H NMR** (600 MHz, $CDCl_3$) δ 7.61 – 7.54 (m, 2H), 7.28 – 7.23 (m, 1H), 7.11 (t, *J* = 7.3 Hz, 1H), 7.03 (ddd, *J* = 10.5, 8.3, 0.9 Hz, 1H), 6.24 (d, *J* = 18.6 Hz, 1H), 1.31 (s, 12H). **^{13}C{^{1}H} NMR** (151 MHz, $CDCl_3$) δ 160.7 (d, *J* = 251.6 Hz), 141.3 (d, *J* = 4.0 Hz), 130.2 (d, *J* = 8.5 Hz), 127.4 (d, *J* = 3.4 Hz), 125.4, 124.1 (d, *J* = 3.5 Hz), 115.8 (d, *J* = 22.1 Hz), 83.5, 24.8. ^{19}F NMR (565 MHz, $CDCl_3$) δ -117.65. The carbon signal attached to B was not observed due to low intensity.

(E)-2-(4-fluorostyryl)-4,4,5,5-tetramethyl-1,3,2-dioxaborolane (4h)(27)

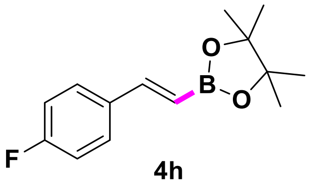

4h

Prepared according to general procedure (B), under air atmosphere, 1-ethynyl-4-fluorobenzene **1h** (24.0 mg, 0.2 mmol, 1.0 eq), B_2Pin_2 **2** (111.8 mg, 0.44 mmol, 2.2 equiv.), microcrystalline Cu_4NC catalysts (2.8 mg, 2.0 mol%), K_2CO_3 (60.7 mg, 0.44 mmol, 2.2 equiv.) and the mixture solvent (2.0 mL, MeCN-H_2O, v/v 4/1) were added into a tube. The reaction mixture was stirred at room temperature for 1 h. The reactions were monitored by TLC (PE-EA, v/v 10/1). When alkynes were consumed, the reactions were quenched and concentrated. The crude product was then purified by column chromatography (PE-EA, v/v 20/1) to give the target product **4h** as a colorless oil with an overall isolated yield: 92% (45.5 mg). **^{1}H NMR** (600 MHz, $CDCl_3$) δ 7.49 – 7.43 (m, 2H), 7.35 (d, *J* = 18.4 Hz, 1H), 7.02 (dd, *J* = 12.1, 5.3 Hz, 2H), 6.07 (d, *J* = 18.4 Hz, 1H), 1.31 (s, 12H). **^{13}C{^{1}H} NMR** (151 MHz, $CDCl_3$) δ 163.1 (d, *J* = 248.6 Hz), 148.2, 133.7 (d, *J* = 3.1 Hz), 128.7 (d, *J* = 8.3 Hz), 115.5 (d, *J* = 21.8 Hz), 83.4, 24.8. **^{19}F NMR** (565 MHz, $CDCl_3$) δ -112.40. The carbon signal attached to B was not observed due to low intensity.

(E)-2-(2-chlorostyryl)-4,4,5,5-tetramethyl-1,3,2-dioxaborolane (4i)[28]

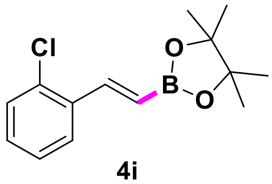

4i

Prepared according to general procedure (B), under air atmosphere, 1-chloro-2-ethynylbenzene **1i** (27.3 mg, 0.2 mmol, 1.0 eq), B_2Pin_2 **2** (111.8 mg, 0.44 mmol, 2.2 equiv.), microcrystalline Cu_4NC catalysts (2.8 mg, 2.0 mol%), K_2CO_3 (60.7 mg, 0.44 mmol, 2.2 equiv.) and the mixture solvent (2.0 mL, MeCN-H_2O, v/v 4/1) were added into a tube. The reaction mixture was stirred at room temperature for 1 h. The reactions were monitored by TLC (PE-EA, v/v 10/1). When alkynes were consumed, the reactions were quenched and concentrated. The crude product was then purified by column chromatography (PE-EA, v/v 20/1) to give the target product **4i** as a colorless oil with an overall isolated yield: 95% (50.0 mg). **^{1}H NMR** (600 MHz, $CDCl_3$) δ 7.78 (d, J = 18.3 Hz, 1H), 7.63 (dd, J = 7.4, 2.0 Hz, 1H), 7.35 (dd, J = 7.6, 1.6 Hz, 1H), 7.23 (pd, J = 7.3, 1.7 Hz, 2H), 6.17 (d, J = 18.3 Hz, 1H), 1.32 (s, 12H). **^{13}C{^{1}H} NMR** (151 MHz, $CDCl_3$) δ 145.0, 135.6, 133.9, 129.8, 129.6, 127.0, 126.8, 83.5, 24.8. The carbon signal attached to B was not observed due to low intensity.

(E)-2-(3-chlorostyryl)-4,4,5,5-tetramethyl-1,3,2-dioxaborolane (4j)[28]

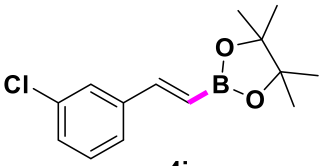

4j

Prepared according to general procedure (B), under air atmosphere, 3-chloro-2-ethynylbenzene **1j** (27.3 mg, 0.2 mmol, 1.0 eq), B_2Pin_2 **2** (111.8 mg, 0.44 mmol, 2.2 equiv.), microcrystalline Cu_4NC catalysts (2.8 mg, 2.0 mol%), K_2CO_3 (60.7 mg, 0.44 mmol, 2.2 equiv.) and the mixture solvent (2.0 mL, MeCN-H_2O, v/v 4/1) were added into a tube. The reaction mixture was stirred at room temperature for 1 h. The reactions were monitored by TLC (PE-EA, v/v 10/1). When alkynes were consumed, the reactions were quenched and concentrated. The crude product was then purified by column chromatography (PE-EA, v/v 20/1) to give the target product **4j** as a colorless oil with an overall isolated yield: 98% (51.5 mg). **^{1}H NMR** (600 MHz, $CDCl_3$) δ 7.45 (s, 1H), 7.37 – 7.29 (m, 2H), 7.28 – 7.25 (m, 2H), 6.17 (d, J = 18.4 Hz, 1H), 1.31 (s, 12H). **^{13}C{^{1}H} NMR** (151 MHz, $CDCl_3$) δ 147.8, 139.4, 134.6, 129.8, 128.7, 126.9, 125.2, 83.5, 24.8. The carbon signal attached to B was not observed due to low intensity.

(E)-2-(4-chlorostyryl)-4,4,5,5-tetramethyl-1,3,2-dioxaborolane (4k)[28]

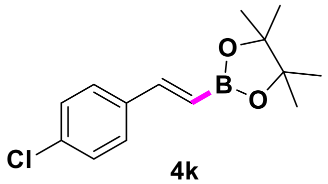

4k

Prepared according to general procedure (B), under air atmosphere, 4-chloro-2-ethynylbenzene **1k** (27.3 mg, 0.2 mmol, 1.0 eq), B_2Pin_2 **2** (111.8 mg, 0.44 mmol, 2.2 equiv.), microcrystalline Cu_4NC catalysts (2.8 mg, 2.0 mol%), K_2CO_3 (60.7 mg, 0.44 mmol, 2.2 equiv.) and the mixture solvent (2.0 mL, MeCN-H_2O, v/v 4/1) were added into a tube. The reaction mixture was stirred at room temperature for 1 h. The reactions were monitored by TLC (PE-EA, v/v 20/1). When alkynes were consumed, the reactions were quenched and concentrated. The crude product was then purified by column chromatography (PE-EA, v/v 40/1) to give the target product **4k** as a white solid with an overall isolated yield: 99% (52.1 mg). **^{1}H NMR** (600 MHz, $CDCl_3$) δ 7.41 (d, *J* = 8.5 Hz, 2H), 7.32 (dd, *J* = 19.5, 13.5 Hz, 3H), 6.13 (d, *J* = 18.4 Hz, 1H), 1.31 (s, 12H). **^{13}C{^{1}H} NMR** (151 MHz, $CDCl_3$) δ 148.0, 136.0, 134.6, 128.8, 128.2, 83.4, 24.8. The carbon signal attached to B was not observed due to low intensity.

(E)-2-(2-bromostyryl)-4,4,5,5-tetramethyl-1,3,2-dioxaborolane (4l)[23]

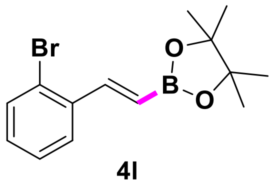

4l

Prepared according to general procedure (B), under air atmosphere, 1-bromo-2-ethynylbenzene **1l** (36.2 mg, 0.2 mmol, 1.0 eq), B_2Pin_2 **2** (111.8 mg, 0.44 mmol, 2.2 equiv.), microcrystalline Cu_4NC catalysts (2.8 mg, 2.0 mol%), K_2CO_3 (60.7 mg, 0.44 mmol, 2.2 equiv.) and the mixture solvent (2.0 mL, MeCN-H_2O, v/v 4/1) were added into a tube. The reaction mixture was stirred at room temperature for 1 h. The reactions were monitored by TLC (PE-EA, v/v 20/1). When alkynes were consumed, the reactions were quenched and concentrated. The crude product was then purified by column chromatography (PE-EA, v/v 40/1) to give the target product **4l** as a pale-yellow oil with an overall isolated yield: 95% (58.6 mg). **^{1}H NMR** (600 MHz, $CDCl_3$) δ 7.71 (d, *J* = 18.3 Hz, 1H), 7.61 (s, 1H), 7.55 (dd, *J* = 8.0, 1.1 Hz, 1H), 7.29 (dd, *J* = 11.3, 3.9 Hz, 1H), 7.14 (td, *J* = 7.9, 1.6 Hz, 1H), 6.12 (d, *J* = 18.2 Hz, 1H), 1.32 (s, 12H). **^{13}C{^{1}H} NMR** (151 MHz, $CDCl_3$) δ 147.5, 137.4, 133.0, 129.9, 127.5, 127.3, 124.3, 83.5, 24.8. The carbon signal attached to B was not observed due to low intensity.

(E)-2-(4-bromostyryl)-4,4,5,5-tetramethyl-1,3,2-dioxaborolane (4m)[(28)]

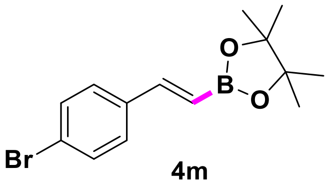

4m

Prepared according to general procedure (B), under air atmosphere, 4-bromo-2-ethynylbenzene **1m** (36.2 mg, 0.2 mmol, 1.0 eq), B_2Pin_2 **2** (111.8 mg, 0.44 mmol, 2.2 equiv.), microcrystalline Cu_4NC catalysts (2.8 mg, 2.0 mol%), K_2CO_3 (60.7 mg, 0.44 mmol, 2.2 equiv.) and the mixture solvent (2.0 mL, MeCN-H_2O, v/v 4/1) were added into a tube. The reaction mixture was stirred at room temperature for 1 h. The reactions were monitored by TLC (PE-EA, v/v 20/1). When alkynes were consumed, the reactions were quenched and concentrated. The crude product was then purified by column chromatography (PE-EA, v/v 40/1) to give the target product **4m** as a white solid with an overall isolated yield: 96% (59.0 mg). **^{1}H NMR** (600 MHz, $CDCl_3$) δ 7.46 (d, *J* = 8.4 Hz, 2H), 7.33 (t, *J* = 13.8 Hz, 3H), 6.15 (d, *J* = 18.4 Hz, 1H), 1.31 (s, 12H). **^{13}C{^{1}H} NMR** (151 MHz, $CDCl_3$) δ 148.1, 136.4, 131.7, 128.5, 122.9, 83.5, 24.8. The carbon signal attached to B was not observed due to low intensity.

(E)-2-(3-bromostyryl)-4,4,5,5-tetramethyl-1,3,2-dioxaborolane (4n)[(23)]

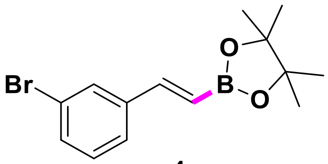

4n

Prepared according to general procedure (B), under air atmosphere, 3-bromo-2-ethynylbenzene **1n** (36.2 mg, 0.2 mmol, 1.0 eq), B_2Pin_2 **2** (111.8 mg, 0.44 mmol, 2.2 equiv.), microcrystalline Cu_4NC catalysts (2.8 mg, 2.0 mol%), K_2CO_3 (60.7 mg, 0.44 mmol, 2.2 equiv.) and the mixture solvent (2.0 mL, MeCN-H_2O, v/v 4/1) were added into a tube. The reaction mixture was stirred at room temperature for 1 h. The reactions were monitored by TLC (PE-EA, v/v 10/1). When alkynes were consumed, the reactions were quenched and concentrated. The crude product was then purified by column chromatography (PE-EA, v/v 20/1) to give the target product **4n** as a colorless oil with an overall isolated yield: 94% (57.7 mg). **^{1}H NMR** (600 MHz, $CDCl_3$) δ 7.61 (t, *J* = 1.7 Hz, 1H), 7.43 – 7.37 (m, 2H), 7.30 (d, *J* = 18.4 Hz, 1H), 7.20 (t, *J* = 7.8 Hz, 1H), 6.15 (d, *J* = 18.4 Hz, 1H), 1.31 (s, 12H). **^{13}C{^{1}H} NMR** (151 MHz, $CDCl_3$) δ 147.7, 139.6, 131.6, 130.1, 129.9, 125.6, 122.8, 83.5, 24.8. The carbon signal attached to B was not observed due to low intensity.

(E)-4,4,5,5-tetramethyl-2-(4-(trifluoromethyl)styryl)-1,3,2-dioxaborolane (4o)[22]

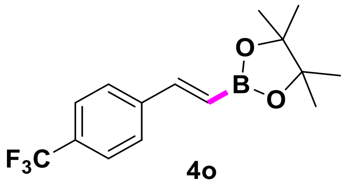

4o

Prepared according to general procedure (B), under air atmosphere, 1-ethynyl-4-(trifluoromethyl) benzene **1o** (34.0 mg, 0.2 mmol, 1.0 eq), B_2Pin_2 **2** (111.8 mg, 0.44 mmol, 2.2 equiv.), microcrystalline Cu_4NC catalysts (2.8 mg, 2.0 mol%), K_2CO_3 (60.7 mg, 0.44 mmol, 2.2 equiv.) and the mixture solvent (2.0 mL, MeCN-H_2O, v/v 4/1) were added into a tube. The reaction mixture was stirred at room temperature for 1 h. The reactions were monitored by TLC (PE-EA, v/v 10/1). When alkynes were consumed, the reactions were quenched and concentrated. The crude product was then purified by column chromatography (PE-EA, v/v 20/1) to give the target product **4o** as a colorless oil with an overall isolated yield: 96% (57.3 mg). **^{1}H NMR** (600 MHz, $CDCl_3$) δ 7.58 (q, J = 8.6 Hz, 4H), 7.40 (d, J = 18.4 Hz, 1H), 6.26 (d, J = 18.4 Hz, 1H), 1.32 (s, 12H). **^{13}C{^{1}H} NMR** (151 MHz, $CDCl_3$) δ 147.7, 140.8, 130.5 (d, J = 32.3 Hz), 127.1, 125.6 (q, J = 3.8 Hz), 83.6, 24.8. ^{19}F NMR (565 MHz, $CDCl_3$) δ -62.64. The carbon signal attached to B was not observed due to low intensity.

(E)-4-(2-(4,4,5,5-tetramethyl-1,3,2-dioxaborolan-2-yl)vinyl)benzonitrile (4p)[23]

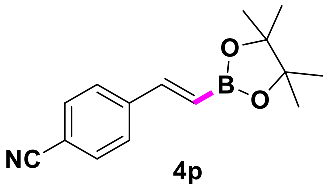

4p

Prepared according to general procedure (B), under air atmosphere, 4-ethynylbenzonitrile **1p** (25.4 mg, 0.2 mmol, 1.0 eq), B_2Pin_2 **2** (111.8 mg, 0.44 mmol, 2.2 equiv.), microcrystalline Cu_4NC catalysts (2.8 mg, 2.0 mol%), K_2CO_3 (60.7 mg, 0.44 mmol, 2.2 equiv.) and the mixture solvent (2.0 mL, MeCN-H_2O, v/v 4/1) were added into a tube. The reaction mixture was stirred at room temperature for 1 h. The reactions were monitored by TLC (PE-EA, v/v 10/1). When alkynes were consumed, the reactions were quenched and concentrated. The crude product was then purified by column chromatography (PE-EA, v/v 1/0) to give the target product **4p** as a white solid with an overall isolated yield: 92% (46.7 mg). **^{1}H NMR** (600 MHz, $CDCl_3$) δ 7.61 (d, J = 8.1 Hz, 2H), 7.54 (d, J = 8.2 Hz, 2H), 7.35 (d, J = 18.4 Hz, 1H), 6.27 (d, J = 18.4 Hz, 1H), 1.31 (s, 12H). **^{13}C{^{1}H} NMR** (151 MHz, $CDCl_3$) δ 147.1, 141.6, 132.4, 127.4, 118.7, 112.0, 83.7, 24.8. The carbon signal attached to B was not observed due to low intensity.

(E)-3-(2-(4,4,5,5-tetramethyl-1,3,2-dioxaborolan-2-yl)vinyl)benzaldehyde (4q)

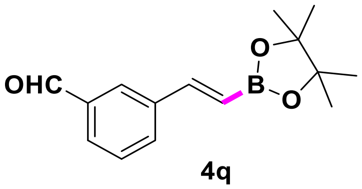

4q

Prepared according to general procedure (B), under air atmosphere, 3-ethynylbenzaldehyde **1q** (26.0 mg, 0.2 mmol, 1.0 eq), B_2Pin_2 **2** (111.8 mg, 0.44 mmol, 2.2 equiv.), microcrystalline Cu_4NC catalysts (2.8 mg, 2.0 mol%), K_2CO_3 (60.7 mg, 0.44 mmol, 2.2 equiv.) and the mixture solvent (2.0 mL, MeCN-H_2O, v/v 4/1) were added into a tube. The reaction mixture was stirred at room temperature for 1 h. The reactions were monitored by TLC (PE-EA, v/v 20/1). When alkynes were consumed, the reactions were quenched and concentrated. The crude product was then purified by column chromatography (PE-EA, v/v 40/1) to give the target product **4q** was purified by flash chromatography (PE-EA, v/v 40/1) as a colorless oil with an overall isolated yield: 90% (46.5 mg). **^{1}H NMR** (600 MHz, $CDCl_3$) δ 10.02 (s, 1H), 7.97 (s, 1H), 7.81 (d, *J* = 7.5 Hz, 1H), 7.73 (d, *J* = 7.6 Hz, 1H), 7.51 (t, *J* = 7.6 Hz, 1H), 7.43 (d, *J* = 18.4 Hz, 1H), 6.26 (d, *J* = 18.4 Hz, 1H), 1.31 (d, *J* = 7.2 Hz, 12H). **^{13}C{^{1}H} NMR** (151 MHz, $CDCl_3$) δ 192.1, 147.7, 138.5, 136.8, 132.6, 129.6, 129.3, 128.3, 83.6, 24.8. ^{11}B**{^{1}H}** NMR (193 MHz, $CDCl_3$) δ 30.64. **HRMS (ESI)** m/z: $[M+H]^+$ calcd for $C_{15}H_{20}BO_3^+$ 259.1500; found 259.1471. The carbon signal attached to B was not observed due to low intensity.

(E)-2-(2-([1,1'-biphenyl]-4-yl)vinyl)-4,4,5,5-tetramethyl-1,3,2-dioxaborolane (4r)[22]

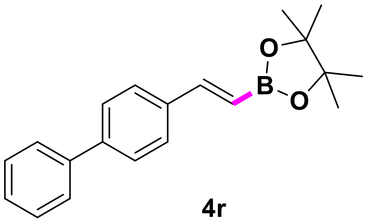

4r

Prepared according to general procedure (B), under air atmosphere, 4-ethynyl-1,1'-biphenyl **1r** (35.6 mg, 0.2 mmol, 1.0 eq), B_2Pin_2 **2** (111.8 mg, 0.44 mmol, 2.2 equiv.), microcrystalline Cu_4NC catalysts (2.8 mg, 2.0 mol%), K_2CO_3 (60.7 mg, 0.44 mmol, 2.2 equiv.) and the mixture solvent (2.0 mL, MeCN-H_2O, v/v 4/1) were added into a tube. The reaction mixture was stirred at room temperature for 1 h. The reactions were monitored by TLC (PE-EA, v/v 20/1). When alkynes were consumed, the reactions were quenched and concentrated. The crude product was then purified by column chromatography (PE-EA, v/v 40/1) to give the target product **4r** as a pare-yellow solid with an overall isolated yield: 99% (60.4 mg). **^{1}H NMR** (600 MHz, $CDCl_3$) δ 7.59 (dt, *J* = 12.9, 7.9 Hz, 6H), 7.47 – 7.42 (m, 3H), 7.35 (t, *J* = 7.4 Hz, 1H), 6.21 (dd, *J* = 18.4, 1.2 Hz, 1H), 1.33 (s, 12H). **^{13}C{^{1}H} NMR** (151 MHz, $CDCl_3$) δ 149.0, 141.6, 140.6,

136.5, 128.8, 127.5, 127.4, 127.3, 127.0, 83.4, 24.8. The carbon signal attached to B was not observed due to low intensity.

(E)-4-(2-(4,4,5,5-tetramethyl-1,3,2-dioxaborolan-2-yl)vinyl)pyridine (4s)[(21)]

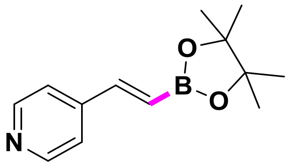

4s

Prepared according to general procedure (B), under air atmosphere, 4-ethynylpyridine **1s** (20.6 mg, 0.2 mmol, 1.0 eq), B_2Pin_2 **2** (111.8 mg, 0.44 mmol, 2.2 equiv.), microcrystalline Cu_4NC catalysts (2.8 mg, 2.0 mol%), K_2CO_3 (60.7 mg, 0.44 mmol, 2.2 equiv.) and the mixture solvent (2.0 mL, MeCN-H_2O, v/v 4/1) were added into a tube. The reaction mixture was stirred at room temperature for 1 h. The reactions were monitored by TLC (DCM-EA, v/v 3/1). When alkynes were consumed, the reactions were quenched and concentrated. The crude product was then purified by column chromatography (DCM-EA, v/v 5/1) to give the target product **4s** as a pare-yellow solid with an overall isolated yield: 86% (39.8 mg). **^{1}H NMR** (600 MHz, $CDCl_3$) δ 8.59 (d, J = 4.4 Hz, 2H), 7.36 – 7.27 (m, 3H), 6.38 (d, J = 18.4 Hz, 1H), 1.32 (s, 12H). **^{13}C{^{1}H} NMR** (151 MHz, $CDCl_3$) δ 150.0, 146.5, 144.8, 121.2, 83.8, 24.8. The carbon signal attached to B was not observed due to low intensity.

(E)-4,4,5,5-tetramethyl-2-(2-(thiophen-3-yl)vinyl)-1,3,2-dioxaborolane (4t)[(24)]

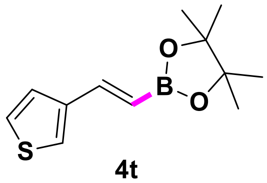

4t

Prepared according to general procedure (B), under air atmosphere, 3-ethynylthiophene **1t** (21.6 mg, 0.2 mmol, 1.0 eq), B_2Pin_2 **2** (111.8 mg, 0.44 mmol, 2.2 equiv.), microcrystalline Cu_4NC catalysts (2.8 mg, 2.0 mol%), K_2CO_3 (60.7 mg, 0.44 mmol, 2.2 equiv.) and the mixture solvent (2.0 mL, MeCN-H_2O, v/v 4/1) were added into a tube. The reaction mixture was stirred at room temperature for 1 h. The reactions were monitored by TLC (PE-EA, v/v 20/1). When alkynes were consumed, the reactions were quenched and concentrated. The crude product was then purified by column chromatography (PE-EA, v/v 40/1) to give the target product **4t** as a colorless oil with an overall isolated yield: 95% (44.7 mg). **^{1}H NMR** (600 MHz, $CDCl_3$) δ 7.38 (d, J = 18.4 Hz, 1H), 7.32 – 7.25 (m, 3H), 5.94 (d, J = 18.3 Hz, 1H), 1.30 (s, 12H). **^{13}C{^{1}H} NMR** (151 MHz, $CDCl_3$) δ 143.1, 141.2, 126.1, 125.0, 124.8, 83.3, 24.8. The carbon signal attached to B was not observed due to low intensity.

(E)-4,4,5,5-tetramethyl-2-(2-(thiophen-2-yl)vinyl)-1,3,2-dioxaborolane (4u)[(23)]

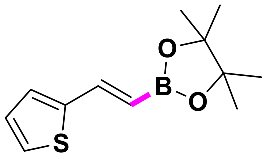

4u

Prepared according to general procedure (B), under air atmosphere, 2-ethynylthiophene **1u** (21.6 mg, 0.2 mmol, 1.0 eq), B_2Pin_2 **2** (111.8 mg, 0.44 mmol, 2.2 equiv.), microcrystalline Cu_4NC catalysts (2.8 mg, 2.0 mol%), K_2CO_3 (60.7 mg, 0.44 mmol, 2.2 equiv.) and the mixture solvent (2.0 mL, MeCN-H_2O, v/v 4/1) were added into a tube. The reaction mixture was stirred at room temperature for 1 h. The reactions were monitored by TLC (PE-EA, v/v 20/1). When alkynes were consumed, the reactions were quenched and concentrated. The crude product was then purified by column chromatography (PE-EA, v/v 40/1) to give the target product **4u** as a yellow oil with an overall isolated yield: 97% (45.8 mg). **^{1}H NMR** (600 MHz, $CDCl_3$) δ 7.47 (d, *J* = 18.0 Hz, 1H), 7.24 (d, *J* = 5.0 Hz, 1H), 7.08 (d, *J* = 3.4 Hz, 1H), 6.99 (dd, *J* = 5.0, 3.6 Hz, 1H), 5.91 (d, *J* = 18.1 Hz, 1H), 1.30 (s, 12H). **^{13}C{^{1}H} NMR** (151 MHz, $CDCl_3$) δ 143.9, 141.8, 127.7, 127.6, 126.3, 83.4, 24.8. The carbon signal attached to B was not observed due to low intensity.

(E)-6-(2-(4,4,5,5-tetramethyl-1,3,2-dioxaborolan-2-yl)vinyl)quinoline (4v)

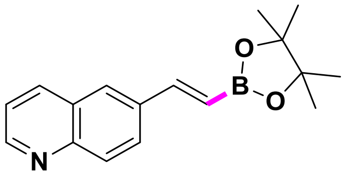

4v

Prepared according to general procedure (B), under air atmosphere, 6-ethynylquinoline **1v** (30.6 mg, 0.2 mmol, 1.0 eq), B_2Pin_2 **2** (111.8 mg, 0.44 mmol, 2.2 equiv.), microcrystalline Cu_4NC catalysts (2.8 mg, 2.0 mol%), K_2CO_3 (60.7 mg, 0.44 mmol, 2.2 equiv.) and the mixture solvent (2.0 mL, MeCN-H_2O, v/v 4/1) were added into a tube. The reaction mixture was stirred at room temperature for 1 h. The reactions were monitored by TLC (PE-EA, v/v 1/1). When alkynes were consumed, the reactions were quenched and concentrated. The crude product was then purified by column chromatography (PE-EA, v/v 2/1) to give the target product **4v** as a colorless oil with an overall isolated yield: 96% (53.8 mg). **^{1}H NMR** (600 MHz, $CDCl_3$) δ 8.89 – 8.84 (m, 1H), 8.12 (d, *J* = 7.9 Hz, 1H), 8.05 (d, *J* = 8.8 Hz, 1H), 7.91 (d, *J* = 8.8 Hz, 1H), 7.79 (s, 1H), 7.55 (d, *J* = 18.4 Hz, 1H), 7.37 (dd, *J* = 8.1, 4.2 Hz, 1H), 6.31 (d, *J* = 18.4 Hz, 1H), 1.32 (s, 12H). **^{13}C{^{1}H} NMR** (151 MHz, $CDCl_3$) δ 150.6, 148.6, 148.4, 136.3, 135.6, 129.8, 128.3, 127.2, 127.1, 121.4, 83.5, 24.8. **^{11}B{^{1}H} NMR** (193 MHz, $CDCl_3$) δ 31.04. **HRMS (ESI)** m/z: $[M+H]^+$ calcd for $C_{17}H_{21}BNO_2^+$ 282.1660; found 282.1662. The carbon signal attached to B was not observed due to low intensity.

(E)-4,4,5,5-tetramethyl-2-(2-(naphthalen-2-yl)vinyl)-1,3,2-dioxaborolane (4w)[23]

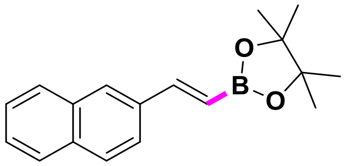

4w

Prepared according to general procedure (B), under air atmosphere, 2-ethynylnaphthalene **1w** (30.4 mg, 0.2 mmol, 1.0 eq), B_2Pin_2 **2** (111.8 mg, 0.44 mmol, 2.2 equiv.), microcrystalline Cu_4NC catalysts (2.8 mg, 2.0 mol%), K_2CO_3 (60.7 mg, 0.44 mmol, 2.2 equiv.) and the mixture solvent (2.0 mL, MeCN-H_2O, v/v 4/1) were added into a tube. The reaction mixture was stirred at room temperature for 1 h. The reactions were monitored by TLC (PE-EA, v/v 20/1). When alkynes were consumed, the reactions were quenched and concentrated. The crude product was then purified by column chromatography (PE-EA, v/v 40/1) to give the target product **4w** as a colorless oil with an overall isolated yield: 95% (53.3 mg). **^{1}H NMR** (600 MHz, $CDCl_3$) δ 7.86 – 7.77 (m, 4H), 7.70 (dd, *J* = 8.6, 1.6 Hz, 1H), 7.57 (d, *J* = 18.4 Hz, 1H), 7.50 – 7.43 (m, 2H), 6.29 (d, *J* = 18.4 Hz, 1H), 1.34 (s, 12H). **^{13}C{^{1}H} NMR** (151 MHz, $CDCl_3$) δ 149.5, 135.0, 133.7, 133.4, 128.4, 128.2, 128.0, 127.7, 126.4, 126.3, 123.4, 83.4, 24.8. The carbon signal attached to B was not observed due to low intensity.

(E)-4,4,5,5-tetramethyl-2-(2-(phenanthren-9-yl)vinyl)-1,3,2-dioxaborolane (4x)[26]

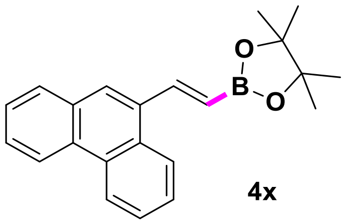

4x

Prepared according to general procedure (B), under air atmosphere, 9-ethynylphenanthrene **1x** (40.4 mg, 0.2 mmol, 1.0 eq), B_2Pin_2 **2** (111.8 mg, 0.44 mmol, 2.2 equiv.), microcrystalline Cu_4NC catalysts (2.8 mg, 2.0 mol%), K_2CO_3 (60.7 mg, 0.44 mmol, 2.2 equiv.) and the mixture solvent (2.0 mL, MeCN-H_2O, v/v 4/1) were added into a tube. The reaction mixture was stirred at room temperature for 1 h. The reactions were monitored by TLC (PE-EA, v/v 20/1). When alkynes were consumed, the reactions were quenched and concentrated. The crude product was then purified by column chromatography (PE-EA, v/v 1/0) to give the target product **4x** as a yellow solid with an overall isolated yield: 80% (52.7 mg). **^{1}H NMR** (600 MHz, $CDCl_3$) δ 8.72 (d, *J* = 7.9 Hz, 1H), 8.66 (d, *J* = 8.2 Hz, 1H), 8.31 – 8.28 (m, 1H), 8.21 (d, *J* = 17.9 Hz, 1H), 7.96 (s, 1H), 7.90 (d, *J* = 7.2 Hz, 1H), 7.65 (dddd, *J* = 9.5, 7.0, 3.2, 1.4 Hz, 3H), 7.61 – 7.57 (m, 1H), 6.35 (d, *J* = 18.0 Hz, 1H), 1.38 (s, 12H). **^{13}C{^{1}H} NMR** (151 MHz, $CDCl_3$) δ 147.2, 134.6, 131.6, 130.6, 130.3, 130.3, 129.0, 126.9, 126.7, 126.6, 126.5,

125.3, 124.7, 123.0, 122.5, 83.5, 24.9. The carbon signal attached to B was not observed due to low intensity.

(E)-4,4,5,5-tetramethyl-2-vinylferrocene-1,3,2-dioxaborolane (4y)

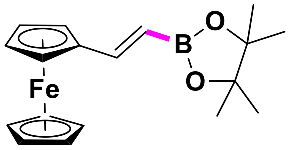

4y

Prepared according to general procedure (B), under air atmosphere, ethynylferrocene **1y** (42.0 mg, 0.2 mmol, 1.0 eq), B_2Pin_2 **2** (111.8 mg, 0.44 mmol, 2.2 equiv.), microcrystalline Cu_4NC catalysts (2.8 mg, 2.0 mol%), K_2CO_3 (60.7 mg, 0.44 mmol, 2.2 equiv.) and the mixture solvent (2.0 mL, MeCN-H_2O, v/v 4/1) were added into a tube. The reaction mixture was stirred at room temperature for 2 h. The reactions were monitored by TLC (PE-EA, v/v 20/1). When alkynes were consumed, the reactions were quenched and concentrated. The crude product was then purified by column chromatography (PE-EA, v/v 1/0) to give the target product **4y** as an orange solid with an overall isolated yield: 95% (64.1 mg). **^{1}H NMR** (600 MHz, $CDCl_3$) δ 7.14 (d, *J* = 18.2 Hz, 1H), 5.65 (d, *J* = 18.2 Hz, 1H), 4.38 – 4.34 (m, 2H), 4.23 – 4.19 (m, 2H), 4.03 (s, 5H), 1.23 (s, 12H). **^{13}C{^{1}H} NMR** (151 MHz, $CDCl_3$) δ 149.4, 83.1, 82.8, 69.6, 69.4, 67.6, 24.8. **^{11}B{^{1}H} NMR** (193 MHz, $CDCl_3$) δ 30.82. **HRMS(ESI)** m/z: $[M+H]^+$ calcd for $C_{18}H_{24}BFeO_2^+$ 339.1213. found: 339.1229. The carbon signal attached to B was not observed due to low intensity.

4,4'-bis((E)-2-(4,4,5,5-tetramethyl-1,3,2-dioxaborolan-2-yl)vinyl)-1,1'-biphenyl (4z)

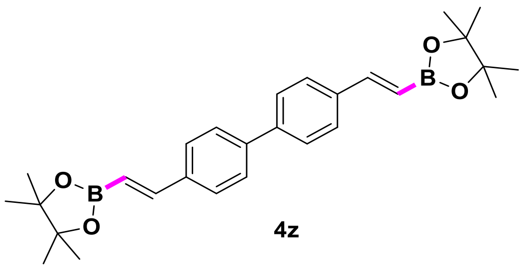

4z

Prepared according to general procedure (G), under air atmosphere, 4,4'-diethynyl-1,1'-biphenyl **1z** (20.2 mg, 0.1 mmol, 1.0 eq), B_2Pin_2 2 (111.8 mg, 0.44 mmol, 2.2 equiv.), microcrystalline Cu_4NC catalysts (2.8 mg, 2.0 mol%), K_2CO_3 (60.7 mg, 0.44 mmol, 2.2 equiv.) and the mixture solvent (2.0 mL, MeCN-H_2O, v/v 4/1) were added into a tube. The reaction mixture was stirred at room temperature for 1 h. The reactions were monitored by TLC (PE-EA, v/v 20/1). When alkynes were consumed, the reaction was quenched and concentrated. The crude products were then purified by column chromatography to give product **4z** as a yellow solid with an overall isolated yield: 93% (42.7 mg). **^{1}H NMR** (600 MHz, $CDCl_3$) δ 7.57 (dd, *J* = 20.5, 8.3 Hz, 8H), 7.43 (d, *J* =

18.4 Hz, 2H), 6.21 (d, *J* = 18.4 Hz, 2H), 1.32 (s, 24H). **^{13}C{^{1}H} NMR** (151 MHz, $CDCl_3$) δ 148.9, 140.9, 136.7, 127.6, 127.1, 83.4, 24.8. **^{11}B{^{1}H} NMR** (193 MHz, $CDCl_3$) δ 31.23. **HRMS** (ESI) m/z: $[M+H]^+$ calcd for $C_{28}H_{37}B_2O_4^+$ 459.2872; found 459.2875. The carbon signal attached to B was not observed due to low intensity.

(8R,9S,13S,14S,17R)-13-methyl-17-((E)-2-(4,4,5,5-tetramethyl-1,3,2-dioxaborolan-2-yl)vinyl)-7,8,9,11,12,13,14,15,16,17-decahydro-6H-cyclopenta[a]phenanthrene-3,17-diol (4aa)[25]

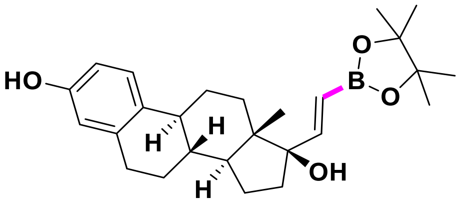

4aa

Prepared according to general procedure (B), under air atmosphere, (8*R*,9*S*,13*S*,14*S*,17*R*)-17-ethynyl-13-methyl-7,8,9,11,12,13,14,15,16,17-decahydro-6H-cyclopenta[*a*]phenanthrene-3,17-diol **1aa** (59.3 mg, 0.2 mmol, 1.0 eq), B_2Pin_2 **2** (111.8 mg, 0.44 mmol, 2.2 equiv.), microcrystalline Cu_4NC catalysts (2.8 mg, 2.0 mol%), K_2CO_3 (60.7 mg, 0.44 mmol, 2.2 equiv.) and the mixture solvent (2.0 mL, MeCN-H_2O, v/v 4/1) were added into a tube. The reaction mixture was stirred at room temperature for 1 h. The reactions were monitored by TLC (PE-EA, v/v 2/1). When alkynes were consumed, the reactions were quenched and concentrated. The crude product was then purified by column chromatography (PE-EA, v/v 7/3) to give the target product **4aa** as a white solid with an overall isolated yield: 85% (71.9 mg). **^{1}H NMR** (600 MHz, $CDCl_3$) δ 7.39 (s, 1H), 6.95 (dd, *J* = 17.6, 13.5 Hz, 2H), 6.63 – 6.55 (m, 2H), 5.68 (d, *J* = 18.2 Hz, 1H), 2.80 – 2.65 (m, 2H), 2.00 – 1.62 (m, 6H), 1.36 (d, *J* = 22.0 Hz, 12H), 1.21 (ddd, *J* = 37.8, 26.9, 16.5 Hz, 8H), 0.95 – 0.85 (m, 3H). **^{13}C{^{1}H} NMR** (151 MHz, $CDCl_3$) δ 158.7, 154.1, 138.3, 132.3, 125.8, 115.4, 112.0, 84.9, 84.0, 49.1, 47.1, 42.1, 38.8, 37.1, 32.2, 29.6, 27.1, 25.7, 24.8, 24.6, 23.3, 14.1. The carbon signal attached to B was not observed due to low intensity.

(E)-4,4,5,5-tetramethyl-2-(4-vinylstyryl)-1,3,2-dioxaborolane (4ab)[20]

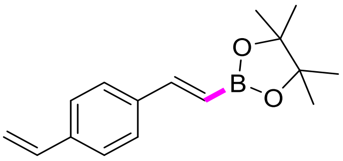

4ab

Prepared according to general procedure (B), under air atmosphere, 1-ethynyl-4-vinylbenzene **1ab** (25.6 mg, 0.2 mmol, 1.0 eq), B_2Pin_2 **2** (111.8 mg, 0.44 mmol, 2.2 equiv.), microcrystalline Cu_4NC catalysts (2.8 mg, 2.0 mol%), K_2CO_3 (60.7 mg, 0.44 mmol, 2.2 equiv.) and the mixture solvent (2.0 mL, MeCN-H_2O, v/v 4/1) were added into a tube. The reaction mixture was stirred at room temperature for 1 h. The reactions

were monitored by TLC (PE-EA, v/v 5/1). When alkynes were consumed, the reactions were quenched and concentrated. The crude product was then purified by column chromatography (PE-EA, v/v 10/0) to give the target product **4ab** as a yellow oil with an overall isolated yield: 95% (48.7 mg). **^{1}H NMR** (600 MHz, $CDCl_3$) δ 7.45 (d, *J* = 8.2 Hz, 2H), 7.38 (dd, *J* = 13.3, 5.1 Hz, 3H), 6.70 (dd, *J* = 17.6, 10.9 Hz, 1H), 6.16 (d, *J* = 18.4 Hz, 1H), 5.76 (d, *J* = 17.6 Hz, 1H), 5.26 (d, *J* = 10.9 Hz, 1H), 1.32 (s, 12H). **^{13}C{^{1}H} NMR** (151 MHz, $CDCl_3$) δ 149.0, 138.1, 137.0, 136.4, 127.3, 126.4, 114.3, 83.4, 24.8.

Comparison of TON and TOF

Supplementary Table 5. Comparison of TON and TOF for **microcrystalline Cu_4NC** with previous reports for the hydroboration of alkynes.

Entry	Catalyst	TON	TOF/ h^{-1}	Condition	Reference
1	Vanadium(III) catalys	4000	----	Homogeneous, rt, N_2, 16h	24
2	JNM-4-Ns	41734[a]	41734	Heterogeneous, rt, air, 20 min	29
3	Cu_1-O(I)/CeO_2	198	198	Heterogeneous, 90 °C, dried, N_2, 1h	30
4	$Co(OAc)_2$-ligand	18.2	0.76	Homogeneous, rt, dried, N_2, 24h	31
5	Cu-PC-1	37.2	5.3	Homogeneous, rt, 405 nm, Ar, 7h	32
6	Cu-$CuFe_2O_4$	190	23.75	Heterogeneous, rt, air, 8 h	33
7	$MgBu_2$	12.14	0.67	Homogeneous, 80 °C, dried and N_2, 18h	34
8	micro copper powder	9.6	0.4	Heterogeneous, rt, Ar, 11h or 24h	35
9	Pd_2dba_2-ligand	46.5	----	Homogeneous, rt, 0.5h-2.5h	36
10	R_3Al·DABCO	8.9	4.45	Homogeneous, 110 °C, dried, N_2, 2h	37
11	(^{Cy}APDI)$CoCH_3$	27.67	4.61	Homogeneous, rt, dried, N_2, 6h	38
12	$[Pd(OAc)_2]_3$-ligand	18.6	1.55	Homogeneous, 80 °C, dried, Ar, 12h	39
13	LCuCl	23.5	1.96	Homogeneous, rt, dried, Ar, 12h	40
14	JNM-5	1182	394	Heterogeneous, rt, N_2, 3 h	41
15	$Cu_{45}NC$	3680	736	Heterogeneous, rt, air, 5 h	42
16	$Co(acac)_2$-ligand	198	16.5	Homogeneous, rt, N_2, 12h	43
17	*dom*-NENU-3_2	99.5	24.88	Heterogeneous, 50 °C, N_2, 4h	44
18	FeO/MgO	34	1.7	Heterogeneous, 160 °C, Ar, 20 h	45
19	**microcrystalline Cu_4NC**	**77786**	**16286**	**Heterogeneous, rt, air, 1h**	**This work**

"a" represents the absence of the definitive values in the article.

^{1}H, ^{13}C and ^{19}F NMR Spectra of Compounds

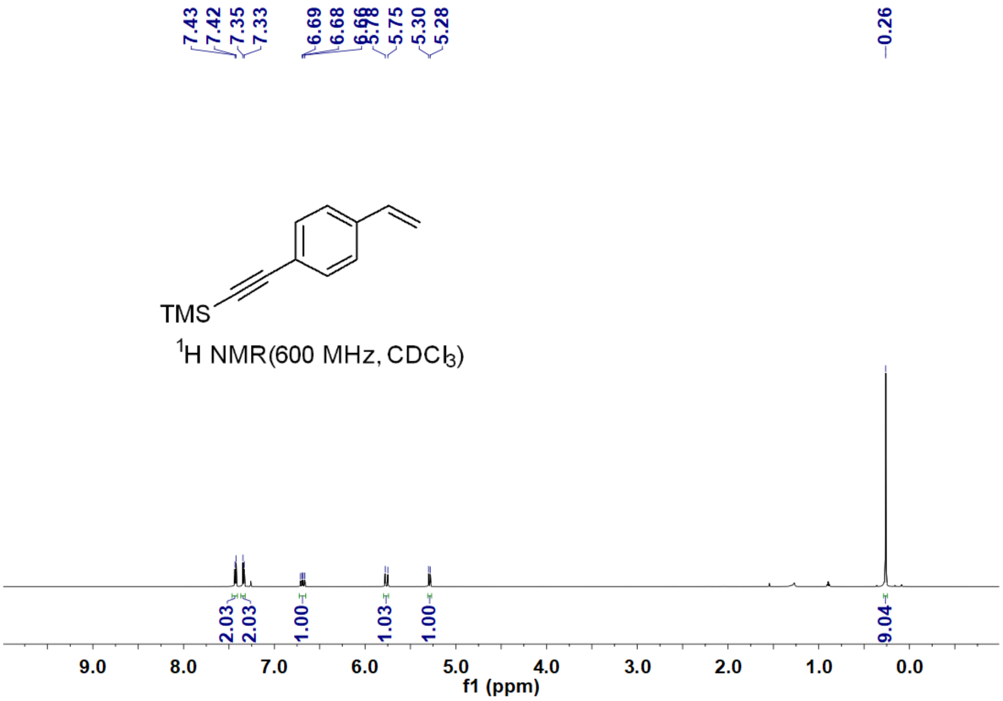

Supplementary Figure 110. Characterization of compound trimethyl((4-vinylphenyl) ethynyl) silane. ^{1}H NMR spectrum of trimethyl((4-vinylphenyl) ethynyl) silane in $CDCl_3$.

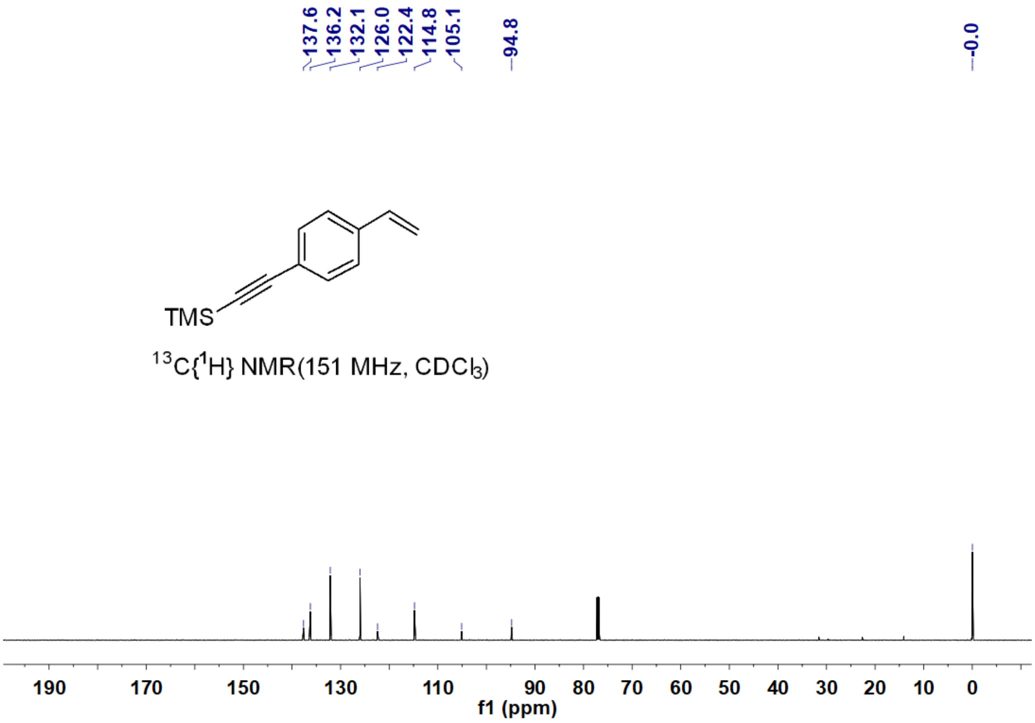

Supplementary Figure 111. Characterization of compound trimethyl((4-vinylphenyl) ethynyl) silane. $^{13}C\{^{1}H\}$ NMR spectrum of trimethyl((4-vinylphenyl) ethynyl) silane in $CDCl_3$.

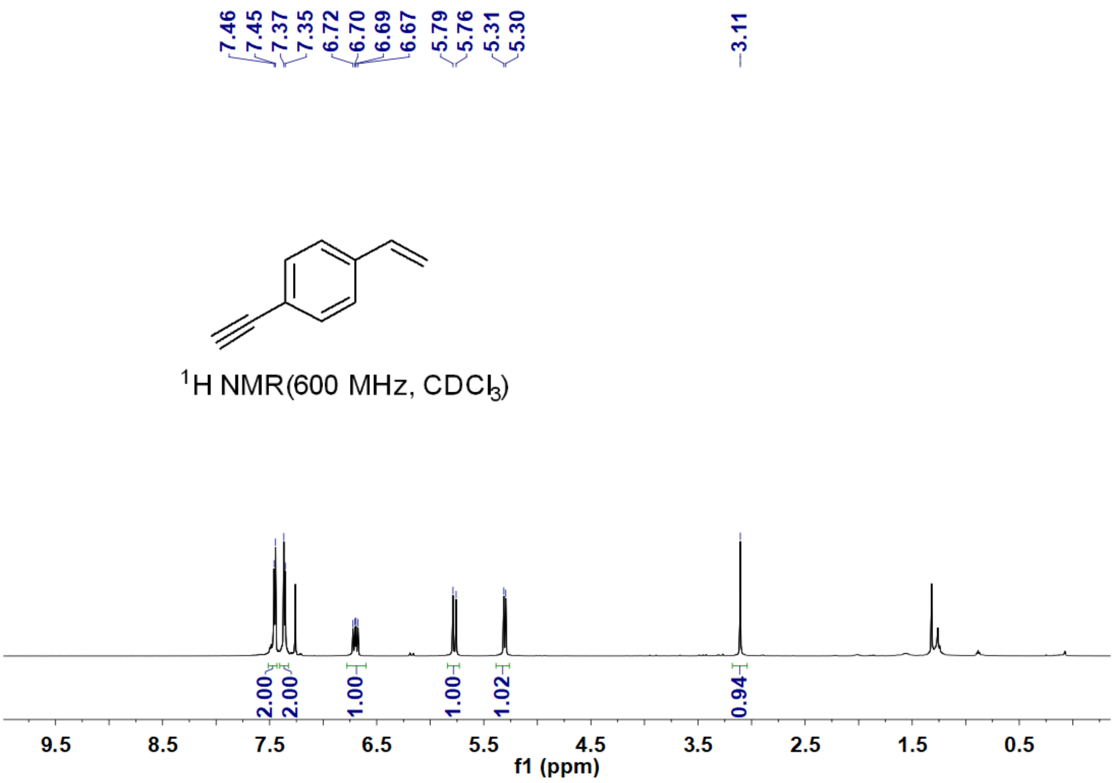

Supplementary Figure 112. Characterization of compound 1ab. ^{1}H NMR spectrum of compound **1ab** in $CDCl_3$.

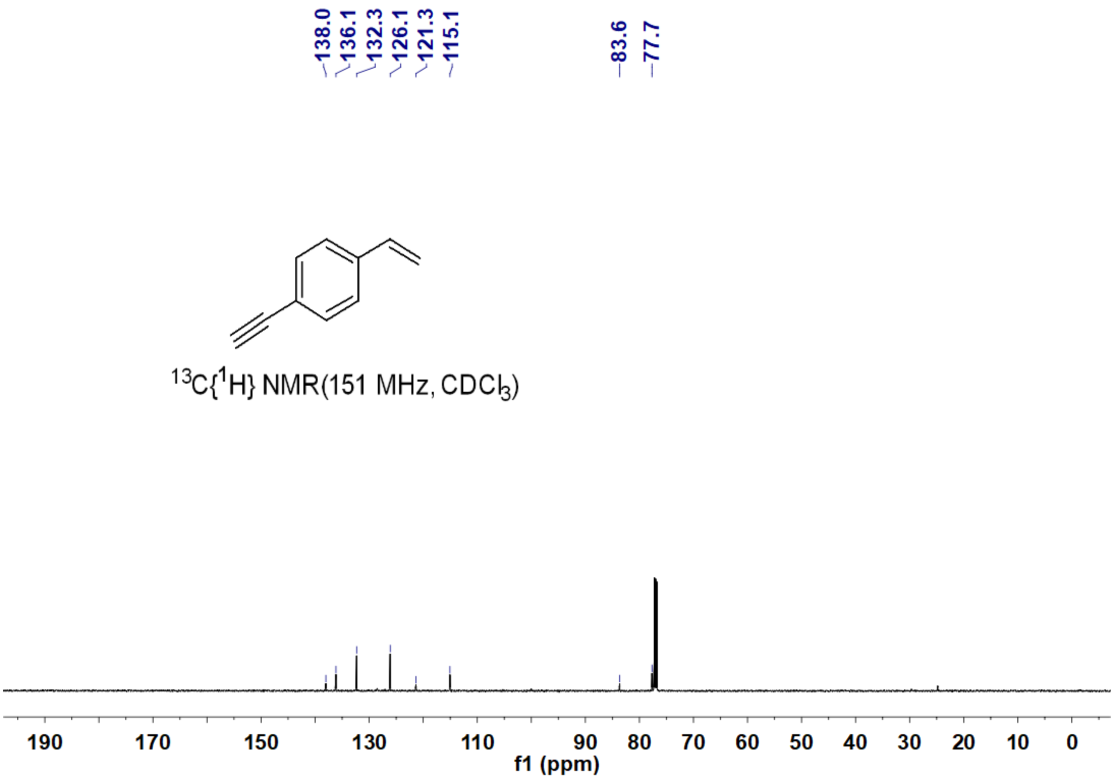

Supplementary Figure 113. Characterization of compound 1ab. ^{13}C{^{1}H} NMR spectrum of compound **1ab** in $CDCl_3$.

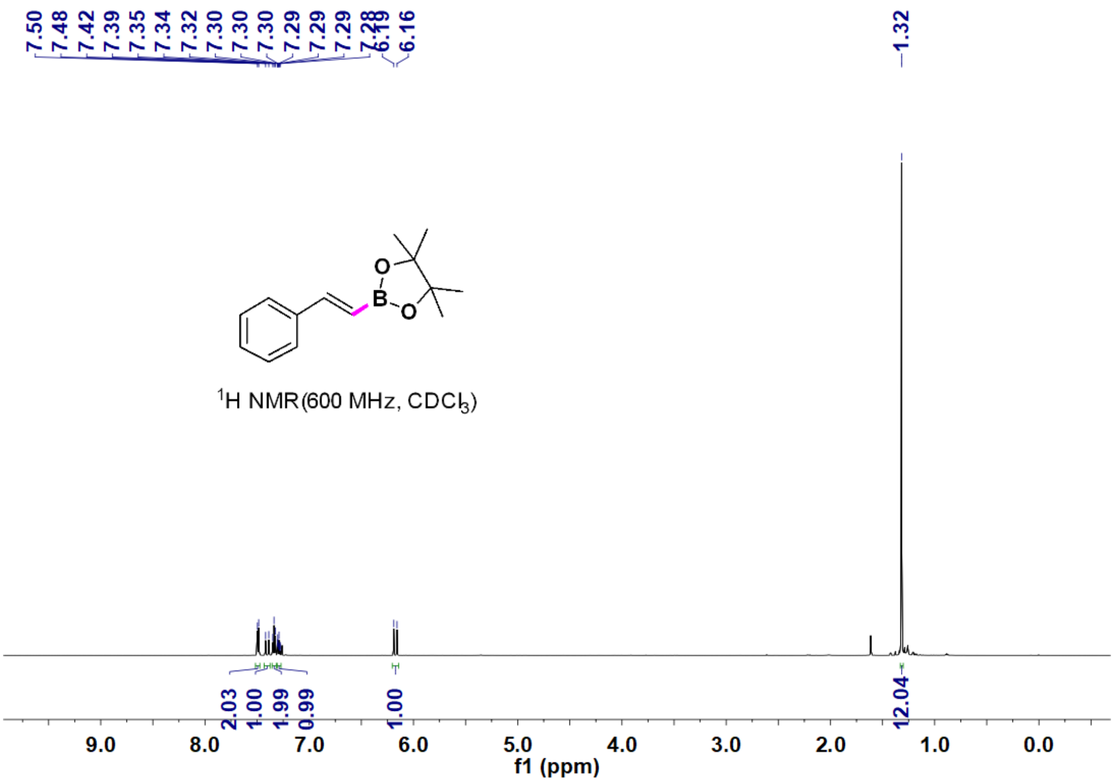

Supplementary Figure 114. Characterization of compound 4a. ^{1}H NMR spectrum of compound **4a** in CDCl$_3$.

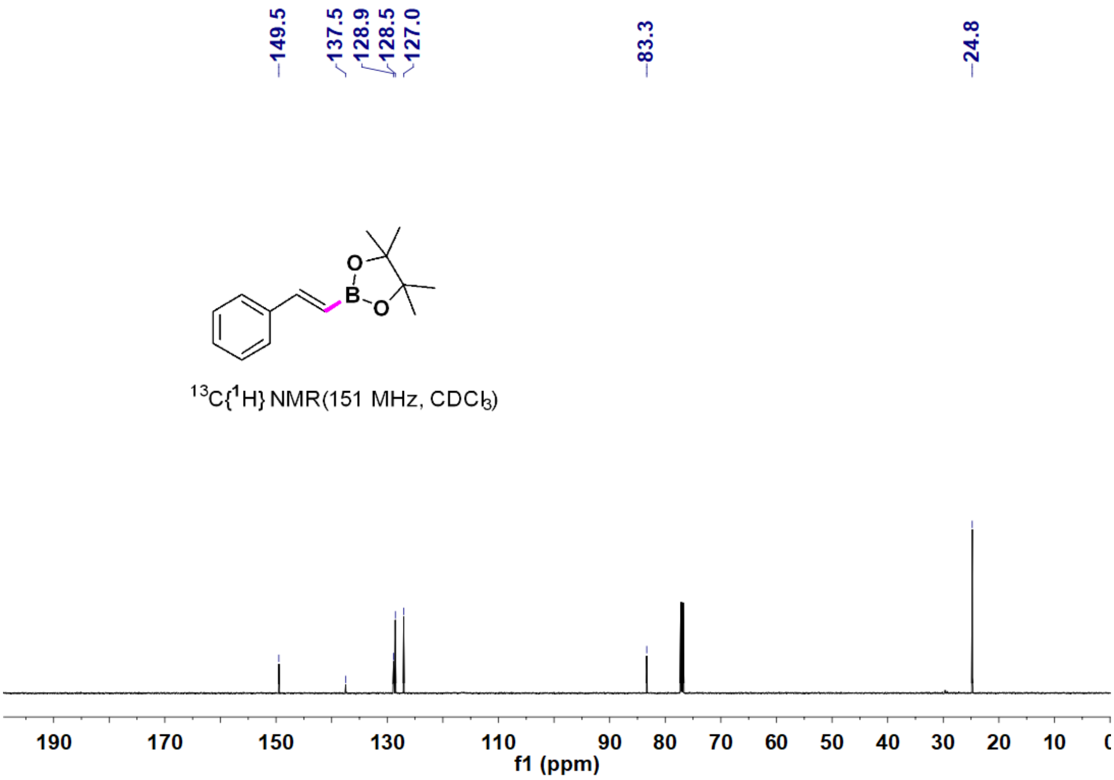

Supplementary Figure 115. Characterization of compound 4a. ^{13}C{^{1}H} NMR spectrum of compound **4a** in CDCl$_3$.

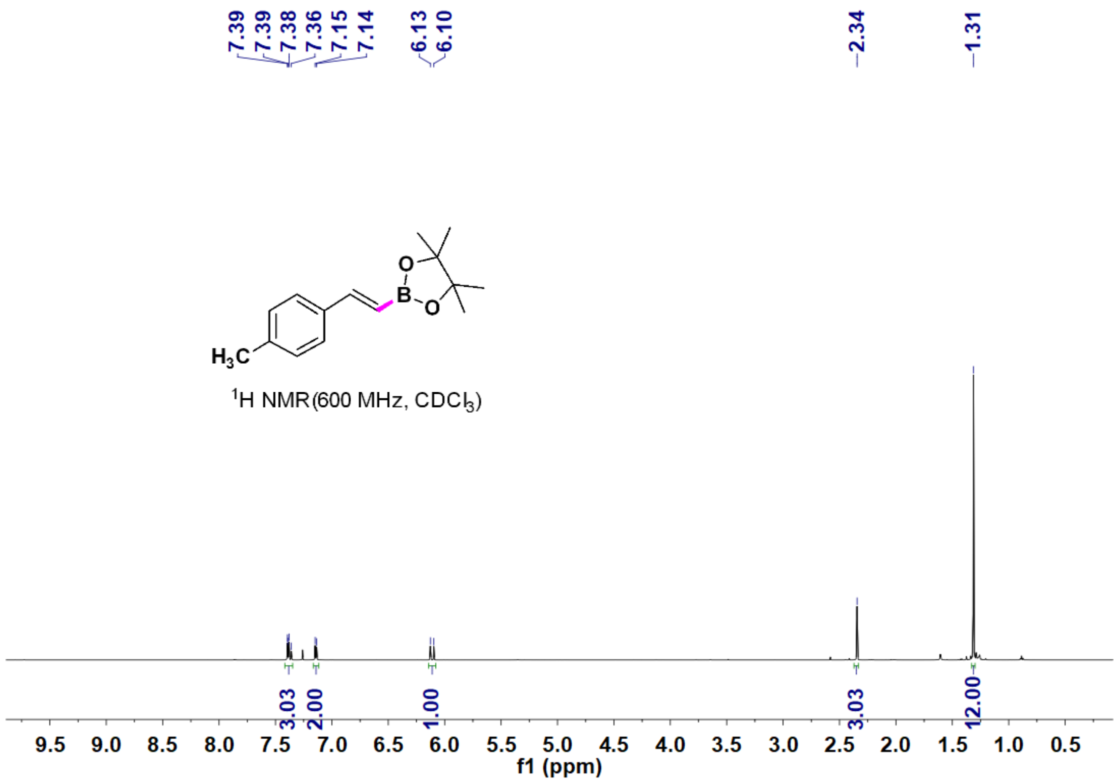

Supplementary Figure 116. Characterization of compound 4b. ^{1}H NMR spectrum of compound **4b** in $CDCl_3$.

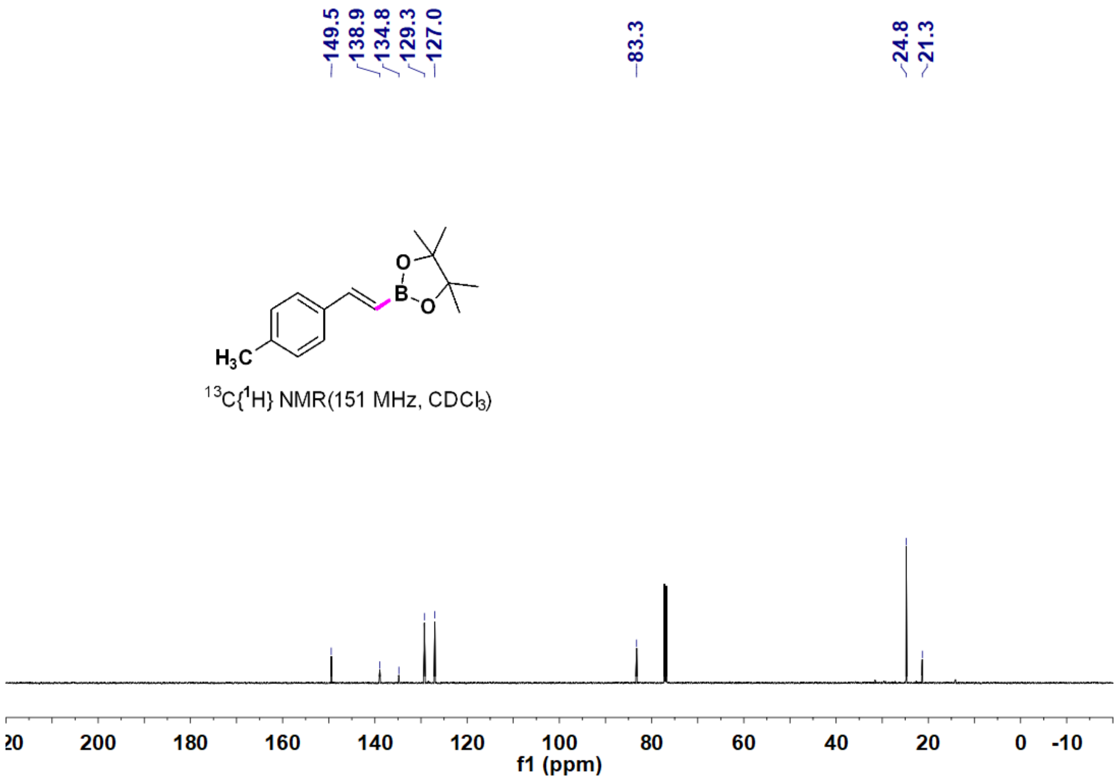

Supplementary Figure 117. Characterization of compound 4b. $^{13}C\{^{1}H\}$ NMR spectrum of compound **4b** in $CDCl_3$.

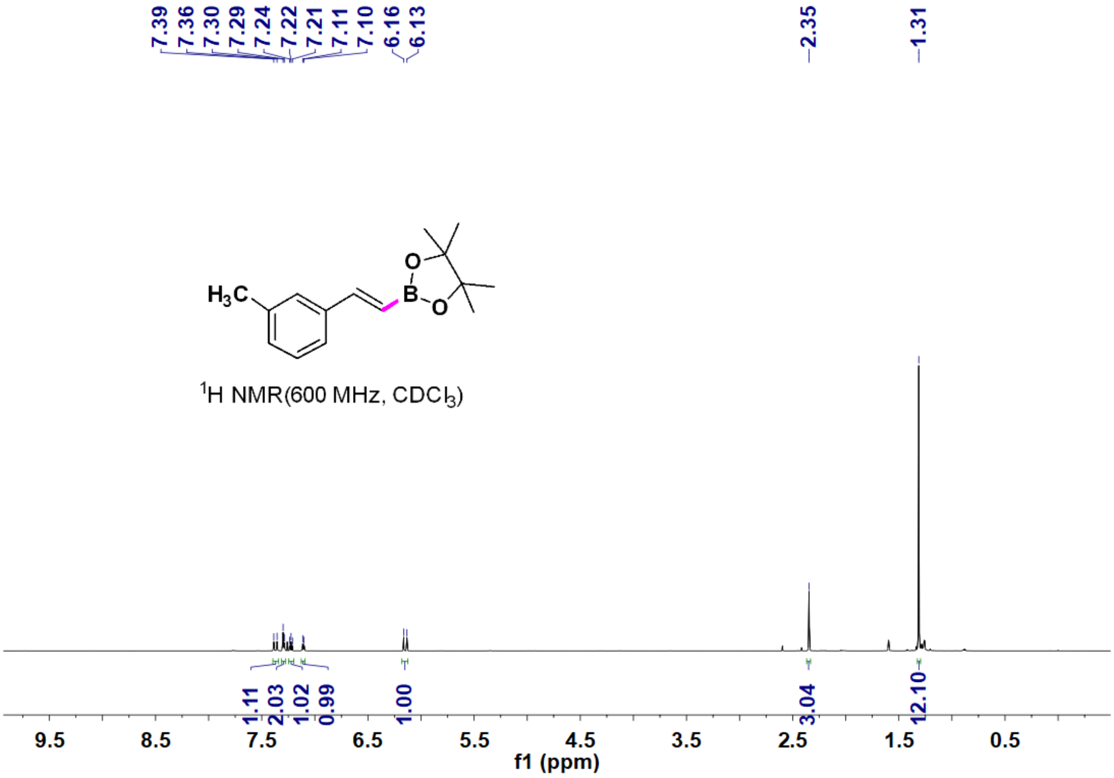

Supplementary Figure 118. Characterization of compound 4c. ^{1}H NMR spectrum of compound **4c** in $CDCl_3$.

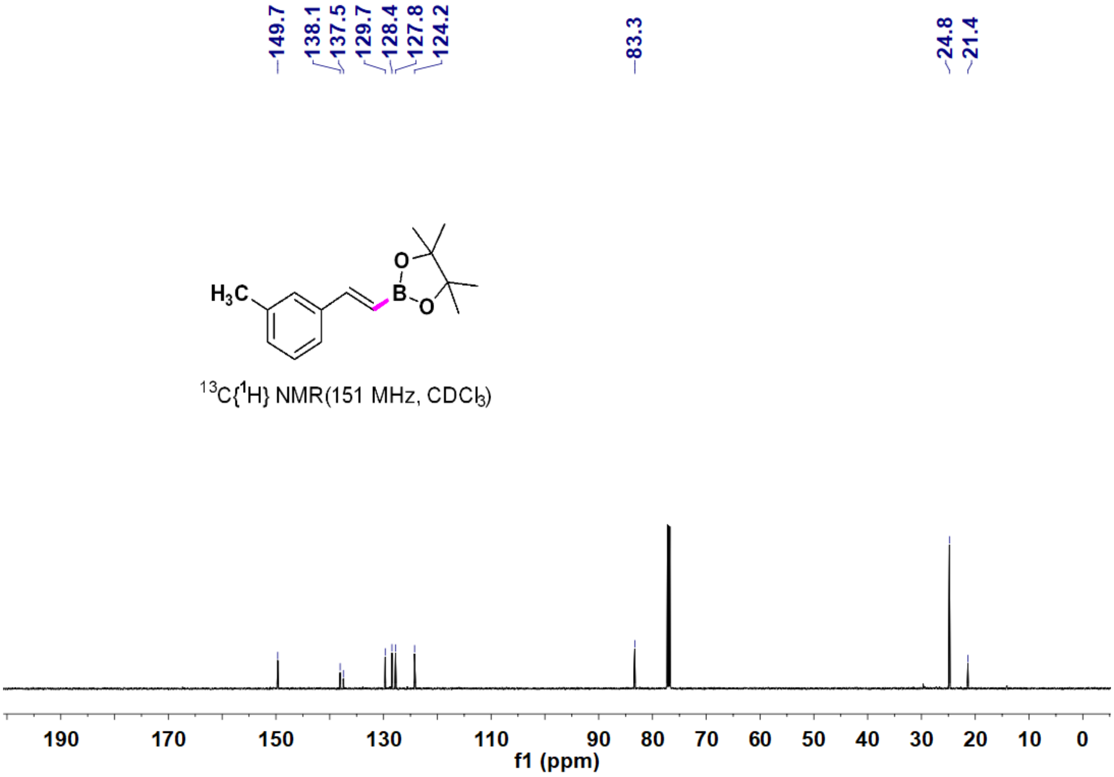

Supplementary Figure 119. Characterization of compound 4c. $^{13}C\{^{1}H\}$ NMR spectrum of compound **4c** in $CDCl_3$.

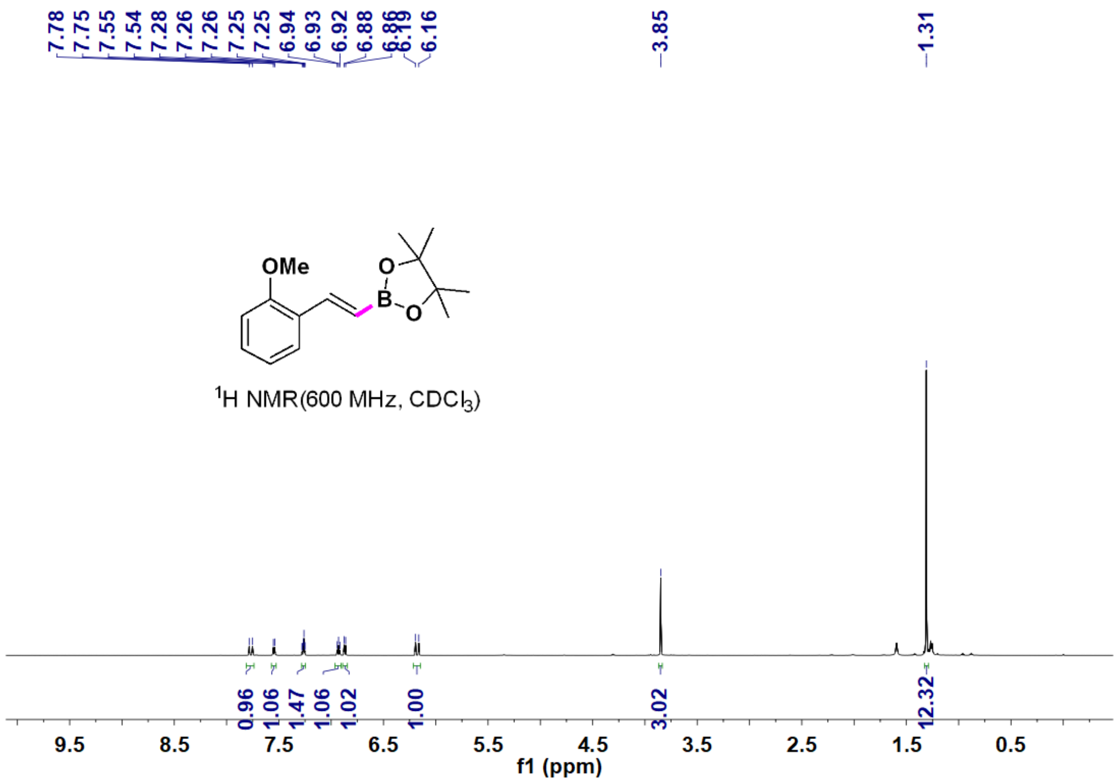

Supplementary Figure 120. Characterization of compound 4d. ^{1}H NMR spectrum of compound **4d** in $CDCl_3$.

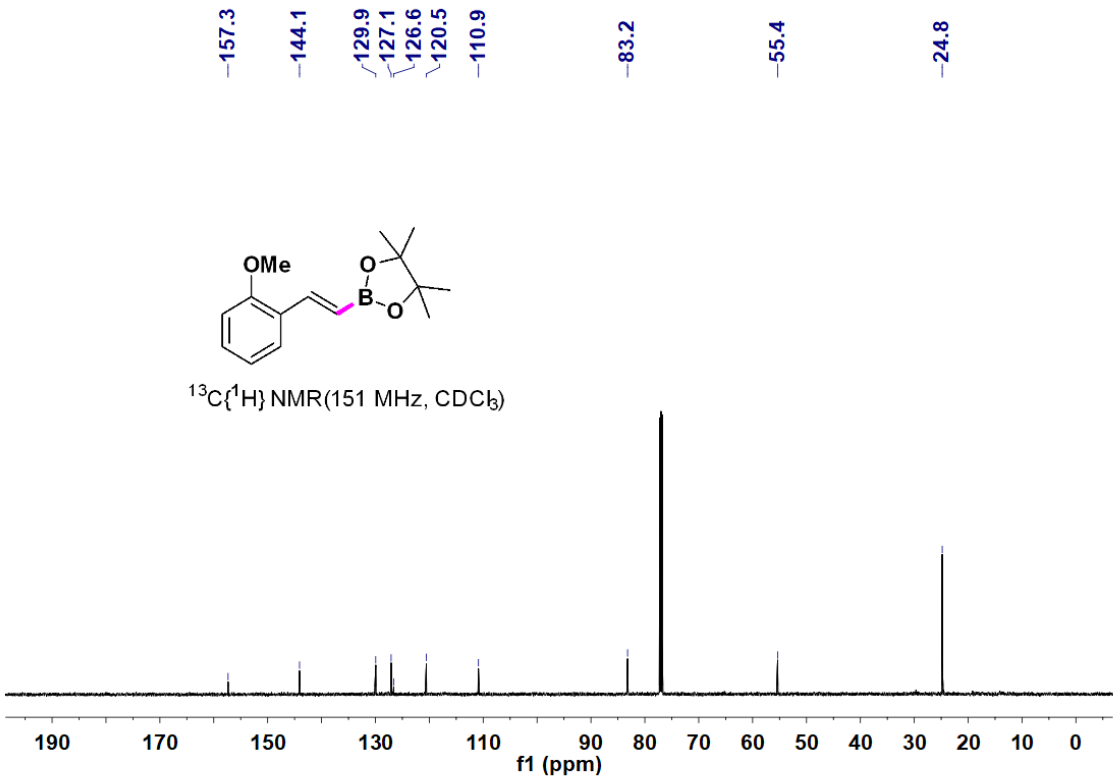

Supplementary Figure 121. Characterization of compound 4d. $^{13}C\{^1H\}$ NMR spectrum of compound **4d** in $CDCl_3$.

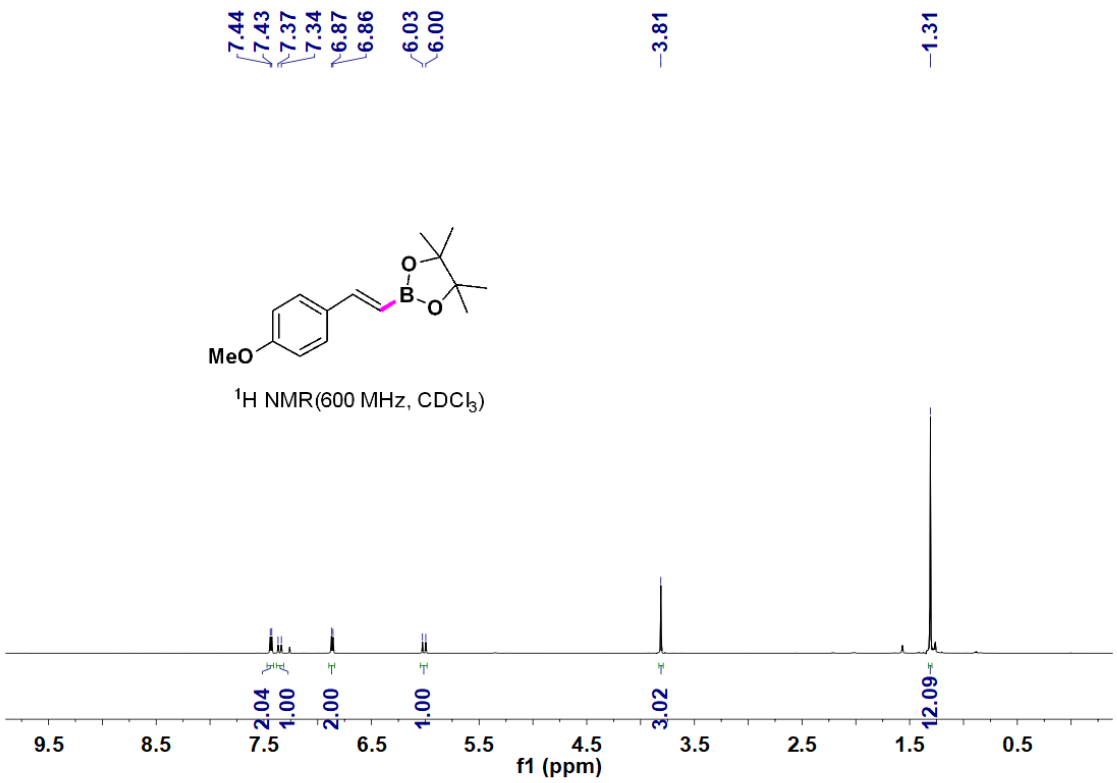

Supplementary Figure 122. Characterization of compound 4e. ^{1}H NMR spectrum of compound **4e** in $CDCl_3$.

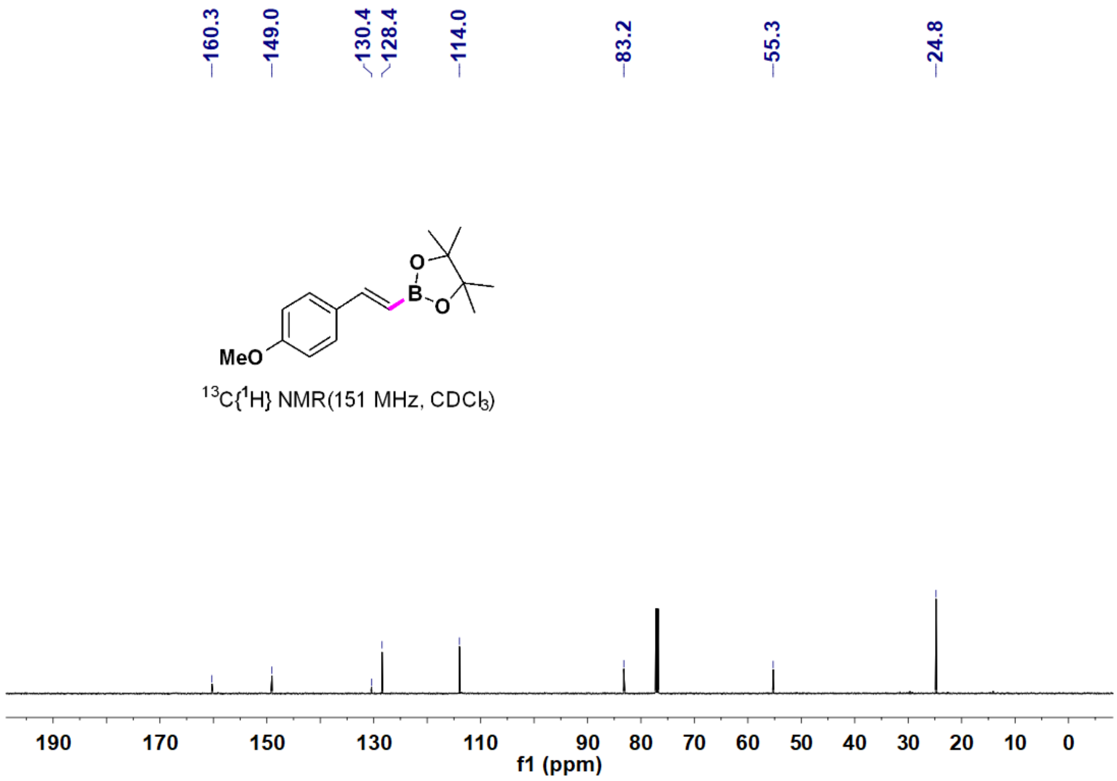

Supplementary Figure 123. Characterization of compound 4e. $^{13}C\{^{1}H\}$ NMR spectrum of compound **4e** in $CDCl_3$.

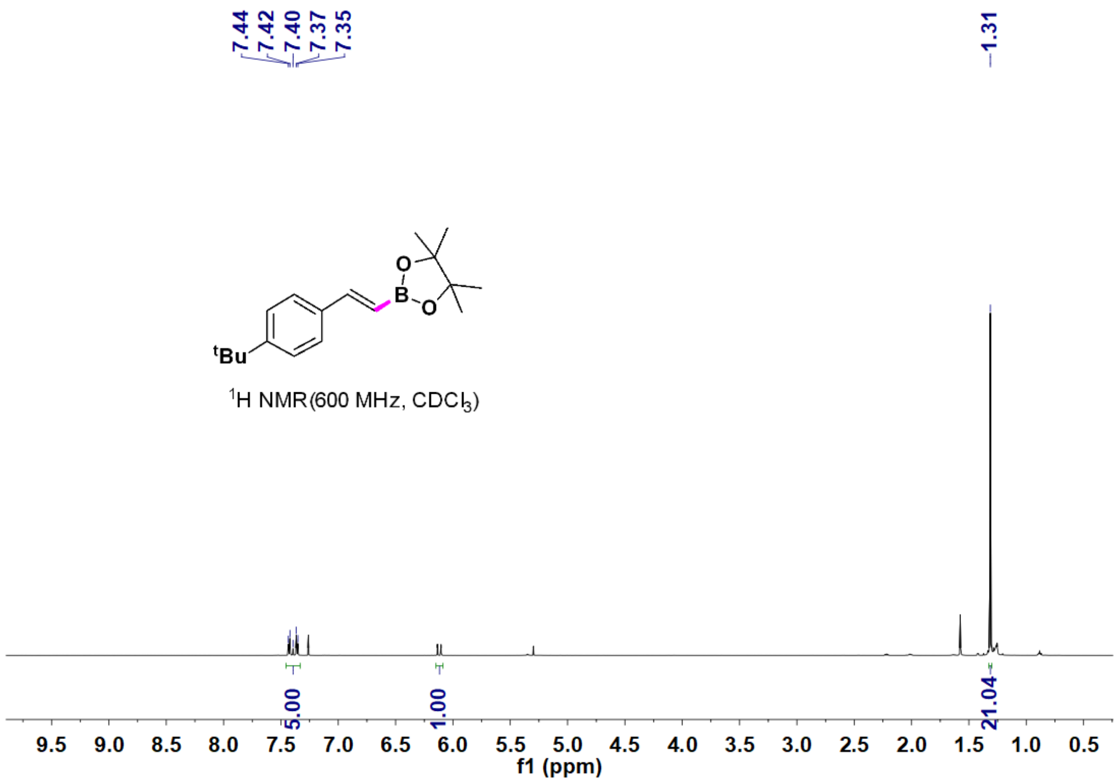

Supplementary Figure 124. Characterization of compound 4f. ^{1}H NMR spectrum of compound **4f** in $CDCl_3$.

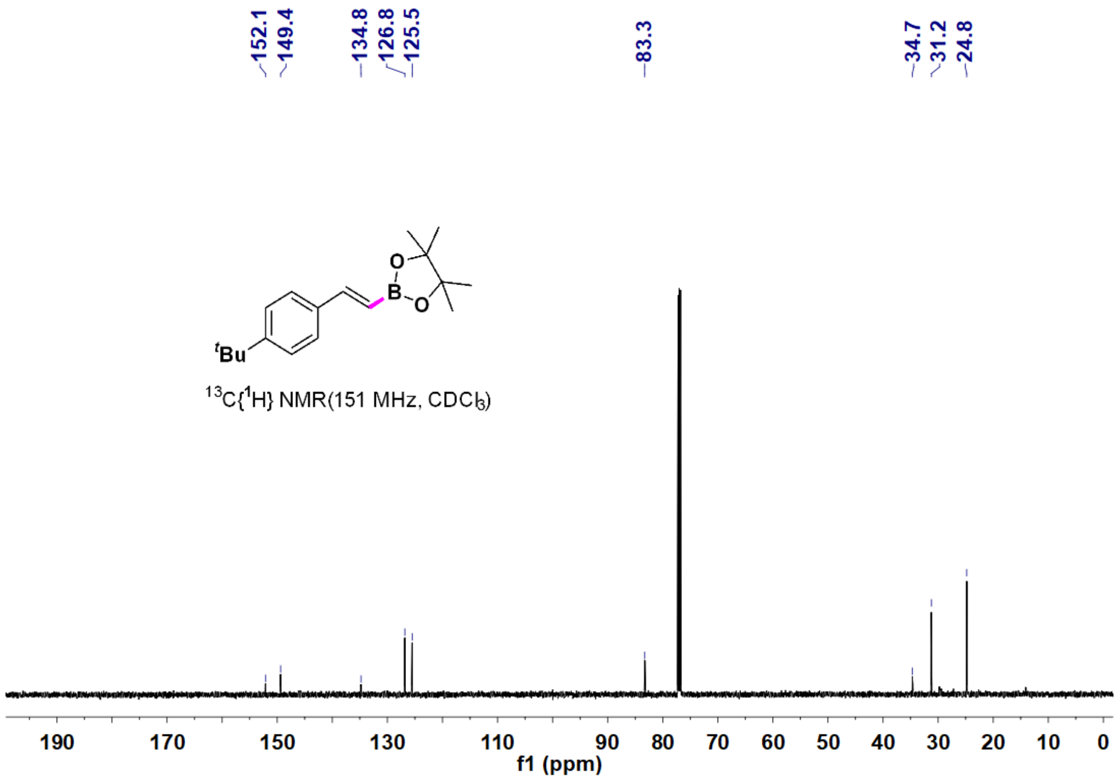

Supplementary Figure 125. Characterization of compound 4f. $^{13}C\{^{1}H\}$ NMR spectrum of compound **4f** in $CDCl_3$.

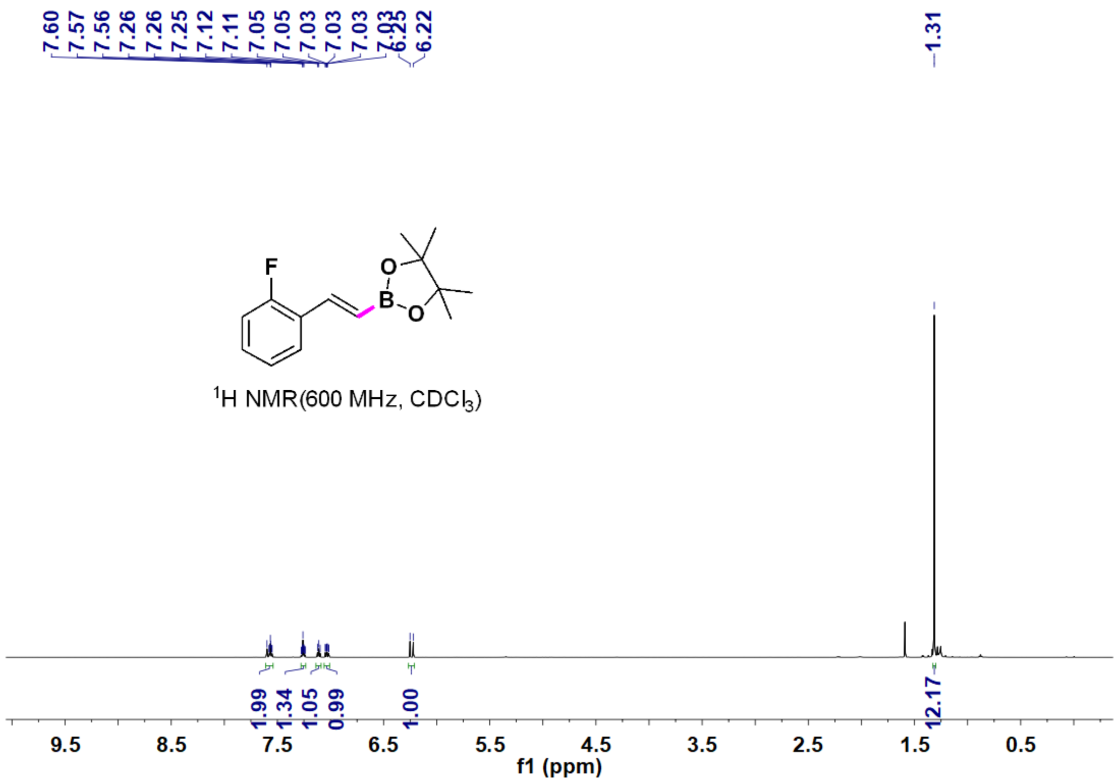

Supplementary Figure 126. Characterization of compound 4g. ^{1}H NMR spectrum of compound **4g** in $CDCl_3$.

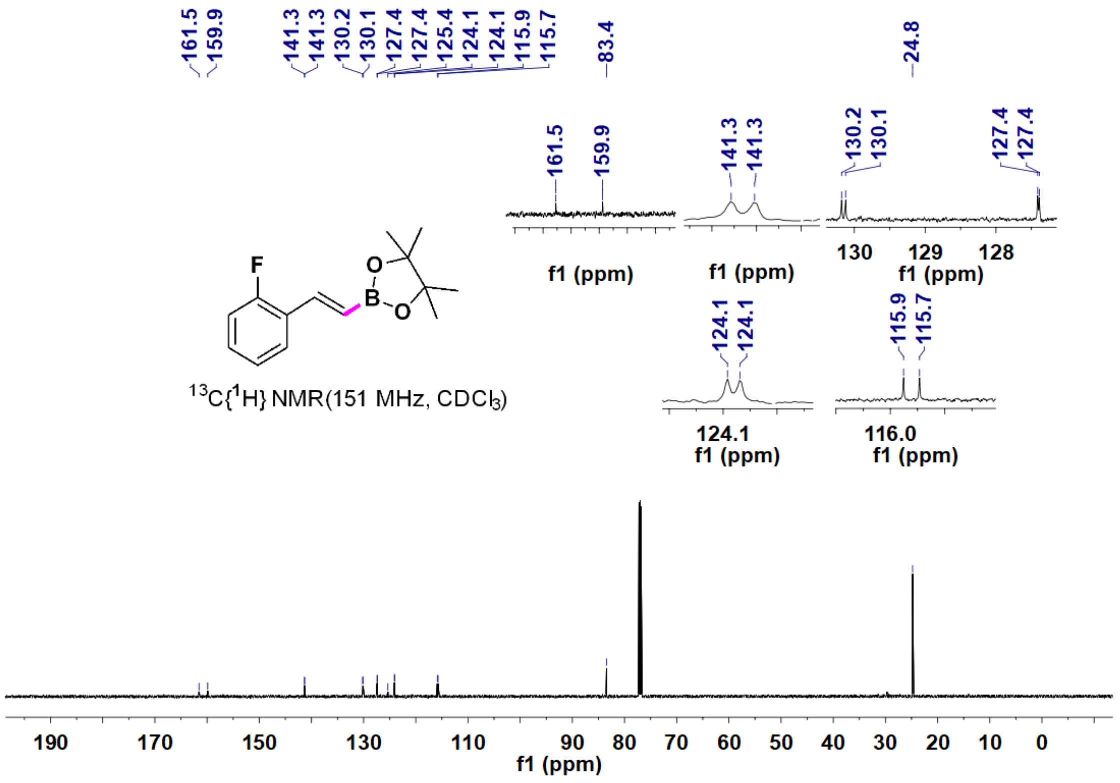

Supplementary Figure 127. Characterization of compound 4g. $^{13}C\{^1H\}$ NMR spectrum of compound **4g** in $CDCl_3$.

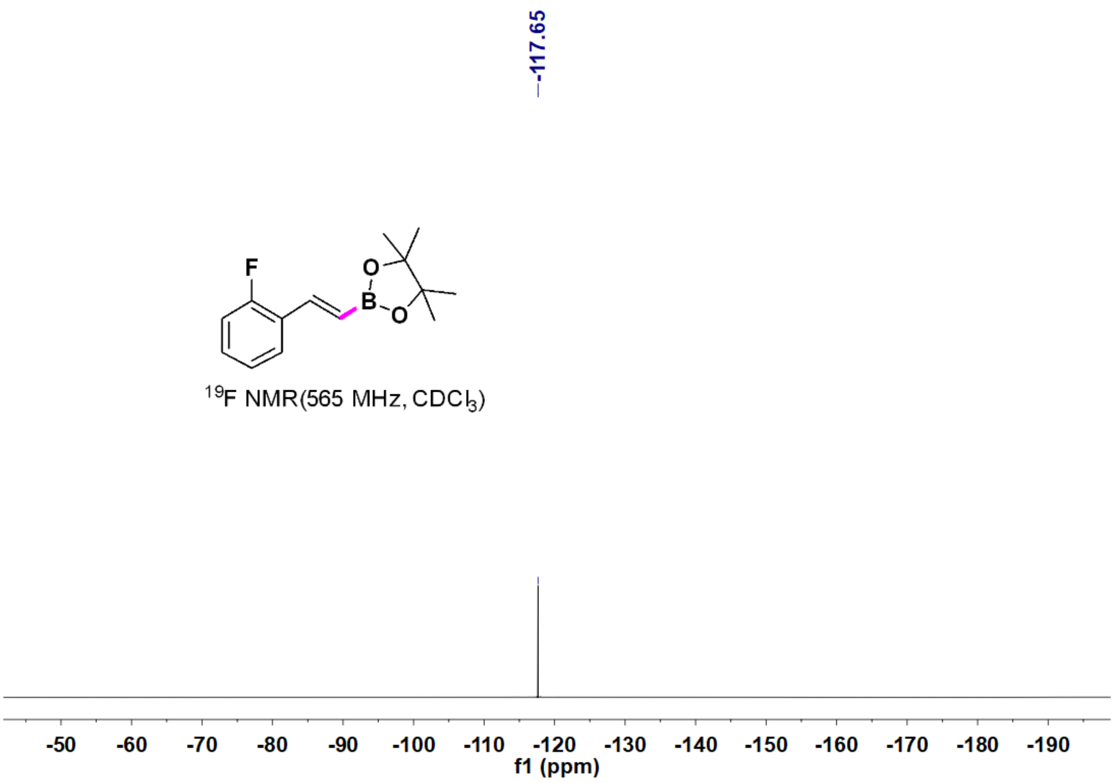

Supplementary Figure 128. Characterization of compound 4g. ^{19}F NMR spectrum of compound **4g** in $CDCl_3$.

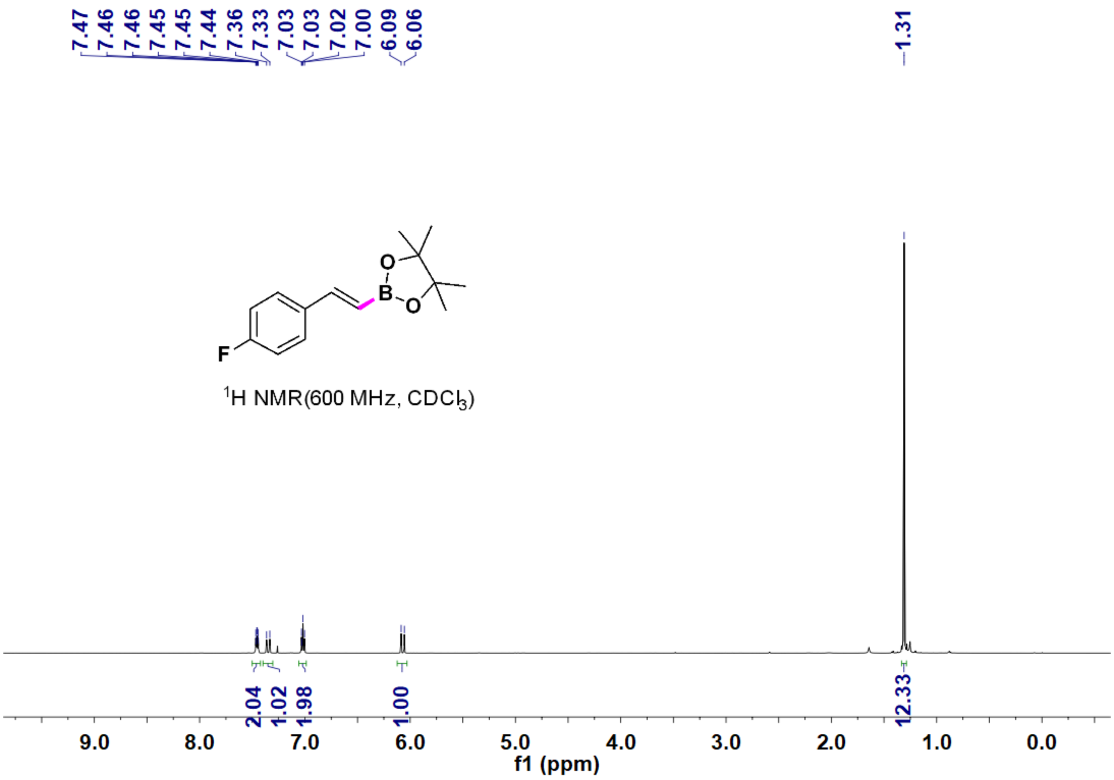

Supplementary Figure 129. Characterization of compound 4h. ^{1}H NMR spectrum of compound **4h** in $CDCl_3$.

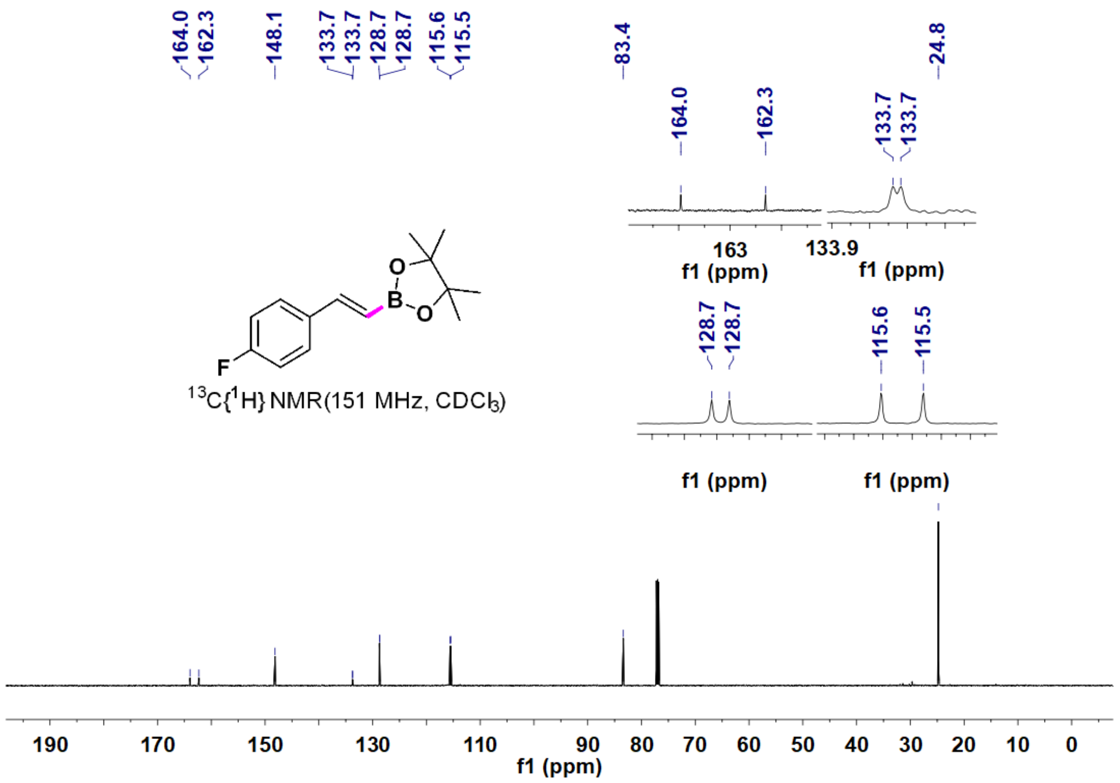

Supplementary Figure 130. Characterization of compound 4h. $^{13}C\{^1H\}$ NMR spectrum of compound **4h** in $CDCl_3$.

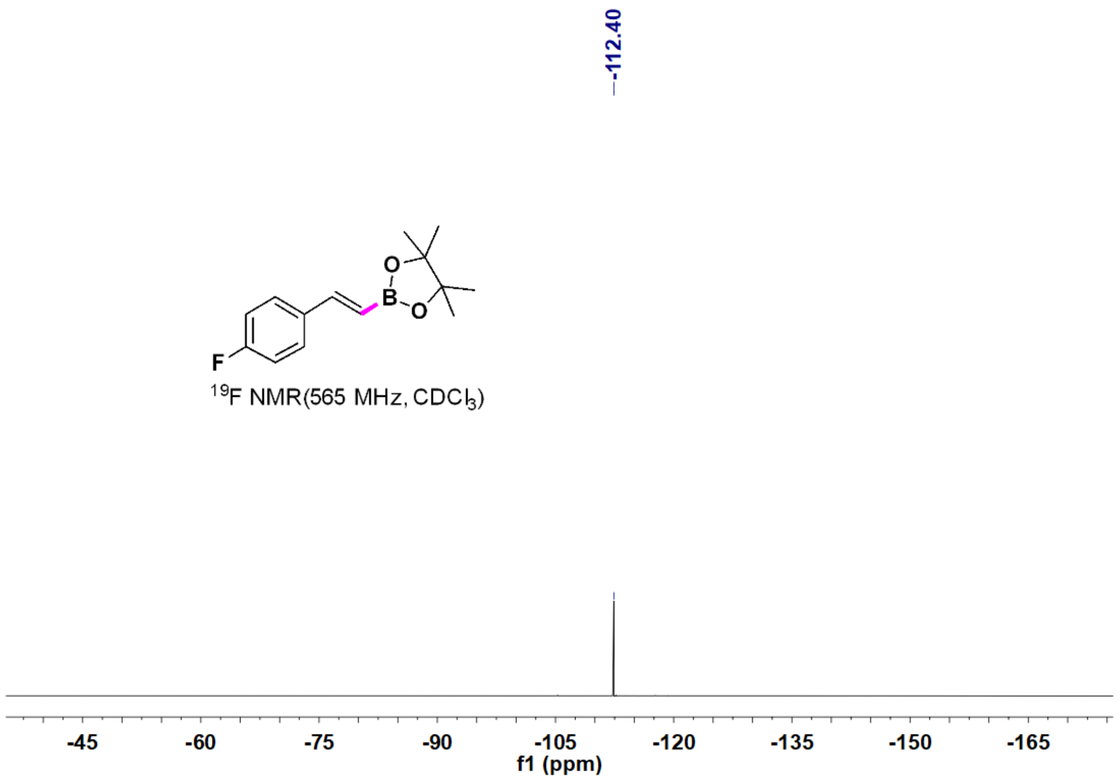

Supplementary Figure 131. Characterization of compound 4h. ^{19}F NMR spectrum of compound **4h** in $CDCl_3$.

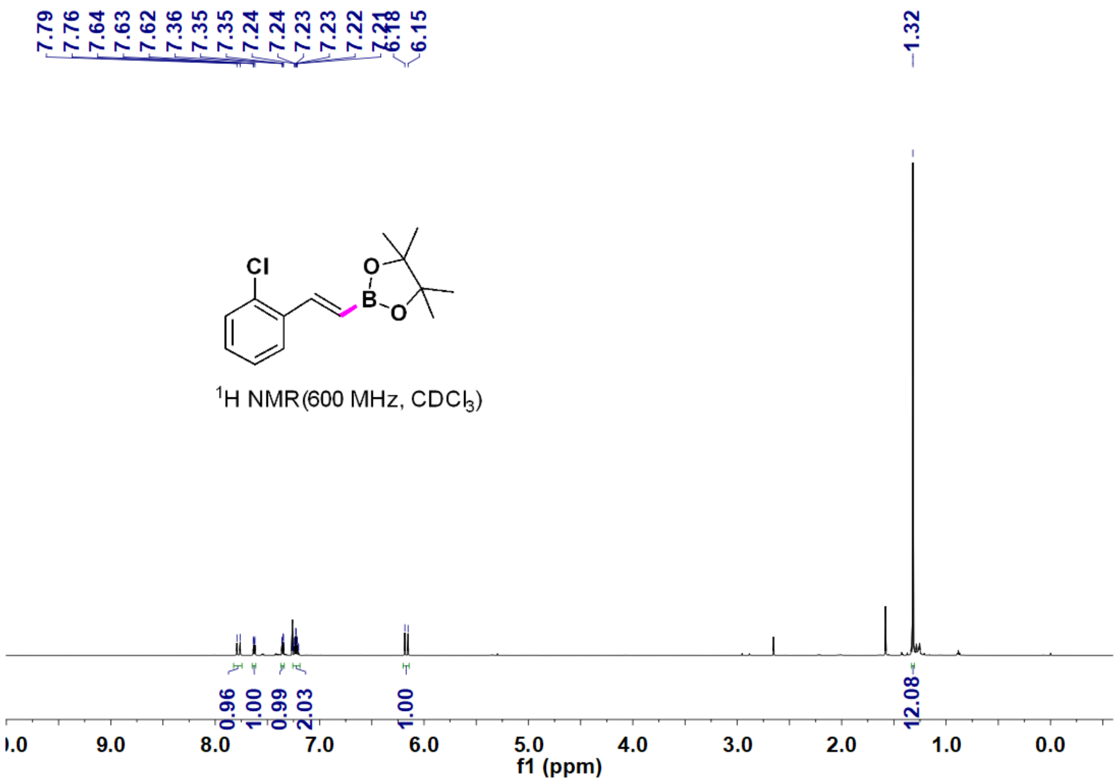

Supplementary Figure 132. Characterization of compound 4i. ^{1}H NMR spectrum of compound **4i** in $CDCl_3$.

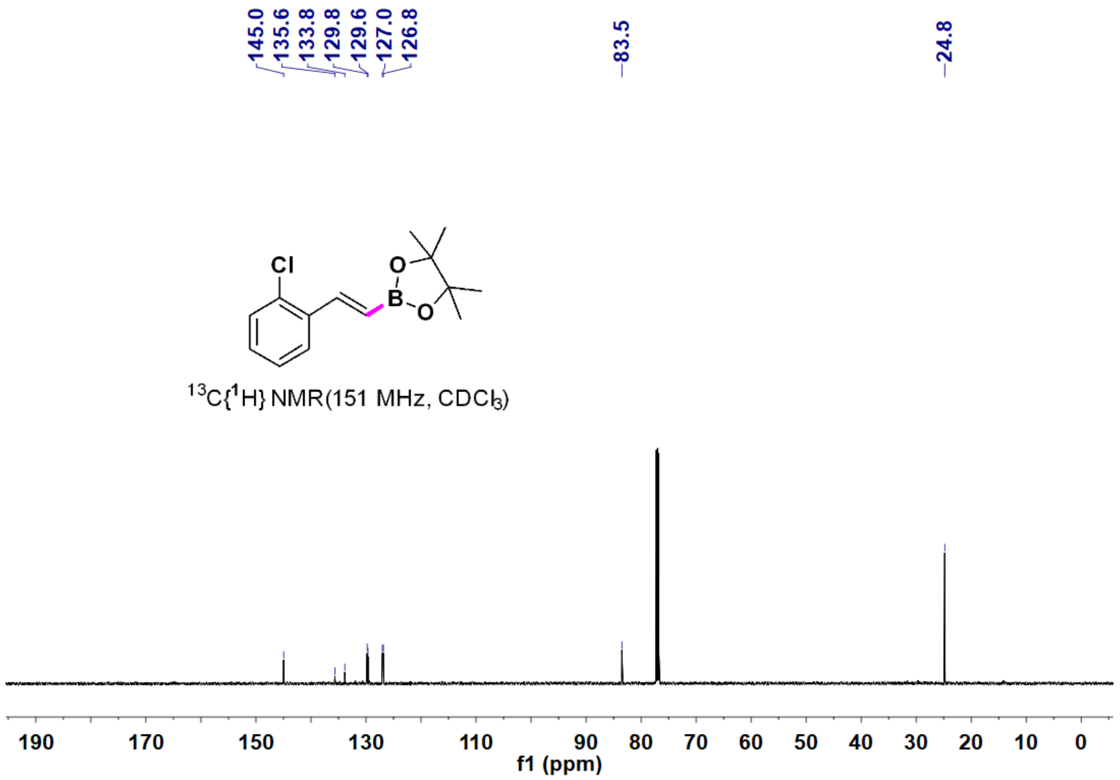

Supplementary Figure 133. Characterization of compound 4i. $^{13}C\{^{1}H\}$ NMR spectrum of compound **4i** in $CDCl_3$.

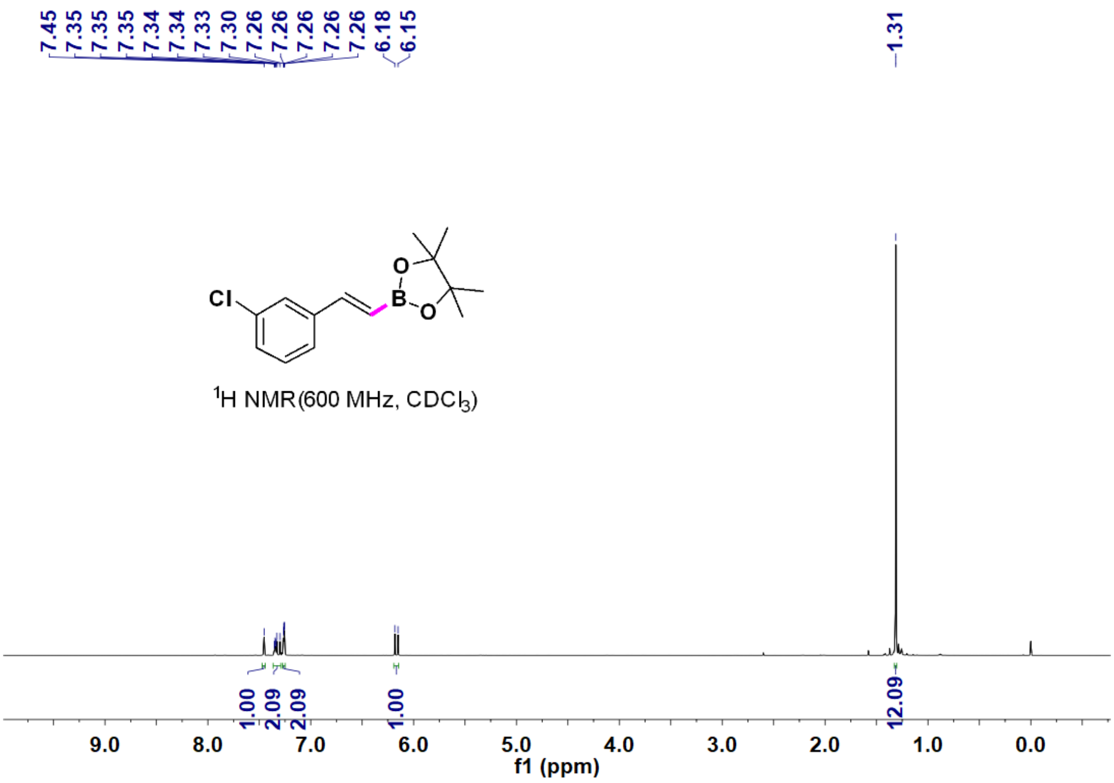

Supplementary Figure 134. Characterization of compound 4j. ^{1}H NMR spectrum of compound **4j** in $CDCl_3$.

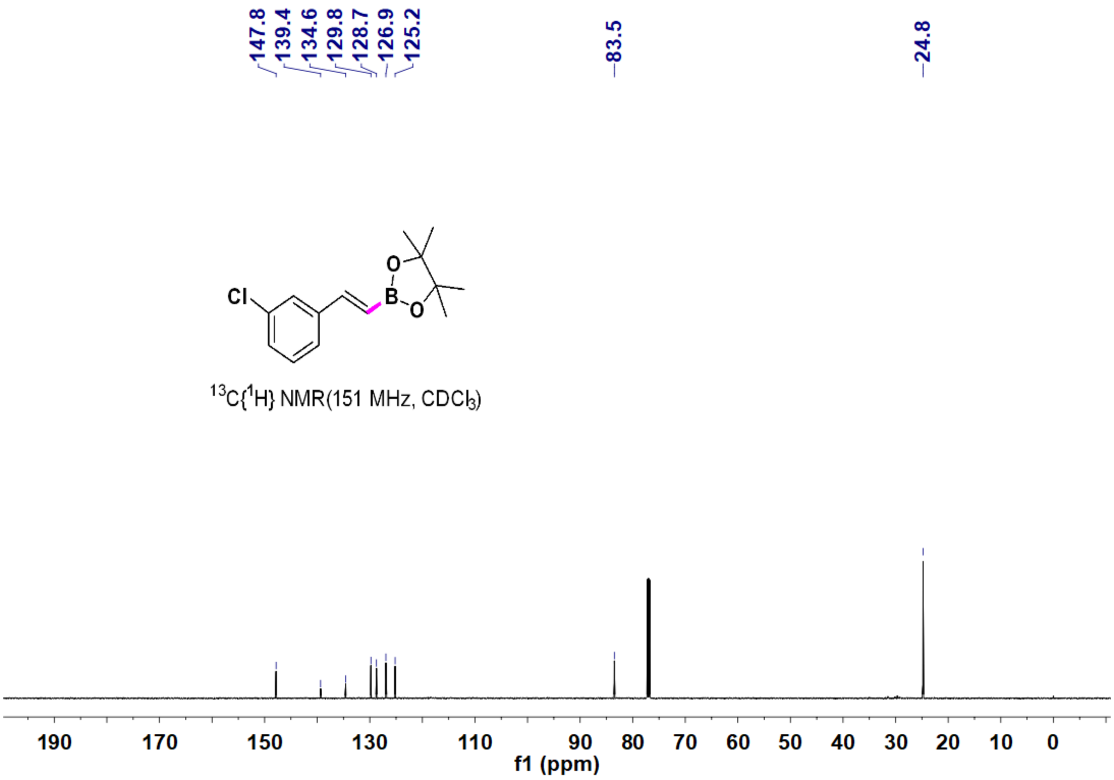

Supplementary Figure 135. Characterization of compound 4j. ^{13}C{^{1}H} NMR spectrum of compound **4j** in $CDCl_3$.

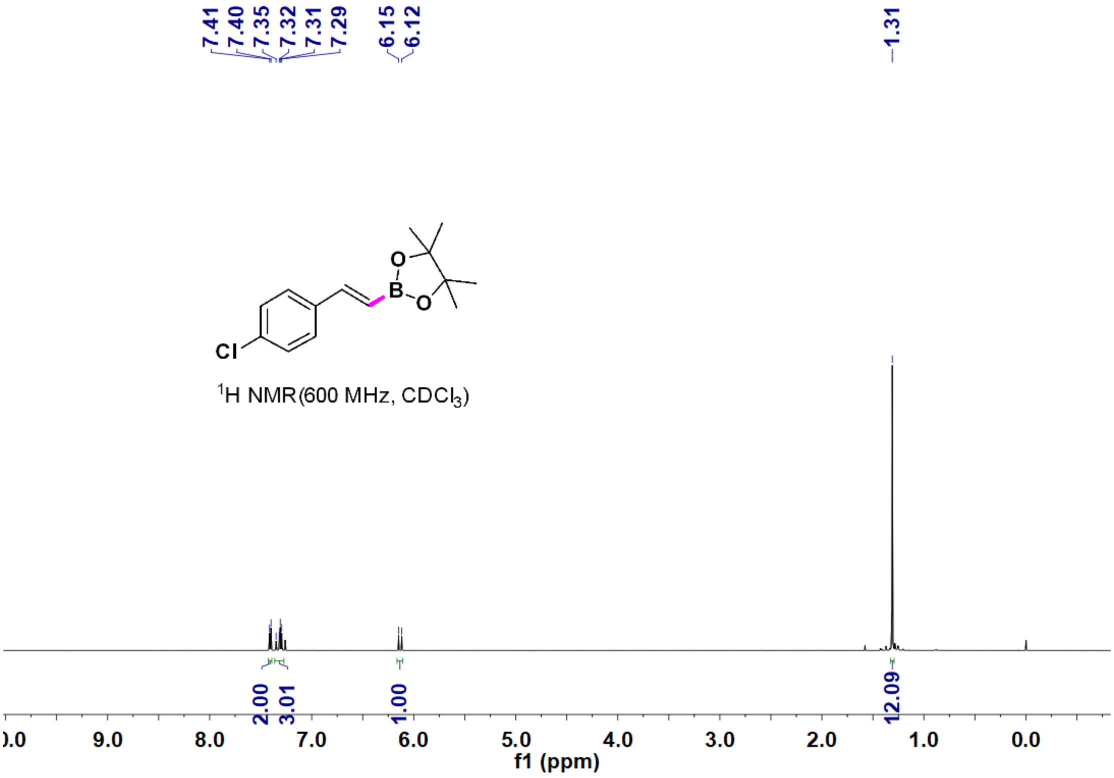

Supplementary Figure 136. Characterization of compound 4k. ^{1}H NMR spectrum of compound **4k** in $CDCl_3$.

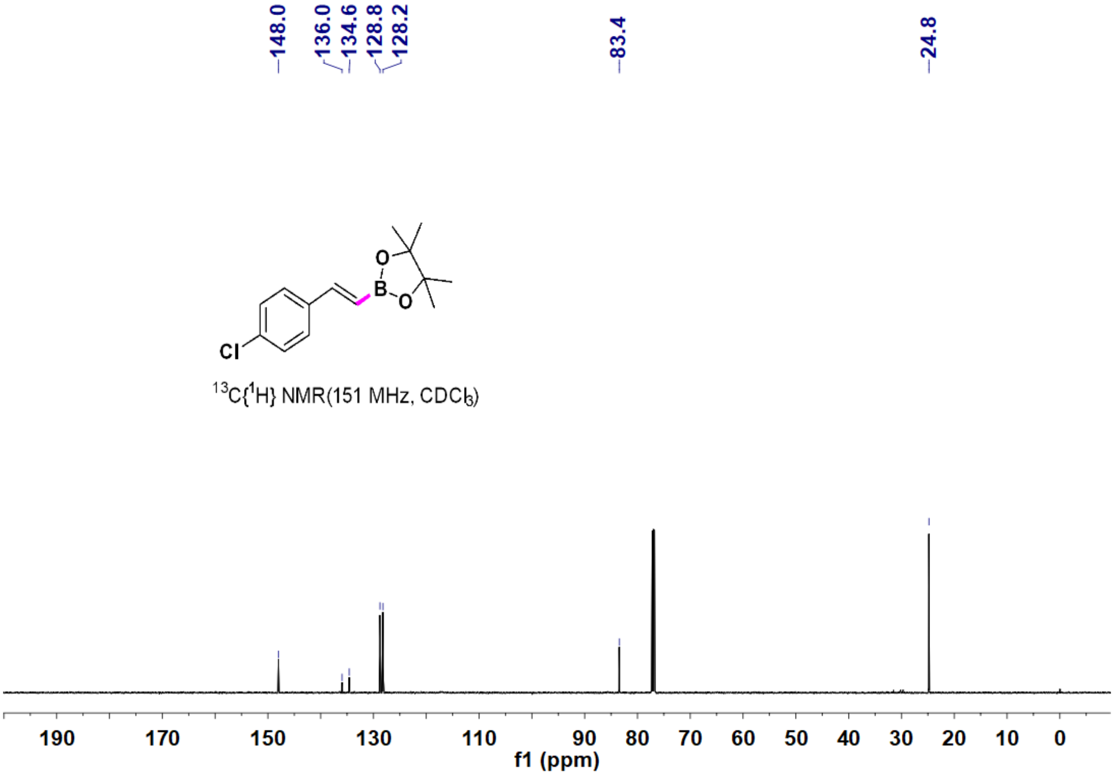

Supplementary Figure 137. Characterization of compound 4k. $^{13}C\{^1H\}$ NMR spectrum of compound **4k** in $CDCl_3$.

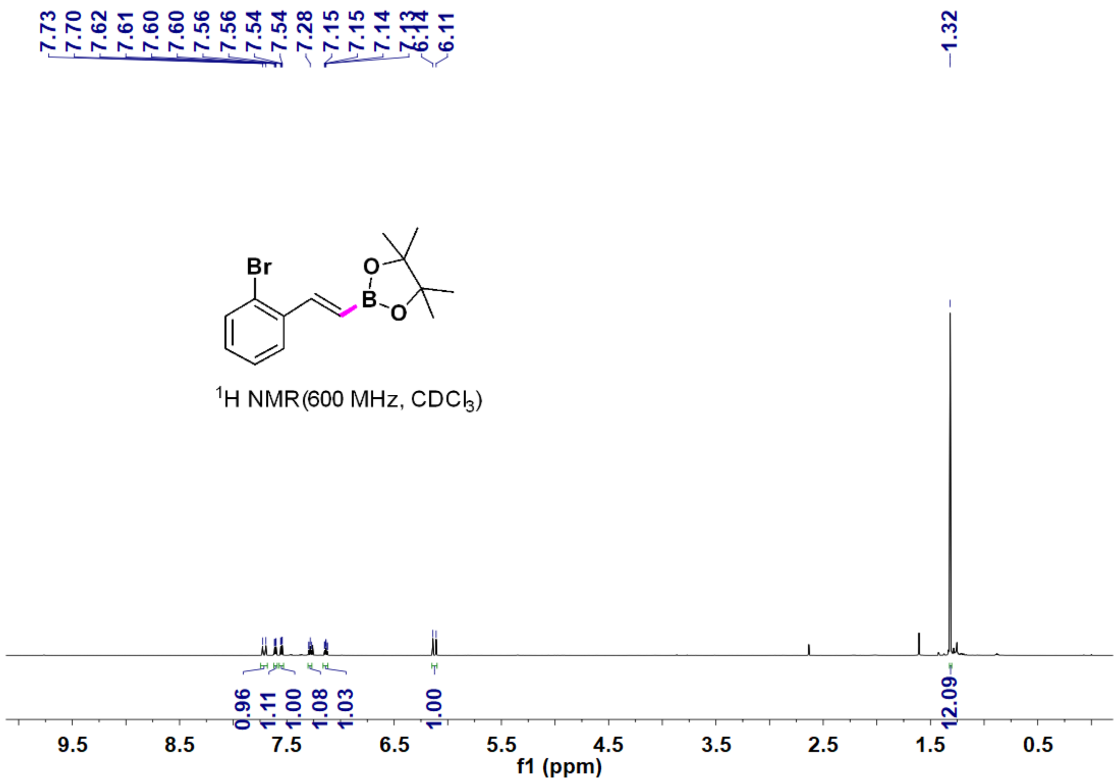

Supplementary Figure 138. Characterization of compound 4l. ^{1}H NMR spectrum of compound **4l** in $CDCl_3$.

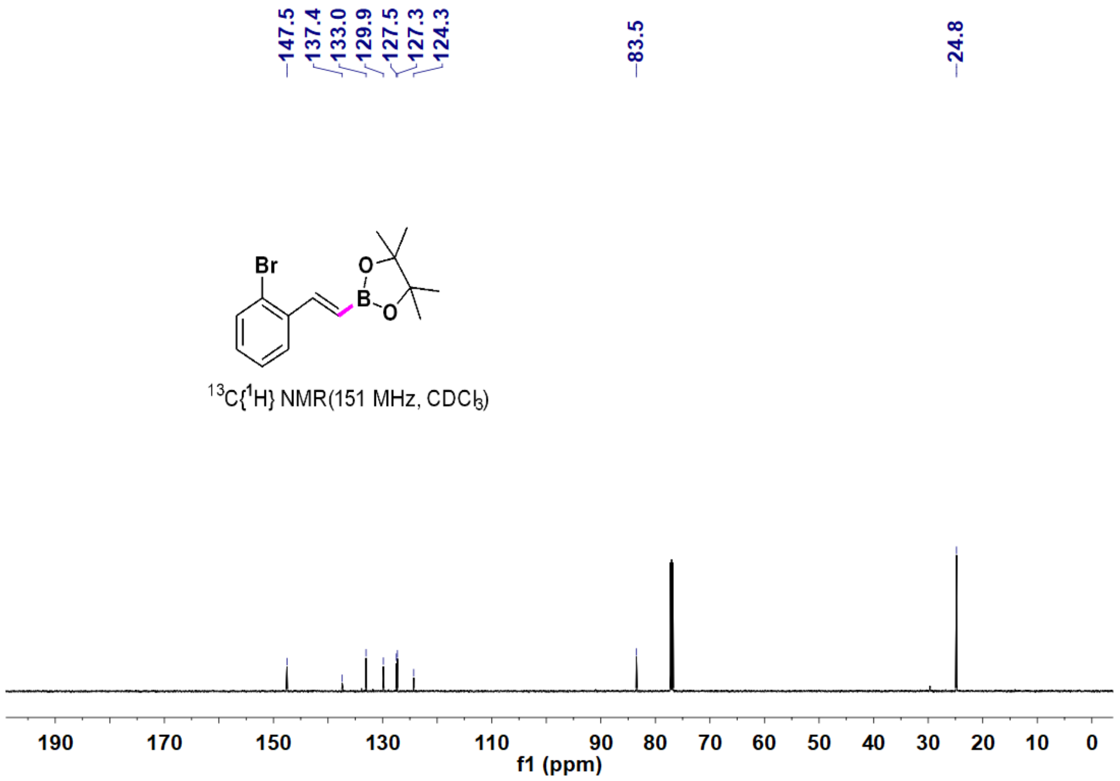

Supplementary Figure 139. Characterization of compound 4l. ^{13}C{^{1}H} NMR spectrum of compound **4l** in $CDCl_3$.

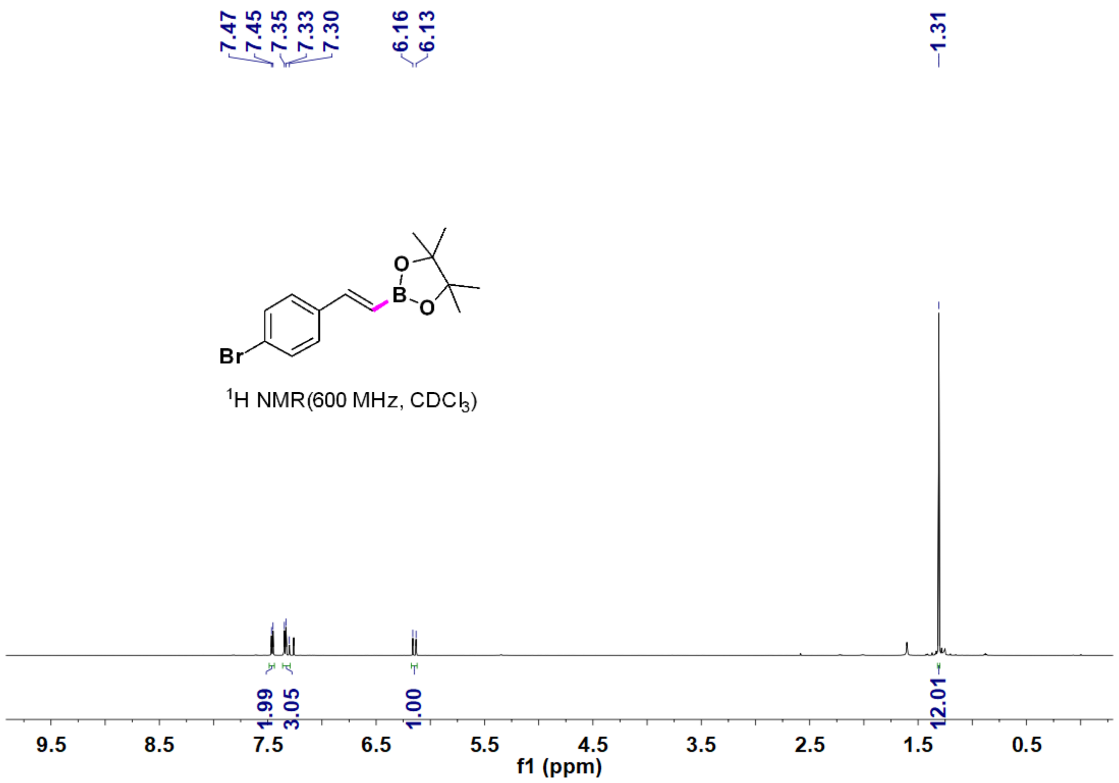

Supplementary Figure 140. Characterization of compound 4m. ^{1}H NMR spectrum of compound **4m** in $CDCl_3$.

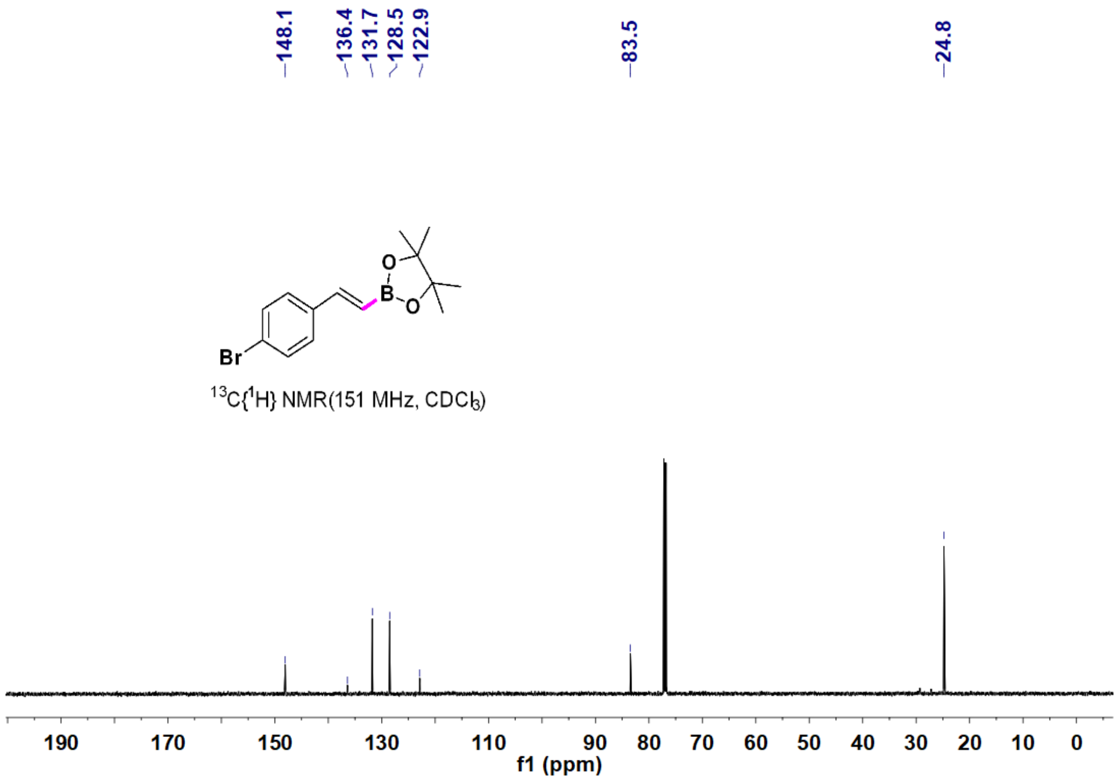

Supplementary Figure 141. Characterization of compound 4m. ^{13}C{^{1}H} NMR spectrum of compound **4m** in $CDCl_3$.

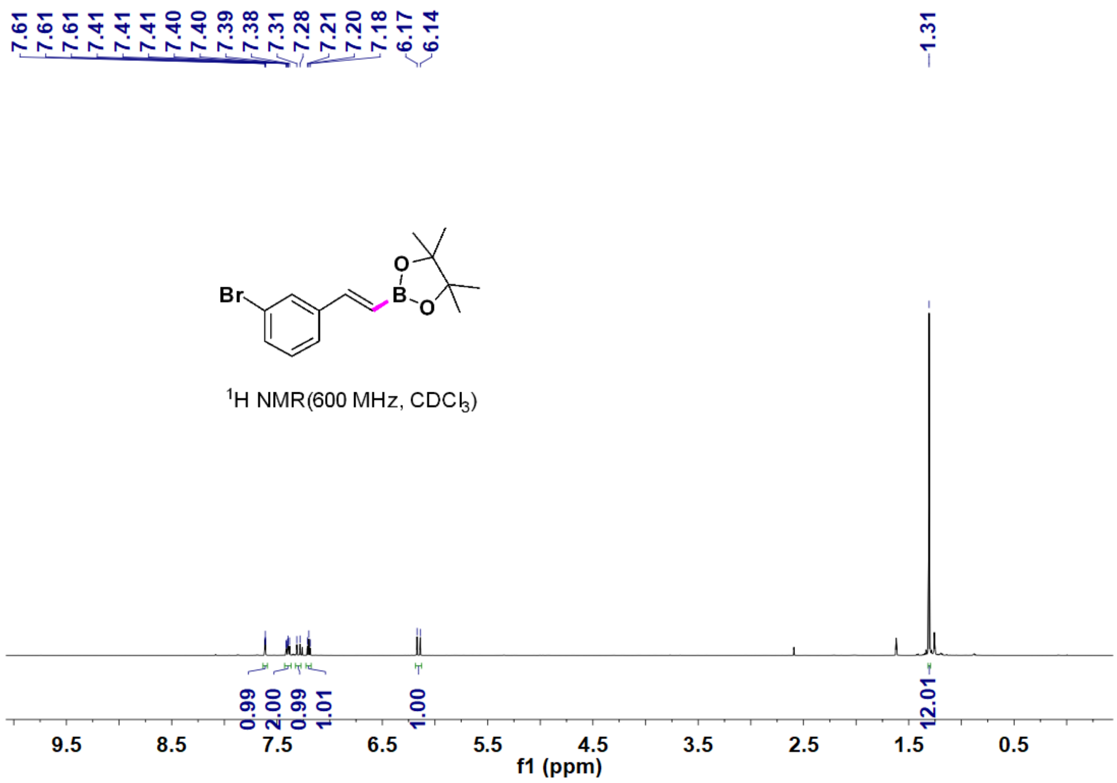

Supplementary Figure 142. Characterization of compound 4n. ^{1}H NMR spectrum of compound **4n** in $CDCl_3$.

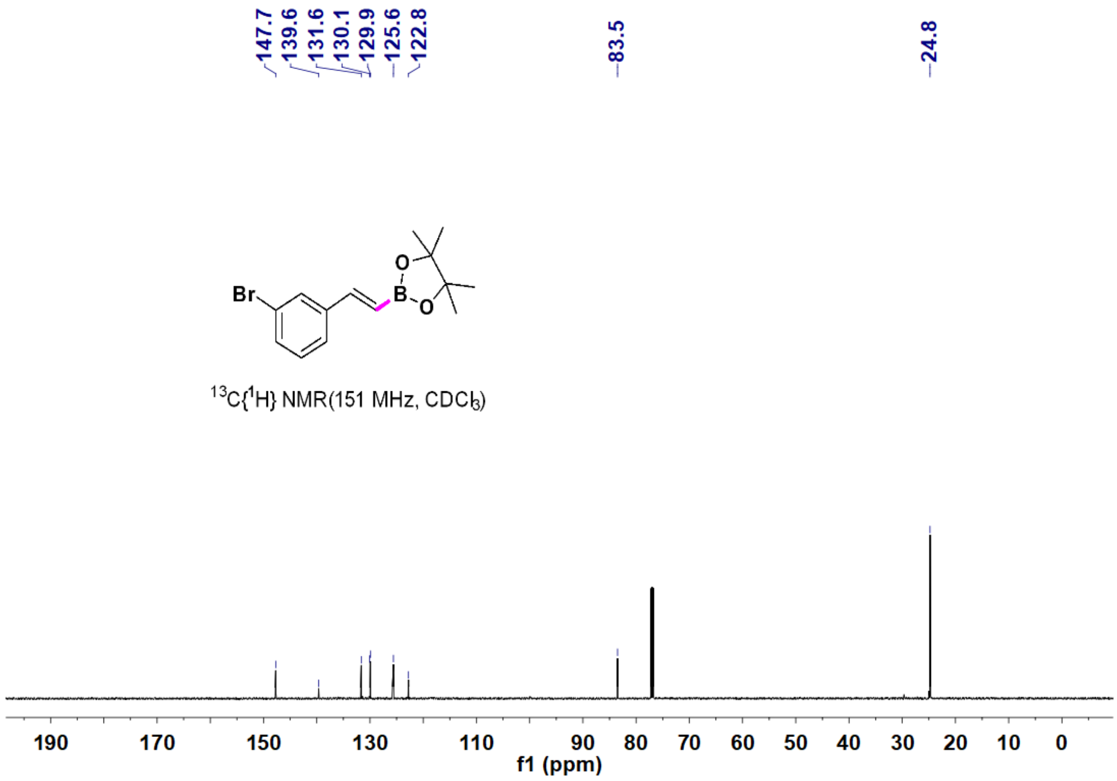

Supplementary Figure 143. Characterization of compound 4n. $^{13}C\{^{1}H\}$ NMR spectrum of compound **4n** in $CDCl_3$.

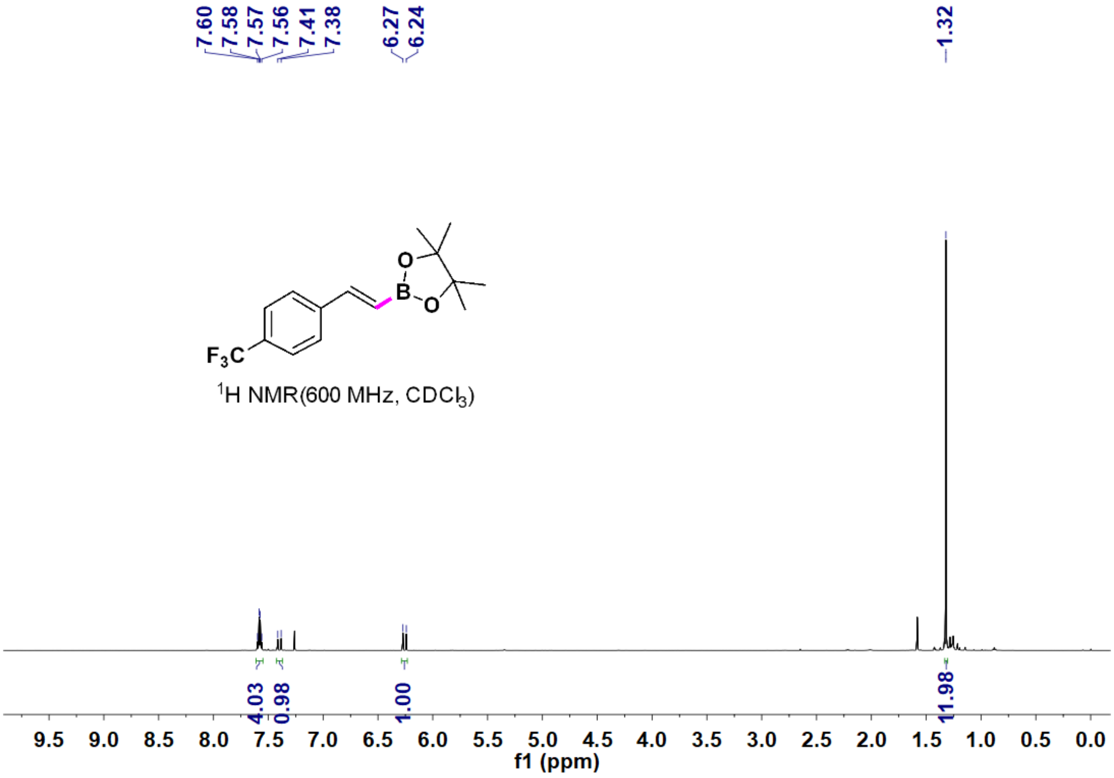

Supplementary Figure 144. Characterization of compound 4o. ^{1}H NMR spectrum of compound **4o** in $CDCl_3$.

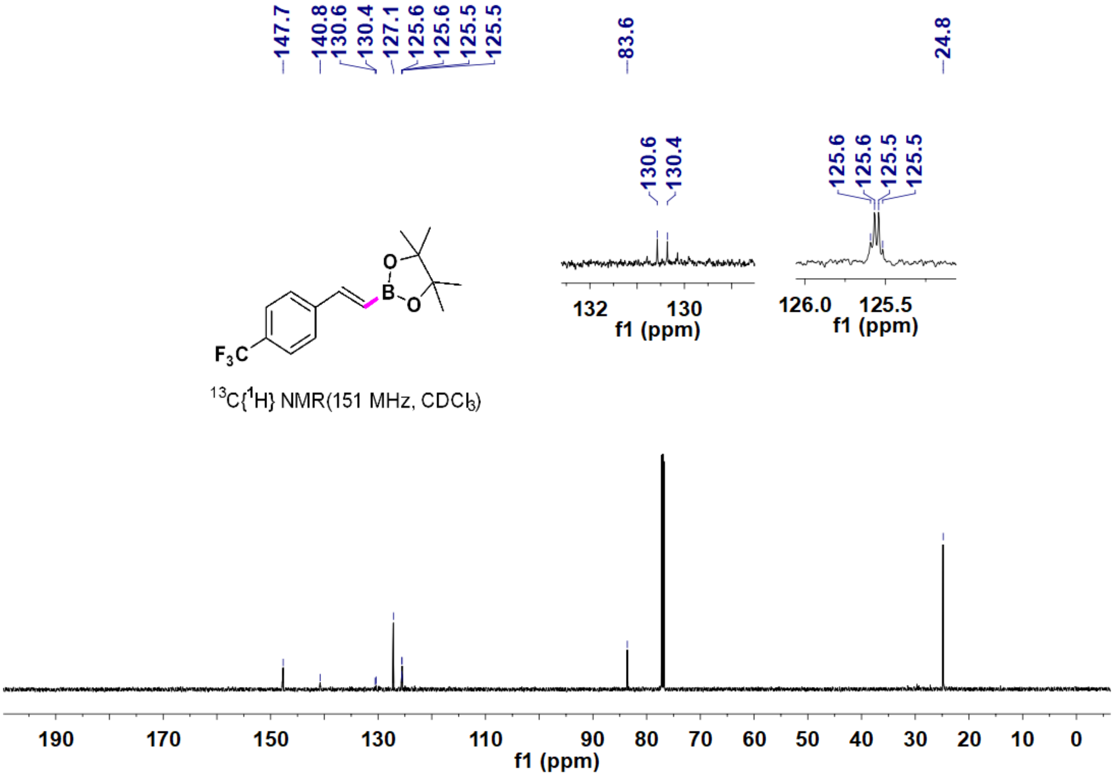

Supplementary Figure 145. Characterization of compound 4o. $^{13}C\{^{1}H\}$ NMR spectrum of compound **4o** in $CDCl_3$.

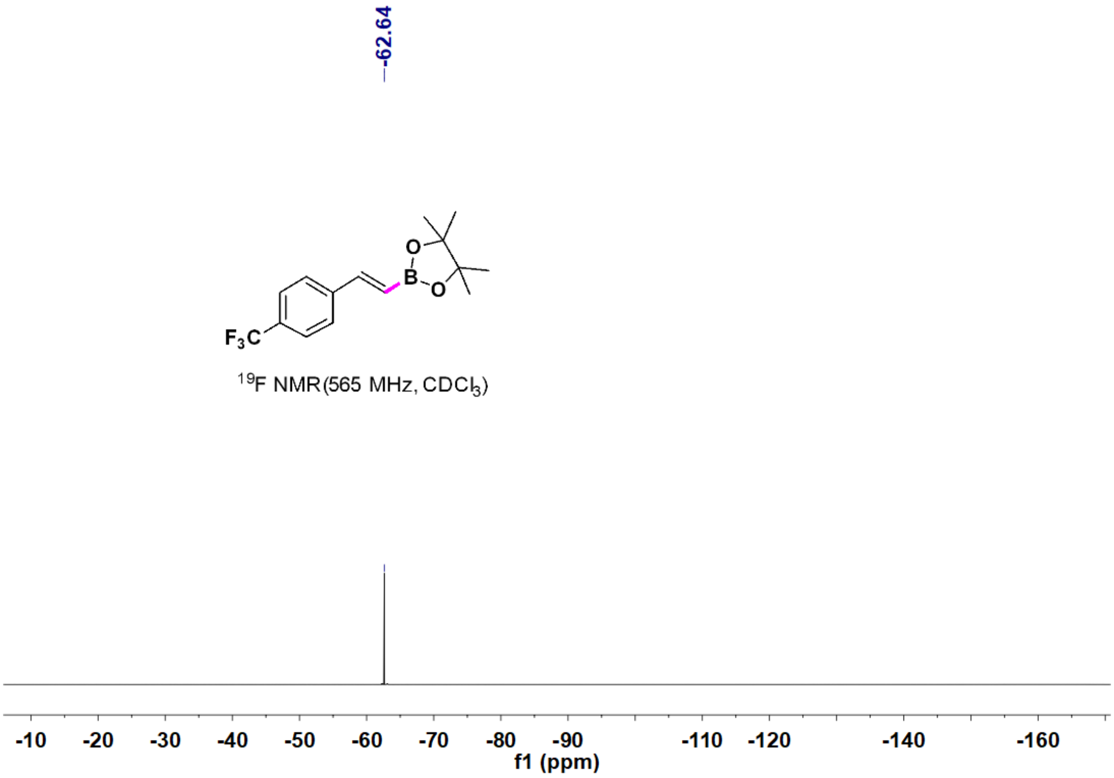

Supplementary Figure 146. Characterization of compound 4o. ^{19}F NMR spectrum of compound **4o** in $CDCl_3$.

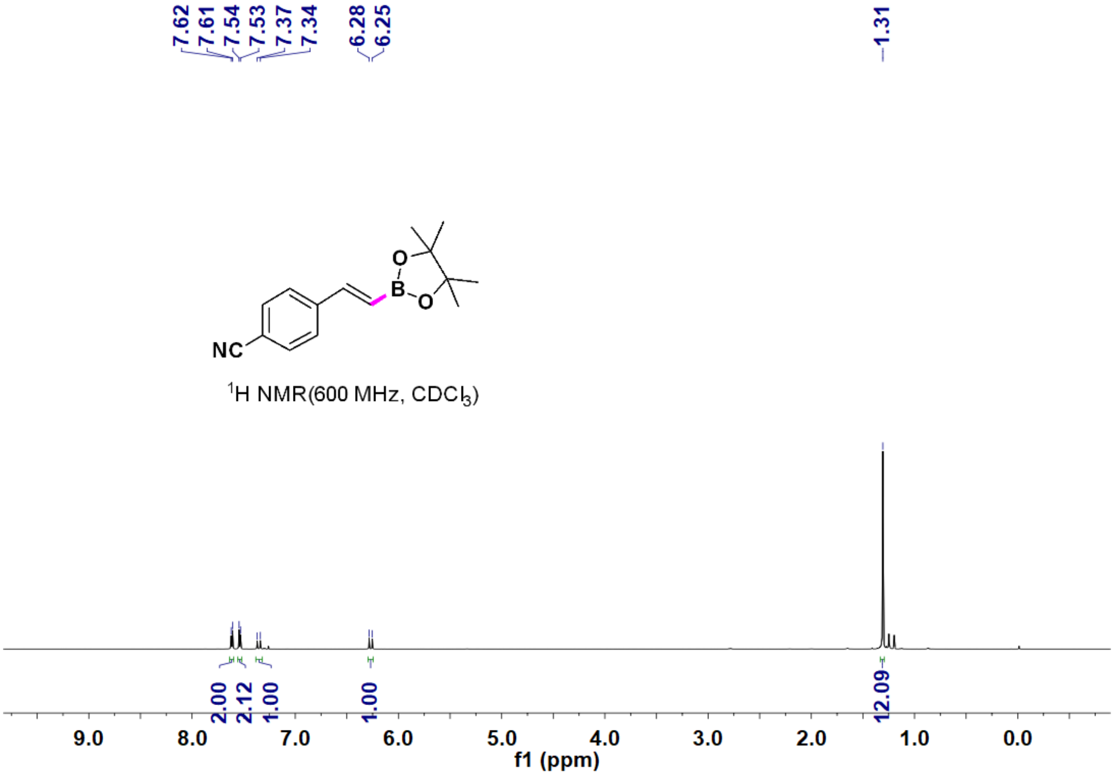

Supplementary Figure 147. Characterization of compound 4p. ^{1}H NMR spectrum of compound **4p** in $CDCl_3$.

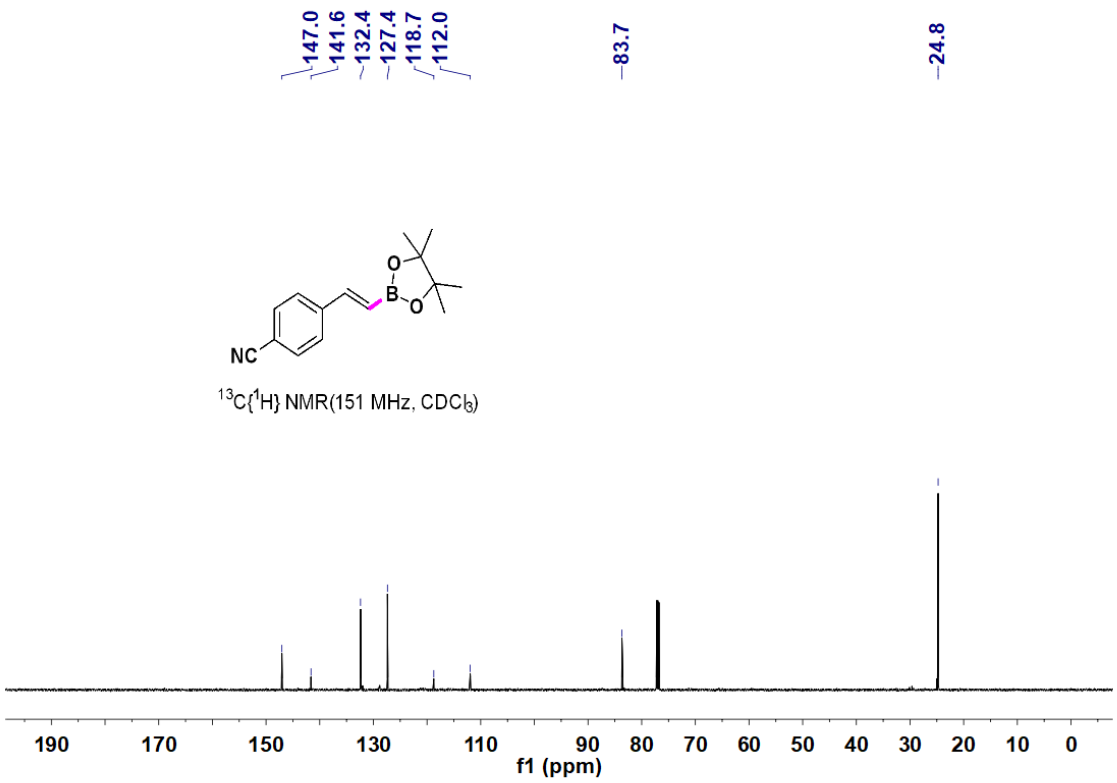

Supplementary Figure 148. Characterization of compound 4p. $^{13}C\{^1H\}$ NMR spectrum of compound **4p** in $CDCl_3$.

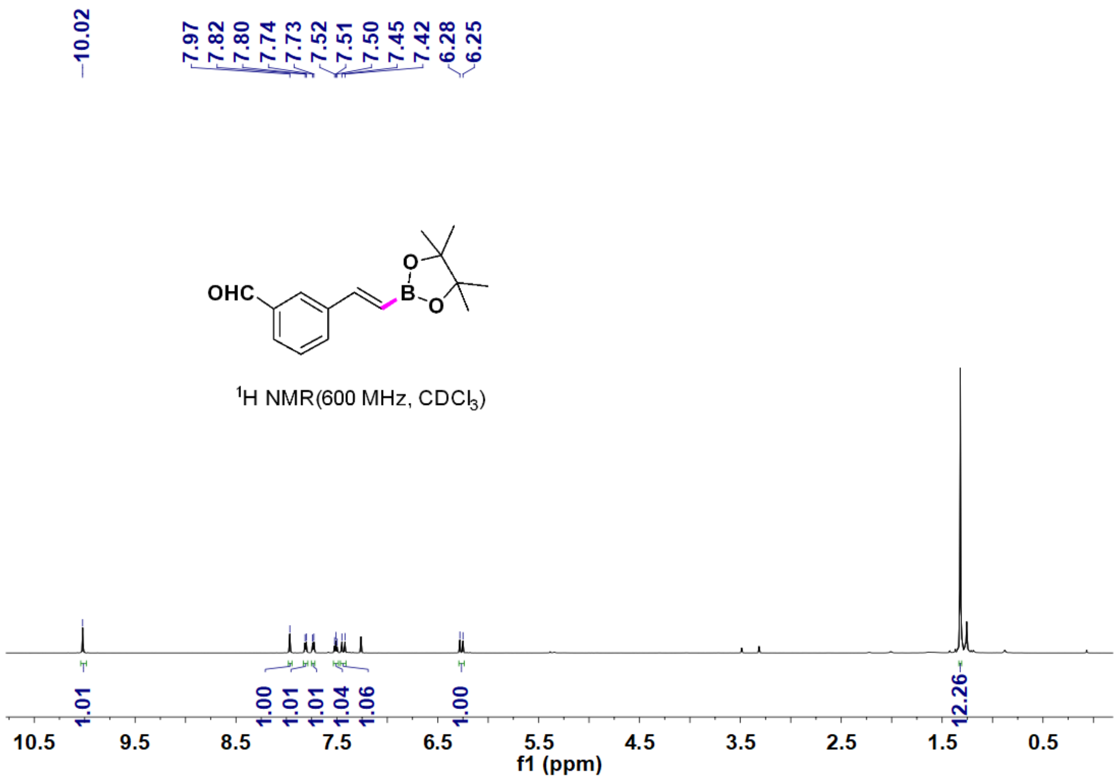

Supplementary Figure 149. Characterization of compound 4q. ^1H NMR spectrum of compound **4q** in $CDCl_3$.

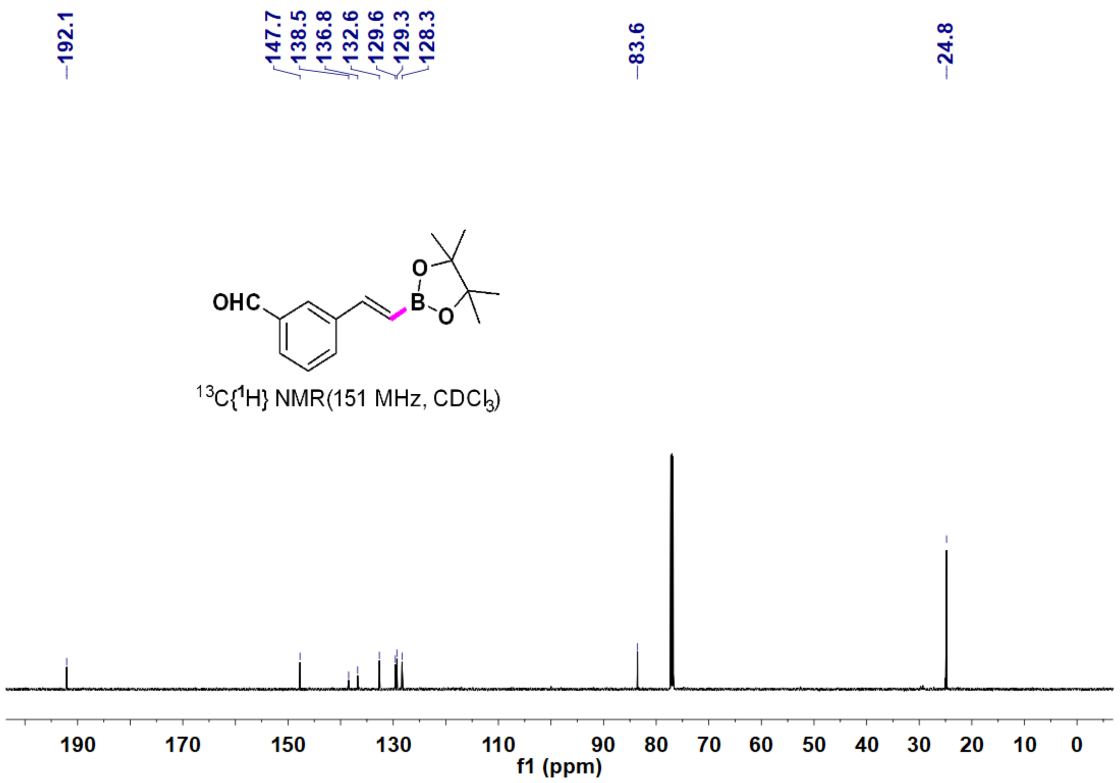

Supplementary Figure 150. Characterization of compound 4q. ^{13}C{^{1}H} NMR spectrum of compound **4q** in $CDCl_3$.

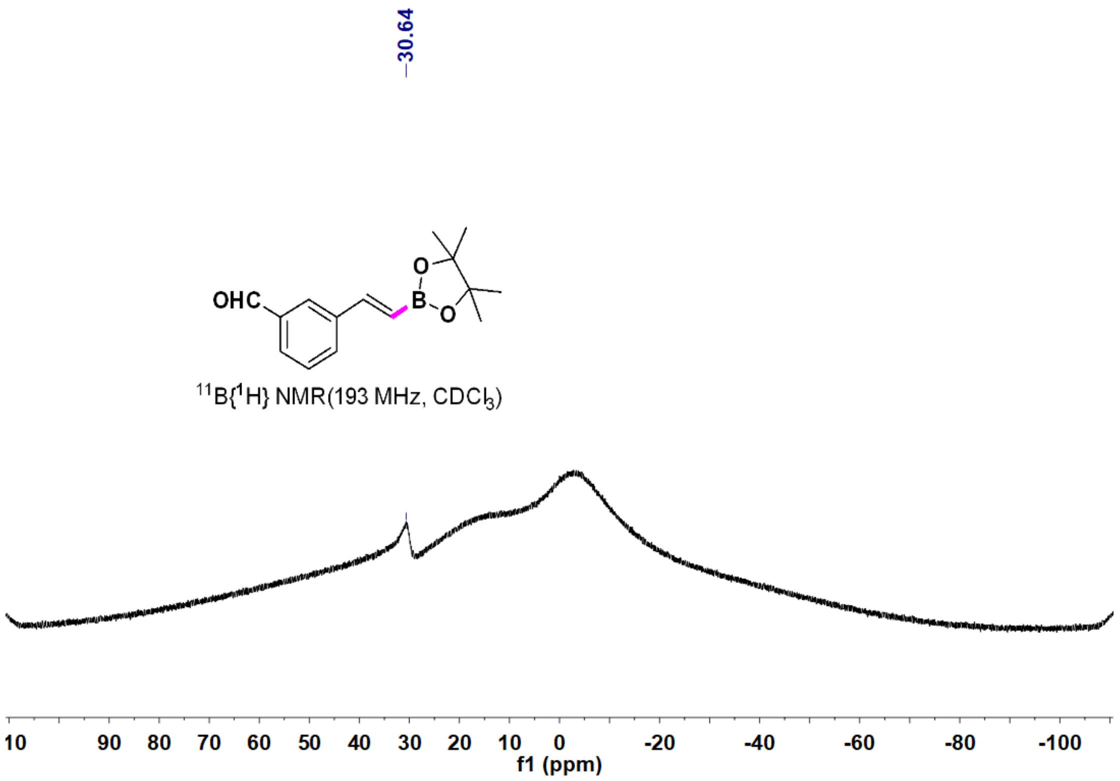

Supplementary Figure 151. Characterization of compound 4q. ^{11}B{^{1}H} NMR spectrum of compound **4q** in $CDCl_3$.

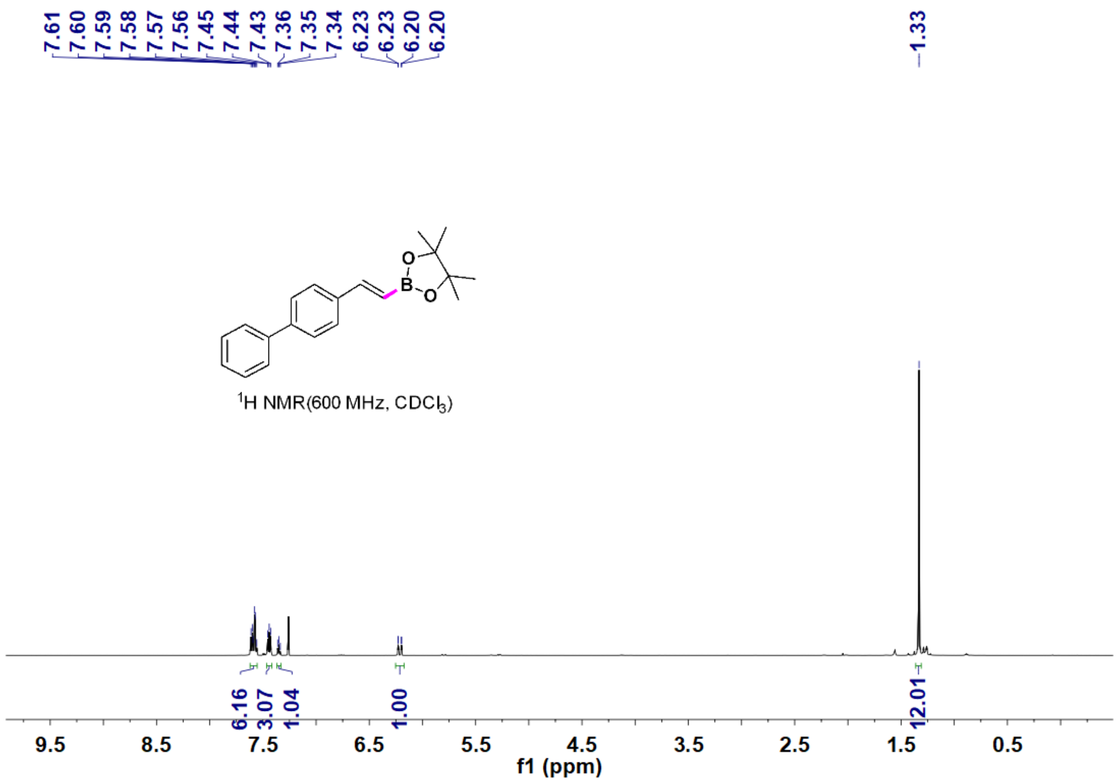

Supplementary Figure 152. Characterization of compound 4r. ^{1}H NMR spectrum of compound **4r** in $CDCl_3$.

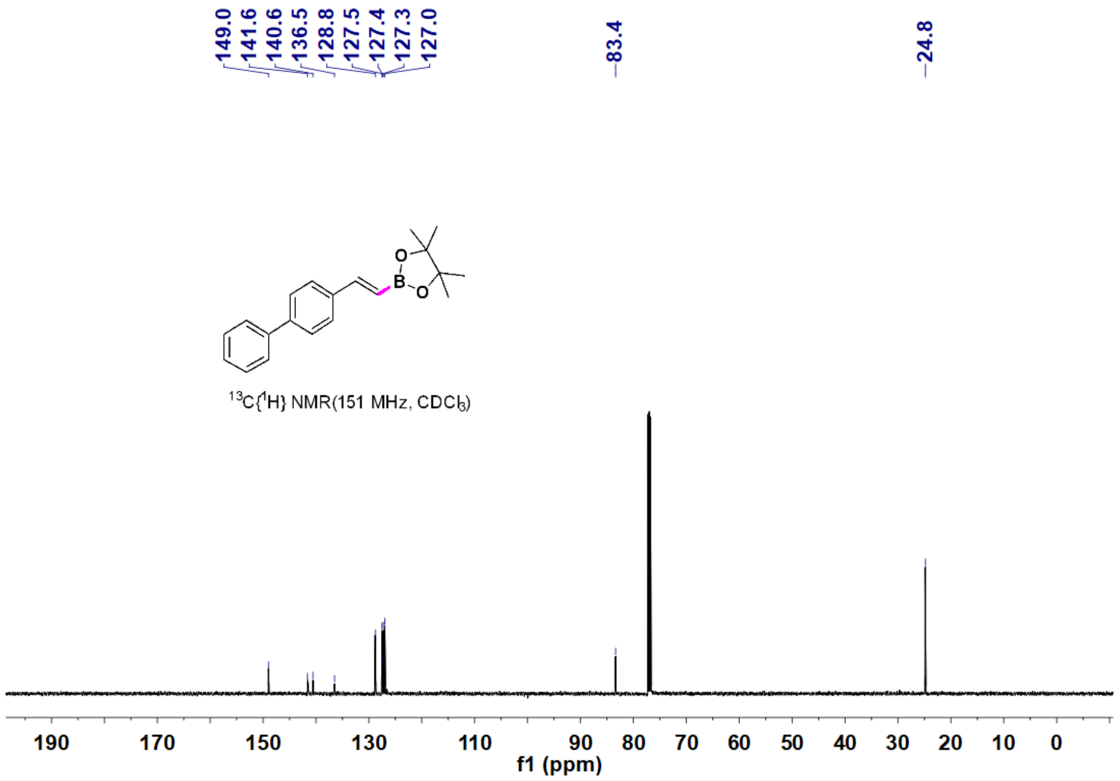

Supplementary Figure 153. Characterization of compound 4r. $^{13}C\{^{1}H\}$ NMR spectrum of compound **4r** in $CDCl_3$.

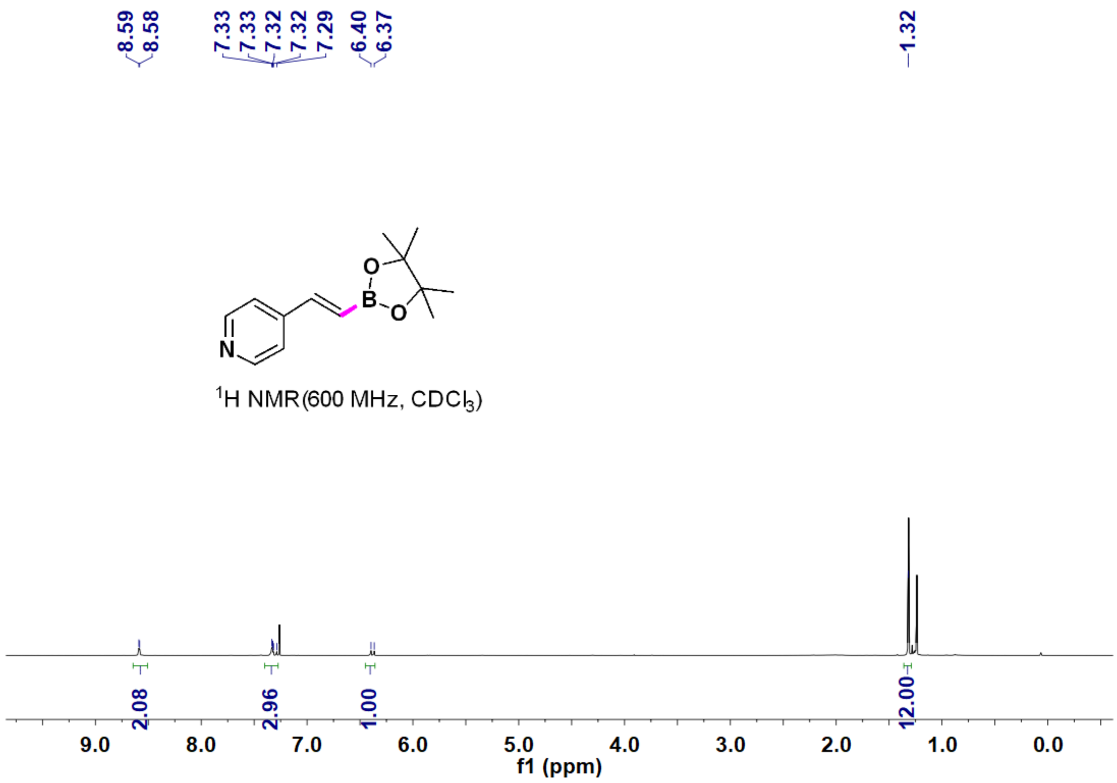

Supplementary Figure 154. Characterization of compound 4s. ^{1}H NMR spectrum of compound **4s** in $CDCl_3$.

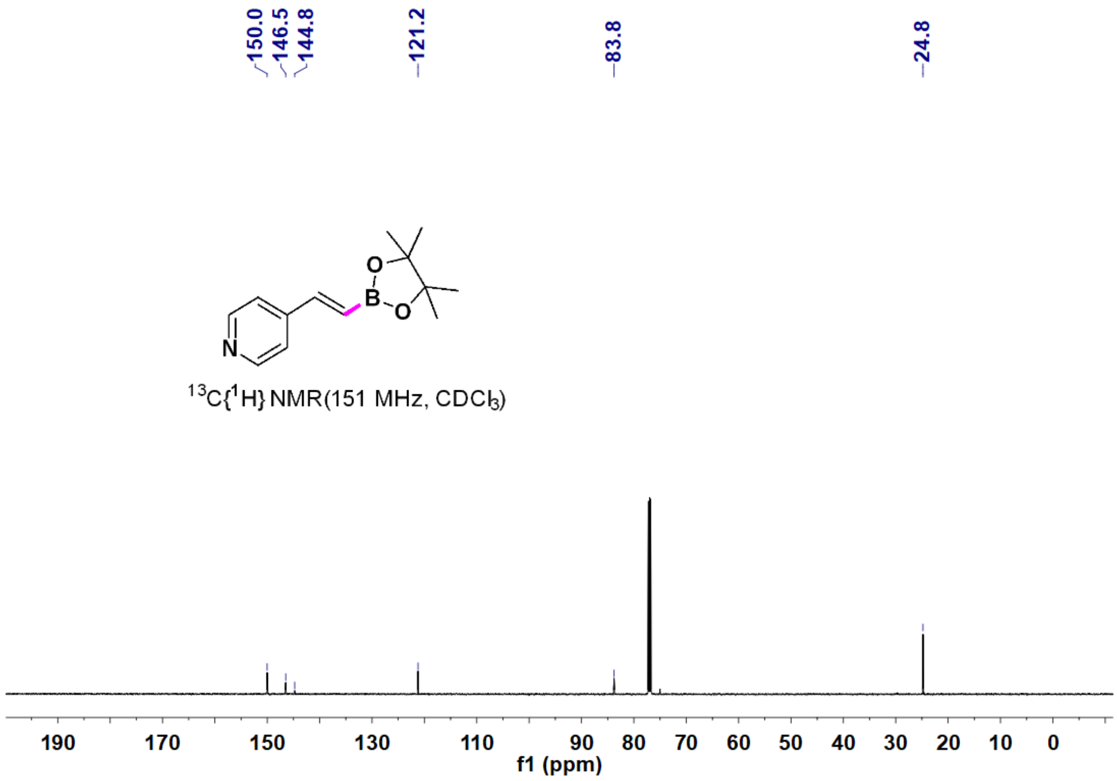

Supplementary Figure 155. Characterization of compound 4s. ^{13}C{^{1}H} NMR spectrum of compound **4s** in $CDCl_3$.

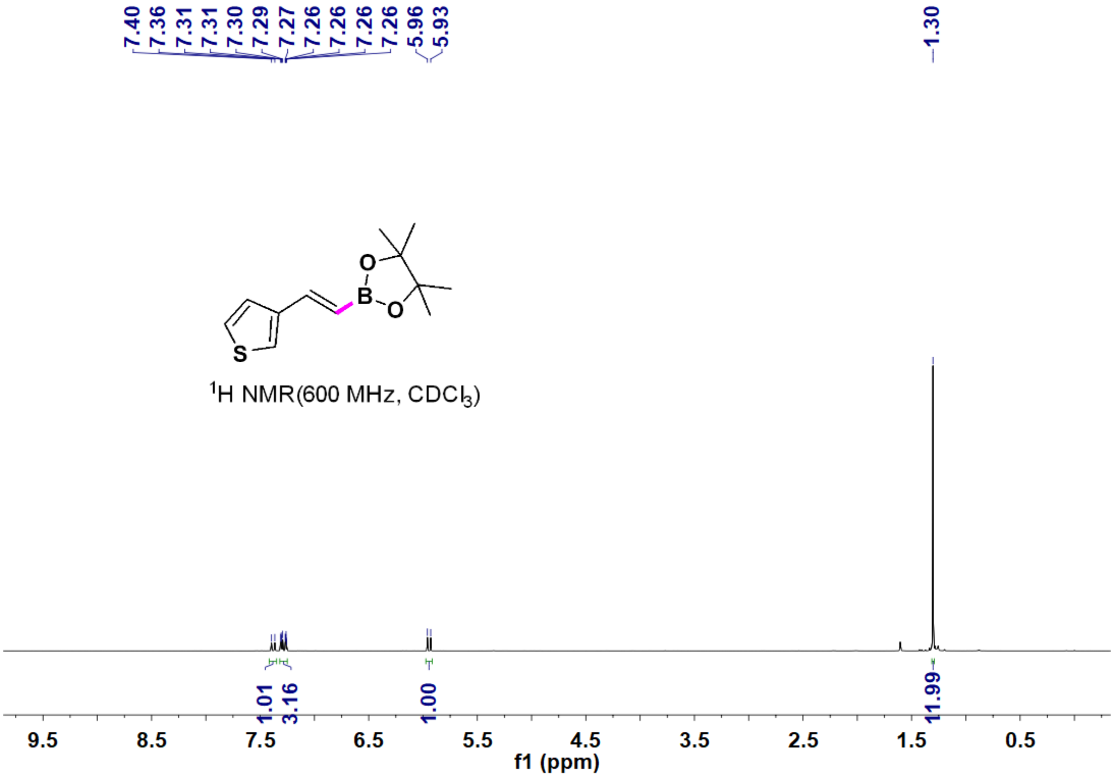

Supplementary Figure 156. Characterization of compound 4t. ^{1}H NMR spectrum of compound **4t** in $CDCl_3$.

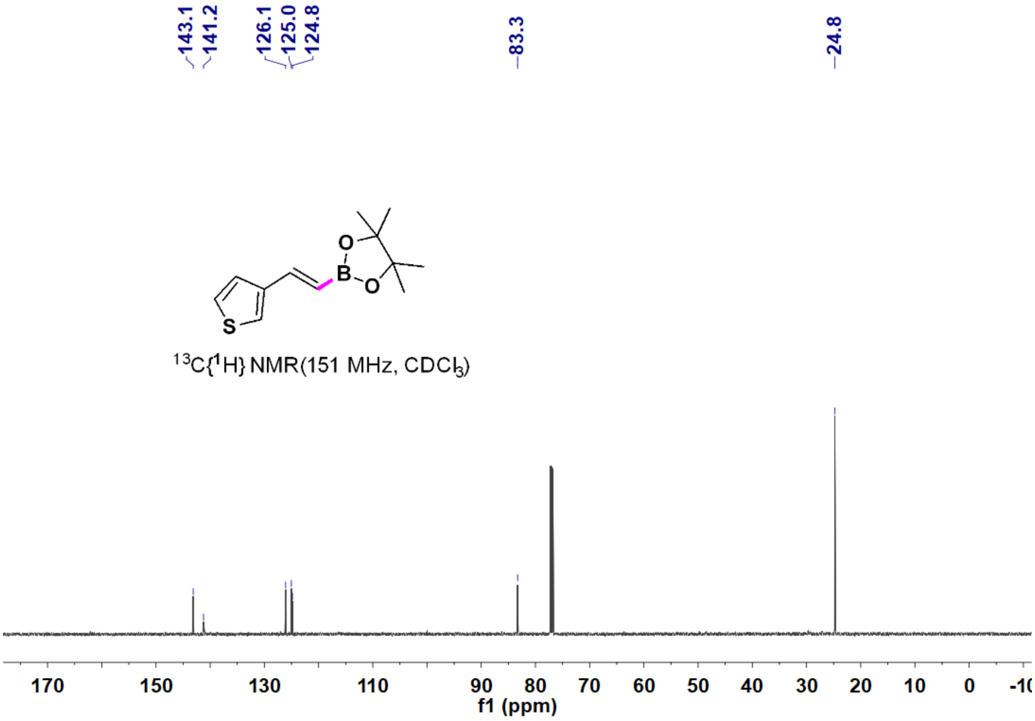

Supplementary Figure 157. Characterization of compound 4t. ^{13}C{^{1}H} NMR spectrum of compound **4t** in $CDCl_3$.

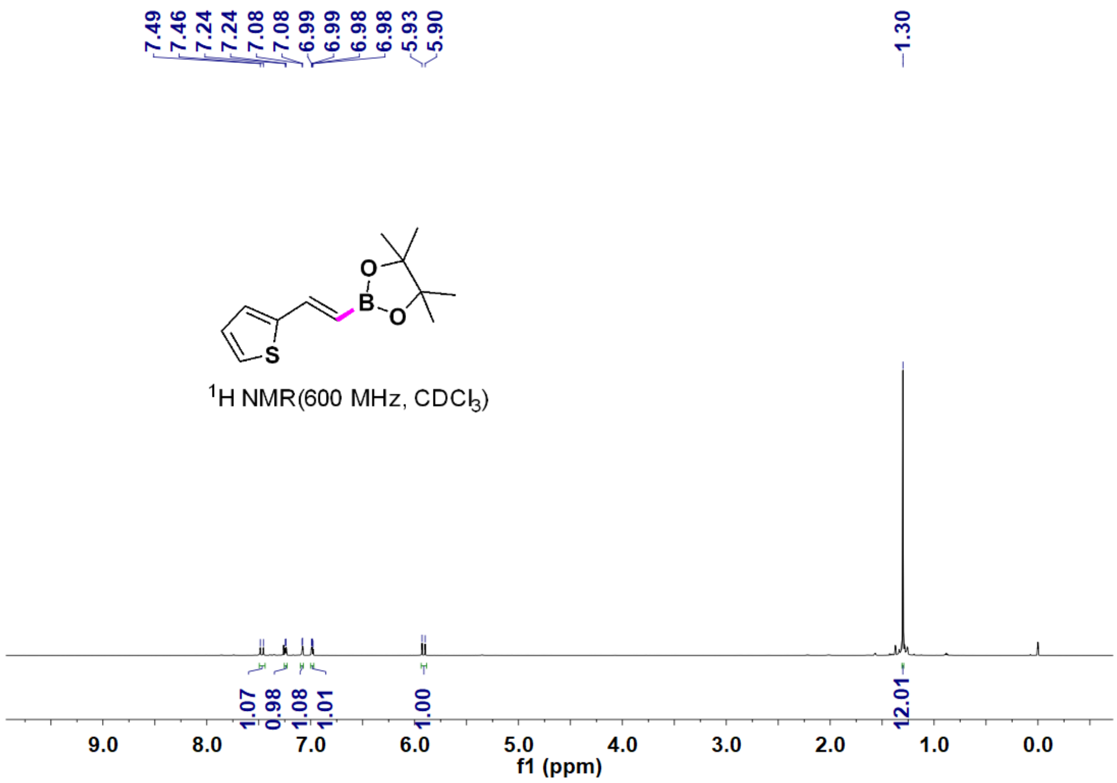

Supplementary Figure 158. Characterization of compound 4u. ^{1}H NMR spectrum of compound **4u** in $CDCl_3$.

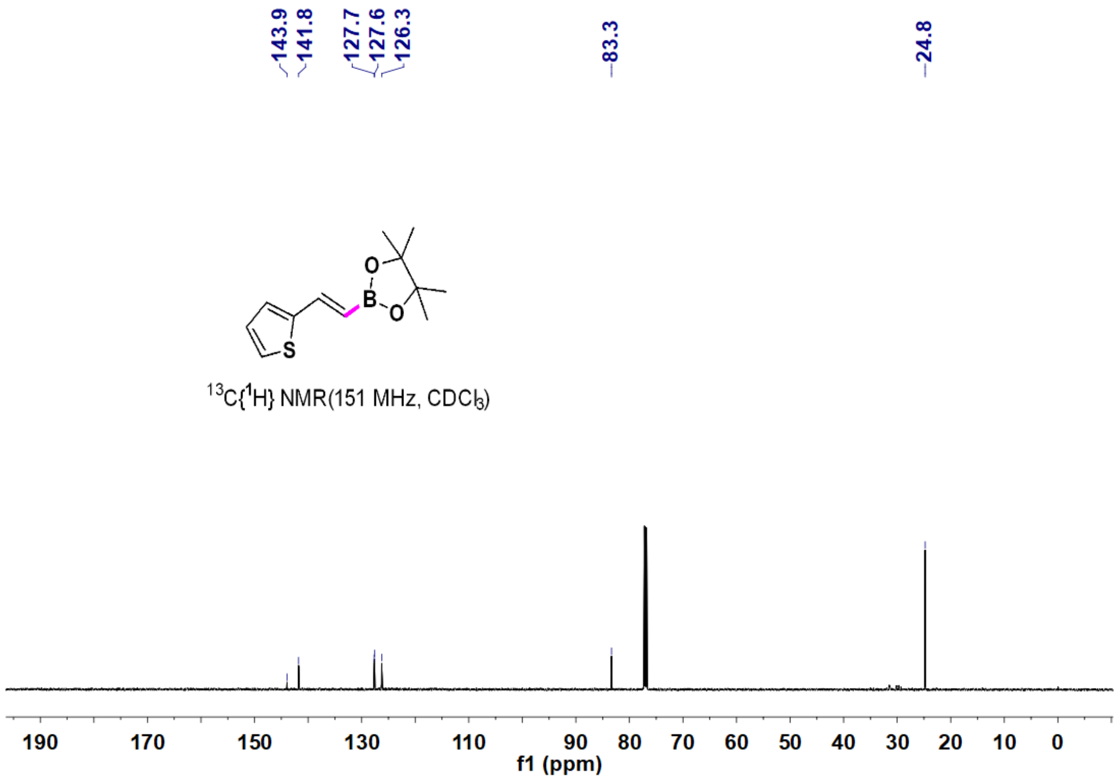

Supplementary Figure 159. Characterization of compound 4u. $^{13}C\{^1H\}$ NMR spectrum of compound **4u** in $CDCl_3$.

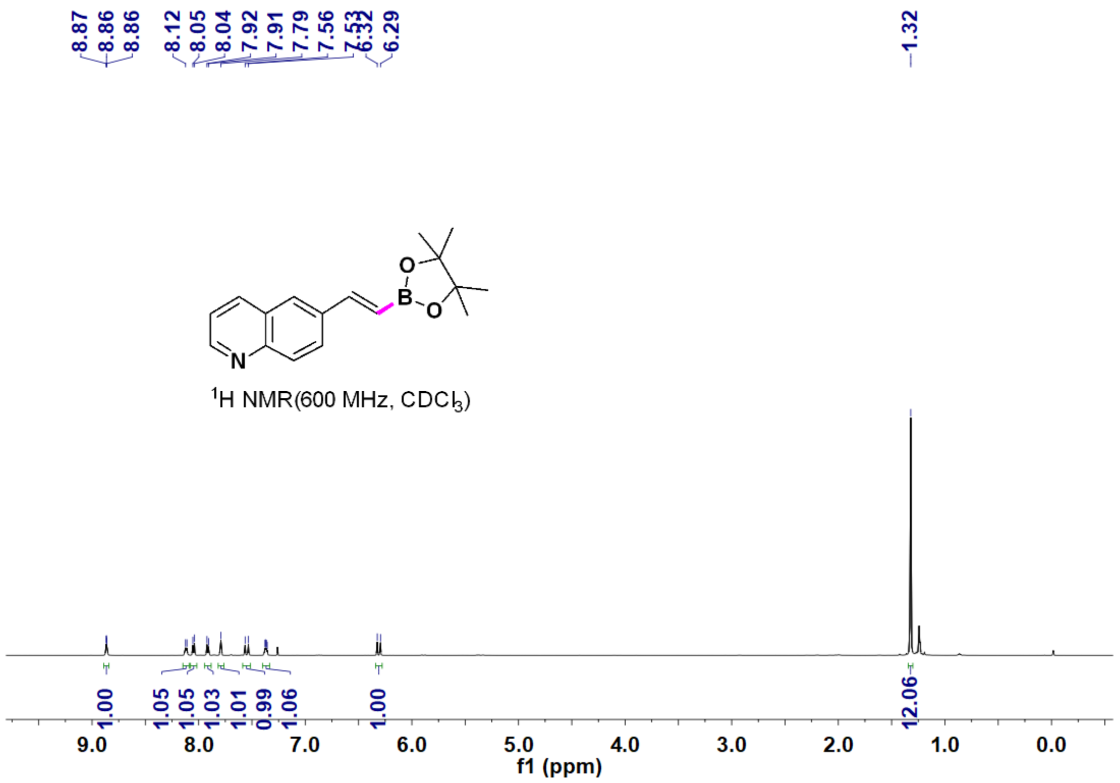

Supplementary Figure 160. Characterization of compound 4v. ^{1}H NMR spectrum of compound **4v** in $CDCl_3$.

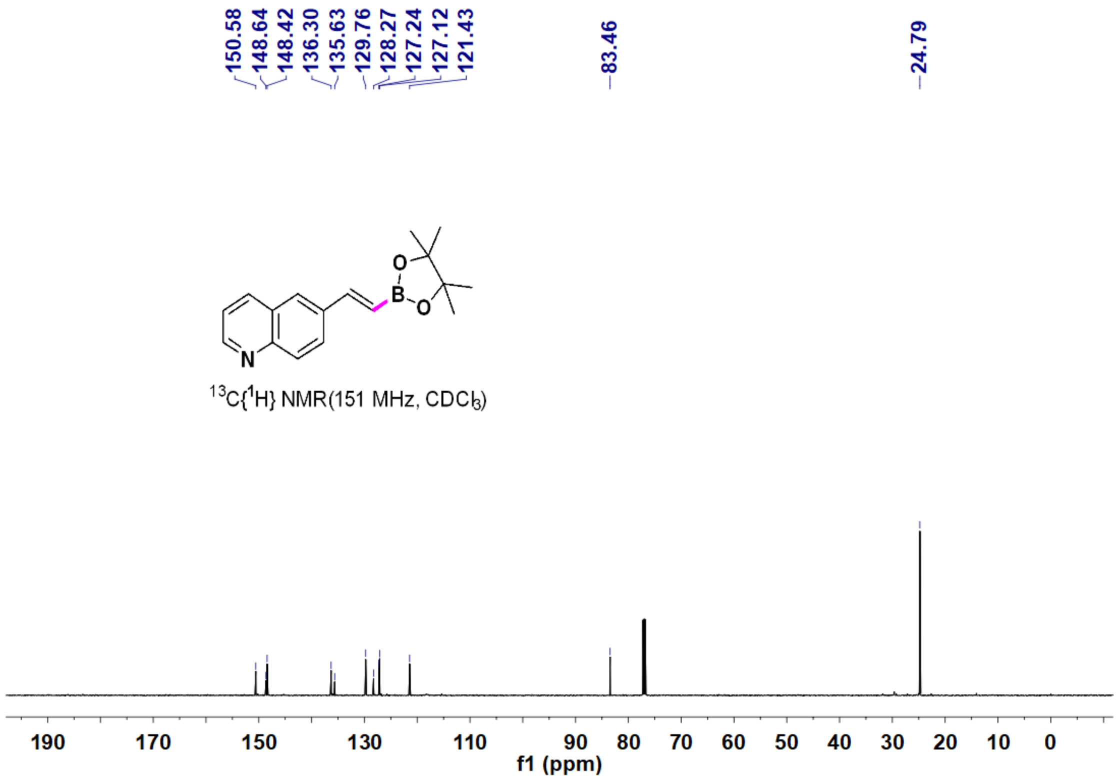

Supplementary Figure 161. Characterization of compound 4v. $^{13}C\{^{1}H\}$ NMR spectrum of compound **4v** in $CDCl_3$.

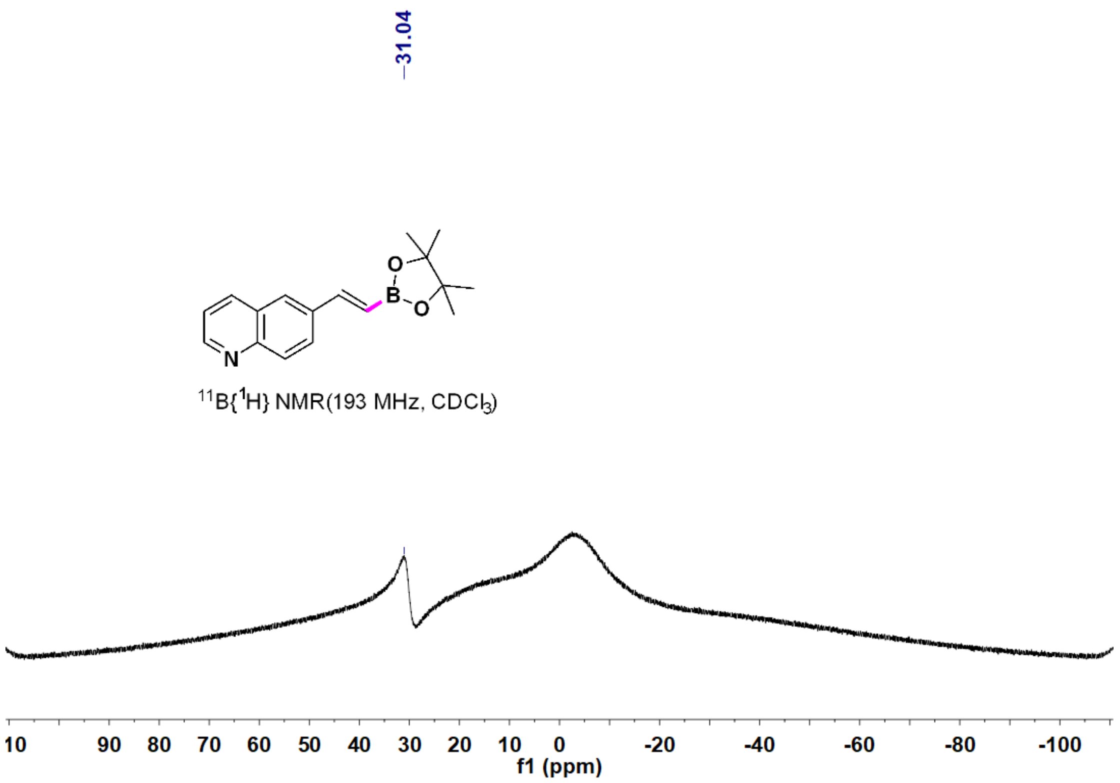

Supplementary Figure 162. Characterization of compound 4v. $^{11}B\{^{1}H\}$ NMR spectrum of compound **4v** in $CDCl_3$.

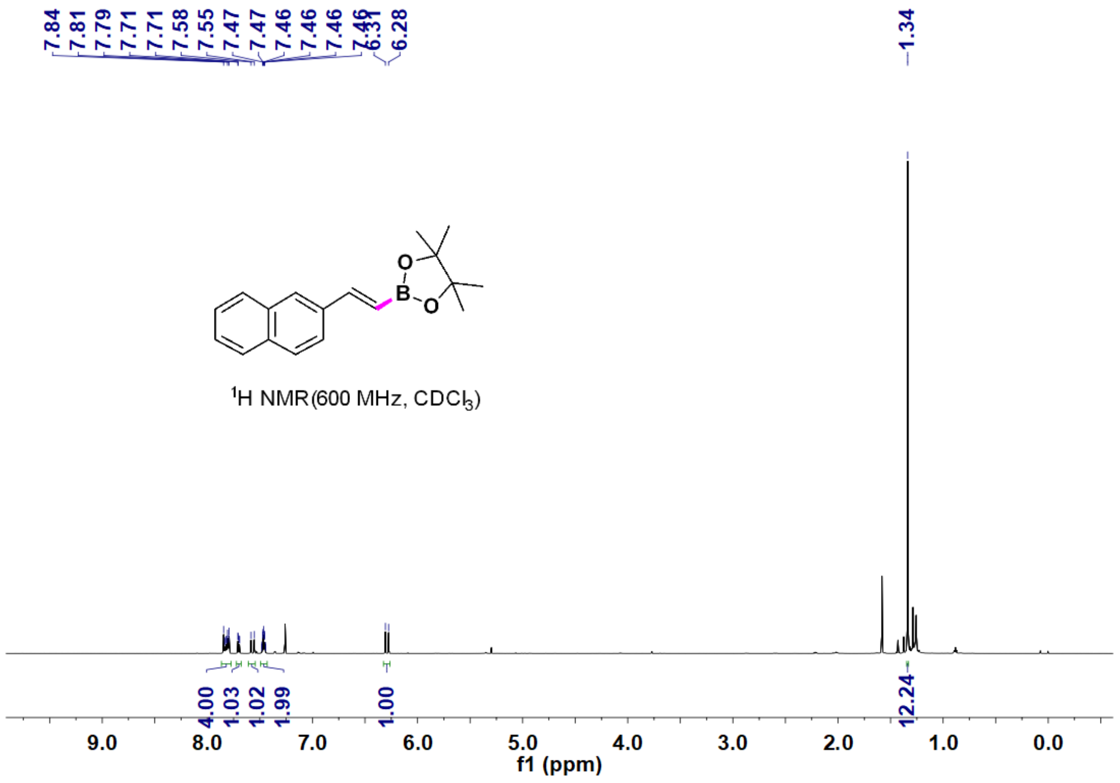

Supplementary Figure 163. Characterization of compound 4w. ^{1}H NMR spectrum of compound **4w** in $CDCl_3$.

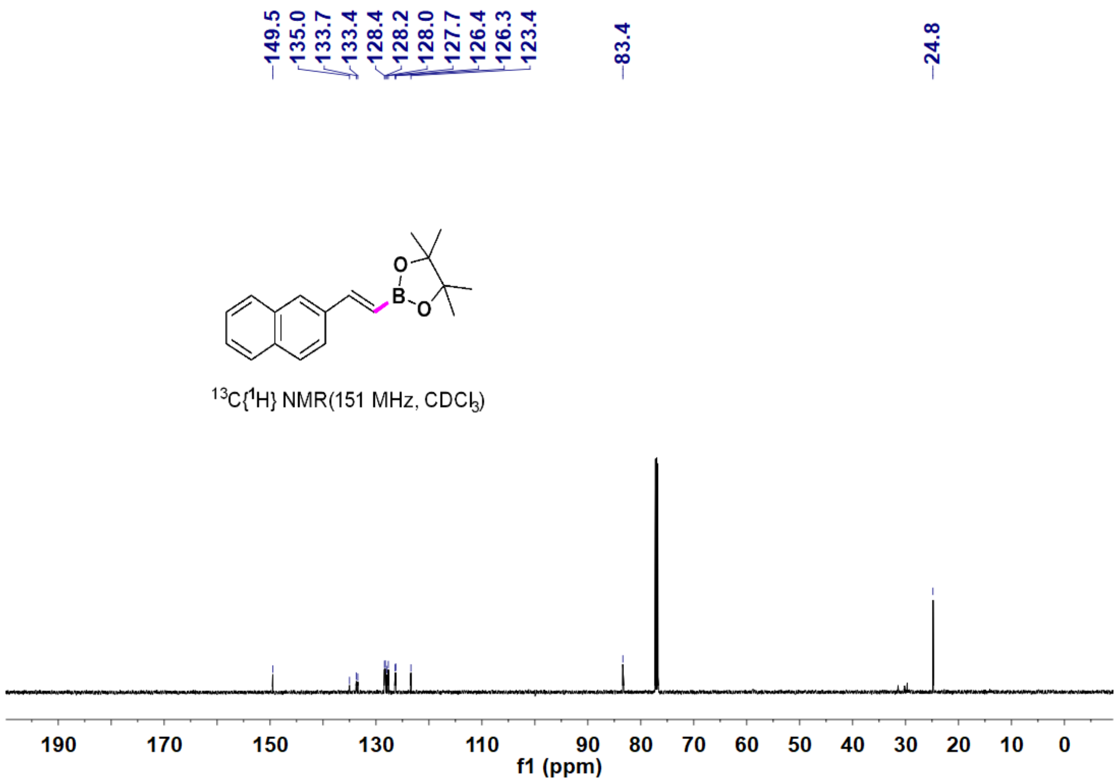

Supplementary Figure 164. Characterization of compound 4w. $^{13}C\{^{1}H\}$ NMR spectrum of compound **4w** in $CDCl_3$.

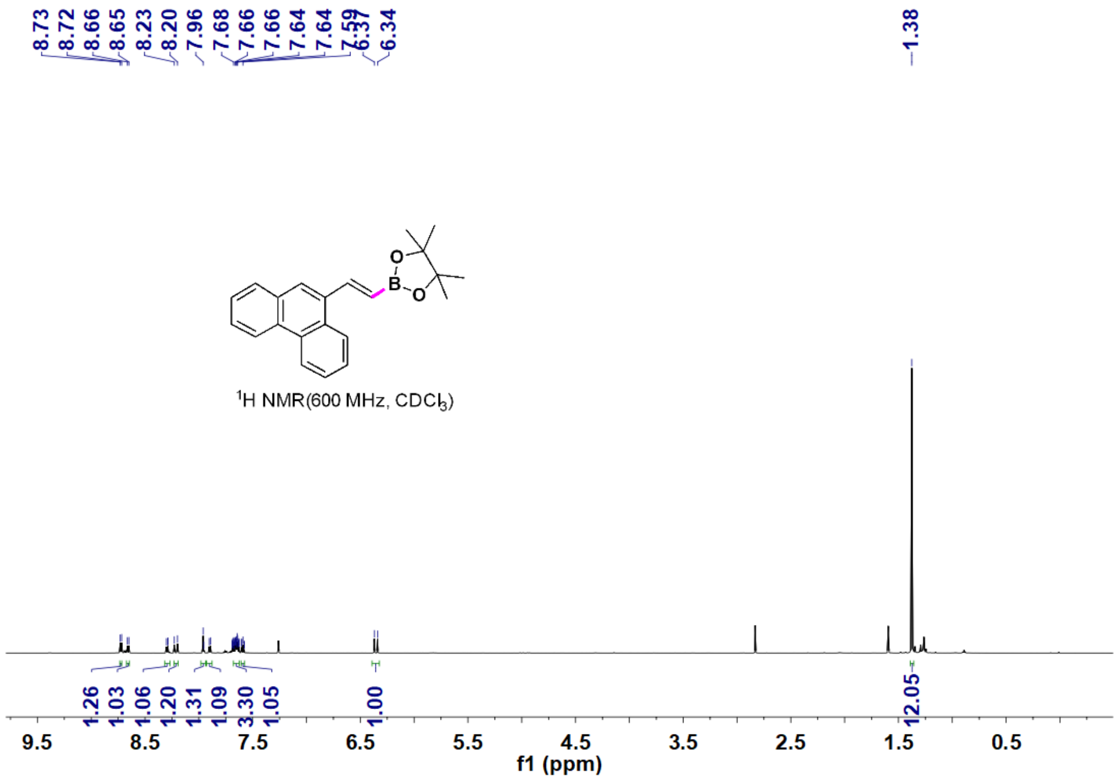

Supplementary Figure 165. Characterization of compound 4x. ^{1}H NMR spectrum of compound **4x** in $CDCl_3$.

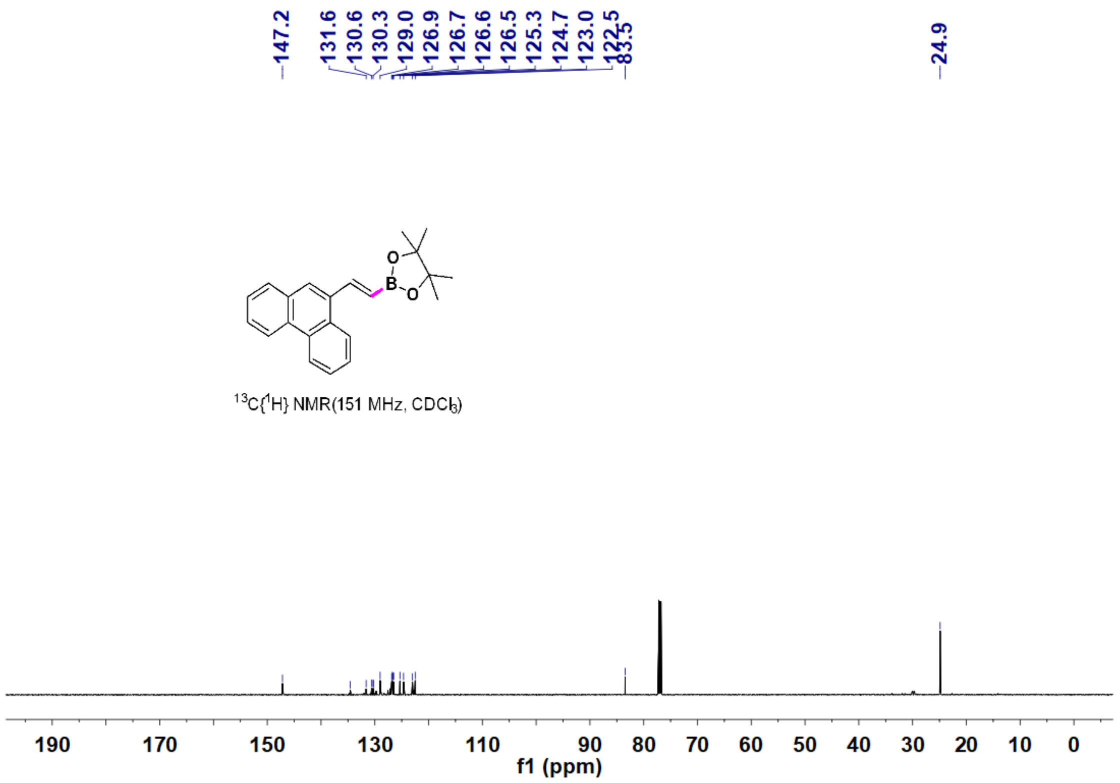

Supplementary Figure 166. Characterization of compound 4x. $^{13}C\{^1H\}$ NMR spectrum of compound **4x** in $CDCl_3$.

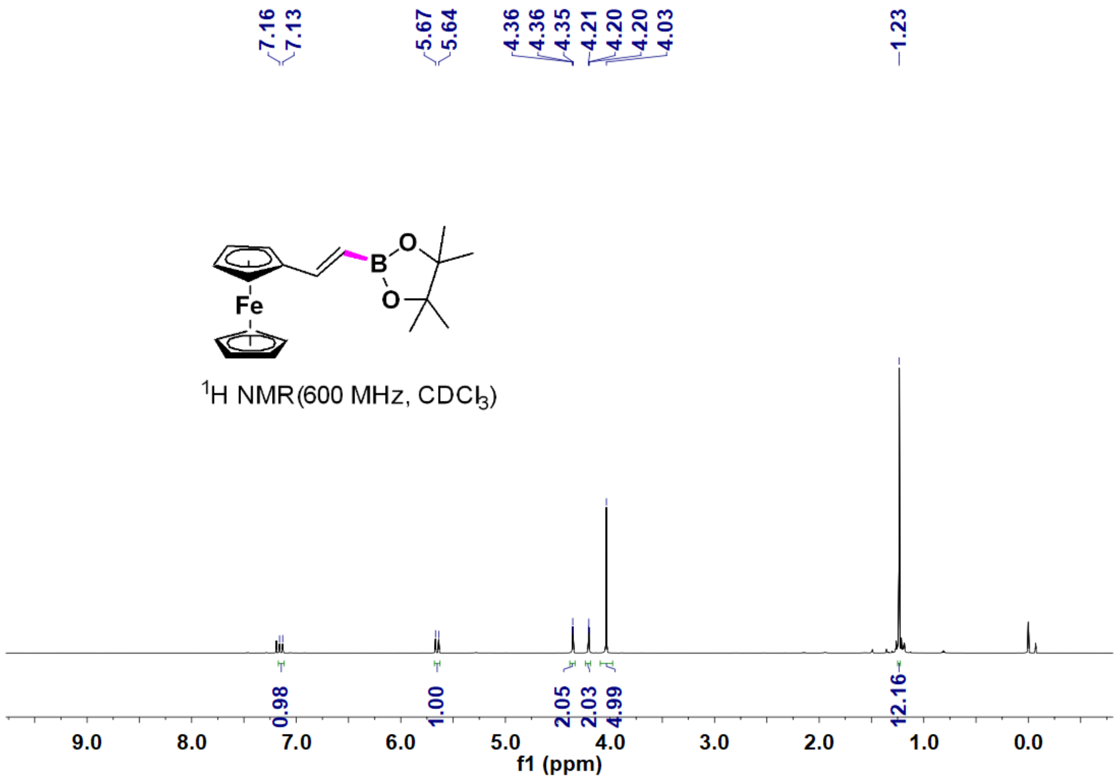

Supplementary Figure 167. Characterization of compound 4y. ^1H NMR spectrum of compound **4y** in $CDCl_3$.

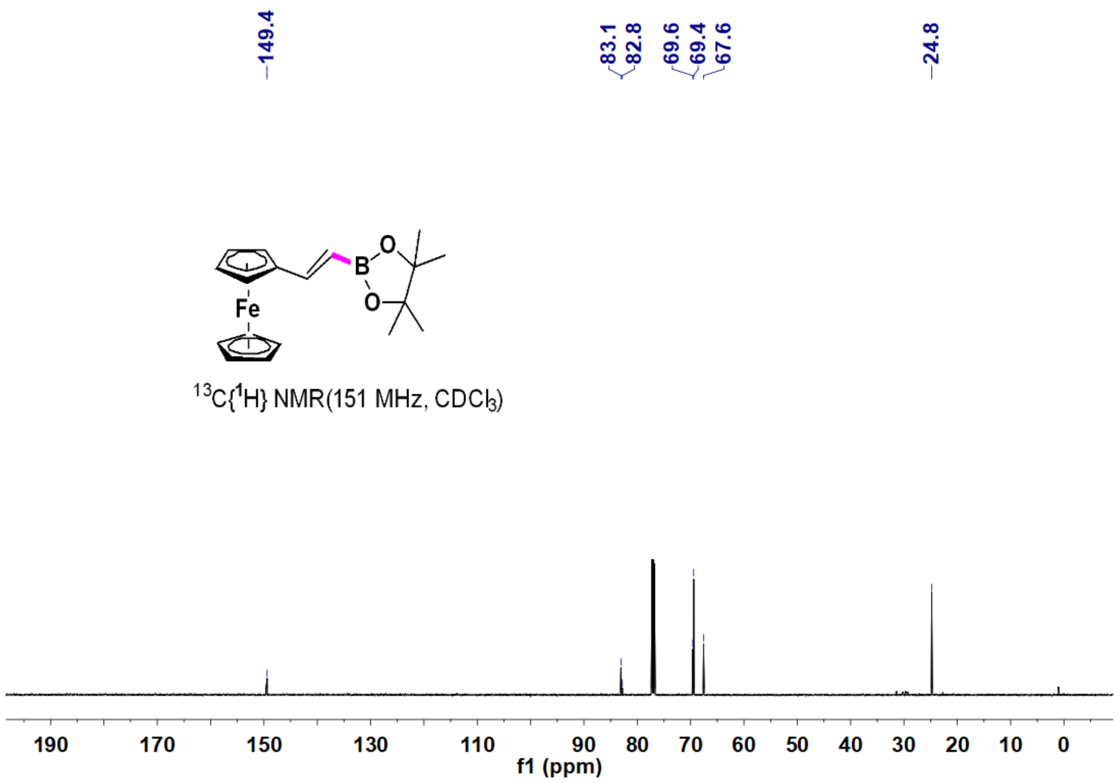

Supplementary Figure 168. Characterization of compound 4y. $^{13}C\{^{1}H\}$ NMR spectrum of compound **4y** in $CDCl_3$.

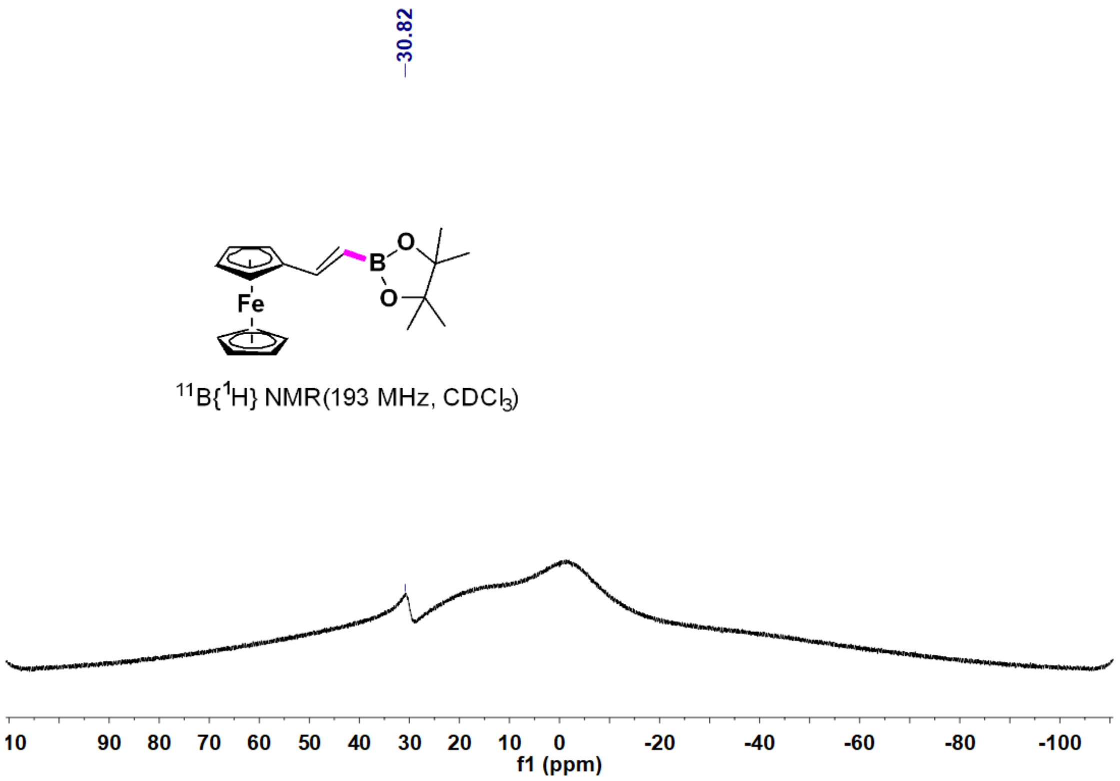

Supplementary Figure 169. Characterization of compound 4y. $^{11}B\{^{1}H\}$ NMR spectrum of compound **4y** in $CDCl_3$.

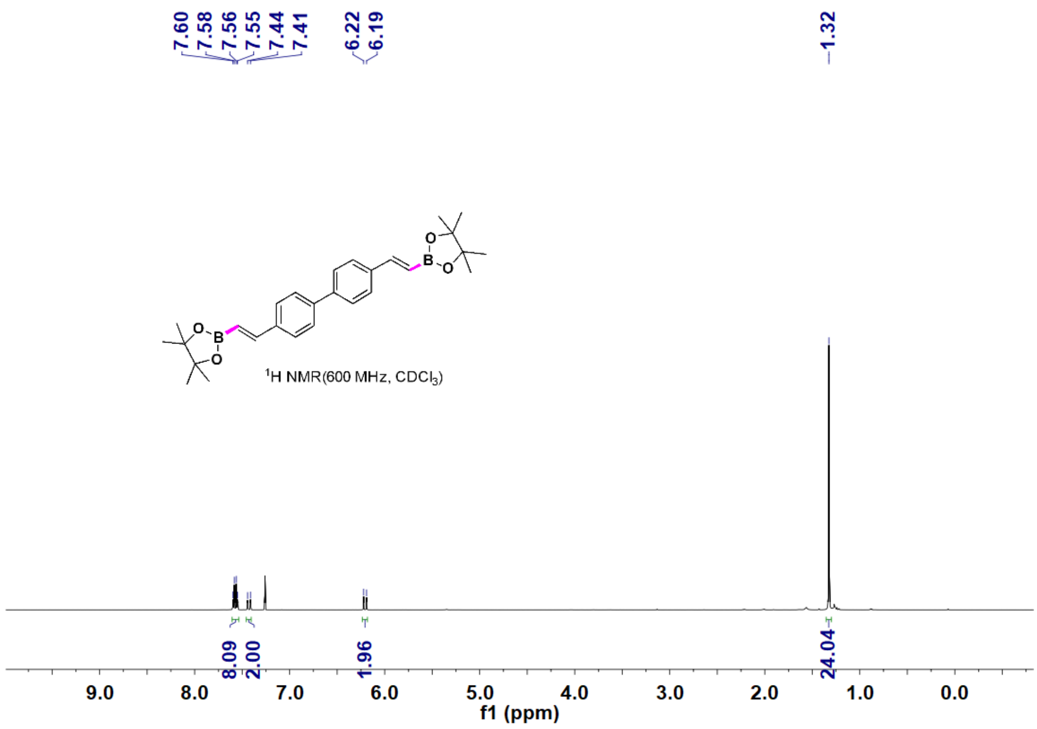

Supplementary Figure 170. Characterization of compound 4z. ^{1}H NMR spectrum of compound **4z** in $CDCl_3$.

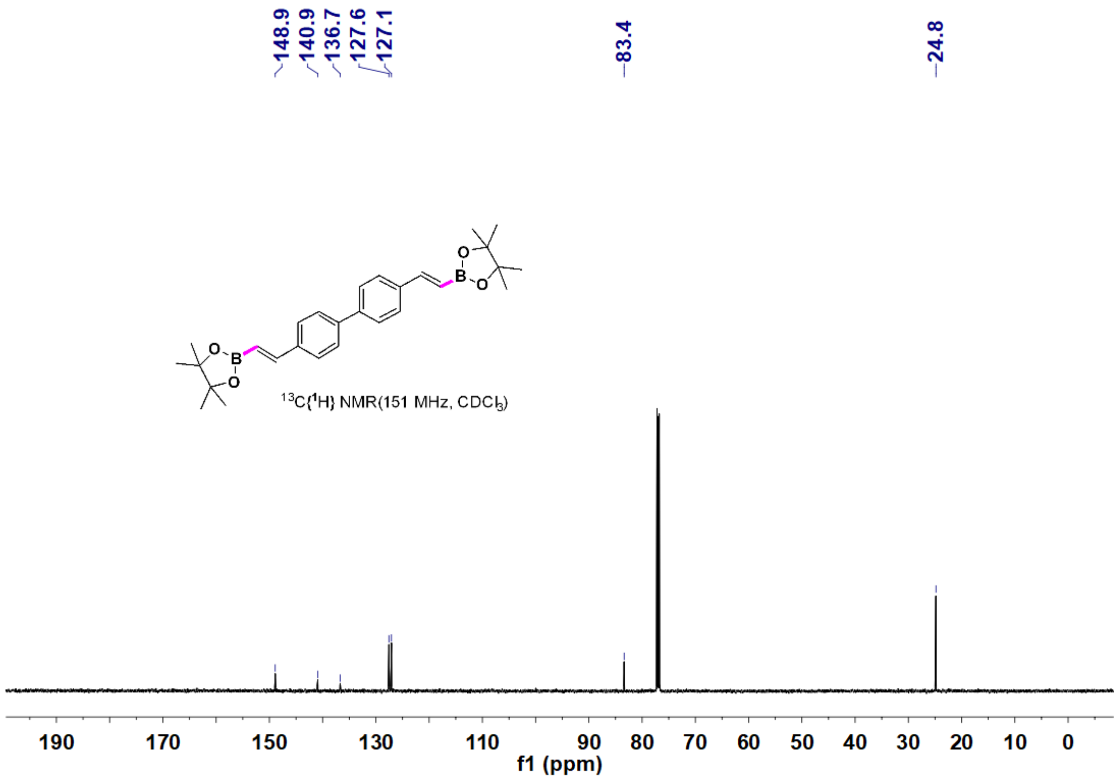

Supplementary Figure 171. Characterization of compound 4z. $^{13}C\{^{1}H\}$ NMR spectrum of compound **4z** in $CDCl_3$.

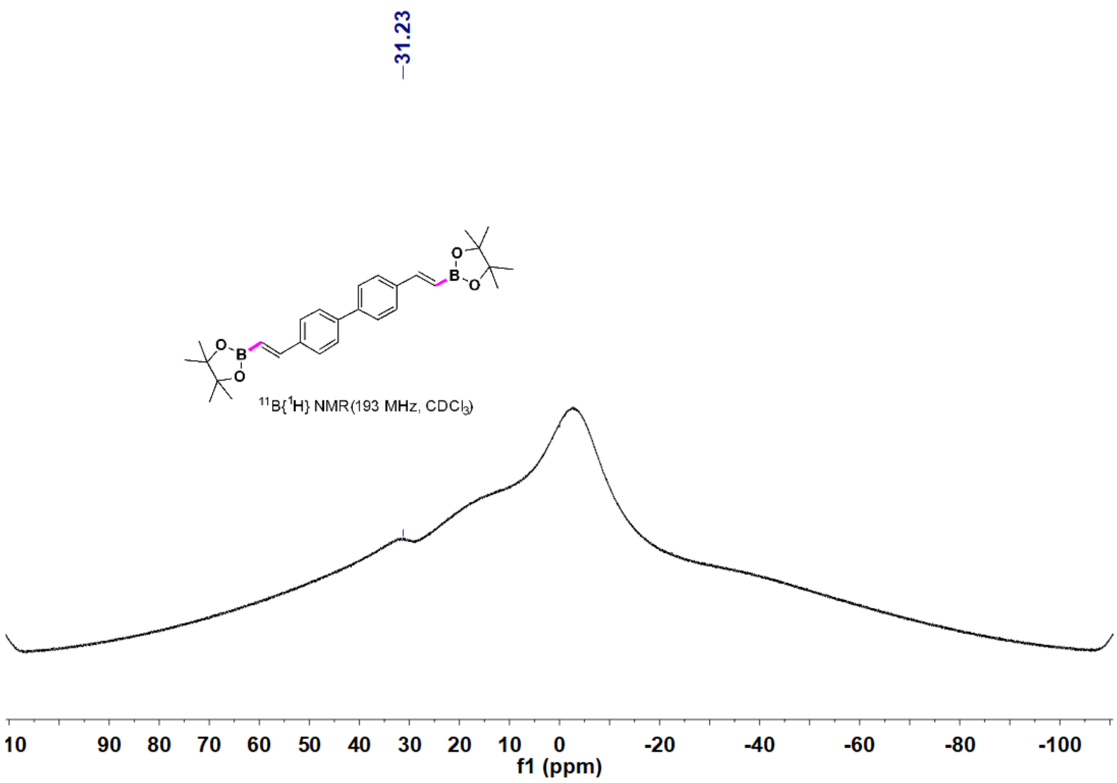

Supplementary Figure 172. Characterization of compound 4z. $^{11}B\{^1H\}$ NMR spectrum of compound **4z** in $CDCl_3$.

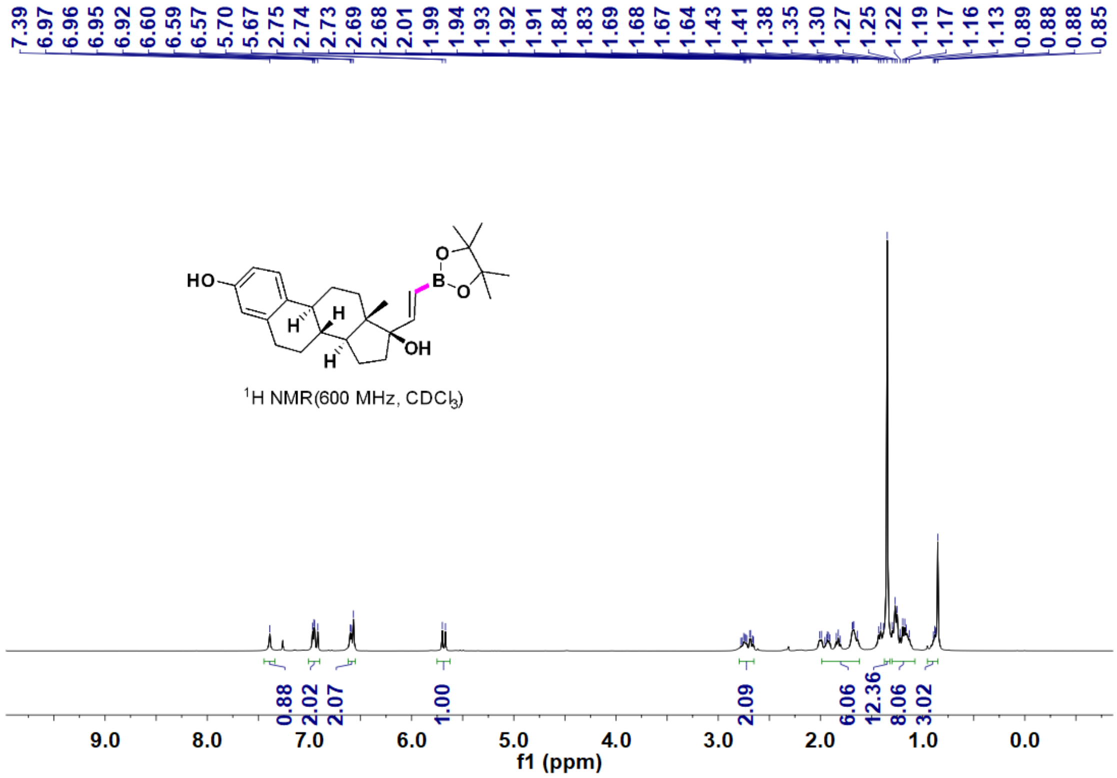

Supplementary Figure 173. Characterization of compound 4aa. ^1H NMR spectrum of compound **4aa** in $CDCl_3$.

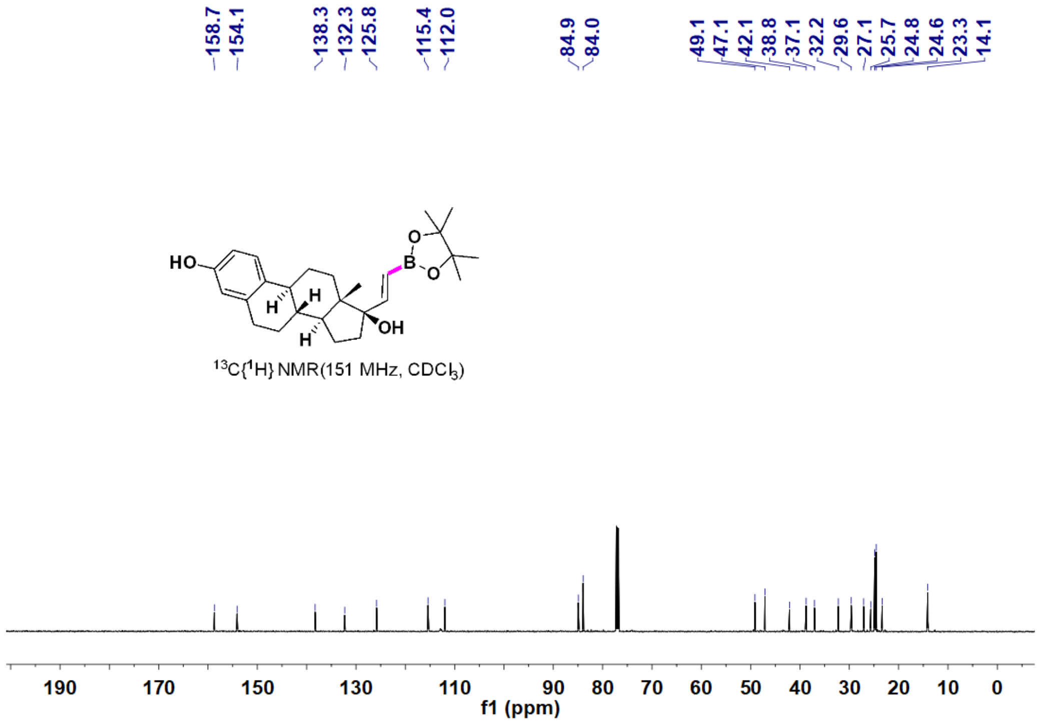

Supplementary Figure 174. Characterization of compound 4aa. ^{13}C{^{1}H} NMR spectrum of compound **4aa** in $CDCl_3$.

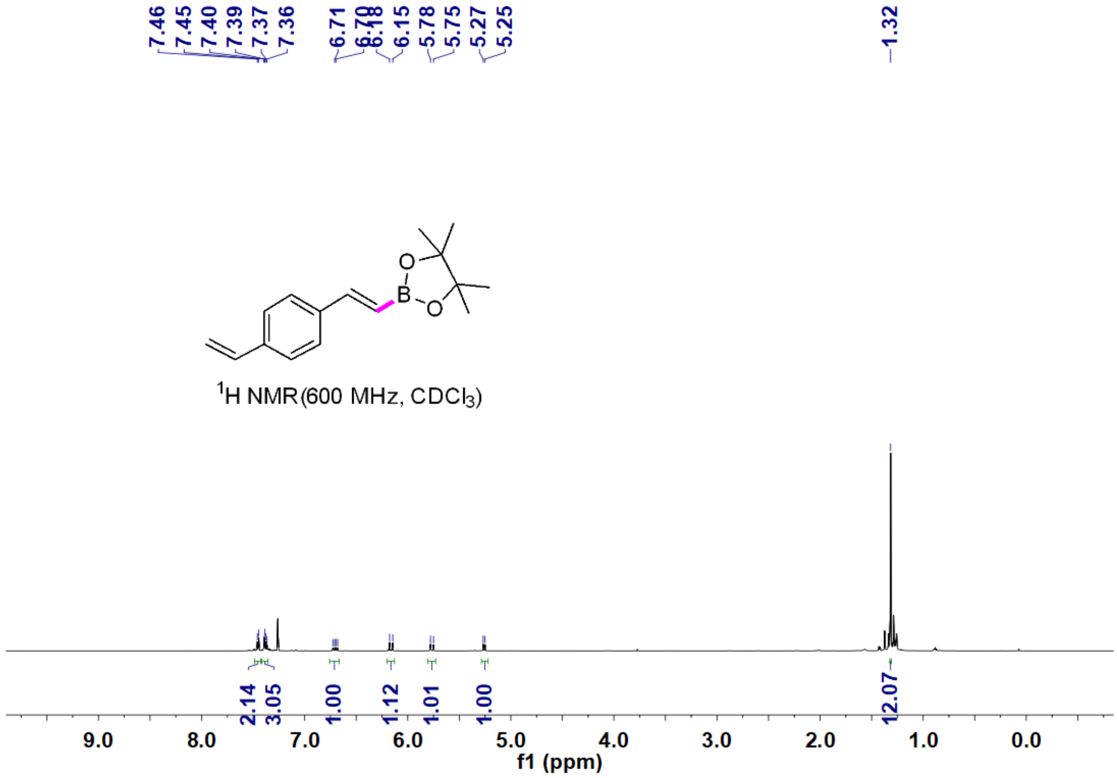

Supplementary Figure 175. Characterization of compound 4ab. ^{1}H NMR spectrum of compound **4ab** in $CDCl_3$.

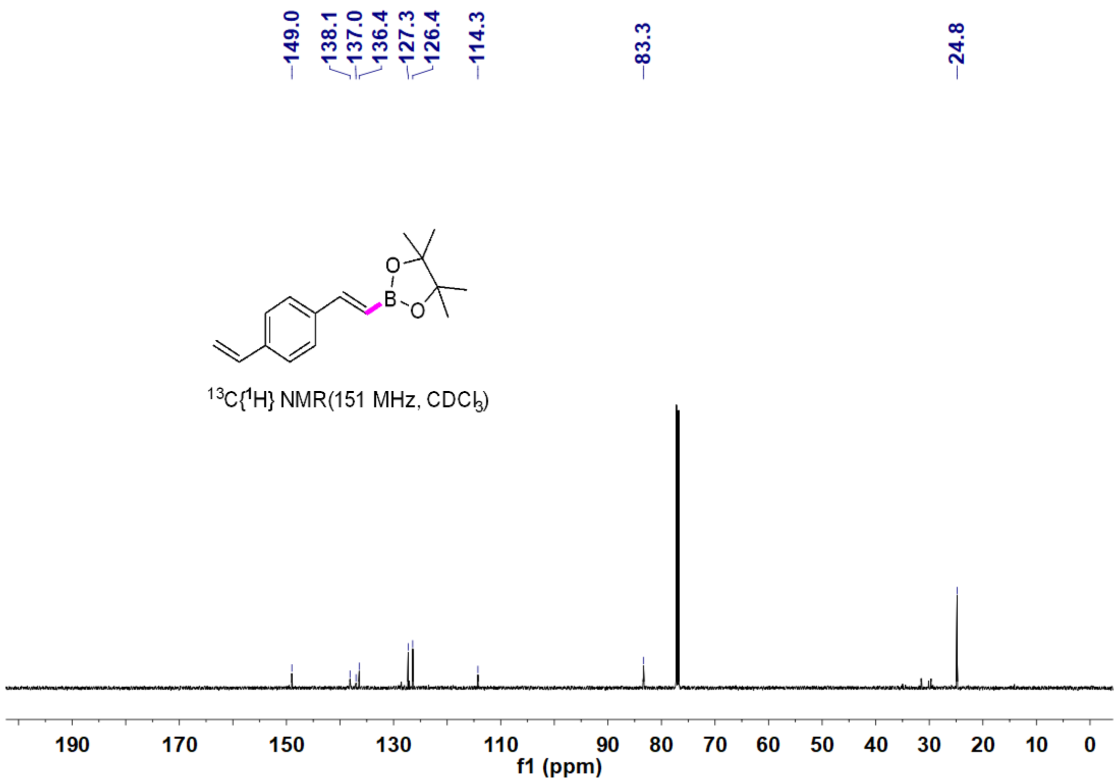

Supplementary Figure 176. Characterization of compound 4ab. $^{13}C\{^{1}H\}$ NMR spectrum of compound **4ab** in $CDCl_3$.

Coordinates of the Structures

14

yne, E = -307.81480106, G = -307.734806

6	6.363200	-3.064867	2.069886
6	5.596234	-3.505268	3.144940
6	4.192504	-3.426475	3.090372
6	3.577006	-2.899275	1.940289
6	4.353218	-2.461555	0.870742
6	5.745722	-2.542728	0.931686
1	7.453097	-3.130023	2.121374
1	6.077686	-3.914202	4.036280
1	2.487293	-2.836600	1.894088
1	3.866313	-2.053362	-0.018674
1	6.351532	-2.198050	0.089595
6	3.399600	-3.878135	4.193187
6	2.727077	-4.262296	5.129564
1	2.129987	-4.602795	5.960671

3

H_2O, E = -76.283631047, G = -76.280445

8	2.310074	2.755575	5.923822

1	2.325665	3.285407	5.117180
1	2.785837	1.958545	5.659129

42

B_2pin_2, E = -821.03673632, G = -820.720244

6	-3.001857	0.366375	-0.737938
6	-2.582417	0.068052	0.742536
6	0.352439	5.320638	0.668329
6	1.010730	4.848822	-0.673590
5	-1.552451	1.954670	0.000927
5	-0.558890	3.347965	-0.000730
8	-2.030746	1.360632	-1.135101
8	-1.957201	1.309954	1.137848
8	-0.343338	4.129905	1.101164
8	0.109161	3.804304	-1.104241
6	-2.908278	-0.819381	-1.677791
1	-3.572110	-1.630679	-1.342867
1	-3.222310	-0.517571	-2.688480
1	-1.883129	-1.207583	-1.742148
6	-4.372495	1.022423	-0.848655
1	-4.508533	1.392539	-1.875842
1	-5.180710	0.310044	-0.628871
1	-4.462392	1.878632	-0.163255
6	-1.517020	-1.015412	0.853737
1	-1.122662	-1.023285	1.880919
1	-1.929015	-2.011103	0.634856
1	-0.677990	-0.823570	0.168067
6	-3.733403	-0.227595	1.683840
1	-4.284838	-1.119926	1.351128
1	-3.344801	-0.424378	2.694496
1	-4.433852	0.615678	1.747259
6	1.089524	5.910954	-1.752368
1	1.697711	6.762447	-1.411617
1	1.564468	5.491460	-2.652166
1	0.094311	6.279788	-2.033922
6	2.373369	4.197367	-0.472724
1	2.675048	3.706248	-1.409976
1	3.141145	4.939091	-0.209361
1	2.338476	3.431958	0.317159
6	-0.705348	6.398659	0.467466
1	-1.266181	6.526229	1.405486
1	-0.252519	7.364888	0.202140
1	-1.419448	6.116646	-0.320845
6	1.333073	5.740202	1.745586
1	1.942175	6.590341	1.403086

1 0.783592 6.055605 2.645457
1 2.003757 4.917831 2.027767
36
pdt, E = -719.01388375, G = -718.751496
5 2.344927 -5.731097 5.844576
8 1.008355 -5.760143 5.547592
8 2.651421 -6.411914 6.990829
6 0.326356 -6.304966 6.696620
6 1.465393 -7.129085 7.395490
6 -0.856528 -7.128296 6.227291
6 -0.148233 -5.122411 7.531831
6 1.596217 -8.544008 6.846154
6 1.393314 -7.155374 8.909231
1 -1.337020 -7.633927 7.078386
1 -1.602701 -6.470706 5.756090
1 -0.557123 -7.885719 5.490745
1 -0.779285 -4.474964 6.904698
1 -0.742213 -5.449826 8.397206
1 0.700377 -4.522938 7.894860
1 2.532423 -8.985603 7.219221
1 0.761138 -9.182106 7.169172
1 1.633346 -8.544118 5.746272
1 0.448865 -7.611449 9.242257
1 2.222640 -7.757606 9.310186
1 1.467353 -6.146733 9.336981
6 6.126910 -2.803670 2.232703
6 5.333691 -3.518517 3.124070
6 3.934002 -3.544692 2.979637
6 3.362521 -2.831303 1.911623
6 4.157035 -2.114723 1.018389
6 5.542917 -2.098213 1.175995
1 7.212650 -2.795171 2.361048
1 5.806931 -4.065385 3.943401
1 2.275806 -2.843052 1.785876
1 3.691322 -1.566812 0.194924
1 6.169793 -1.537669 0.477402
6 3.051879 -4.280411 3.892013
6 3.401176 -5.002428 4.976797
1 4.459241 -5.077648 5.256405
1 1.985479 -4.218280 3.639121
55
1A, E = -9885.6424861, G = -9885.337297
29 3.779606 3.687606 9.638277
16 2.205070 0.670088 10.276902

7	3.982184	2.520573	11.315770
7	3.698692	0.730527	12.567905
6	3.361073	1.348866	11.405577
6	4.734461	2.659010	12.452585
1	5.348979	3.539933	12.630411
6	4.569351	1.553874	13.244511
1	4.985312	1.276729	14.210655
6	3.208054	-0.550792	13.018331
1	2.114204	-0.525413	13.132091
1	3.665138	-0.784191	13.987626
1	3.469993	-1.339032	12.297882
29	0.843163	2.512311	10.259378
16	2.435399	5.559803	9.644097
7	1.103819	4.080638	11.567069
7	1.751023	6.074554	12.246322
6	1.707675	5.206255	11.202107
6	0.750110	4.230419	12.881758
1	0.232988	3.443644	13.429032
6	1.147030	5.465680	13.322025
1	1.054376	5.965308	14.283907
6	2.363585	7.382684	12.237039
1	1.970495	7.983128	11.404731
1	3.455391	7.298820	12.127020
1	2.135682	7.888547	13.183149
29	1.360468	4.030823	8.312803
16	5.001345	2.562462	7.994880
7	6.030862	0.227380	9.011035
7	6.935291	2.065270	9.862711
6	6.025619	1.549371	8.981002
6	6.969595	-0.121171	9.944034
1	7.187333	-1.163061	10.182813
6	7.542719	1.003493	10.486492
1	8.309955	1.146835	11.244821
6	7.203850	3.461741	10.096451
1	6.262690	3.999957	10.285724
1	7.858522	3.559945	10.971160
1	7.694501	3.920323	9.224377
29	2.635882	1.822873	8.339699
16	-0.866804	3.297899	8.893561
7	-1.047824	0.761835	7.808600
7	-1.176849	2.407071	6.328473
6	-1.041301	2.075496	7.652268
6	-1.191012	0.231145	6.554868
1	-1.225898	-0.845832	6.384907

6	-1.274996	1.231731	5.618702
1	-1.392759	1.223322	4.536873
6	-1.210188	3.742344	5.782531
1	-0.333356	4.308703	6.131178
1	-1.189873	3.681381	4.687240
1	-2.118893	4.276491	6.097715
19	3.803802	4.175801	5.709448
8	1.930088	2.866883	6.741220
1	1.144966	2.425269	6.396535

97

Int1, E = -10706.738221, G = 10706.089262

29	4.076256	3.107105	9.334542
16	1.712045	0.636517	9.317447
7	3.977546	1.516472	10.633454
7	3.310871	-0.495548	11.236780
6	3.072710	0.564625	10.421790
6	4.815095	1.054493	11.614560
1	5.654962	1.644441	11.977482
6	4.415159	-0.196880	12.002203
1	4.807945	-0.895747	12.737380
6	2.548070	-1.721423	11.274972
1	1.490372	-1.509288	11.487541
1	2.951241	-2.366000	12.065449
1	2.616850	-2.248135	10.311745
29	1.128948	2.797326	9.860262
16	3.439495	5.293649	9.701603
7	1.752751	3.951855	11.423284
7	2.815673	5.643313	12.352697
6	2.609785	4.944224	11.207060
6	1.398998	4.015454	12.745424
1	0.697873	3.309009	13.187309
6	2.053379	5.061984	13.340084
1	2.047298	5.445199	14.358161
6	3.701835	6.773216	12.509550
1	3.586006	7.176893	13.522708
1	3.455143	7.557556	11.780113
1	4.748637	6.468606	12.361093
29	2.000828	4.424368	8.137015
16	5.010301	2.139280	7.408826
7	5.546968	-0.526995	7.785862
7	6.853869	0.835397	8.950626
6	5.818767	0.739241	8.061554
6	6.432976	-1.268392	8.520732
1	6.437609	-2.358795	8.490723

6	7.254204	-0.443869	9.250473
1	8.067051	-0.641923	9.946361
6	7.411149	2.053949	9.481052
1	6.605346	2.702657	9.857904
1	8.093736	1.809095	10.304155
1	7.963821	2.606456	8.705668
29	2.555400	1.967475	7.629267
16	-0.359910	4.158011	8.676331
7	-0.514802	3.136445	6.123744
7	-1.565417	1.922325	7.643939
6	-0.826638	3.047551	7.409947
6	-1.060901	2.037960	5.515137
1	-0.939936	1.858035	4.447078
6	-1.719203	1.269889	6.441976
1	-2.275442	0.337806	6.364327
6	-2.041871	1.473043	8.928370
1	-2.750198	2.195755	9.359391
1	-2.543760	0.505282	8.805249
1	-1.194238	1.359132	9.622057
5	1.758826	5.207175	3.815583
8	2.516210	6.158437	4.465665
8	0.508887	5.658488	3.523544
6	1.632691	7.234869	4.867232
6	0.430181	7.059521	3.870837
6	2.378123	8.548623	4.744724
6	1.243132	6.980815	6.315911
6	0.603999	7.842753	2.577083
6	-0.934216	7.326954	4.476424
1	1.704683	9.393813	4.951389
1	3.195772	8.579576	5.481251
1	2.812434	8.680507	3.744920
1	2.148757	6.940809	6.941359
1	0.603491	7.785534	6.705911
1	0.715558	6.023816	6.436005
1	-0.166474	7.522183	1.860066
1	0.492107	8.923891	2.742828
1	1.589529	7.655758	2.125071
1	-0.999825	8.360493	4.847987
1	-1.711847	7.189103	3.710120
1	-1.146417	6.637426	5.304381
19	4.455213	4.680036	5.730997
5	2.393129	3.668210	3.422286
8	1.678795	2.571514	3.041134
8	3.751523	3.430666	3.423134

6	2.582713	1.440433	3.006053
6	3.976757	2.133060	2.817760
6	2.172961	0.523701	1.871075
6	2.456041	0.726037	4.344532
6	4.326035	2.391918	1.358627
6	5.126735	1.446816	3.527305
1	2.896813	-0.297592	1.759538
1	1.188826	0.082645	2.090254
1	2.100687	1.061693	0.916379
1	1.406662	0.435113	4.500888
1	3.074544	-0.182460	4.375681
1	2.749658	1.389190	5.171775
1	5.201785	3.056693	1.313339
1	4.574742	1.458974	0.832758
1	3.496099	2.883783	0.829367
1	5.256850	0.424140	3.142216
1	6.061168	1.998663	3.340914
1	4.967333	1.396418	4.613363
8	2.239200	3.520836	6.362465
1	1.304668	3.458395	6.074037

97

TS1-2, E = -10706.735961, G = 10706.084819

29	4.034307	3.066128	9.212935
16	1.712463	0.597079	9.166117
7	3.954197	1.478171	10.517143
7	3.298572	-0.545538	11.093009
6	3.061040	0.519433	10.285346
6	4.783037	1.015315	11.505461
1	5.612017	1.609626	11.885569
6	4.389797	-0.243224	11.876041
1	4.779302	-0.945011	12.610149
6	2.548077	-1.779627	11.108202
1	1.482618	-1.578119	11.289741
1	2.934278	-2.420363	11.910160
1	2.650944	-2.304999	10.147214
29	1.164944	2.757196	9.730618
16	3.398054	5.272117	9.436624
7	1.726294	4.017499	11.232336
7	2.646158	5.862141	12.011705
6	2.534393	5.032087	10.944093
6	1.306544	4.201627	12.523400
1	0.625401	3.505935	13.011109
6	1.871794	5.344742	13.024851
1	1.796011	5.834311	13.993313

6	3.455373	7.057338	12.075193
1	3.263683	7.565936	13.027778
1	3.200034	7.734812	11.248177
1	4.524395	6.805677	12.009807
29	1.910870	4.352068	7.903328
16	5.023478	2.087566	7.302511
7	5.496131	-0.588596	7.686704
7	6.816042	0.745402	8.869737
6	5.789101	0.670764	7.970159
6	6.360100	-1.348882	8.428445
1	6.343859	-2.439024	8.395351
6	7.189380	-0.541989	9.168983
1	7.990933	-0.757497	9.872697
6	7.390798	1.952691	9.407635
1	6.590391	2.626880	9.748321
1	8.036094	1.697177	10.257179
1	7.986760	2.480492	8.647116
29	2.597079	1.909415	7.478191
16	-0.392333	4.031026	8.520480
7	-0.516916	2.676006	6.127659
7	-1.599318	1.685601	7.780429
6	-0.853331	2.766451	7.405576
6	-1.052329	1.503351	5.666368
1	-0.917331	1.180241	4.634453
6	-1.730671	0.872022	6.678028
1	-2.289789	-0.060619	6.718998
6	-2.076607	1.408270	9.112167
1	-2.739129	2.211484	9.465517
1	-2.630477	0.461367	9.104126
1	-1.224082	1.326740	9.805134
5	1.796640	5.191675	4.187715
8	2.590037	6.187684	4.739739
8	0.558775	5.672171	3.864312
6	1.745549	7.322164	5.039675
6	0.554260	7.104279	4.045261
6	2.538011	8.595779	4.824355
6	1.324470	7.198261	6.496623
6	0.802333	7.723160	2.675836
6	-0.801060	7.524694	4.579702
1	1.890716	9.477855	4.942064
1	3.341683	8.663692	5.573884
1	2.996743	8.626262	3.827080
1	2.218222	7.142623	7.136767
1	0.729124	8.064198	6.820637

1	0.733371	6.285629	6.666681
1	0.035040	7.359526	1.976107
1	0.744776	8.820730	2.710804
1	1.788134	7.434532	2.281119
1	-0.807381	8.598064	4.822280
1	-1.572551	7.342809	3.816287
1	-1.073158	6.957054	5.479649
19	4.560809	4.765701	5.877266
5	2.399287	3.607868	3.968883
8	1.689345	2.535814	3.463622
8	3.777783	3.402396	3.791969
6	2.608627	1.459023	3.220727
6	3.958866	2.220941	2.992034
6	2.117187	0.666598	2.023380
6	2.649102	0.566867	4.455603
6	4.138064	2.687300	1.551510
6	5.194249	1.476788	3.461931
1	2.852841	-0.098547	1.732162
1	1.177890	0.153374	2.281261
1	1.922081	1.315778	1.159078
1	1.624657	0.266610	4.721113
1	3.242640	-0.342063	4.277958
1	3.083376	1.105158	5.309841
1	4.984678	3.389283	1.506903
1	4.351811	1.847927	0.873693
1	3.241012	3.212069	1.189679
1	5.292601	0.516905	2.932134
1	6.094116	2.074348	3.246697
1	5.161219	1.287598	4.543272
8	2.149628	3.383748	6.117068
1	1.194656	3.194002	5.946377

97

Int2, E = -10706.747339, G = 10706.100031

29	4.124124	2.756982	9.176832
16	2.013169	0.063821	9.409911
7	4.181320	1.289442	10.607156
7	3.649264	-0.654538	11.495983
6	3.351468	0.252885	10.531442
6	5.032485	1.040281	11.651045
1	5.822325	1.736922	11.927037
6	4.715751	-0.166512	12.216359
1	5.144938	-0.719270	13.048974
6	2.969102	-1.909070	11.719218
1	1.896568	-1.738562	11.891231

1	3.401743	-2.393475	12.602813
1	3.087589	-2.573154	10.850578
29	1.222356	2.167593	9.813898
16	3.295539	4.879759	9.425694
7	1.778660	3.508342	11.275833
7	2.634394	5.391131	12.038234
6	2.517798	4.567279	10.966074
6	1.409009	3.656017	12.586273
1	0.789182	2.918904	13.094538
6	1.934809	4.822203	13.077633
1	1.877178	5.296519	14.054915
6	3.375013	6.631040	12.077712
1	3.232314	7.098587	13.059393
1	3.014966	7.316539	11.297244
1	4.447782	6.445720	11.919658
29	1.833390	3.768413	8.000248
16	5.120863	1.708760	7.285211
7	6.041156	-0.721442	8.185621
7	7.128218	1.022372	9.019292
6	6.115106	0.599294	8.203352
6	7.032355	-1.163543	9.020349
1	7.203945	-2.225419	9.201387
6	7.720863	-0.098620	9.547597
1	8.557775	-0.032250	10.239984
6	7.481438	2.390317	9.303979
1	6.602448	2.943402	9.673024
1	8.263894	2.405759	10.072916
1	7.854088	2.897693	8.401235
29	2.801439	1.322056	7.626075
16	-0.421324	3.543163	8.837691
7	-0.689732	1.976251	6.581394
7	-2.578667	2.359905	7.648132
6	-1.234090	2.581344	7.628176
6	-1.703967	1.352293	5.907630
1	-1.522419	0.783784	4.995972
6	-2.889367	1.578043	6.557697
1	-3.910385	1.263210	6.352464
6	-3.504486	2.853756	8.639218
1	-3.513236	3.953641	8.648505
1	-4.510764	2.489368	8.398921
1	-3.222157	2.498147	9.641099
5	1.635101	4.723373	4.835321
8	2.516182	5.748599	5.196004
8	0.392285	5.247459	4.574940

6	1.752663	6.952469	5.426572
6	0.472429	6.685234	4.567469
6	2.575569	8.151341	4.997815
6	1.459125	7.014680	6.919042
6	0.632472	7.115192	3.114204
6	-0.810075	7.249734	5.147889
1	1.980590	9.074884	5.064967
1	3.446601	8.262756	5.662453
1	2.942794	8.044660	3.968270
1	2.405059	7.020054	7.480252
1	0.897875	7.921710	7.186849
1	0.885842	6.132503	7.247918
1	-0.206945	6.710078	2.529305
1	0.629591	8.210028	3.009252
1	1.566681	6.722742	2.684560
1	-0.746003	8.343735	5.250390
1	-1.652347	7.018780	4.478070
1	-1.031218	6.814862	6.132048
19	4.822908	4.641885	6.070011
5	2.144379	3.061742	4.861391
8	1.527043	2.172481	3.891553
8	3.600429	2.913517	4.641836
6	2.512875	1.282218	3.408187
6	3.802687	2.165836	3.456618
6	2.117035	0.813840	2.016602
6	2.625379	0.071977	4.340216
6	3.870617	3.139017	2.277596
6	5.102274	1.384170	3.555774
1	2.919008	0.221538	1.548259
1	1.219666	0.177612	2.079339
1	1.878309	1.663849	1.362240
1	1.619102	-0.344484	4.503412
1	3.263729	-0.720697	3.919660
1	3.035843	0.367647	5.317300
1	4.661747	3.881415	2.470654
1	4.105977	2.633610	1.328290
1	2.917657	3.677572	2.162171
1	5.212040	0.691938	2.705899
1	5.961857	2.074369	3.538785
1	5.150405	0.818229	4.494975
8	1.840688	2.568922	6.296355
1	0.863070	2.285531	6.306962

97

TS2-3, E = -10706.726267, G = 10706.078402

29	4.032954	2.608881	9.279786
16	2.177525	-0.201067	9.868753
7	4.201151	1.360346	10.917780
7	3.786615	-0.468258	12.074992
6	3.455322	0.261216	10.978747
6	5.030136	1.336892	12.008391
1	5.753567	2.128591	12.196606
6	4.785735	0.205312	12.740704
1	5.225576	-0.182452	13.656833
6	3.190823	-1.724037	12.467304
1	2.113225	-1.599831	12.649419
1	3.672813	-2.073011	13.388482
1	3.331913	-2.479006	11.680317
29	1.293679	1.921449	9.749771
16	3.095906	4.673223	9.312103
7	1.633104	3.344944	11.213856
7	2.357836	5.314696	11.885415
6	2.325967	4.421759	10.862099
6	1.211238	3.547432	12.500568
1	0.617241	2.806978	13.034632
6	1.653058	4.769786	12.935730
1	1.534132	5.297489	13.879636
6	3.021766	6.596877	11.867566
1	2.908140	7.070354	12.850307
1	2.580802	7.250996	11.100942
1	4.092471	6.470688	11.649833
29	1.801955	3.430376	7.610701
16	5.154021	1.388759	7.572584
7	6.249683	-0.813323	8.804414
7	7.168063	1.113494	9.407747
6	6.214603	0.498488	8.643421
6	7.251439	-1.052878	9.706013
1	7.505819	-2.062216	10.032011
6	7.836102	0.128338	10.092330
1	8.646770	0.358454	10.780823
6	7.402763	2.531973	9.514525
1	6.493569	3.051308	9.858627
1	8.207943	2.706289	10.238881
1	7.698978	2.955272	8.542936
29	2.887876	0.850931	7.940313
16	-0.389469	2.984975	8.631041
7	-0.616475	1.059858	6.660820
7	-2.534570	1.795795	7.441958
6	-1.178623	1.894914	7.527965

6	-1.627082	0.415813	5.998684
1	-1.428568	-0.323007	5.223120
6	-2.830898	0.861142	6.474351
1	-3.857595	0.604779	6.222180
6	-3.488261	2.545229	8.224368
1	-3.420835	3.619403	7.996433
1	-4.498700	2.190350	7.987368
1	-3.297637	2.403133	9.298022
5	1.762777	4.698877	5.997385
8	2.803755	5.510647	5.503964
8	0.565358	5.356414	5.763451
6	2.299932	6.833318	5.235995
6	0.780683	6.552861	4.997833
6	3.037939	7.424786	4.049869
6	2.565717	7.665909	6.486054
6	0.451477	6.229094	3.546153
6	-0.150288	7.636823	5.510591
1	2.624968	8.411949	3.791114
1	4.101544	7.559929	4.301666
1	2.977989	6.780392	3.163241
1	3.647256	7.666230	6.693606
1	2.243227	8.709803	6.359503
1	2.056373	7.237913	7.362074
1	-0.574853	5.835514	3.493983
1	0.514598	7.118440	2.901817
1	1.126630	5.457932	3.150962
1	0.052378	8.597574	5.012591
1	-1.194051	7.358323	5.298995
1	-0.051182	7.774642	6.595714
19	4.910422	4.141960	6.185828
5	2.175622	2.165175	5.220665
8	1.297013	2.349614	4.149613
8	3.506988	2.261452	4.780736
6	2.023644	1.950890	2.981735
6	3.506284	2.342751	3.343876
6	1.455977	2.661670	1.768840
6	1.838332	0.441760	2.828699
6	3.857132	3.762911	2.921007
6	4.556383	1.379856	2.816256
1	2.035233	2.416938	0.865677
1	0.416906	2.337153	1.605817
1	1.453542	3.750972	1.900499
1	0.760333	0.226283	2.775063
1	2.314485	0.055877	1.915542

1	2.246264	-0.101353	3.694348
1	4.867848	4.021669	3.278058
1	3.878382	3.859900	1.826240
1	3.146001	4.486588	3.335578
1	4.522398	1.326589	1.717300
1	5.558968	1.730139	3.107533
1	4.420898	0.369840	3.224684
8	1.869150	1.286555	6.249198
1	0.829025	1.157235	6.384160

88

Int3, E = -10528.286356, G = 10527.720693

29	3.526897	3.273342	9.795311
16	1.655504	0.598876	9.856626
7	3.861815	1.697600	11.061123
7	3.587912	-0.444641	11.501987
6	3.093021	0.634802	10.842464
6	4.872033	1.291185	11.893820
1	5.648867	1.974001	12.233338
6	4.719245	-0.040572	12.176454
1	5.299451	-0.729919	12.785845
6	3.037081	-1.778984	11.469641
1	1.967360	-1.754298	11.721294
1	3.565332	-2.400526	12.202874
1	3.153225	-2.223236	10.469284
29	0.802448	2.573817	10.543912
16	2.728923	5.400158	10.415221
7	1.349418	3.769426	12.136915
7	2.146012	5.589901	13.091710
6	2.036266	4.888664	11.933736
6	1.007131	3.752316	13.462731
1	0.432415	2.935203	13.896313
6	1.496226	4.877560	14.073484
1	1.444995	5.234303	15.099914
6	2.840131	6.845268	13.258366
1	3.912472	6.724429	13.044481
1	2.715720	7.184613	14.293667
1	2.427899	7.605091	12.579025
29	1.295000	4.320012	8.780445
16	4.733994	2.693150	7.831646
7	6.261919	0.441589	8.217033
7	6.899337	2.166891	9.451268
6	6.003276	1.704664	8.521454
6	7.347929	0.080643	8.967178
1	7.776848	-0.921041	8.917649

6	7.762688	1.136856	9.738387
1	8.569803	1.250710	10.459442
6	6.952680	3.486313	10.025179
1	5.950756	3.794049	10.363240
1	7.626430	3.475877	10.891097
1	7.336418	4.221573	9.299015
29	2.085015	2.194258	7.935269
16	-0.882756	3.985952	9.724086
7	-1.917191	1.909250	8.215416
7	-1.916140	3.878472	7.198243
6	-1.596038	3.188761	8.334822
6	-2.453871	1.771919	6.963455
1	-2.802317	0.808938	6.587556
6	-2.460742	2.981429	6.312504
1	-2.791420	3.283602	5.320676
6	-1.693043	5.282503	6.966388
1	-0.645790	5.529263	7.191001
1	-1.897865	5.509701	5.913227
1	-2.347815	5.896057	7.603793
5	1.814246	4.104714	6.761759
8	2.853871	4.953574	6.342549
8	0.933851	3.958040	5.698903
6	2.532096	5.513347	5.048656
6	1.536229	4.452832	4.491601
6	3.807275	5.673714	4.242807
6	1.879726	6.868157	5.296457
6	2.239366	3.275952	3.822761
6	0.453642	5.004784	3.585726
1	3.573170	5.958897	3.205867
1	4.434163	6.475488	4.665798
1	4.395442	4.746488	4.226692
1	2.565517	7.507770	5.874273
1	1.654911	7.386646	4.353021
1	0.948308	6.759481	5.871642
1	1.505066	2.478179	3.636289
1	2.684626	3.562941	2.859079
1	3.028123	2.864506	4.470471
1	0.894738	5.460573	2.686190
1	-0.209161	4.187040	3.264118
1	-0.160360	5.757993	4.096484
19	4.758813	5.813556	7.907580
6	2.090276	-3.522987	7.229922
6	1.447652	-2.328895	6.913680
6	2.166225	-1.119648	6.919918

6	3.532217	-1.127463	7.248380
6	4.164207	-2.326703	7.560946
6	3.448846	-3.526403	7.554452
1	1.524411	-4.458240	7.226274
1	0.383476	-2.322149	6.666541
1	4.098213	-0.193372	7.286456
1	5.225449	-2.314980	7.821911
1	3.949126	-4.465502	7.805603
6	1.499410	0.107167	6.618144
6	0.869691	1.123060	6.349467
1	0.276770	1.938527	5.958450

88

Int4, E = -10528.286356, G = 10527.720693

29	3.526897	3.273342	9.795311
16	1.655504	0.598876	9.856626
7	3.861815	1.697600	11.061123
7	3.587912	-0.444641	11.501987
6	3.093021	0.634802	10.842464
6	4.872033	1.291185	11.893820
1	5.648867	1.974001	12.233338
6	4.719245	-0.040572	12.176454
1	5.299451	-0.729919	12.785845
6	3.037081	-1.778984	11.469641
1	1.967360	-1.754298	11.721294
1	3.565332	-2.400526	12.202874
1	3.153225	-2.223236	10.469284
29	0.802448	2.573817	10.543912
16	2.728923	5.400158	10.415221
7	1.349418	3.769426	12.136915
7	2.146012	5.589901	13.091710
6	2.036266	4.888664	11.933736
6	1.007131	3.752316	13.462731
1	0.432415	2.935203	13.896313
6	1.496226	4.877560	14.073484
1	1.444995	5.234303	15.099914
6	2.840131	6.845268	13.258366
1	3.912472	6.724429	13.044481
1	2.715720	7.184613	14.293667
1	2.427899	7.605091	12.579025
29	1.295000	4.320012	8.780445
16	4.733994	2.693150	7.831646
7	6.261919	0.441589	8.217033
7	6.899337	2.166891	9.451268
6	6.003276	1.704664	8.521454

6	7.347929	0.080643	8.967178
1	7.776848	-0.921041	8.917649
6	7.762688	1.136856	9.738387
1	8.569803	1.250710	10.459442
6	6.952680	3.486313	10.025179
1	5.950756	3.794049	10.363240
1	7.626430	3.475877	10.891097
1	7.336418	4.221573	9.299015
29	2.085015	2.194258	7.935269
16	-0.882756	3.985952	9.724086
7	-1.917191	1.909250	8.215416
7	-1.916140	3.878472	7.198243
6	-1.596038	3.188761	8.334822
6	-2.453871	1.771919	6.963455
1	-2.802317	0.808938	6.587556
6	-2.460742	2.981429	6.312504
1	-2.791420	3.283602	5.320676
6	-1.693043	5.282503	6.966388
1	-0.645790	5.529263	7.191001
1	-1.897865	5.509701	5.913227
1	-2.347815	5.896057	7.603793
5	1.814246	4.104714	6.761759
8	2.853871	4.953574	6.342549
8	0.933851	3.958040	5.698903
6	2.532096	5.513347	5.048656
6	1.536229	4.452832	4.491601
6	3.807275	5.673714	4.242807
6	1.879726	6.868157	5.296457
6	2.239366	3.275952	3.822761
6	0.453642	5.004784	3.585726
1	3.573170	5.958897	3.205867
1	4.434163	6.475488	4.665798
1	4.395442	4.746488	4.226692
1	2.565517	7.507770	5.874273
1	1.654911	7.386646	4.353021
1	0.948308	6.759481	5.871642
1	1.505066	2.478179	3.636289
1	2.684626	3.562941	2.859079
1	3.028123	2.864506	4.470471
1	0.894738	5.460573	2.686190
1	-0.209161	4.187040	3.264118
1	-0.160360	5.757993	4.096484
19	4.758813	5.813556	7.907580
6	2.090276	-3.522987	7.229922

6	1.447652	-2.328895	6.913680
6	2.166225	-1.119648	6.919918
6	3.532217	-1.127463	7.248380
6	4.164207	-2.326703	7.560946
6	3.448846	-3.526403	7.554452
1	1.524411	-4.458240	7.226274
1	0.383476	-2.322149	6.666541
1	4.098213	-0.193372	7.286456
1	5.225449	-2.314980	7.821911
1	3.949126	-4.465502	7.805603
6	1.499410	0.107167	6.618144
6	0.869691	1.123060	6.349467
1	0.276770	1.938527	5.958450

88

TS4-5, E = -10528.260662, G = 10527.691720

29	3.685828	3.437704	9.689259
16	1.892901	0.674387	10.032358
7	3.813734	2.170264	11.314822
7	3.511961	0.166782	12.182571
6	3.137062	1.032149	11.204501
6	4.631405	2.040007	12.406910
1	5.300264	2.839035	12.722677
6	4.457516	0.798672	12.959793
1	4.915847	0.307564	13.815262
6	3.019466	-1.179765	12.348440
1	1.930505	-1.177056	12.502981
1	3.505633	-1.632001	13.221249
1	3.248105	-1.781291	11.456061
29	0.886790	2.698290	10.206499
16	2.867828	5.598640	9.958510
7	1.331451	4.097501	11.694711
7	2.209947	5.905789	12.600380
6	2.081957	5.169384	11.465025
6	0.967128	4.147344	13.013307
1	0.341847	3.378357	13.464829
6	1.506243	5.265489	13.594214
1	1.454736	5.662947	14.605576
6	2.976099	7.122076	12.739886
1	2.628769	7.880126	12.023402
1	4.043942	6.928271	12.559065
1	2.848482	7.506827	13.758929
29	1.561625	4.267376	8.439224
16	4.910690	2.243970	8.087465
7	5.606594	-0.225700	9.082131

7	6.754614	1.464044	9.945077
6	5.770278	1.086055	9.071893
6	6.511652	-0.710277	9.985866
1	6.597035	-1.776427	10.199857
6	7.237198	0.320936	10.532299
1	8.031470	0.350216	11.275592
6	7.185739	2.811992	10.216209
1	6.307640	3.455594	10.377967
1	7.808167	2.815989	11.119627
1	7.772737	3.218958	9.377551
29	2.452913	1.956764	8.029066
16	-0.749560	3.984637	9.178766
7	-1.194279	1.772426	7.575384
7	-2.296819	3.596911	6.973799
6	-1.419694	3.043861	7.864277
6	-1.934570	1.503032	6.456596
1	-1.933656	0.517767	5.988129
6	-2.630591	2.621171	6.065316
1	-3.324939	2.810150	5.248725
6	-2.808583	4.944182	7.003670
1	-1.985097	5.660863	7.122748
1	-3.332133	5.151024	6.061787
1	-3.511900	5.081195	7.839948
5	2.057226	3.417696	6.433516
8	3.100743	4.121864	5.782941
8	0.840418	3.995923	6.059996
6	2.532028	5.245375	5.062924
6	1.042270	4.809149	4.894245
6	3.288535	5.430736	3.761287
6	2.658719	6.497339	5.928462
6	0.822480	3.926056	3.671288
6	0.034130	5.940147	4.904793
1	2.804036	6.203137	3.144986
1	4.320354	5.764663	3.955525
1	3.337086	4.498185	3.184606
1	3.712505	6.793792	6.054279
1	2.175315	7.359510	5.449486
1	2.205343	6.350099	6.921659
1	-0.171128	3.458871	3.746169
1	0.857386	4.514734	2.743251
1	1.573704	3.126299	3.598046
1	0.249025	6.661978	4.102467
1	-0.973948	5.534533	4.737152
1	0.032642	6.467471	5.867956

19	5.322985	5.002080	7.118357
6	3.278024	-3.027883	7.397172
6	2.664711	-1.784155	7.324225
6	3.164023	-0.783095	6.450123
6	4.306023	-1.093674	5.665163
6	4.916215	-2.337472	5.767103
6	4.412088	-3.317033	6.629443
1	2.872401	-3.783323	8.076719
1	1.802850	-1.554434	7.953897
1	4.712474	-0.332653	4.994509
1	5.801623	-2.549034	5.160286
1	4.896571	-4.293893	6.702385
6	2.596123	0.500181	6.412060
6	1.988634	1.574825	6.065226
1	1.179903	1.727432	5.341667

88

Int5, E = -10528.368702, G = 10527.794785

29	3.635944	3.369321	9.484841
16	1.508139	0.761914	10.940299
7	3.581704	2.529832	11.366240
7	2.848031	1.535263	13.191722
6	2.709904	1.649955	11.843742
6	4.301650	2.992702	12.438820
1	5.092271	3.732317	12.320725
6	3.857376	2.384498	13.582836
1	4.158993	2.474147	14.624040
6	2.041465	0.704811	14.054073
1	0.978067	0.964693	13.945781
1	2.347627	0.868819	15.094427
1	2.171042	-0.358414	13.804278
29	0.225212	2.646162	10.504381
16	2.278275	5.281291	9.645937
7	0.784226	4.186482	11.701580
7	2.100886	5.844122	12.308985
6	1.673527	5.076548	11.270816
6	0.639620	4.388127	13.050495
1	-0.035697	3.781611	13.651968
6	1.451094	5.418439	13.444511
1	1.625102	5.883748	14.412251
6	3.090155	6.892253	12.231273
1	2.762289	7.684760	11.543151
1	4.048160	6.488587	11.871064
1	3.232921	7.319983	13.230950
29	1.013313	3.802234	8.404889

16	5.554508	3.520711	8.226526
7	7.117381	1.865971	6.736409
7	6.273719	0.846311	8.509902
6	6.331991	2.015075	7.806304
6	7.584601	0.578810	6.775144
1	8.255671	0.184194	6.011743
6	7.072981	-0.070470	7.867308
1	7.184967	-1.087663	8.236692
6	5.490651	0.594709	9.691034
1	5.860185	1.180863	10.544707
1	4.441801	0.879698	9.516946
1	5.542085	-0.474306	9.931930
29	1.954893	1.430799	8.785517
16	-1.187991	3.045454	8.744429
7	-1.632390	0.329701	8.738817
7	-1.258070	1.174570	6.724478
6	-1.345741	1.431719	8.064145
6	-1.739502	-0.665091	7.805444
1	-1.963002	-1.696015	8.083179
6	-1.512377	-0.162750	6.547314
1	-1.488930	-0.624314	5.562098
6	-0.974777	2.132492	5.684686
1	-0.239480	2.855991	6.054209
1	-0.557340	1.611057	4.813843
1	-1.886314	2.671108	5.380948
5	2.567853	3.569134	5.779854
8	3.330642	4.401968	4.985217
8	1.529023	4.276909	6.361773
6	2.885417	5.767627	5.199447
6	1.410849	5.547260	5.656943
6	3.040486	6.543017	3.908684
6	3.748325	6.362196	6.303796
6	0.457854	5.313337	4.493266
6	0.855242	6.598647	6.589632
1	2.598624	7.545392	4.011324
1	4.108315	6.667770	3.671350
1	2.558808	6.029005	3.066485
1	4.802934	6.381625	5.988139
1	3.457385	7.399667	6.519458
1	3.676109	5.769224	7.227967
1	-0.511335	4.972793	4.884462
1	0.291151	6.242334	3.930105
1	0.841583	4.547501	3.802424
1	0.834895	7.574782	6.082691

1	-0.176115	6.333808	6.867971
1	1.441217	6.684112	7.512521
19	5.942079	3.658592	5.081598
6	2.269258	-2.553098	7.066600
6	1.963461	-1.201327	7.197830
6	2.865570	-0.201072	6.782362
6	4.106432	-0.618972	6.263937
6	4.424361	-1.973325	6.155667
6	3.506247	-2.948355	6.548238
1	1.540397	-3.305119	7.382480
1	1.003793	-0.895772	7.625967
1	4.843606	0.133689	5.972253
1	5.403809	-2.267407	5.767104
1	3.756106	-4.009391	6.462833
6	2.515483	1.233556	6.922942
6	2.801401	2.053487	5.875565
1	3.255009	1.599699	4.971244

91

Int6, E = -10604.650239, G = 10604.060187

29	3.897685	3.225169	9.903403
16	3.163769	0.089107	10.720259
7	4.535801	2.308988	11.606893
7	4.845132	0.512343	12.840881
6	4.223031	1.025094	11.745628
6	5.374316	2.631760	12.642112
1	5.781320	3.634493	12.762093
6	5.580008	1.522802	13.419469
1	6.170677	1.358347	14.318081
6	4.748527	-0.851617	13.304209
1	3.704263	-1.106622	13.537385
1	5.356543	-0.963555	14.210016
1	5.117259	-1.546790	12.535753
29	1.563150	1.660240	10.145596
16	2.496737	5.067473	10.141348
7	1.213409	3.127089	11.632369
7	1.189732	5.130883	12.550534
6	1.585329	4.393119	11.479693
6	0.560742	3.049448	12.833574
1	0.148128	2.110925	13.201830
6	0.537267	4.287362	13.420713
1	0.124204	4.644856	14.361509
6	1.433411	6.540902	12.745756
1	1.025681	7.120958	11.905391
1	2.512601	6.740745	12.822144

1	0.942921	6.861110	13.672965
29	1.795727	3.610799	8.498001
16	5.149861	2.560766	8.130929
7	6.789242	0.456504	8.781895
7	7.392255	2.384131	9.695737
6	6.499250	1.743662	8.884405
6	7.903544	0.260090	9.551438
1	8.367719	-0.721204	9.655352
6	8.294841	1.443746	10.128494
1	9.114846	1.701933	10.795660
6	7.400667	3.789383	10.020018
1	6.386949	4.113172	10.295761
1	8.078049	3.958422	10.866008
1	7.737846	4.390310	9.161333
29	3.447758	0.794473	8.515998
16	-0.267437	2.563900	8.926236
7	-0.841949	0.688070	6.978754
7	-0.798720	2.780548	6.250738
6	-0.649741	1.944325	7.335887
6	-1.123127	0.702578	5.640371
1	-1.317170	-0.215615	5.084732
6	-1.108361	1.989927	5.164928
1	-1.286591	2.415767	4.179442
6	-0.773251	4.225178	6.265833
1	0.157143	4.590241	6.726816
1	-0.830433	4.592295	5.233626
1	-1.619993	4.627223	6.841940
5	1.026015	-1.620342	7.873611
8	0.150545	-2.665515	7.679488
8	1.022445	-1.187024	9.171950
6	-0.642755	-2.816235	8.869941
6	0.240984	-2.096587	9.956974
6	-0.871801	-4.296138	9.119775
6	-1.973141	-2.117570	8.621527
6	1.218230	-3.040657	10.648632
6	-0.529849	-1.297760	10.991826
1	-1.405607	-4.453546	10.069330
1	-1.488371	-4.715793	8.310103
1	0.072272	-4.856325	9.150555
1	-2.458604	-2.580680	7.748210
1	-2.650403	-2.217646	9.482446
1	-1.815150	-1.054228	8.392757
1	1.926152	-2.438876	11.236891
1	0.703375	-3.741751	11.321609

1	1.797623	-3.619796	9.913620
1	-1.185334	-1.950347	11.588327
1	0.185016	-0.807405	11.670399
1	-1.138161	-0.512876	10.524041
19	4.509515	3.365069	5.242234
6	5.862246	0.432284	4.506241
6	5.106460	0.158816	5.647246
6	3.696694	0.115154	5.595633
6	3.081093	0.385479	4.354488
6	3.836831	0.690986	3.219449
6	5.233943	0.712658	3.286132
1	6.954210	0.445162	4.573357
1	5.604911	-0.009137	6.607002
1	1.988507	0.380405	4.293624
1	3.330627	0.911377	2.274957
1	5.827090	0.947045	2.398224
6	2.900261	-0.139033	6.816123
6	1.981529	-1.143867	6.749220
1	1.910364	-1.724428	5.807794
8	2.232381	2.763460	6.517704
1	1.397180	2.644833	6.036457
1	2.442179	1.845094	6.851644

91

TS6-7, E = -10604.637526, G = 10604.045184

29	3.755753	3.361881	9.678743
16	2.839013	0.289853	10.687586
7	4.191268	2.548865	11.508684
7	4.290482	0.869312	12.930198
6	3.817811	1.295101	11.729302
6	4.915057	2.946281	12.602132
1	5.341374	3.945155	12.677744
6	4.989132	1.911038	13.496654
1	5.467053	1.817160	14.469317
6	4.086207	-0.440040	13.504491
1	3.014885	-0.626380	13.670508
1	4.612722	-0.491672	14.465032
1	4.480899	-1.218565	12.835691
29	1.243508	1.849296	10.159452
16	2.350934	5.174358	9.623466
7	1.101645	3.525169	11.455799
7	1.196738	5.641394	12.065127
6	1.500529	4.739147	11.095528
6	0.524023	3.650803	12.690964
1	0.106874	2.794047	13.218695

6	0.575702	4.961468	13.087725
1	0.233803	5.469386	13.987002
6	1.492233	7.054680	12.031797
1	1.032827	7.523239	11.149551
1	2.578894	7.222278	11.995629
1	1.087160	7.522852	12.936920
29	1.552457	3.484874	8.255249
16	5.162727	2.458640	8.146539
7	6.487093	0.255419	9.098465
7	7.183805	2.170797	9.972990
6	6.324251	1.568174	9.099363
6	7.483216	0.001892	10.001705
1	7.827395	-1.011310	10.211675
6	7.930471	1.176783	10.556106
1	8.694358	1.396129	11.299433
6	7.311093	3.587181	10.213606
1	6.311901	4.041778	10.260497
1	7.826847	3.745529	11.168664
1	7.881989	4.075263	9.408339
29	3.218590	0.985468	8.499724
16	-0.595066	2.762080	8.988319
7	-1.641779	0.753920	7.414236
7	-1.290801	2.658117	6.332254
6	-1.198539	1.991283	7.535459
6	-2.021789	0.605467	6.109012
1	-2.415334	-0.337741	5.727816
6	-1.821110	1.773747	5.418705
1	-2.003444	2.061044	4.384913
6	-1.094679	4.070623	6.094944
1	-0.259954	4.442677	6.704440
1	-0.865497	4.231605	5.033229
1	-1.996455	4.644720	6.359737
5	1.835567	-1.854030	8.097249
8	0.483110	-1.677746	8.062434
8	2.227397	-2.718284	9.084518
6	-0.109149	-2.351087	9.185556
6	1.052612	-3.310088	9.668167
6	-1.360650	-3.065433	8.704098
6	-0.494701	-1.286768	10.202992
6	0.949265	-4.720290	9.100697
6	1.236230	-3.363108	11.173640
1	-1.814439	-3.653219	9.516064
1	-2.096150	-2.318549	8.369179
1	-1.149567	-3.735287	7.859830

1	-1.159064	-0.563823	9.708790
1	-1.013898	-1.723631	11.068582
1	0.384501	-0.733472	10.561281
1	1.870178	-5.270908	9.345635
1	0.096151	-5.269581	9.524503
1	0.847105	-4.703588	8.005388
1	0.331615	-3.749475	11.666591
1	2.073148	-4.034516	11.419825
1	1.467917	-2.369247	11.578828
19	4.151291	3.501577	5.463017
6	6.097531	0.591246	4.541291
6	5.288419	0.256360	5.628746
6	3.882847	0.299823	5.528900
6	3.326833	0.665147	4.285411
6	4.137031	0.993690	3.195614
6	5.530016	0.972277	3.321144
1	7.185672	0.558496	4.649924
1	5.739948	-0.018940	6.585914
1	2.238169	0.696910	4.178710
1	3.676041	1.270884	2.243086
1	6.166074	1.240276	2.473495
6	3.004853	0.026503	6.688249
6	2.799908	-1.276896	7.018463
1	3.343944	-2.055404	6.441934
8	1.803342	2.555471	6.362618
1	0.937736	2.410907	5.948879
1	2.121764	1.609851	6.635977

91

Int7, E = -10604.696706, G = 10604.102168

29	3.593757	3.514897	9.842760
16	1.781662	0.853471	10.461411
7	3.805139	2.370587	11.532692
7	3.654411	0.353572	12.407397
6	3.138300	1.220720	11.498685
6	4.763986	2.243398	12.502502
1	5.458721	3.048647	12.734834
6	4.683102	0.993282	13.059057
1	5.256070	0.503962	13.843614
6	3.190717	-0.993583	12.647692
1	2.224445	-0.984721	13.174681
1	3.930361	-1.518285	13.264840
1	3.070451	-1.520827	11.691541
29	0.746725	2.857568	10.494956
16	2.650567	5.606819	9.663966

7	1.040101	4.527182	11.632936
7	1.652250	6.609230	12.013218
6	1.726715	5.559990	11.153674
6	0.509301	4.926077	12.831322
1	-0.105731	4.261430	13.436181
6	0.881721	6.219805	13.084850
1	0.674511	6.891876	13.914817
6	2.284395	7.895574	11.835503
1	1.946817	8.364270	10.899858
1	3.378505	7.786887	11.801075
1	2.014464	8.543186	12.678329
29	1.493674	4.057516	8.395839
16	4.828269	2.375007	8.264690
7	6.140501	0.113020	9.096925
7	6.936832	1.991331	9.964170
6	6.002903	1.430506	9.137530
6	7.191272	-0.182376	9.923039
1	7.526226	-1.207451	10.085495
6	7.701638	0.968507	10.470968
1	8.517130	1.153872	11.167218
6	7.105399	3.397822	10.231458
1	6.123714	3.861528	10.408962
1	7.734393	3.523416	11.121472
1	7.579952	3.908455	9.378882
29	2.606260	1.178455	8.185351
16	-0.830785	3.723393	8.993110
7	-1.424402	1.221650	7.974250
7	-1.162080	2.801396	6.441802
6	-1.165244	2.505634	7.782585
6	-1.587093	0.674545	6.729868
1	-1.801346	-0.385707	6.590202
6	-1.431049	1.634780	5.761557
1	-1.487264	1.603949	4.675163
6	-0.942631	4.098850	5.849782
1	-0.032683	4.552467	6.270469
1	-0.819494	3.980756	4.765762
1	-1.789645	4.773449	6.046365
5	2.118545	-1.763663	8.579439
8	0.832051	-1.979110	8.165500
8	2.467611	-2.548654	9.648291
6	0.190677	-2.832441	9.133238
6	1.420325	-3.527578	9.822402
6	-0.744219	-3.778825	8.404526
6	-0.598919	-1.939050	10.080379

6	1.878041	-4.785367	9.093769
6	1.236557	-3.816667	11.299099
1	-1.168727	-4.516899	9.101651
1	-1.577094	-3.208998	7.964986
1	-0.232194	-4.313531	7.593388
1	-1.278482	-1.305001	9.491729
1	-1.197460	-2.532963	10.786431
1	0.068205	-1.272504	10.645137
1	2.849880	-5.099527	9.503208
1	1.165272	-5.612487	9.222852
1	2.007250	-4.597673	8.017087
1	0.407785	-4.525223	11.448083
1	2.151719	-4.273155	11.705930
1	1.023697	-2.905959	11.872545
19	3.879196	3.720669	5.707105
6	5.754404	0.833347	4.619890
6	4.985663	0.277979	5.640834
6	3.582628	0.232194	5.535971
6	2.981699	0.751170	4.373682
6	3.751725	1.308897	3.352209
6	5.144617	1.355452	3.473048
1	6.842682	0.864924	4.720712
1	5.473217	-0.105920	6.540542
1	1.891924	0.721958	4.282169
1	3.264119	1.707726	2.458417
1	5.752646	1.790119	2.675265
6	2.722788	-0.321694	6.587377
6	3.111613	-0.861396	7.795621
1	4.172472	-0.857677	8.080192
8	1.919030	2.710792	6.994418
1	1.106182	2.400514	6.577960
1	1.671829	-0.439247	6.292986

115

B, E = -18668.283339, G = 18667.522546

29	-7.103979	25.368845	12.331213
29	-6.715627	23.518280	10.347112
29	-8.773742	21.496640	11.045243
29	-6.961665	19.673339	11.339166
16	-6.207151	23.399100	12.657389
16	-6.724169	21.303123	9.776530
16	-7.044157	25.134668	8.646541
16	-10.291237	19.637984	11.080573
7	-6.053298	27.490318	9.721044
7	-4.508479	26.054427	9.049913

7	-8.059539	18.072205	10.699551
7	-9.914689	17.183695	9.911950
6	-5.846426	26.302513	9.170587
6	-4.360172	23.388863	12.869467
6	-9.367372	18.252750	10.548615
6	-4.813456	28.021458	9.961871
6	-3.840249	27.147031	9.549284
6	-7.365394	21.069008	8.037433
6	-7.759495	16.852495	10.155003
6	-8.903505	16.284992	9.657508
6	-3.796820	22.347343	11.912721
6	-11.309893	17.023459	9.576042
6	-3.910872	24.849211	8.527523
6	-3.843025	24.779615	12.536142
6	-4.080729	23.012156	14.318139
6	-6.335057	21.720454	7.121067
6	-8.712810	21.770165	7.914852
6	-7.499076	19.585936	7.730759
29	-8.617699	25.229780	10.321220
29	-8.914406	23.760403	13.782419
29	-7.247494	21.468580	13.358369
29	-9.220135	19.827326	13.126951
16	-9.629689	23.598543	11.545770
16	-9.321801	21.611375	14.535126
16	-8.384497	25.529536	15.187792
16	-5.888342	19.509759	13.382787
7	-11.078891	26.091231	15.341634
7	-10.126093	25.730132	17.307776
7	-8.252060	18.178687	13.850898
7	-6.483110	17.169807	14.690251
6	-9.935207	25.792564	15.955474
6	-11.482948	23.529899	11.366125
6	-6.932867	18.247507	13.995228
6	-12.022816	26.215754	16.326733
6	-11.452726	25.999261	17.554157
6	-8.808540	21.378477	16.316767
6	-8.659905	17.022013	14.459503
6	-7.570842	16.380843	14.989478
6	-12.025193	22.597890	12.438203
6	-5.106714	16.900281	15.034870
6	-9.118657	25.487892	18.311075
6	-12.040188	24.935048	11.546722
6	-11.780076	23.008253	9.967239
6	-9.841192	22.144490	17.136295

6	-7.419725	21.970356	16.519292
6	-8.824364	19.900758	16.671883
1	-2.993098	22.981949	14.500327
1	-4.529320	23.740237	15.011749
1	-4.497359	22.017808	14.544194
1	-2.743163	24.808890	12.619149
1	-4.119886	25.059561	11.508639
1	-4.263418	25.533854	13.219958
1	-2.708182	22.243500	12.057930
1	-4.269360	21.366999	12.076346
1	-3.989984	22.632484	10.867660
1	-2.753365	27.195310	9.565843
1	-4.685479	29.001387	10.422113
1	-4.012636	24.796723	7.432930
1	-2.846851	24.831856	8.793275
1	-4.410132	23.971594	8.965173
1	-9.556223	24.923302	19.145032
1	-8.302185	24.906457	17.863910
1	-8.707997	26.435167	18.695190
1	-11.857314	26.007044	18.564254
1	-13.063239	26.451784	16.101409
1	-11.568310	25.637765	10.839451
1	-13.126863	24.941533	11.358549
1	-11.888226	25.276389	12.583123
1	-11.366557	21.996803	9.837223
1	-12.869710	22.964311	9.799533
1	-11.336619	23.664617	9.201573
1	-11.840070	23.008516	13.442138
1	-13.111022	22.454063	12.306777
1	-11.527297	21.618197	12.395618
1	-6.743395	16.460821	10.146660
1	-9.092634	15.338146	9.156302
1	-11.929087	17.032124	10.485066
1	-11.641961	17.837860	8.915818
1	-11.443147	16.065408	9.059273
1	-7.466791	15.449504	15.541905
1	-9.707376	16.725590	14.487558
1	-4.685924	17.736695	15.611298
1	-5.065130	15.988780	15.643277
1	-4.501116	16.756384	14.127853
1	-9.867206	23.199263	16.828844
1	-10.849591	21.723026	17.000856
1	-9.590080	22.098849	18.209857
1	-7.397758	23.020002	16.187255

1	-7.136352	21.929012	17.585557
1	-6.667729	21.407329	15.942496
1	-8.600683	19.771579	17.744910
1	-9.807780	19.448330	16.470526
1	-8.070611	19.348836	16.094983
1	-9.053593	21.778440	6.865120
1	-9.473492	21.252697	8.521006
1	-8.647258	22.808033	8.277139
1	-5.349488	21.239817	7.224983
1	-6.652813	21.627126	6.068182
1	-6.228626	22.789935	7.358246
1	-6.540913	19.059886	7.862983
1	-8.242078	19.113256	8.386548
1	-7.824138	19.447543	6.685243
19	-10.051418	27.231220	13.038273
8	-8.068946	26.849321	11.555373
1	-7.440826	27.290238	10.946090

157

Int8, E = -19489.370017, G = 19488.266682

29	-7.111691	25.305964	12.279340
29	-6.563780	23.407707	10.354001
29	-8.717823	21.337402	11.010731
29	-6.879949	19.557037	11.354947
16	-6.224173	23.334172	12.681914
16	-6.609266	21.183603	9.802787
16	-6.829386	24.974293	8.633843
16	-10.208231	19.465830	11.029412
7	-6.066819	27.426991	9.661245
7	-4.385968	26.074607	9.174135
7	-7.942767	17.934982	10.717010
7	-9.770158	16.987876	9.932862
6	-5.743122	26.234222	9.185069
6	-4.392998	23.347636	13.013576
6	-9.252040	18.084986	10.545709
6	-4.888860	28.056033	9.964918
6	-3.831335	27.232683	9.668320
6	-7.222574	20.945343	8.053551
6	-7.611821	16.707419	10.209135
6	-8.738234	16.103309	9.714695
6	-3.758656	22.302161	12.106686
6	-11.158313	16.788164	9.588973
6	-3.671618	24.895868	8.747599
6	-3.866532	24.739292	12.698629
6	-4.200658	22.990886	14.481233

6	-6.163506	21.562129	7.146220
6	-8.553300	21.673175	7.901107
6	-7.385153	19.462284	7.761293
29	-8.537054	25.061751	10.205102
29	-8.899688	23.648714	13.727674
29	-7.251602	21.377914	13.347198
29	-9.184342	19.698114	13.096015
16	-9.536235	23.451242	11.472725
16	-9.352949	21.483594	14.485745
16	-8.509196	25.316401	15.290760
16	-5.858898	19.448688	13.431697
7	-11.203637	25.891002	15.208475
7	-10.396575	25.647057	17.257289
7	-8.202747	18.065114	13.840690
7	-6.426978	17.072529	14.685186
6	-10.109583	25.623968	15.919410
6	-11.386525	23.440370	11.276952
6	-6.887963	18.157440	14.008929
6	-12.213184	26.082100	16.115035
6	-11.734294	25.939729	17.391313
6	-8.877638	21.237733	16.276463
6	-8.596318	16.885966	14.414723
6	-7.502825	16.254597	14.947450
6	-11.974047	22.531157	12.344513
6	-5.050629	16.822480	15.044158
6	-9.465632	25.464842	18.343126
6	-11.882015	24.869314	11.444503
6	-11.688159	22.919643	9.878646
6	-9.876427	22.065601	17.077345
6	-7.461336	21.753292	16.496873
6	-8.980294	19.765725	16.641870
1	-3.126085	22.969470	14.729969
1	-4.693762	23.725802	15.136469
1	-4.625758	21.997330	14.694423
1	-2.772948	24.774374	12.840400
1	-4.088998	25.013473	11.656276
1	-4.324610	25.494523	13.355006
1	-2.679485	22.215983	12.318869
1	-4.225508	21.316879	12.254509
1	-3.890027	22.572090	11.047842
1	-2.754245	27.356834	9.760202
1	-4.875689	29.057349	10.394583
1	-3.704445	24.785414	7.653036
1	-2.626815	24.971875	9.072806

1	-4.129476	24.003335	9.199674
1	-9.953723	24.922638	19.164211
1	-8.606797	24.885156	17.981134
1	-9.102063	26.434641	18.718924
1	-12.209637	26.012634	18.367485
1	-13.232701	26.309541	15.801764
1	-11.361912	25.540366	10.742661
1	-12.965256	24.929707	11.244898
1	-11.711605	25.215738	12.475950
1	-11.304347	21.894790	9.758804
1	-12.777178	22.907914	9.701458
1	-11.218492	23.556512	9.111911
1	-11.785878	22.936726	13.349884
1	-13.062917	22.426400	12.201166
1	-11.510546	21.533926	12.307325
1	-6.588174	16.336166	10.223557
1	-8.902521	15.139648	9.237363
1	-11.785139	16.796962	10.492824
1	-11.504654	17.582863	8.912357
1	-11.264026	15.818580	9.087528
1	-7.388254	15.312553	15.479278
1	-9.637626	16.567381	14.417643
1	-4.663454	17.638196	15.671737
1	-4.995139	15.881512	15.604646
1	-4.424724	16.743223	14.143083
1	-9.835298	23.116672	16.761014
1	-10.905358	21.701044	16.930852
1	-9.642333	22.013126	18.154589
1	-7.381242	22.800685	16.168082
1	-7.193122	21.696457	17.566493
1	-6.734308	21.150990	15.928062
1	-8.779239	19.632402	17.718991
1	-9.984797	19.367275	16.429721
1	-8.251700	19.167767	16.079215
1	-8.870620	21.689300	6.844147
1	-9.337812	21.169694	8.489181
1	-8.477385	22.708460	8.269050
1	-5.192785	21.055963	7.267370
1	-6.469310	21.468288	6.089836
1	-6.030371	22.630455	7.375408
1	-6.439866	18.917204	7.908123
1	-8.143781	19.013920	8.416420
1	-7.704188	19.319357	6.714480
19	-9.892527	27.268933	13.133481

8	-8.119446	26.714632	11.401541
1	-7.483610	27.179666	10.819060
6	-9.960256	29.631654	9.089603
6	-11.023148	29.647377	10.238820
6	-6.564929	29.186540	14.185390
6	-5.815283	30.349730	13.454360
5	-8.907537	29.745796	11.096631
5	-7.677448	29.771657	12.284995
8	-8.763621	30.065645	9.779472
8	-10.201549	29.383043	11.402949
8	-7.855841	29.210580	13.526371
8	-6.442269	30.339431	12.148120
6	-9.675582	28.230029	8.563020
1	-10.510459	27.848363	7.957551
1	-8.775254	28.263859	7.931743
1	-9.475054	27.526504	9.386147
6	-10.245290	30.585083	7.947612
1	-9.450299	30.504174	7.191108
1	-11.201588	30.332236	7.465453
1	-10.287595	31.628208	8.288347
6	-12.091406	28.579131	10.130471
1	-12.777637	28.645339	10.988772
1	-12.681795	28.720738	9.212779
1	-11.660298	27.570594	10.110353
6	-11.656748	31.015069	10.452015
1	-12.351292	31.267459	9.637998
1	-12.222125	31.003441	11.395964
1	-10.892658	31.803874	10.520186
6	-6.096247	31.715657	14.066357
1	-5.593497	31.831869	15.037287
1	-5.720928	32.496176	13.387786
1	-7.175709	31.875705	14.208381
6	-4.323530	30.139454	13.291392
1	-3.884017	30.996524	12.759119
1	-3.838069	30.064262	14.276098
1	-4.099433	29.229699	12.719720
6	-6.773023	29.384829	15.672861
1	-7.293743	28.509133	16.089707
1	-5.805261	29.481791	16.187559
1	-7.376291	30.277762	15.884866
6	-5.939109	27.825934	13.914056
1	-4.979840	27.719020	14.440378
1	-6.621162	27.039144	14.270880
1	-5.775931	27.665337	12.838447

157
Int9, E = -19489.376401, G = 19488.271001

29	-7.642124	25.525854	12.497807
29	-6.698259	23.072584	9.931990
29	-8.936573	21.121395	10.756155
29	-7.070407	19.398362	11.183871
16	-6.492297	23.436871	12.204999
16	-6.870512	20.877989	9.477668
16	-6.875573	24.723878	8.318289
16	-10.375914	19.249156	10.972223
7	-5.709746	26.868199	9.616015
7	-4.275495	25.495434	8.653650
7	-8.108403	17.719267	10.680314
7	-9.940998	16.736869	9.953987
6	-5.590658	25.755027	8.905785
6	-4.726773	23.555049	12.781574
6	-9.421217	17.854882	10.525491
6	-4.442982	27.346547	9.816929
6	-3.536020	26.507654	9.221634
6	-7.584640	20.608341	7.772673
6	-7.776058	16.479986	10.202040
6	-8.906013	15.853606	9.744875
6	-3.885265	22.635892	11.906666
6	-11.332126	16.520413	9.632948
6	-3.762200	24.342268	7.953405
6	-4.294106	25.005761	12.625360
6	-4.672564	23.126222	14.241907
6	-6.547601	21.140016	6.790077
6	-8.881784	21.400711	7.654626
6	-7.835122	19.123909	7.560113
29	-8.515360	24.899756	9.986533
29	-9.072820	23.547432	13.405799
29	-7.452460	21.474916	13.055439
29	-9.298052	19.633172	12.998457
16	-9.645901	23.301133	11.127249
16	-9.663703	21.491020	14.236153
16	-7.906348	25.056571	14.759824
16	-5.966412	19.602373	13.196875
7	-10.221698	26.070391	15.929579
7	-8.445858	25.970269	17.247834
7	-8.202021	18.135736	13.848752
7	-6.321415	17.261876	14.591639
6	-8.935864	25.747817	15.993550
6	-11.478137	23.547265	10.939662

6	-6.884744	18.297989	13.917614
6	-10.564212	26.516572	17.179913
6	-9.475643	26.461209	18.012434
6	-9.215904	21.307365	16.040442
6	-8.491663	16.957384	14.485096
6	-7.331451	16.399907	14.954864
6	-12.189088	22.308486	11.459472
6	-4.910368	17.085465	14.843304
6	-7.096648	25.715011	17.692212
6	-11.849543	24.778683	11.753529
6	-11.761393	23.764077	9.458503
6	-9.966324	22.407475	16.781790
6	-7.713294	21.482633	16.211577
6	-9.665496	19.932199	16.512267
1	-3.637837	23.197629	14.619079
1	-5.316570	23.768434	14.861892
1	-5.012717	22.085190	14.355515
1	-3.243933	25.128979	12.941194
1	-4.377205	25.333073	11.579169
1	-4.921747	25.670565	13.241261
1	-2.829578	22.661818	12.227386
1	-4.239726	21.595584	11.972250
1	-3.936091	22.947958	10.851201
1	-2.450158	26.527862	9.157639
1	-4.256478	28.255034	10.389105
1	-3.960832	24.412202	6.873256
1	-2.679981	24.275621	8.119197
1	-4.247474	23.433353	8.338922
1	-7.000284	26.038303	18.735910
1	-6.863616	24.641925	17.623216
1	-6.375356	26.269756	17.074845
1	-9.339241	26.718219	19.060856
1	-11.574124	26.853628	17.414492
1	-11.269272	25.647072	11.404041
1	-12.924472	25.003860	11.646860
1	-11.637844	24.594342	12.821013
1	-11.463087	22.883645	8.868359
1	-12.837996	23.942033	9.295140
1	-11.202237	24.634119	9.078346
1	-11.900503	22.096253	12.500641
1	-13.281703	22.455859	11.418484
1	-11.936968	21.429401	10.847837
1	-6.749664	16.116333	10.210750
1	-9.070906	14.874628	9.300047

1	-11.947500	16.543085	10.544375
1	-11.692351	17.299172	8.945089
1	-11.437457	15.540315	9.152321
1	-7.131197	15.484211	15.506979
1	-9.512093	16.587535	14.573227
1	-4.499556	17.970815	15.348917
1	-4.771125	16.207867	15.485982
1	-4.364683	16.932387	13.900157
1	-9.683062	23.394544	16.390484
1	-11.056080	22.294441	16.669140
1	-9.726616	22.375931	17.858662
1	-7.399119	22.458401	15.809987
1	-7.437863	21.434894	17.279760
1	-7.161319	20.695334	15.675221
1	-9.426873	19.800452	17.581845
1	-10.752204	19.803063	16.386657
1	-9.159378	19.140521	15.942632
1	-9.249350	21.389029	6.614089
1	-9.664592	20.967465	8.299090
1	-8.729357	22.445113	7.969696
1	-5.595518	20.593756	6.880226
1	-6.912226	21.026517	5.754580
1	-6.354871	22.208158	6.976737
1	-6.908454	18.539996	7.670931
1	-8.568469	18.744451	8.285117
1	-8.231136	18.950119	6.544844
19	-10.673812	27.312703	13.573250
8	-8.005136	26.740765	10.917412
1	-7.129560	26.936675	10.450934
6	-9.772715	28.828449	9.078693
6	-10.880843	28.571862	10.153888
6	-7.925017	29.877658	14.533980
6	-6.640099	30.153570	13.677681
5	-8.761815	28.063999	11.053486
5	-8.113032	28.890213	12.448763
8	-8.600435	28.891236	9.862379
8	-10.208186	27.734963	11.078601
8	-8.836525	29.343244	13.547347
8	-6.784672	29.227056	12.588222
6	-9.664100	27.671990	8.083177
1	-10.498312	27.654591	7.364686
1	-8.723850	27.778485	7.520817
1	-9.626298	26.704149	8.604081
6	-9.928066	30.134447	8.313563

1	-9.129462	30.220731	7.559673
1	-10.895210	30.181619	7.788408
1	-9.847994	31.000939	8.984574
6	-12.119970	27.864738	9.629571
1	-12.852361	27.729435	10.441613
1	-12.603291	28.457392	8.836962
1	-11.878858	26.872492	9.226708
6	-11.293660	29.861688	10.867760
1	-11.856169	30.541831	10.210638
1	-11.947597	29.614962	11.720494
1	-10.412493	30.392165	11.257095
6	-6.617044	31.550918	13.068752
1	-6.447169	32.326890	13.829333
1	-5.800559	31.603294	12.332868
1	-7.558979	31.772234	12.544316
6	-5.330707	29.870142	14.389797
1	-4.489435	30.087303	13.713987
1	-5.223000	30.504576	15.282656
1	-5.257029	28.817198	14.694181
6	-8.551519	31.108406	15.161062
1	-9.442991	30.817126	15.737752
1	-7.845959	31.594744	15.851713
1	-8.861256	31.839240	14.402306
6	-7.719934	28.799196	15.588092
1	-7.047730	29.138455	16.389313
1	-8.689451	28.552774	16.047085
1	-7.309804	27.881631	15.141635

157

TS8-9, E = -19489.361645, G = 19488.251836

29	-7.102786	25.685106	12.167740
29	-6.891139	23.511785	10.431791
29	-8.820692	21.510993	11.020290
29	-6.917763	19.762033	11.375946
16	-6.396679	23.649852	12.726513
16	-6.718453	21.330739	9.760254
16	-7.073129	24.987134	8.602433
16	-10.216397	19.542043	11.062736
7	-5.839284	27.244609	9.609772
7	-4.446189	25.682761	8.900696
7	-7.906012	18.082147	10.758348
7	-9.684795	17.120068	9.884657
6	-5.754502	26.040551	9.063692
6	-4.544079	23.557012	12.931255
6	-9.213035	18.204673	10.554109

6	-4.554954	27.678670	9.801024
6	-3.673399	26.723872	9.360481
6	-7.393617	21.050210	8.037016
6	-7.528085	16.884503	10.213418
6	-8.623643	16.271437	9.663384
6	-4.013244	22.519185	11.954151
6	-11.055739	16.903739	9.485876
6	-3.968243	24.428792	8.369060
6	-3.965126	24.930216	12.630353
6	-4.286733	23.148353	14.375693
6	-6.387736	21.675425	7.076336
6	-8.750563	21.734209	7.915629
6	-7.522328	19.558142	7.775448
29	-8.728133	25.321372	10.256276
29	-9.100868	23.686410	13.790291
29	-7.373080	21.619598	13.360189
29	-9.184939	19.792424	13.113853
16	-9.684519	23.644407	11.512298
16	-9.526138	21.537113	14.506463
16	-8.445021	25.267416	15.345322
16	-5.872375	19.749243	13.435659
7	-11.040531	26.157757	15.623343
7	-10.003969	25.775648	17.543556
7	-8.118124	18.223428	13.876220
7	-6.269088	17.328113	14.670509
6	-9.902192	25.746591	16.179463
6	-11.542540	23.635958	11.329093
6	-6.809642	18.394628	14.025101
6	-11.888893	26.451937	16.658348
6	-11.265540	26.226127	17.857810
6	-9.056229	21.281150	16.298525
6	-8.426931	17.009537	14.429878
6	-7.286504	16.438290	14.931106
6	-12.099035	22.392252	12.000322
6	-4.872640	17.154280	14.995259
6	-8.967748	25.447794	18.491033
6	-12.084579	24.884772	12.007772
6	-11.848883	23.640422	9.837608
6	-9.981379	22.187460	17.101961
6	-7.602220	21.684526	16.505730
6	-9.276142	19.822621	16.668049
1	-3.201929	23.073582	14.561282
1	-4.708921	23.885842	15.076050
1	-4.743381	22.169357	14.589512

1	-2.863857	24.897563	12.690030
1	-4.242382	25.259733	11.617228
1	-4.324896	25.681278	13.348545
1	-2.937735	22.345367	12.127741
1	-4.547439	21.563483	12.058146
1	-4.152548	22.856407	10.916589
1	-2.586435	26.682344	9.337842
1	-4.337713	28.648615	10.248340
1	-4.023834	24.414164	7.269738
1	-2.926547	24.280674	8.679824
1	-4.586948	23.609282	8.762693
1	-9.415605	24.998411	19.387458
1	-8.275478	24.731725	18.029460
1	-8.399833	26.345335	18.784109
1	-11.594331	26.335731	18.889338
1	-12.905185	26.808171	16.486556
1	-11.672650	25.786786	11.534568
1	-13.181611	24.930312	11.904493
1	-11.844250	24.882400	13.083205
1	-11.426521	22.747552	9.351215
1	-12.939609	23.642177	9.672033
1	-11.422155	24.531200	9.348899
1	-11.787101	22.337946	13.054148
1	-13.201123	22.405939	11.955953
1	-11.748901	21.483004	11.493020
1	-6.495627	16.539511	10.242805
1	-8.749266	15.322334	9.146849
1	-11.715194	16.887519	10.365927
1	-11.390015	17.704200	8.809742
1	-11.126810	15.941180	8.965267
1	-7.104261	15.495122	15.441695
1	-9.446112	16.626313	14.443308
1	-4.517435	17.986954	15.619256
1	-4.750905	16.214742	15.547570
1	-4.264878	17.115293	14.079156
1	-9.851784	23.231914	16.788661
1	-11.037287	21.909793	16.957154
1	-9.750092	22.112089	18.178415
1	-7.444288	22.720609	16.168674
1	-7.332841	21.615175	17.574276
1	-6.927580	21.024687	15.937143
1	-9.053218	19.668464	17.737995
1	-10.319110	19.516664	16.489315
1	-8.623767	19.164841	16.079371

1	-9.103029	21.711601	6.870027
1	-9.500361	21.222666	8.541028
1	-8.694406	22.781544	8.248619
1	-5.393957	21.213228	7.184603
1	-6.722401	21.526352	6.035079
1	-6.295815	22.755796	7.259372
1	-6.558509	19.041802	7.902949
1	-8.250537	19.100773	8.457190
1	-7.866429	19.390233	6.740403
19	-9.940770	27.099525	13.246696
8	-8.052385	27.121969	11.179485
1	-7.314652	27.345662	10.552638
6	-9.527954	29.319515	8.996074
6	-10.752367	28.981535	9.951769
6	-6.709182	29.069794	14.488844
6	-6.085025	30.294345	13.747526
5	-8.760831	29.022829	11.153071
5	-7.757472	29.389328	12.486649
8	-8.440162	29.515717	9.897177
8	-10.146153	28.796247	11.237008
8	-7.950288	28.899304	13.766123
8	-6.603037	30.127037	12.410668
6	-9.151051	28.186760	8.047905
1	-9.927577	28.007061	7.289966
1	-8.218651	28.457070	7.529390
1	-8.958992	27.252833	8.590825
6	-9.700934	30.599884	8.190395
1	-8.790864	30.777561	7.597152
1	-10.550521	30.524680	7.495420
1	-9.853869	31.471988	8.840215
6	-11.481913	27.701450	9.572763
1	-12.310572	27.524300	10.274575
1	-11.906486	27.764734	8.560019
1	-10.803630	26.836344	9.616920
6	-11.756759	30.117504	10.100106
1	-12.288370	30.320856	9.159121
1	-12.502426	29.836755	10.859949
1	-11.267173	31.042845	10.435304
6	-6.605312	31.628299	14.267927
1	-6.191626	31.864683	15.259154
1	-6.307640	32.425857	13.570714
1	-7.703634	31.626729	14.336971
6	-4.570074	30.301299	13.693489
1	-4.221794	31.196415	13.156051

1	-4.145901	30.326905	14.708780
1	-4.179417	29.417600	13.171479
6	-7.015088	29.281810	15.956853
1	-7.436559	28.357387	16.380386
1	-6.096298	29.523601	16.512433
1	-7.741684	30.091583	16.108210
6	-5.894574	27.798558	14.290113
1	-4.957881	27.821250	14.865685
1	-6.495252	26.939869	14.624930
1	-5.651710	27.648983	13.226775

134

Int10, E = -19003.085721, G = 19002.167356

29	-7.225824	25.368731	13.372059
29	-6.636662	23.319729	10.508031
29	-8.532376	21.452603	11.012780
29	-6.860180	19.510273	11.431513
16	-6.358470	23.181293	12.883633
16	-6.284023	21.140200	9.980896
16	-7.473042	24.906090	8.953289
16	-10.122335	19.651011	10.864830
7	-6.421136	26.764596	7.258952
7	-5.006525	26.176250	8.862167
7	-7.958960	17.962730	10.684313
7	-9.832406	17.087966	9.926943
6	-6.252953	26.001946	8.328582
6	-4.626375	23.064638	13.556873
6	-9.248441	18.190814	10.466053
6	-5.246573	27.446917	7.095779
6	-4.353096	27.095907	8.077501
6	-6.631812	20.797275	8.180611
6	-7.706272	16.677539	10.282451
6	-8.863452	16.117566	9.807986
6	-3.843658	22.127518	12.648391
6	-11.227957	16.952615	9.581100
6	-4.464282	25.524242	10.026674
6	-4.019692	24.461317	13.571832
6	-4.714578	22.516632	14.975580
6	-5.716143	21.725112	7.388916
6	-8.089447	21.111310	7.873854
6	-6.312115	19.337599	7.892521
29	-7.943876	25.464274	11.132446
29	-8.914780	23.412782	13.988491
29	-7.469263	21.239094	13.517647
29	-9.462788	19.690007	13.074377

16	-9.355719	23.567285	11.667654
16	-9.768479	21.377110	14.537571
16	-7.807532	24.809569	15.545908
16	-6.183425	19.236039	13.620272
7	-9.826140	26.655974	16.062090
7	-8.904686	25.733017	17.849793
7	-8.619712	17.962592	13.761592
7	-6.920020	16.701035	14.368554
6	-8.907408	25.797490	16.486257
6	-11.103907	23.587824	11.022829
6	-7.304105	17.932982	13.941955
6	-10.426059	27.160508	17.186548
6	-9.867060	26.601178	18.306469
6	-9.465076	20.956457	16.329516
6	-9.094226	16.716085	14.075956
6	-8.048124	15.917027	14.455830
6	-11.928281	22.622723	11.861694
6	-5.564183	16.284991	14.640944
6	-8.028393	24.926529	18.667254
6	-11.667410	24.998900	11.128116
6	-11.047104	23.156174	9.562214
6	-10.188203	22.026716	17.140723
6	-7.971825	20.997458	16.620370
6	-10.040500	19.574667	16.604656
1	-3.705698	22.451668	15.418349
1	-5.332187	23.171044	15.610211
1	-5.160110	21.510031	14.982491
1	-2.991146	24.422447	13.969564
1	-3.978465	24.894966	12.563661
1	-4.611170	25.137570	14.210383
1	-2.831660	21.953570	13.052749
1	-4.360003	21.160141	12.551431
1	-3.749674	22.551627	11.637231
1	-3.334537	27.410965	8.295620
1	-5.100448	28.159989	6.283196
1	-4.191649	24.480005	9.802991
1	-3.571502	26.067302	10.360498
1	-5.212352	25.533820	10.831724
1	-6.994301	25.299630	18.619288
1	-8.376712	24.966618	19.706632
1	-8.040345	23.883379	18.323303
1	-10.060890	26.734493	19.368870
1	-11.228883	27.895970	17.130277
1	-10.996377	25.735304	10.655670

1	-12.644528	25.056460	10.620652
1	-11.851942	25.261966	12.181720
1	-10.646525	22.134720	9.474434
1	-12.058983	23.169851	9.122444
1	-10.402502	23.830705	8.977269
1	-12.002002	22.975154	12.901381
1	-12.946491	22.525358	11.447686
1	-11.459284	21.629105	11.892366
1	-6.712796	16.237405	10.354238
1	-9.085959	15.133947	9.400079
1	-11.382599	15.977259	9.104114
1	-11.857407	17.017868	10.481161
1	-11.528436	17.746096	8.882240
1	-8.000014	14.879101	14.777795
1	-10.153364	16.471332	14.011472
1	-5.110480	16.928703	15.408024
1	-5.576781	15.249970	15.003058
1	-4.953479	16.340010	13.727482
1	-9.793033	23.023614	16.891667
1	-11.270168	22.023215	16.934860
1	-10.042606	21.852949	18.220787
1	-7.562963	21.985707	16.358896
1	-7.780926	20.809842	17.691551
1	-7.434984	20.236818	16.032936
1	-9.882694	19.298612	17.661569
1	-11.122903	19.545669	16.402078
1	-9.554790	18.819449	15.969930
1	-8.300298	20.957163	6.801136
1	-8.762499	20.465334	8.457970
1	-8.313397	22.159499	8.125715
1	-4.655553	21.523435	7.607625
1	-5.876579	21.586538	6.305760
1	-5.928426	22.775670	7.641906
1	-5.257279	19.109747	8.113742
1	-6.939026	18.673611	8.504076
1	-6.495100	19.110025	6.828161
19	-10.183702	26.910478	13.404866
6	-7.399743	29.425961	12.683510
6	-6.061267	29.127260	11.938500
5	-7.239027	27.160685	12.214794
8	-8.143152	28.214460	12.435122
8	-5.972182	27.693688	12.039947
6	-6.121803	29.466591	10.453954
1	-6.109043	30.553121	10.283390

1	-5.246966	29.028162	9.951778
1	-7.025048	29.046673	9.986785
6	-4.829075	29.743324	12.571564
1	-3.939387	29.485378	11.977021
1	-4.912552	30.840490	12.601379
1	-4.672643	29.373410	13.593820
6	-8.181822	30.609602	12.150474
1	-9.103539	30.743640	12.737668
1	-7.590124	31.533954	12.234347
1	-8.464326	30.467835	11.098840
6	-7.228653	29.537340	14.195216
1	-6.707608	30.463687	14.477809
1	-8.220410	29.547286	14.673458
1	-6.668896	28.678537	14.595671

148

Int11, E = -19310.907000, G = 19309.887292

29	-7.722639	25.029429	14.046098
29	-6.678553	23.032758	11.006448
29	-8.681873	20.979940	10.696129
29	-6.964902	19.144486	11.227279
16	-6.769901	22.779387	13.330102
16	-6.351074	20.989984	10.056229
16	-6.993381	24.969555	9.732212
16	-10.107212	19.150387	10.063898
7	-4.728117	25.817642	11.078990
7	-5.511203	27.244374	9.577726
7	-7.799750	17.653255	10.111243
7	-9.340946	16.865870	8.749288
6	-5.678597	26.029716	10.180328
6	-5.051992	22.367403	13.954659
6	-9.024207	17.849391	9.632913
6	-3.934564	26.933524	11.061490
6	-4.401748	27.833712	10.136373
6	-6.478112	21.026321	8.193638
6	-7.320585	16.510938	9.527189
6	-8.267707	16.006761	8.674889
6	-5.206619	21.664806	15.296838
6	-10.588994	16.743426	8.033664
6	-6.334506	27.791341	8.528997
6	-4.371997	21.474326	12.928936
6	-4.266476	23.659538	14.117730
6	-5.313560	21.887433	7.715666
6	-7.799788	21.663493	7.784292
6	-6.371043	19.606887	7.658602

29	-8.319200	24.880442	11.727708
29	-9.595957	22.810519	13.793988
29	-7.920183	20.736268	13.357904
29	-9.670234	19.098649	12.339701
16	-9.596112	22.943411	11.449131
16	-10.309468	20.614478	13.892467
16	-9.550084	24.390694	15.449932
16	-6.578298	18.730329	13.461361
7	-8.145354	22.873860	17.286297
7	-10.028929	23.755248	18.051611
7	-8.912400	17.336108	13.058768
7	-7.318575	16.141435	13.999095
6	-9.185406	23.630173	16.981199
6	-11.238741	23.398448	10.721035
6	-7.666440	17.363504	13.516280
6	-8.325186	22.489903	18.586381
6	-9.489545	23.023670	19.081923
6	-10.467328	19.866644	15.593492
6	-9.375878	16.059891	13.240943
6	-8.395775	15.301965	13.826639
6	-12.116070	22.156546	10.685934
6	-6.035793	15.787673	14.559866
6	-11.280798	24.470832	18.076417
6	-11.847083	24.475028	11.609720
6	-10.989664	23.938195	9.318087
6	-11.272098	20.860324	16.423151
6	-9.082941	19.660889	16.190434
6	-11.210554	18.543329	15.469544
1	-3.219669	23.425704	14.379023
1	-4.271275	24.245438	13.185836
1	-4.685470	24.281252	14.920561
1	-3.406905	21.107729	13.320108
1	-5.000423	20.611076	12.669077
1	-4.186414	22.026371	11.995214
1	-4.213684	21.431754	15.719631
1	-5.752572	22.298867	16.011521
1	-5.765078	20.722738	15.185453
1	-4.055226	28.816796	9.823586
1	-3.066049	27.032481	11.714075
1	-6.224279	27.210743	7.599989
1	-7.393414	27.771655	8.823556
1	-6.033446	28.829028	8.339573
1	-11.987285	24.041544	17.349349
1	-11.713738	24.394725	19.081522

1	-11.123519	25.533253	17.839087
1	-9.974432	22.960628	20.053910
1	-7.607335	21.851869	19.102629
1	-11.159276	25.336159	11.663259
1	-12.815044	24.817536	11.206407
1	-12.012286	24.062601	12.620047
1	-10.504735	23.176441	8.687982
1	-11.942360	24.226249	8.841280
1	-10.331545	24.821112	9.350292
1	-12.220021	21.719486	11.691187
1	-13.119483	22.407476	10.301366
1	-11.676589	21.392298	10.027154
1	-6.326442	16.125658	9.748957
1	-8.280972	15.129358	8.031872
1	-10.534067	15.872203	7.369834
1	-11.425832	16.610492	8.735271
1	-10.775424	17.643737	7.430444
1	-8.363475	14.259092	14.134399
1	-10.382250	15.765791	12.946311
1	-5.797490	16.437890	15.413757
1	-6.072620	14.746110	14.901216
1	-5.242252	15.892385	13.805065
1	-10.783798	21.844345	16.419678
1	-12.288796	20.985393	16.017929
1	-11.357508	20.512377	17.466808
1	-8.538008	20.615482	16.247200
1	-9.160813	19.246929	17.211162
1	-8.492872	18.966968	15.575024
1	-11.341202	18.084455	16.465013
1	-12.208797	18.687103	15.026498
1	-10.651353	17.841881	14.834582
1	-7.853754	21.765009	6.686234
1	-8.653159	21.048548	8.114405
1	-7.897363	22.663483	8.232974
1	-4.346334	21.455626	8.017411
1	-5.325830	21.968664	6.614911
1	-5.390757	22.900697	8.141380
1	-5.432028	19.127609	7.976934
1	-7.205849	18.989786	8.018126
1	-6.395219	19.615135	6.555183
19	-11.497685	26.319155	14.250641
5	-8.323113	26.713787	12.696579
8	-9.557320	27.371986	12.919750
8	-7.390531	27.680861	12.352874

6	-9.470706	28.718371	12.405850
6	-7.940980	28.994544	12.517496
6	-10.347368	29.637055	13.235878
6	-9.966121	28.667596	10.965108
6	-7.528723	29.498265	13.895631
6	-7.386040	29.910819	11.444026
1	-10.197574	30.685441	12.935961
1	-11.412548	29.404087	13.073085
1	-10.127785	29.554059	14.309723
1	-10.990130	28.263236	10.951969
1	-9.985565	29.665237	10.502942
1	-9.332760	28.004432	10.356461
1	-6.431983	29.461248	13.973968
1	-7.852011	30.535942	14.064156
1	-7.943779	28.863199	14.690672
1	-7.857195	30.904189	11.500884
1	-6.302879	30.038332	11.590357
1	-7.547016	29.503025	10.438736
6	-7.163950	25.826331	19.435371
6	-6.596312	25.730070	18.167867
6	-7.174291	26.412675	17.082915
6	-8.334105	27.183133	17.292384
6	-8.891952	27.275810	18.563675
6	-8.310781	26.597234	19.637400
1	-6.710432	25.289495	20.272249
1	-5.704896	25.120665	18.004150
1	-8.789587	27.705804	16.448890
1	-9.790519	27.878859	18.717507
1	-8.754762	26.666521	20.633860
6	-6.603260	26.329438	15.778007
6	-6.113243	26.331841	14.650869
1	-5.522007	26.528046	13.767648

148

TS11-12, E = -19310.886146, G = 19309.862796

29	-7.999898	24.966454	14.402769
29	-6.887524	23.157697	11.065993
29	-8.711537	21.119323	10.781935
29	-6.942179	19.339998	11.368441
16	-7.080548	23.025309	13.432452
16	-6.322746	21.175552	10.188305
16	-7.274040	25.154002	9.892538
16	-10.010697	19.251582	10.023458
7	-4.717635	25.642489	10.801714
7	-5.326836	26.902095	9.084777

7	-7.650684	17.840394	10.167189
7	-9.048650	17.083222	8.642664
6	-5.703714	25.918853	9.961041
6	-5.350438	22.866601	14.124858
6	-8.842139	18.020584	9.605102
6	-3.684131	26.473915	10.466264
6	-4.037927	27.263320	9.401072
6	-6.421910	21.245837	8.322257
6	-7.081951	16.754011	9.557305
6	-7.938824	16.269286	8.604058
6	-5.487785	22.274704	15.519602
6	-10.233108	16.959716	7.826184
6	-6.079320	27.417972	7.967547
6	-4.568088	21.951056	13.196343
6	-4.722142	24.251286	14.152570
6	-5.313201	22.198397	7.887455
6	-7.775620	21.798217	7.896185
6	-6.201018	19.849738	7.761438
29	-8.352019	24.966500	12.017351
29	-9.900087	22.846802	13.767224
29	-8.076866	20.883752	13.504817
29	-9.656925	19.127068	12.307949
16	-9.704686	23.087506	11.461828
16	-10.474988	20.614535	13.805318
16	-10.331076	24.392039	15.369796
16	-6.601925	19.014790	13.624619
7	-8.937670	22.862109	17.197347
7	-10.739728	23.849648	18.024150
7	-8.807327	17.447469	13.114713
7	-7.208424	16.403226	14.213289
6	-9.952338	23.662474	16.918263
6	-11.360118	23.519761	10.746266
6	-7.602318	17.578774	13.657720
6	-9.067417	22.526265	18.516218
6	-10.178433	23.128687	19.051924
6	-10.785897	19.787281	15.449750
6	-9.193626	16.148580	13.314883
6	-8.209581	15.483658	13.998850
6	-12.179472	22.242169	10.639156
6	-5.950365	16.162654	14.880533
6	-11.926475	24.662190	18.124402
6	-12.016146	24.517807	11.691760
6	-11.122116	24.137526	9.373978
6	-11.683731	20.723921	16.248855

6	-9.461952	19.556350	16.163472
6	-11.500273	18.468288	15.183955
1	-3.693756	24.186201	14.547663
1	-4.683676	24.683466	13.140322
1	-5.297393	24.933930	14.794252
1	-3.583050	21.719768	13.635881
1	-5.102991	21.007871	13.014640
1	-4.413643	22.428208	12.216987
1	-4.497512	22.190180	15.998935
1	-6.129587	22.908060	16.149985
1	-5.940506	21.271478	15.470354
1	-3.506385	28.032490	8.844006
1	-2.737902	26.463518	11.008671
1	-5.682561	27.028193	7.016972
1	-7.126009	27.104323	8.065505
1	-6.031375	28.515761	7.951877
1	-12.462133	24.652746	17.165460
1	-12.585211	24.251008	18.900477
1	-11.681185	25.702193	18.397652
1	-10.615669	23.117747	20.048297
1	-8.355884	21.866935	19.014300
1	-11.373930	25.400601	11.833710
1	-12.985887	24.852621	11.285252
1	-12.188594	24.064257	12.680845
1	-10.611324	23.425206	8.707081
1	-12.082411	24.417934	8.907907
1	-10.495383	25.039549	9.450967
1	-12.275075	21.756603	11.622741
1	-13.188820	22.466749	10.253480
1	-11.699471	21.527514	9.952615
1	-6.092441	16.392787	9.833484
1	-7.867045	15.432542	7.912528
1	-10.088077	16.141603	7.110548
1	-11.112559	16.741450	8.450041
1	-10.416267	17.892578	7.273621
1	-8.128175	14.457032	14.349214
1	-10.152833	15.772821	12.961936
1	-5.818391	16.866492	15.714631
1	-5.946754	15.138154	15.271462
1	-5.111416	16.284457	14.179394
1	-11.215369	21.710510	16.359980
1	-12.655110	20.864127	15.748666
1	-11.871302	20.309266	17.254189
1	-8.939720	20.510900	16.327087

1	-9.631383	19.080413	17.145298
1	-8.809146	18.903850	15.567027
1	-11.722110	17.957414	16.137011
1	-12.452201	18.631184	14.654272
1	-10.875920	17.802829	14.571712
1	-7.811713	21.915023	6.798947
1	-8.592865	21.122564	8.196486
1	-7.948399	22.781630	8.359895
1	-4.322940	21.832723	8.201174
1	-5.310217	22.302873	6.788716
1	-5.473333	23.193567	8.331531
1	-5.241763	19.427245	8.099082
1	-7.003473	19.169147	8.076434
1	-6.191080	19.884184	6.658481
19	-10.683965	27.433964	15.592943
5	-8.211395	26.833550	13.322100
8	-9.454386	27.503385	13.301494
8	-7.295096	27.555182	12.577917
6	-9.408381	28.521488	12.276763
6	-7.873236	28.811026	12.181315
6	-10.253723	29.703388	12.714419
6	-9.983228	27.908982	11.006478
6	-7.402294	29.865389	13.175049
6	-7.388141	29.146344	10.786255
1	-10.173725	30.522220	11.983666
1	-11.314009	29.408810	12.765351
1	-9.948742	30.096207	13.695046
1	-11.011381	27.572479	11.204153
1	-10.013234	28.637514	10.183669
1	-9.388570	27.037323	10.691237
1	-6.302535	29.857015	13.207513
1	-7.731249	30.872029	12.878824
1	-7.776840	29.673030	14.192083
1	-7.887830	30.049116	10.403841
1	-6.303916	29.333478	10.804967
1	-7.578209	28.311837	10.101779
6	-5.334964	25.011892	19.168214
6	-5.674911	25.224955	17.835645
6	-6.950264	25.721741	17.485963
6	-7.864582	25.984913	18.530681
6	-7.517770	25.757540	19.859199
6	-6.251537	25.269661	20.191303
1	-4.337967	24.632705	19.410523
1	-4.951929	25.007525	17.047099

1	-8.864871	26.354173	18.290477
1	-8.249471	25.963674	20.645614
1	-5.982485	25.090538	21.235288
6	-7.336550	25.907270	16.119984
6	-7.551647	26.765018	15.177472
1	-7.333238	27.833265	15.074536

148

Int12, E = -19310.981841, G = 19309.951072

29	-7.501296	24.480086	14.164069
29	-6.343231	23.164158	11.918619
29	-8.824299	21.289141	11.056300
29	-7.209880	19.283922	11.170947
16	-6.992043	22.281040	13.974602
16	-6.464390	21.317232	10.536648
16	-5.922879	25.144841	10.797340
16	-10.308863	19.749383	10.022317
7	-4.185934	25.443855	12.912495
7	-4.364438	27.239995	11.627755
7	-8.109524	18.113154	9.771622
7	-9.733905	17.640206	8.361877
6	-4.800510	25.965868	11.862648
6	-5.486389	21.902880	15.017652
6	-9.326781	18.458932	9.367329
6	-3.340238	26.412775	13.377535
6	-3.434945	27.537283	12.595544
6	-6.511949	21.776109	8.726116
6	-7.724580	17.042656	9.009970
6	-8.725062	16.733872	8.125661
6	-5.921755	20.930035	16.103001
6	-11.004534	17.707112	7.678809
6	-4.784035	28.128752	10.569103
6	-4.462553	21.274840	14.081898
6	-4.964259	23.199614	15.614520
6	-5.148993	22.371306	8.392292
6	-7.620520	22.800453	8.513457
6	-6.777919	20.531878	7.894756
29	-8.130512	24.943527	11.626473
29	-9.575881	22.531801	14.353483
29	-8.122017	20.298883	13.540108
29	-9.965979	19.114407	12.231788
16	-9.586857	23.245730	12.081076
16	-10.458759	20.338173	14.075399
16	-10.615821	23.831402	15.961034
16	-6.849016	18.326395	13.255348

7	-9.042444	22.462824	17.761557
7	-10.059665	24.244777	18.599024
7	-9.262988	17.209886	12.536446
7	-7.764091	15.740528	13.204566
6	-9.849768	23.477803	17.483254
6	-11.320819	23.537510	11.456653
6	-8.026743	17.058107	12.998899
6	-8.727523	22.578646	19.089382
6	-9.346907	23.676315	19.631112
6	-10.648822	19.314060	15.624906
6	-9.806297	15.956082	12.433732
6	-8.886081	15.028404	12.845857
6	-12.170338	22.333060	11.826544
6	-6.517089	15.191780	13.683468
6	-10.842607	25.452615	18.668030
6	-11.878680	24.784915	12.121937
6	-11.227464	23.699112	9.945948
6	-11.527807	20.131934	16.564515
6	-9.287731	19.062198	16.257549
6	-11.327945	18.003257	15.254278
1	-4.048391	22.998850	16.196629
1	-4.724230	23.926642	14.823226
1	-5.707166	23.650288	16.289743
1	-3.571788	20.959249	14.651235
1	-4.881975	20.393061	13.573515
1	-4.146419	21.995350	13.310655
1	-5.058979	20.670316	16.739578
1	-6.703131	21.375795	16.737403
1	-6.317737	20.001734	15.662139
1	-2.930644	28.501072	12.626709
1	-2.706141	26.252907	14.250021
1	-3.970370	28.830844	10.346029
1	-5.007242	27.545525	9.665966
1	-5.681021	28.692972	10.861626
1	-11.849409	25.282798	18.260194
1	-10.928587	25.764342	19.716527
1	-10.364771	26.264089	18.095854
1	-9.348611	24.103916	20.631818
1	-8.068431	21.866856	19.588017
1	-11.228970	25.648531	11.926506
1	-12.878349	25.022354	11.722820
1	-11.990228	24.613411	13.205172
1	-10.836897	22.775323	9.491171
1	-12.223908	23.904050	9.519519

1	-10.554495	24.527203	9.674158
1	-12.281862	22.249444	12.916951
1	-13.170286	22.424888	11.370299
1	-11.704827	21.398487	11.483732
1	-6.756505	16.561787	9.142072
1	-8.815030	15.963901	7.362439
1	-11.047032	16.908353	6.928312
1	-11.831660	17.576116	8.391834
1	-11.122381	18.678521	7.176871
1	-8.922967	13.943695	12.919305
1	-10.822973	15.798043	12.076839
1	-6.241046	15.650528	14.643563
1	-6.635871	14.110597	13.823653
1	-5.709331	15.375230	12.959262
1	-11.064201	21.106603	16.772634
1	-12.521560	20.310069	16.123830
1	-11.666216	19.594293	17.518786
1	-8.801352	20.014737	16.513859
1	-9.399110	18.471967	17.184214
1	-8.629852	18.508284	15.570451
1	-11.484753	17.388312	16.157610
1	-12.310009	18.181671	14.788258
1	-10.713210	17.431332	14.545572
1	-7.589696	23.199813	7.485042
1	-8.608216	22.339701	8.675417
1	-7.528502	23.637866	9.221137
1	-4.345115	21.636780	8.557119
1	-5.120208	22.679118	7.332690
1	-4.948232	23.255345	9.016361
1	-6.009321	19.762269	8.064315
1	-7.755366	20.099023	8.146951
1	-6.777200	20.790181	6.821768
19	-10.252256	26.411748	14.343749
5	-7.983826	27.882056	12.523211
8	-8.968822	27.145590	11.860792
8	-7.754415	29.076703	11.899879
6	-9.316176	27.864611	10.629416
6	-8.808051	29.312497	10.949207
6	-10.808784	27.780752	10.386336
6	-8.567798	27.205003	9.484939
6	-9.849338	30.169265	11.660018
6	-8.246498	30.055549	9.753225
1	-11.083846	28.469827	9.573947
1	-11.090330	26.767187	10.070316

1	-11.394088	28.047795	11.276477
1	-8.837325	26.136879	9.442087
1	-8.847304	27.661350	8.524836
1	-7.480054	27.269362	9.614175
1	-9.361345	31.084655	12.025831
1	-10.666706	30.459435	10.985291
1	-10.280887	29.647039	12.527909
1	-9.021204	30.184105	8.982860
1	-7.908229	31.054098	10.067666
1	-7.392457	29.528835	9.308086
6	-4.823059	27.088835	17.412279
6	-5.410151	27.079319	16.146727
6	-6.641895	26.436025	15.923336
6	-7.261949	25.809957	17.021388
6	-6.694596	25.849995	18.293115
6	-5.464265	26.481693	18.493523
1	-3.853851	27.576054	17.554242
1	-4.896475	27.551017	15.305679
1	-8.201311	25.275374	16.854369
1	-7.207328	25.363356	19.127162
1	-5.004309	26.491946	19.485376
6	-7.274843	26.381302	14.583388
6	-7.340983	27.554610	13.886018
1	-6.915978	28.456788	14.366158

151

Int13, E = -19387.281458, G = 19386.232907

29	-5.387078	24.689769	13.739287
29	-6.103016	23.293342	11.373322
29	-8.187361	21.616097	11.710152
29	-6.852723	19.371047	11.553934
16	-5.402554	22.457803	13.503836
16	-6.151085	21.180148	10.420435
16	-6.801998	25.055619	9.986633
16	-10.100798	20.132982	11.267862
7	-4.126894	25.026204	9.316673
7	-5.307470	26.627624	8.338377
7	-8.256102	18.185616	10.648922
7	-10.291092	17.747745	9.930229
6	-5.321118	25.594538	9.234199
6	-3.584292	22.062374	13.325988
6	-9.500278	18.644539	10.578799
6	-3.322206	25.711779	8.446960
6	-4.035181	26.707284	7.824161
6	-6.610992	21.158384	8.610940

6	-8.247061	16.958378	10.039797
6	-9.507368	16.669795	9.585999
6	-3.454894	20.573892	13.043953
6	-11.707403	17.889780	9.687911
6	-6.418134	27.490220	8.019448
6	-3.020779	22.883322	12.174381
6	-2.903293	22.430283	14.639406
6	-5.588807	22.045987	7.910668
6	-8.013847	21.721150	8.433130
6	-6.524591	19.728962	8.096362
29	-7.335560	25.314957	12.243376
29	-9.213679	23.280090	15.159071
29	-6.782632	20.619270	13.921618
29	-9.194891	19.773465	13.354598
16	-8.748294	23.610563	12.874611
16	-8.737283	21.067846	15.154700
16	-10.085825	24.683000	16.757076
16	-6.067908	18.403691	13.517521
7	-12.497545	23.509907	16.144862
7	-12.507987	25.720435	15.980413
7	-8.752836	17.785166	13.671099
7	-7.417919	16.040463	13.829503
6	-11.764223	24.607890	16.273798
6	-10.446187	24.058265	12.238831
6	-7.483846	17.392328	13.696129
6	-13.739520	23.925980	15.748252
6	-13.770053	25.293455	15.637732
6	-9.042225	20.054198	16.693147
6	-9.521657	16.657237	13.784607
6	-8.706641	15.560392	13.884792
6	-11.427927	23.031673	12.785856
6	-6.208899	15.252317	13.871436
6	-12.057591	27.090447	15.989379
6	-10.791797	25.444654	12.760273
6	-10.406501	24.030724	10.718447
6	-9.139890	21.017988	17.868088
6	-7.890459	19.079023	16.881769
6	-10.365512	19.318608	16.510423
1	-1.823905	22.206725	14.583502
1	-3.025305	23.503532	14.857560
1	-3.328527	21.860458	15.480134
1	-1.941055	22.686387	12.059058
1	-3.526358	22.624927	11.231342
1	-3.156020	23.962537	12.353690

1	-2.396739	20.312890	12.872723
1	-3.819464	19.980822	13.896561
1	-4.040154	20.291559	12.155646
1	-3.761132	27.458795	7.086462
1	-2.270500	25.456864	8.311824
1	-7.265723	26.904708	7.633913
1	-6.750583	28.038845	8.914128
1	-6.102847	28.211588	7.255488
1	-11.477882	27.325949	15.081971
1	-12.930215	27.755071	16.024013
1	-11.433319	27.278015	16.873471
1	-14.556726	25.993493	15.363282
1	-14.551932	23.222256	15.562569
1	-10.114156	26.203459	12.343394
1	-11.824393	25.714966	12.482147
1	-10.716628	25.460632	13.858165
1	-10.167547	23.018304	10.356569
1	-11.387133	24.323219	10.305847
1	-9.642836	24.723372	10.331290
1	-11.525641	23.116770	13.878835
1	-12.425700	23.181512	12.339303
1	-11.092347	22.010048	12.562948
1	-7.339107	16.362116	9.961344
1	-9.915413	15.811843	9.056146
1	-12.052666	17.038355	9.089036
1	-12.262170	17.911708	10.637917
1	-11.911641	18.820645	9.139957
1	-8.917689	14.498411	13.989755
1	-10.609907	16.701788	13.787191
1	-5.576743	15.558024	14.717585
1	-6.478669	14.195735	13.988631
1	-5.636127	15.377114	12.940563
1	-8.183522	21.527831	18.048130
1	-9.902774	21.791191	17.689333
1	-9.407325	20.462736	18.783681
1	-6.937100	19.615224	17.010285
1	-8.059543	18.454747	17.776206
1	-7.793662	18.420622	16.008362
1	-10.591430	18.708774	17.402554
1	-11.191050	20.033162	16.364757
1	-10.326232	18.653175	15.635404
1	-8.290306	21.732024	7.364057
1	-8.752628	21.115236	8.979185
1	-8.060771	22.752427	8.815431

1	-4.569280	21.643353	8.020565
1	-5.816999	22.113130	6.832665
1	-5.601960	23.057977	8.338658
1	-5.515403	19.312667	8.244522
1	-7.240600	19.080476	8.618842
1	-6.750118	19.701192	7.016093
5	-5.027355	25.935598	16.653120
8	-6.264600	25.463653	17.038757
8	-4.022224	25.459108	17.443559
6	-6.040985	24.386928	17.985775
6	-4.621050	24.744631	18.547269
6	-6.054175	23.101187	17.177897
6	-7.149147	24.387490	19.017550
6	-3.757824	23.544109	18.879124
6	-4.670223	25.711220	19.722695
1	-5.918133	22.217015	17.816431
1	-7.022767	23.005243	16.664626
1	-5.276995	23.104293	16.400949
1	-8.105030	24.125745	18.540330
1	-6.941138	23.640786	19.798689
1	-7.263632	25.370564	19.493881
1	-2.787707	23.881504	19.274142
1	-4.241177	22.920662	19.646227
1	-3.568922	22.927014	17.990484
1	-5.050280	25.223191	20.631602
1	-3.652562	26.076421	19.926637
1	-5.306945	26.580417	19.498401
6	-5.053791	29.891706	12.303402
6	-5.270072	28.936719	13.300055
6	-4.856707	27.597748	13.131235
6	-4.217839	27.260093	11.925537
6	-3.963233	28.220997	10.951190
6	-4.390582	29.540186	11.127916
1	-5.402694	30.917519	12.452452
1	-5.775557	29.228769	14.226173
1	-3.926735	26.220283	11.756234
1	-3.443635	27.931252	10.034629
1	-4.214296	30.285979	10.348225
6	-5.098778	26.573463	14.171256
6	-4.829118	26.914639	15.463035
1	-4.377529	27.901520	15.680508
19	-8.123032	26.638271	15.398634
8	-7.888647	27.401918	12.722098
1	-7.080555	27.935112	12.599112

1	-8.529647	27.767095	12.092230

151

TS13-14, E = -19387.272118, G = 19386.223359

29	-5.476755	24.864804	13.543592
29	-6.256945	23.346721	11.490941
29	-8.271338	21.548811	11.721325
29	-6.797555	19.387631	11.610281
16	-5.506595	22.585961	13.617412
16	-6.159626	21.236961	10.508450
16	-7.026253	24.966626	9.969993
16	-10.088826	20.004163	11.224730
7	-4.304670	25.233855	9.638733
7	-5.517254	26.639739	8.427731
7	-8.131779	18.144779	10.678404
7	-10.139406	17.558065	9.990537
6	-5.533314	25.642547	9.361854
6	-3.688592	22.167143	13.431748
6	-9.400956	18.526845	10.596232
6	-3.471601	25.990555	8.860230
6	-4.202517	26.866484	8.096039
6	-6.535529	21.161188	8.681700
6	-8.052432	16.895458	10.121059
6	-9.294799	16.514897	9.686334
6	-3.570804	20.672303	13.184472
6	-11.563094	17.602624	9.754466
6	-6.669379	27.320582	7.889086
6	-3.115521	22.953624	12.262158
6	-2.998948	22.554125	14.733134
6	-5.560322	22.121942	8.010514
6	-7.972955	21.594560	8.431619
6	-6.302234	19.737192	8.196712
29	-7.730613	25.322432	12.145382
29	-9.398090	23.266833	15.125218
29	-6.886066	20.697367	13.989513
29	-9.270023	19.720367	13.370024
16	-8.957741	23.515562	12.841005
16	-8.903603	21.050874	15.160221
16	-10.258480	24.621019	16.762965
16	-6.099041	18.490119	13.623687
7	-12.709540	23.556978	16.120331
7	-12.610769	25.761521	15.911561
7	-8.757473	17.748615	13.684989
7	-7.348376	16.057808	13.759367
6	-11.928183	24.621497	16.244832

6	-10.664615	23.899595	12.190544
6	-7.473277	17.411689	13.717213
6	-13.920473	24.022764	15.685611
6	-13.883401	25.387754	15.547512
6	-9.165938	20.031978	16.704062
6	-9.476502	16.582606	13.700113
6	-8.614643	15.518572	13.746555
6	-11.622780	22.865044	12.764053
6	-6.105972	15.322417	13.761121
6	-12.092820	27.106951	15.897723
6	-11.032071	25.295880	12.673742
6	-10.622083	23.830908	10.671189
6	-9.409532	20.995518	17.857668
6	-7.928782	19.181396	16.948351
6	-10.399229	19.160976	16.490297
1	-1.921632	22.322290	14.671644
1	-3.113989	23.628022	14.939560
1	-3.418924	21.999292	15.586506
1	-2.040035	22.733123	12.150379
1	-3.626939	22.685615	11.325411
1	-3.226598	24.039253	12.419267
1	-2.513126	20.401074	13.026534
1	-3.942081	20.102573	14.049891
1	-4.151496	20.371654	12.299780
1	-3.918122	27.622926	7.367377
1	-2.388274	25.869617	8.888853
1	-7.293419	26.630011	7.301910
1	-7.281931	27.738269	8.701672
1	-6.330713	28.137491	7.239892
1	-11.431168	27.267779	15.031035
1	-12.930338	27.812336	15.826930
1	-11.536675	27.311983	16.823048
1	-14.628652	26.118176	15.238861
1	-14.761085	23.354555	15.494223
1	-10.356703	26.055454	12.247856
1	-12.066791	25.544973	12.382675
1	-10.958800	25.342772	13.770631
1	-10.347635	22.818374	10.335524
1	-11.611275	24.079537	10.250367
1	-9.882474	24.539484	10.266457
1	-11.719575	22.976430	13.855122
1	-12.624800	22.982206	12.317027
1	-11.264089	21.845431	12.564300
1	-7.111373	16.350390	10.063079

1	-9.653310	15.613576	9.194096
1	-11.854082	16.719686	9.172448
1	-12.113627	17.604589	10.707341
1	-11.830079	18.508315	9.191819
1	-8.779527	14.443706	13.773053
1	-10.565455	16.578866	13.676610
1	-5.477117	15.628723	14.609100
1	-6.327544	14.251697	13.847180
1	-5.550934	15.501928	12.828049
1	-8.528926	21.621967	18.053555
1	-10.254091	21.668194	17.645563
1	-9.634508	20.425514	18.775712
1	-7.039940	19.813275	17.103237
1	-8.065709	18.550623	17.843852
1	-7.732138	18.529487	16.086135
1	-10.597144	18.552360	17.389904
1	-11.287157	19.782633	16.294798
1	-10.257342	18.482705	15.636739
1	-8.200331	21.563691	7.351454
1	-8.679093	20.934407	8.957101
1	-8.130412	22.623134	8.790304
1	-4.516536	21.813737	8.180662
1	-5.738471	22.145744	6.921360
1	-5.686054	23.137637	8.410063
1	-5.266496	19.415918	8.390684
1	-6.978035	19.035183	8.704131
1	-6.482020	19.673293	7.109395
5	-4.789788	25.564337	16.227429
8	-6.084318	25.404694	16.688791
8	-3.872525	25.010095	17.075666
6	-6.036118	24.465130	17.796363
6	-4.554242	24.616901	18.282186
6	-6.338232	23.092529	17.223922
6	-7.077432	24.852613	18.827365
6	-3.932698	23.338442	18.807575
6	-4.368603	25.751151	19.282171
1	-6.356587	22.328029	18.012764
1	-7.327037	23.106523	16.740228
1	-5.610142	22.801200	16.454536
1	-8.090370	24.670080	18.436710
1	-6.959624	24.236424	19.731401
1	-6.998461	25.909896	19.115638
1	-2.897746	23.533772	19.126392
1	-4.493500	22.965004	19.677427

1	-3.911082	22.554864	18.038744
1	-4.802552	25.503136	20.261310
1	-3.292039	25.933012	19.418014
1	-4.828882	26.682633	18.919047
6	-3.441310	29.949903	12.664763
6	-3.834986	28.912804	13.507046
6	-4.652351	27.862004	13.039326
6	-5.073465	27.914042	11.696700
6	-4.679071	28.949926	10.852670
6	-3.859095	29.973831	11.331826
1	-2.811582	30.754985	13.054588
1	-3.526788	28.924791	14.556233
1	-5.728118	27.119322	11.328628
1	-5.023928	28.965077	9.816366
1	-3.559889	30.794480	10.674328
6	-5.148414	26.804580	13.939442
6	-4.362061	26.396351	14.991268
1	-3.311931	26.746358	15.041272
19	-8.232695	26.671399	15.707170
8	-7.801685	27.223673	13.153142
1	-6.836209	27.043480	13.466180
1	-7.750564	27.987360	12.560005

151

Int14, E = -19387.304928, G = 19386.252911

29	-5.407886	24.805599	13.608620
29	-6.292718	23.392828	11.640833
29	-8.251165	21.618857	11.741919
29	-6.817144	19.431386	11.539167
16	-5.481586	22.530390	13.738605
16	-6.135093	21.325445	10.544547
16	-6.982164	25.045568	10.139545
16	-10.083785	20.112097	11.215215
7	-4.263134	25.354675	9.800606
7	-5.510518	26.672003	8.526389
7	-8.165334	18.250037	10.553695
7	-10.189942	17.731774	9.860603
6	-5.501300	25.733503	9.520248
6	-3.664648	22.123553	13.515548
6	-9.428352	18.657001	10.503904
6	-3.452503	26.064798	8.956558
6	-4.204948	26.884588	8.152587
6	-6.501858	21.327406	8.712907
6	-8.113427	17.028083	9.935692
6	-9.366810	16.690318	9.497083

6	-3.551778	20.636252	13.220436
6	-11.615072	17.812926	9.643014
6	-6.677837	27.313122	7.973156
6	-3.097613	22.941377	12.364139
6	-2.968845	22.470322	14.826191
6	-5.520025	22.308738	8.082496
6	-7.936894	21.773043	8.471510
6	-6.269771	19.922574	8.173897
29	-7.638324	25.560706	12.297343
29	-9.320764	23.165395	15.197919
29	-6.836204	20.589910	13.930312
29	-9.235327	19.711464	13.326345
16	-8.848922	23.581172	12.940133
16	-8.790487	20.962000	15.159458
16	-10.367735	24.377650	16.855286
16	-6.079350	18.396670	13.484884
7	-12.581049	22.865393	16.231309
7	-12.933013	25.052453	16.152216
7	-8.757492	17.720378	13.544096
7	-7.390469	15.993652	13.525116
6	-12.022378	24.058108	16.392656
6	-10.563977	24.007863	12.321961
6	-7.481078	17.350578	13.539746
6	-13.879442	23.099318	15.868562
6	-14.121486	24.449085	15.812308
6	-9.064383	19.894176	16.665691
6	-9.505421	16.573116	13.525607
6	-8.670257	15.486975	13.513575
6	-11.519694	22.920141	12.789178
6	-6.167541	15.227619	13.477071
6	-12.696913	26.474198	16.218612
6	-10.963502	25.346465	12.927335
6	-10.512734	24.076495	10.803087
6	-9.066059	20.812069	17.879767
6	-7.947426	18.868170	16.768498
6	-10.421943	19.217804	16.505272
1	-1.890941	22.245666	14.752236
1	-3.087103	23.537277	15.066228
1	-3.383600	21.887182	15.663391
1	-2.029333	22.702628	12.224907
1	-3.631001	22.716832	11.428352
1	-3.177335	24.023708	12.558308
1	-2.495735	20.367425	13.048798
1	-3.922065	20.038791	14.067489

1	-4.137286	20.367942	12.328327
1	-3.940600	27.600772	7.377229
1	-2.367377	25.959657	8.976348
1	-7.318803	26.582887	7.456207
1	-7.266952	27.790712	8.770232
1	-6.358599	28.080278	7.256801
1	-12.154713	26.830066	15.328204
1	-13.661160	26.995529	16.272113
1	-12.109014	26.718494	17.113679
1	-15.011772	25.028899	15.576711
1	-14.577482	22.285820	15.666997
1	-10.249255	26.141725	12.660794
1	-11.971722	25.634062	12.583499
1	-10.991713	25.258705	14.024973
1	-10.209427	23.104206	10.382613
1	-11.506614	24.333773	10.398742
1	-9.790706	24.836776	10.467343
1	-11.593663	22.899714	13.887007
1	-12.529076	23.097355	12.379623
1	-11.179668	21.927861	12.465094
1	-7.182643	16.470751	9.840360
1	-9.745845	15.820005	8.965942
1	-11.930868	16.956247	9.035378
1	-12.154242	17.793623	10.602071
1	-11.870284	18.742152	9.114200
1	-8.861447	14.416229	13.499784
1	-10.594312	16.597508	13.521536
1	-5.515863	15.487760	14.323194
1	-6.415172	14.160464	13.529884
1	-5.624982	15.425988	12.540459
1	-8.071317	21.252221	18.039664
1	-9.783449	21.638323	17.757331
1	-9.333271	20.238299	18.783971
1	-6.968515	19.360726	16.877110
1	-8.106613	18.219133	17.647122
1	-7.914273	18.239457	15.869907
1	-10.639472	18.579615	17.379501
1	-11.222786	19.968905	16.417982
1	-10.438140	18.588313	15.602000
1	-8.158659	21.780175	7.389774
1	-8.646944	21.096075	8.969647
1	-8.093249	22.788951	8.864758
1	-4.477900	21.994940	8.252191
1	-5.688425	22.365798	6.993041

1	-5.650157	23.311827	8.510452
1	-5.236551	19.589910	8.361903
1	-6.952386	19.203711	8.647478
1	-6.442007	19.903405	7.083658
5	-4.916732	25.734245	16.218470
8	-6.215684	25.830281	16.674485
8	-4.066219	25.224686	17.154583
6	-6.267660	25.052791	17.907587
6	-4.787544	25.141973	18.401301
6	-6.659414	23.636879	17.521501
6	-7.285818	25.663430	18.847555
6	-4.302844	23.930754	19.171590
6	-4.496559	26.422292	19.172590
1	-6.759394	23.001088	18.411283
1	-7.631242	23.641790	17.005232
1	-5.926797	23.183276	16.837191
1	-8.309191	25.485370	18.481127
1	-7.211613	25.189660	19.837858
1	-7.134455	26.744907	18.966166
1	-3.261889	24.088731	19.491178
1	-4.916758	23.774728	20.071173
1	-4.336733	23.020926	18.557753
1	-4.951509	26.400489	20.173078
1	-3.407880	26.527372	19.290932
1	-4.870152	27.307009	18.635202
6	-2.380229	28.386882	11.546639
6	-2.945025	27.635695	12.572458
6	-4.336616	27.637265	12.773127
6	-5.141315	28.392784	11.903176
6	-4.575350	29.138397	10.873049
6	-3.191047	29.143607	10.696181
1	-1.295961	28.384302	11.407463
1	-2.295213	27.059179	13.235200
1	-6.222226	28.371782	12.061613
1	-5.217133	29.718916	10.204991
1	-2.742048	29.731014	9.891089
6	-4.993009	26.946601	13.891505
6	-4.363794	26.162929	14.835303
1	-3.287675	25.983954	14.712024
19	-8.614605	26.568809	15.474135
8	-8.062600	27.267338	13.165059
1	-6.051734	27.223930	14.006163
1	-8.227150	27.932338	12.488513

Supplementary References.

1. CrysAlisPro Version 1.171.36.31. Agilent Technologies Inc. Santa Clara, CA, USA (2012).
2. Sheldrick, G. M. A short history of shelx. Acta. Cryst. **A64**, 112–122 (2008).
3. Dolomanov, O. V., Bourhis, L. J., Gildea, R. J., Howard, J. A. -K., Puschmann, H. Olex2: a complete structure solution, refinement and analysis program. J. Appl. Cryst. **42**, 339–341 (2009).
4. Sheldrick, G. M. Crystal structure refinement with shelxl. Acta. Cryst. **C71**, 3–8 (2015).
5. Jia, T., Li, Y.-X., Ma, X.-H., Zhang, M.-M., Dong, X.-Y., Ai, J. & Zang, S.-Q. Atomically precise ultrasmall copper cluster for room-temperature highly regioselective dehydrogenative coupling. Nat Commun **14**, 6877 (2023).
6. Zi, W. & Toste F. D. Gold(I)-catalyzed enantioselective carboalkoxylation of alkynes. J. Am. Chem. Soc. **135**, 12600–12603 (2013).
7. Adamo, C. & Barone, V. Toward reliable density functional methods without adjustable parameters: The PBE0 model. J. Chem. Phys. **110**, 6158–6170 (1999).
8. Perdew, J. P., Ernzerhof, M. & Burke, K. Rationale for mixing exact exchange with density functional approximations. J. Chem. Phys. **105**, 9982–9985 (1996).
9. Schäfer, A., Huber, C. & Ahlrichs, R. Fully optimized contracted Gaussian basis sets of triple zeta valence quality for atoms Li to Kr. J. Chem. Phys. **100**, 5829–5835 (1994).
10. Schäfer, A., Horn, H. & Ahlrichs, R. Fully optimized contracted Gaussian basis sets for atoms Li to Kr. J. Chem. Phys. **97**, 2571–2577 (1992).
11. Weigend, F. Accurate coulomb-fitting basis sets for H to Rn. Phys. Chem. Chem. Phys. **8**, 1057–1065 (2006).
12. Weigend, F. & Ahlrichs, R. Balanced basis sets of split valence, triple zeta valence and quadruple zeta valence quality for H to Rn: design and assessment of accuracy. Phys. Chem. Chem. Phys. **7**, 3297–3305 (2005).
13. Marenich, A. V., Cramer, C. J. &Truhlar, D. G. Universal solvation model based on solute electron density and on a continuummodel of the solvent defined by the bulk dielectric constant and atomic surface tensions. J. Phys. Chem. B. **113**, 6378–6396 (2009).
14. Grimme, S. Semiempirical GGA-type density functional constructed with a long-range dispersion correction. J. Comput. Chem. **27**, 1787–1799 (2006).
15. Grimme, S. Semiempirical hybrid density functional with perturbative second-order correlation. J. Chem. Phys. **124**, 034108 (2006).
16. Fukui, K. Formulation of the reaction coordinate. J. Phys. Chem. **74**, 4161–4163 (1970).
17. Maeda, S., Harabuchi, Y., Ono, Y., Taketsugu, T. & Morokuma, K. Intrinsic reaction coordinate: calculation, bifurcation, and automated search. Int. J. Quantum Chem. **115**, 258–269 (2015).
18. Fukui, K. The path of chemical reactions–the IRC approach. Acc. Chem. Res. **14**, 363–368 (1981).

19. Frisch, M. J. & Trucks, G. W. et al. Gaussian 16 Rev. C.01, Wallingford, CT, (2016).
20. Nakajima, K., Kato, T. & Nishibayashi, Y. Hydroboration of alkynes catalyzed by pyrrolide-based PNP pincer−iron complexes. Org. Lett. **19**, 4323−4326 (2017).
21. Liu Z.-H., Wei, W., Xiong, L., Feng, Q., Shi, Y.-J., Wang, N.-Y. & Yu, L.-T. Selective and efficient synthesis of transarylvinylboronates and trans-hetarylvinylboronates using palladium catalyzed cross-coupling. New J. Chem. **41**, 3172−3176 (2017).
22. Zhong, M.-B., Gagné, Y., Hope, T. Q., Pannecoucke, X., Frenette, M., Jubault, P. & Poisson, T. Copper-photocatalyzed hydroboration of alkynes and alkenes. Angew. Chem. Int. Ed. **60**, 14498–14503 (2021).
23. Lu, W.-K. & Shen, Z.-M. Direct synthesis of alkenylboronates from alkenes and pinacol diboron via copper catalysis. Org. Lett. **21**, 142–146 (2019).
24. Zhang, G.-Q., Zeng, H.-S., Zheng, S.-P., Neary, M. C. & Dub, P. A. Vanadium-catalyzed stereo- and regioselective hydroboration of alkynes to vinyl boronates. ACS Catal. **12**, 5425-5429 (2022).
25. Aelterman, M., Sayes, M., Jubault, P. & Poisson, T. Electrochemical hydroboration of alkynes. Chem. Eur. J. **27**, 8277 –8282 (2021).
26. Tao, S., Wang, Y., Pan, Q.-X., Zhao, J.-X., Bu, Q.-Q., Chen, F., Liu, J.-C., Dai, B., Wei, D.-H. & Liu, N. Aqueous hydroboration of alkynes via nonclassical generation of N-heterocyclic carbenes. Green Chem. **25**, 6704–6716 (2023).
27. Bismuto, A., Thomas, S. P. & Cowley. M. J. Aluminum hydride catalyzed hydroboration of alkynes. Angew. Chem. Int. Ed. **55**, 15356–15359 (2016).
28. Magre, M., Maity, B., Falconnet, A., Cavallo, L. & Rueping, M. Magnesium-catalyzed hydroboration of terminal and internal alkynes. Angew. Chem. Int. Ed. **58**, 7025–7029 (2019).
29. Wei, R. J. et al. Ultrathin metal-organic framework nanosheets exhibiting exceptional catalytic activity. J. Am. Chem. Soc. **144**, 17487–17495 (2022)
30. Zhang, J. et al. Tuning polarity of Cu-O bond in heterogeneous Cu catalyst to promote additive-free hydroboration of alkynes. Chem. **6**, 725−737 (2020).
31. Chen, J., Shen, X. & Lu, Z. Cobalt-catalyzed markovnikov-type selective hydroboration of terminal alkynes. Angew. Chem. Int. Ed. **60**, 690–694 (2021).
32. Zhong, M. et al. Copper-photocatalyzed hydroboration of alkynes and alkenes. Angew. Chem. Int. Ed. **60**, 14498–14503 (2021).
33. Zeng, X. H. et al. Efficient heterogeneous hydroboration of alkynes: enhancing the catalytic activity by Cu(0) incorporated $CuFe_2O_4$ nanoparticles. New J. Chem. **42**, 17346–17350 (2018).
34. Magre, M., Maity, B., Falconnet, A., Cavallo, L. & Rueping, M. Magnesium-catalyzed hydroboration of terminal and internal alkynes. Angew. Chem. Int. Ed. **58**, 7025–7029 (2019).
35. Zhao, J. et al. Ligand-free hydroboration of alkynes catalyzed by heterogeneous copper powder with high efficiency. Chem. Commun. **50**, 2058–2060 (2014).
36. Xu, S., Zhang, Y., Li, B. & Liu, S. Y. Site-selective and stereoselective trans-hydroboration of 1,3-enynes catalyzed by 1,4-azaborine-based phosphine-Pd complex. J. Am. Chem. Soc. **138**, 14566−14569 (2016).

37. Bismuto, A., Thomas, S. P. & Cowley, M. J. Aluminum hydride catalyzed hydroboration of alkynes. Angew. Chem. Int. Ed. **55**, 15356–15359 (2016).
38. Obligacion, J. V., Neely, J. M., Yazdani, A. N., Pappas, I. & Chirik, P. J. Cobalt catalyzed Z-selective hydroboration of terminal alkynes and elucidation of the origin of selectivity. J. Am. Chem. Soc. **137**, 5855−5858 (2015)
39. Ojha, D. P. & Prabhu, K. R. Pd-catalyzed hydroborylation of alkynes: a ligand controlled regioselectivity switch for the synthesis of α- or β-vinylboronates. Org. Lett. **18**, 432–435 (2016).
40. Gao, Y. et al. Cyclic (alkyl)(amino)carbene ligands enable Cu-catalyzed markovnikov protoboration and protosilylation of terminal alkynes: a versatile portal to functionalized alkenes. Angew. Chem. Int. Ed. **60**, 19871–19878 (2021).
41. Duan, H., Chen, X., Yang, Y.-N., Zhao, J., Lin, X.-C., Tang, W.-J., Gao, Q., Ning, G.-H. J. & Li, D. Tailoring stability, catalytic activity and selectivity of covalent metal–organic frameworks via steric modification of metal nodes. J. Mater. Chem. A. **11**, 12777–12783 (2023).
42. Alamer, B., Sagadevan, A., Bodiuzzaman, M., Murugesan, K., Alsharif, S., Huang, R.-W., Ghosh, A., Naveen, M. H., Dong, C., Nematulloev, S., Yin. J., Shkurenko, A., Abulikemu, M., Dong, X., Han, Y., Eddaoudi, M., Rueping M. & Bakr, O. M. Planar core and macrocyclic shell stabilized atomically precise copper nanocluster catalyst for efficient hydroboration of C–C multiple bond. J. Am. Chem. Soc.**146**, 16295−16305 (2024).
43. Wen, J., Huang, Y., Zhng, Y., Grützmacher, H. & Hu, P. Cobalt catalyzed practical hydroboration of terminal alkynes with time-dependent stereoselectivity. Nat Commun. **15**, 2208 (2024).
44. Zhang, Z., Ma, X., Li, Y., Ma, N., Wang, M., Liu, W., Peng, J., Liu, Y. & Li, Y. Heterovalent Metal Pair Sites on Metal–Organic Framework Ordered Macropores for Multimolecular Co-Activation. J. Am. Chem. Soc. **146**, 8425–8434 (2024).
45. Khan, A., Asiri, A. M., Kosa, S. A., Garcia, H. & Grirrane, A. Catalytic stereoselective addition to alkynes. Borylation or silylation promoted by magnesia-supported iron oxide and cis-diboronation or silaboration by supported platinum nanoparticles. J. Catal. **329**, 401–412 (2015).
